# Oxalyl amide assisted palladium-catalyzed synthesis of pyrrolidones *via* carbonylation of γ-C(sp^3^)–H bonds of aliphatic amine substrates†
†Electronic supplementary information (ESI) available: Experimental procedures, characterization data for all new compounds. CCDC 1048649. For ESI and crystallographic data in CIF or other electronic format see DOI: 10.1039/c5sc00519a


**DOI:** 10.1039/c5sc00519a

**Published:** 2015-05-19

**Authors:** Chao Wang, Li Zhang, Changpeng Chen, Jian Han, Yingming Yao, Yingsheng Zhao

**Affiliations:** a Key Laboratory of Organic Synthesis of Jiangsu Province College of Chemistry , Chemical Engineering and Materials Science Soochow University , Suzhou 215123 , PR China . Email: yszhao@suda.edu.cn

## Abstract

The first Pd-catalyzed regioselective γ-carbonylation of oxalyl amide protected aliphatic amines with carbon monoxide leading to synthesis of pyrrolidones has been developed. Both γ-methyl and cyclopropyl methylene C–H bonds are well activated to obtain the corresponding pyrrolidones in moderate to excellent yields. The role of 3-(trifluoromethyl)benzoic acid as an additive is critical as it helps in stabilizing the palladium intermediate formed during the catalytic cycle. The reaction scope is extended to benzylamine and allyl amine derivatives, thereby affording the corresponding products in good to excellent yields.

## Abstract 2

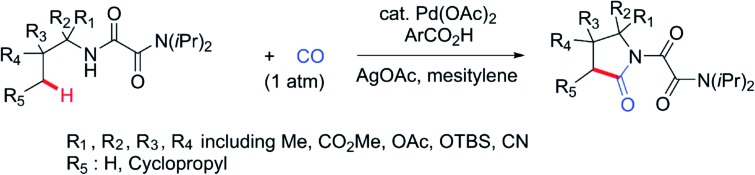
The first Pd-catalyzed regioselective γ-carbonylation of oxalyl amide protected aliphatic amines with carbon monoxide leading to synthesis of pyrrolidones has been developed.

## Introduction

Recently, the development of practical methods for the construction of synthetically useful building blocks through direct C–H functionalization has emerged as an attractive and fruitful area in modern organic chemistry.^1^ Among these methods, the transition-metal-catalyzed carbonylation of aryl C(sp^2^)–H bonds using CO as a carbon atom source to form an easily transformable carbonyl group has experienced landmark development.^2^–^5^ The first example was in the 1950s when Murahashi reported the cobalt-catalysed direct C–H functionalization/carbonylation of arenes with CO.^6^ In 1980, Fujiwara *et al.* also described a protocol involving Pd(OAc)_2_-catalyzed direct carboxylation of some aromatic compounds with CO (15 atm) in an autoclave to give the corresponding carboxylic acids in poor to moderate yields.^7^ However, large excess of the arenes and lack of regioselectivity has hampered further application of this method in organic synthesis.

**Scheme 1:**
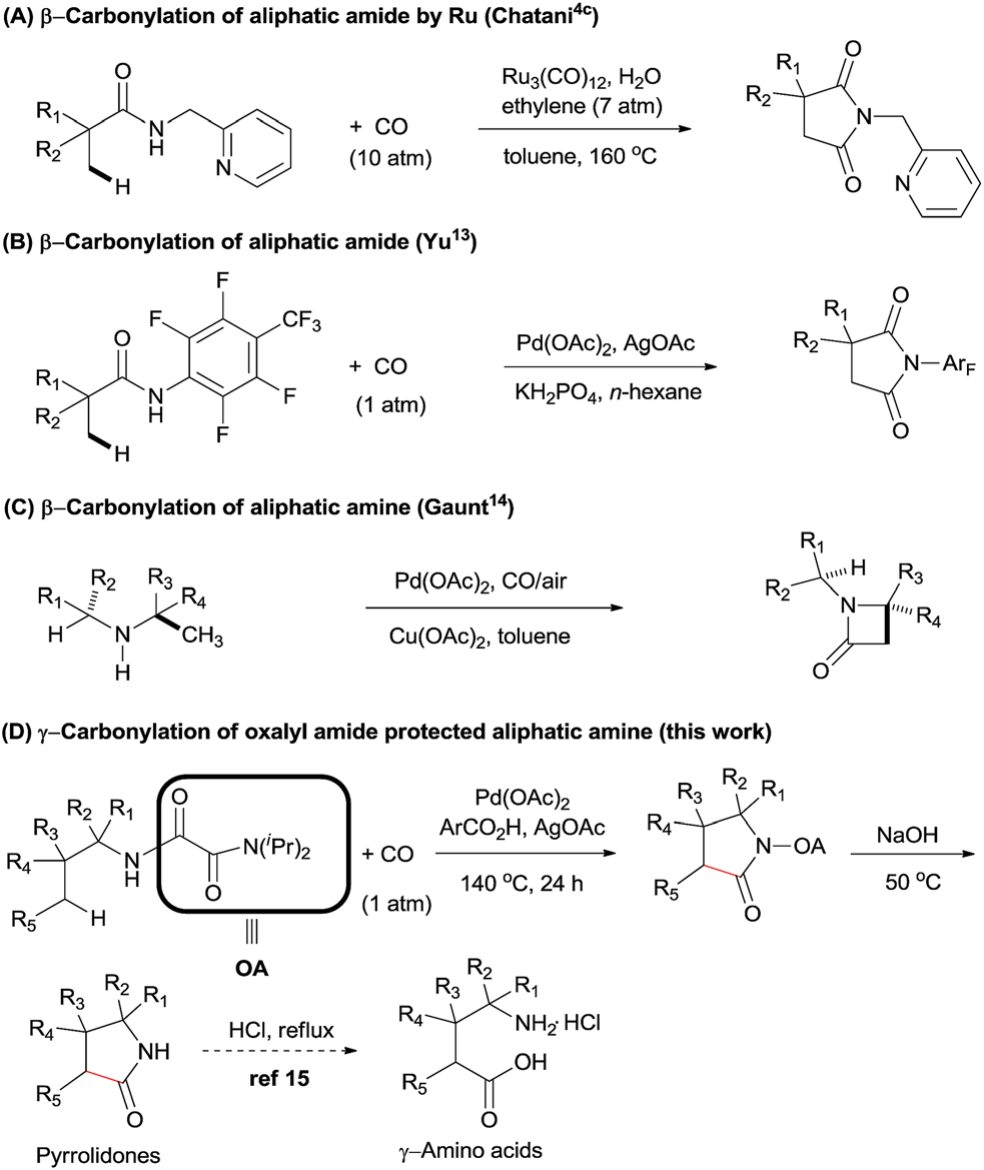
Carbonylation of C(sp^3^)–H Bonds.

In the last decades, the challenge of selectivity in carbonylation of C(sp^2^)–H bonds has been overcome by the employment of directing groups,^8^ or where regioselectivity could also be achieved based on the substrates own intrinsic reactivity, *e.g.* amines,4d,8d,^9^ carboxylics,^10^ phenols,^11^ and alcohols.^12^ In spite of these great developments in the carbonylation of arenes, there is still limited success in the direct functionalization of C(sp^3^)–H bonds with CO. In 2011, Chatani *et al.* described the Ru(0)-catalyzed regioselective carbonylation of unactivated C(sp^3^)–H bonds of aliphatic amides using bidentate directing groups (Scheme 1A). Similarly, Yu *et al.* reported the synthesis of succinimides *via* a Pd(ii)-catalyzed β-carbonylation of aliphatic amide with CO (Scheme 1B).^13^ Recently, Gaunt *et al.* described a Pd-catalyzed carbonylation of secondary aliphatic amines at the β-position *via* a rarely reported four-membered palladacycle, leading to β-lactams in moderate to good yields (Scheme 1C).^14^ To the best of our knowledge, only these two examples of Pd-catalyzed carbonylation of C(sp^3^)–H bonds at specific positions have been reported. We speculated that during the catalytic cycle, Pd(ii) might be easily reduced to Pd(0), thereby undergoing β-hydride elimination, and that there could be a competitive coordination with the palladium centre between CO and the specific C–H bond under CO atmosphere.^15^ For these reasons, the direct carbonylation of C(sp^3^)–H bonds remained a significant challenge.

In 2005, Daugulis and co-workers reported the first use of picolinamide and aminoquinoline auxiliaries as bidentate directing groups in Pd-catalyzed regioselective functionalization of C(sp^3^)–H bonds.^16^ Later, several groups demonstrated that these groups have an immense ability for enabling a wide variety of C–H transformation.^17^ Inspired by these results, our group has developed another bidentate directing group, oxalyl amide, which could be easily synthesized from oxalyl chloride and diisopropylamine *via* an S_N_ type reaction. We found that it could be used as a powerful directing group in enabling a variety of C–H transformations for both unactivated C(sp^3^)–H and C(sp^2^)–H bonds.^18^

In order to expand the reaction scope of oxalyl amide assisted C–H functionalization and explore its potential application towards transformation not achievable by other auxiliary-assisted C–H functionalization, we therefore proceeded to address the unreported γ-carbonylation of aliphatic amine substrates in a bid to synthesize important structural motifs of pyrrolidones.^19^ Herein, we report a novel protocol for the preparation of pyrrolidones by Pd(ii)-catalyzed γ-carbonylation of oxalyl amide protected aliphatic amine substrates. Both γ-methyl and cyclopropyl methylene C–H bonds were well activated and gave the corresponding pyrrolidones in moderate to excellent yields. Furthermore, pyrrolidones could be transformed into synthetically useful γ-amino acids under acidic conditions,^20^ which offers a potential route towards synthesising unnatural amino acids (Scheme 1D). The substrate scope could also be further extended to oxalyl amide protected benzyl amine and allyl amine derivatives, affording the corresponding valuable synthons in good to excellent yields.

## Results and discussion

We started our investigation with the reaction of oxalyl amide protected **1a** in the presence of Pd(OAc)_2_ catalyst and silver acetate as oxidant under atmospheric pressure of CO at 140 °C in mesitylene for 24 h. Gratifyingly, the desired pyrrolidone **2a** was obtained in 22% yield. A variety of additives, such as AcOH, PivOH, (^*n*^BuO)_2_PO_2_H, Ac-Gly-OH, which are well known to improve the reactivity of a palladium center, were applied to this reaction,^21^ with all of them displaying different levels of positive effect on the product yield. Pivalic acid greatly aided the conversion of **1a**, affording **2a** in 57% yield, but benzoic acid had a better promoting effect. However, further screening revealed that 3-(trifluoromethyl)benzoic acid was the most effective additive, with an excellent conversion of **1a** giving 85% yield of **2a**. During optimization studies, reactions with various different additives proceeded cleanly and it was observed that Pd(ii) was reduced to Pd(0) during the reaction thus rationalizing the low efficiency of some of these additives (Table 1, entries 2–8). Although the reason behind the promoting effect of 3-(trifluoromethyl)benzoic acid was not clear, one plausible explanation was that the palladium(0) stabilized by the electron-deficient arene during the catalytic cycle, could be further reoxidized to Pd(ii). Meanwhile, it played the same role as pivalic acid during the C–H activation step.^22^ Other oxidants such as Cu(OAc)_2_ or BQ were not effective in this transformation. Notably, alternative directing groups or protecting groups, such as Cbz, triflamide and picolinamide were also tested under optimized conditions. However, none could give the corresponding carbonylated products in more than 5% yield analyzed by LC-MS.

**Table 1:** Optimization of palladium-catalyzed carbonylation of γ-C(sp^3^)–H bonds

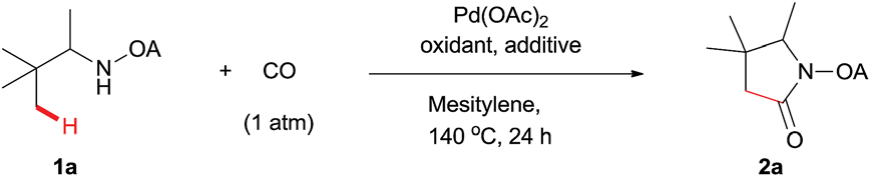					
Entry	Pd(OAc)_2_ (mol%)	Oxidant (2.5 equiv.)	Additive (0.3 equiv.)	Conv^*a*^ (%)	Yield^*a*^ ^,^^*b*^ (%)
1	10	AgOAc		26	22
2	10	AgOAc	AcOH	21	19
3	10	AgOAc	PivOH	60	57
4	10	AgOAc	(^*n*^BuO)_2_PO_2_H	66	62
5	10	AgOAc	Ac-Gly-OH	12	8
6	10	AgOAc	PhCO_2_H	76	75
7	10	AgOAc	*o*-PhPhCO_2_H	27	18
8	10	AgOAc	*m*-CF_3_PhCO_2_H	87	85(83)
9	10	Ag_2_CO_3_	*m*-CF_3_PhCO_2_H	21	20
10	10	Cu(AOc)_2_	*m*-CF_3_PhCO_2_H	2	0
11	10	BQ	*m*-CF_3_PhCO_2_H	5	4
12	0	AgOAc	*m*-CF_3_PhCO_2_H	2	0
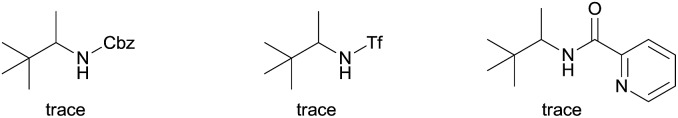					

^*a*^Reactions were carried out at 0.2 mmol scale under CO (1 atm), using mesitylene (0.3 mL) as the solvent; conversion and yield were based on GC using tridecane as the internal standard.

^*b*^Isolated yield in parentheses. Ac = acetyl, Gly = glycine.

With the optimized conditions in hand, reactions involving a series of oxalyl amide protected propylamine derivatives were carried out. The carbonylation of substrates bearing two β-substituents proceeded smoothly to provide the corresponding pyrrolidines in good to excellent yields (Table 2, **2b–f**). Ester derivatives were tolerated, giving the corresponding products in good yields (**2b,c,f,h,i,n,o**). Substrates **1g–k**, bearing only one β methyl group, were less reactive and acceptable yields could only be obtained by slightly modifying the reaction conditions. Delightfully, substrate **1j**, with an α-substituted cyano group, which is a highly valuable synthon in synthetic chemistry, also gave the desired carbonylated product **2j** in 56% yield. The methylene C–H bonds in cyclopropane rings were all carbonylated giving good yields of products (**2l–n**). Interestingly, substrate **1o** gave a mixture of five and six-membered carbonylated products in a total of 75% yield (**2o**), which could be easily separated by silica gel chromatography. Inspired by these result, substrate **1p** was also tested, but a lower yield of product than expected was recovered, together with unreacted starting material. It was thought that the probable competitive carbonylation reaction happening at the aromatic ring was not detected by NMR and GC-MS.^23^ However, when one equivalent of Pd(OAc)_2_ was used, the δ-carbonylated product **2p** was obtained in 72% yield, along with 19% yield of intramolecular aminated product **2p-1**. Unfortunately, when the same reaction was attempted on substrate **1q**, only 12% of the corresponding carbonylated product **2q** could be isolated along with 8% of the product from intramolecular amination. Regrettably, the use of one equivalent of Pd(OAc)_2_ as in the case of substrate **1p**, did nothing to improve the yield of carbonylation and intramolecular amination products. However, it can be inferred from these results that an unstable alkyl seven-membered palladacycle^24^ might have been formed during the catalytic cycle, which could open doors for the development of newer transformation. A series of other oxalyl amide protected amines (**1r–u**) were also tested, all failing to give acceptable yields.

**Table 2:** Palladium-catalyzed carbonylation of γ-C(sp^3^)–H bonds^*a*^

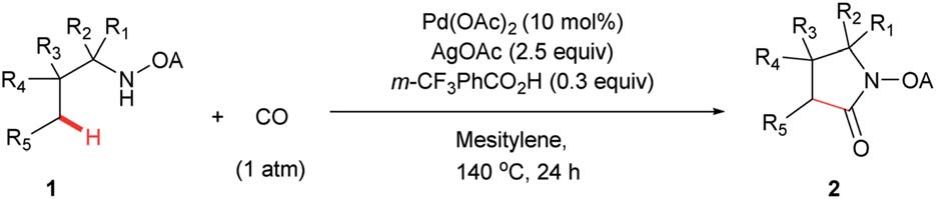
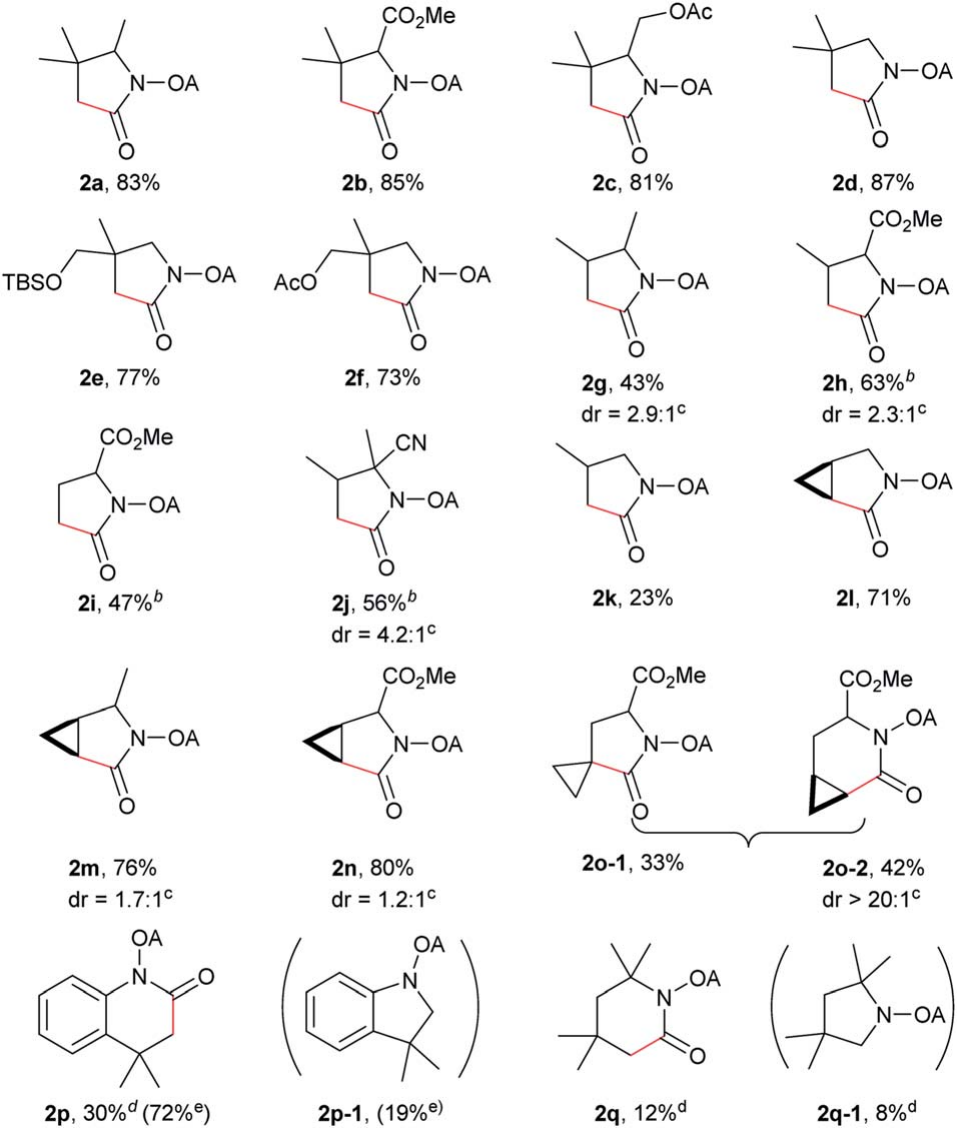
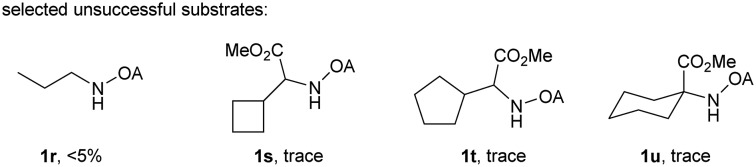

^*a*^Yields based on isolated products on a 0.2 mmol scale under CO (1 atm), using mesitylene (0.3 mL) as the solvent.

^*b*^15 mol% Pd(OAc)_2_ was used.

^*c*^Determinated by H NMR analysis.

^*d*^At 100 °C.

^*e*^1 equiv. Pd(OAc)_2_, without AgOAc.

Despite the great development in palladium-catalyzed C(sp^2^)–H activation/carbonylation,^25^ there have been only few examples on the direct preparation of benzolactams from benzylamines substrates assisted by directing groups. Encouraged by the results obtained above, oxalyl amide protected benzylamines were also examined, and representative data were listed in Table 3. A wide variety of functional groups, such as CF_3_, OMe, Br, vinyl, cyano, were compatible with this protocol, affording the corresponding products in good to excellent yields. Both electron-donating and electron-withdrawing substituents on the phenyl ring had little or no effect on the yield of products. Interestingly, substrates **3d** and **3e** needed slightly higher temperature for complete transformation.

**Table 3:** Palladium-catalyzed carbonylation of γ-C(sp^2^)–H bonds^*a*^

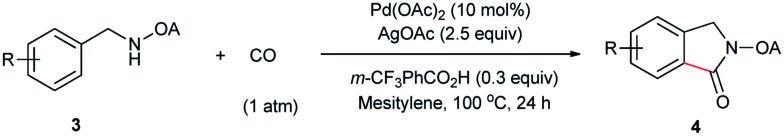
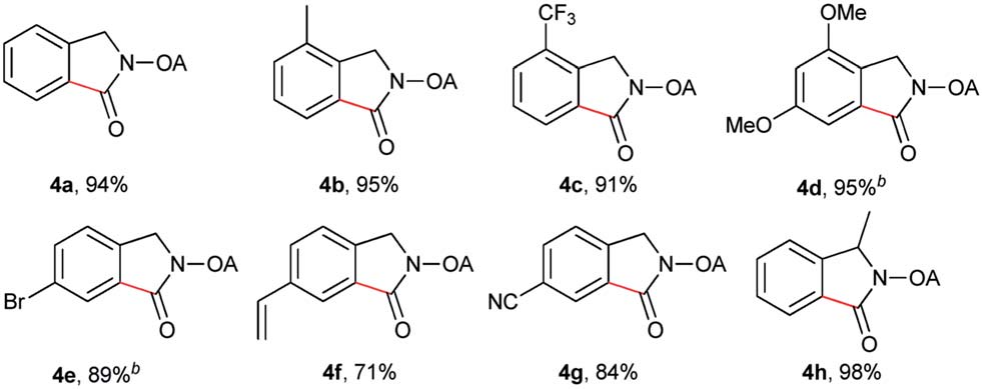

^*a*^Yields based on isolated products on a 0.2 mmol scale under CO (1 atm), using mesitylene (0.3 mL) as the solvent.

^*b*^At 120 °C.

**Scheme 2:**
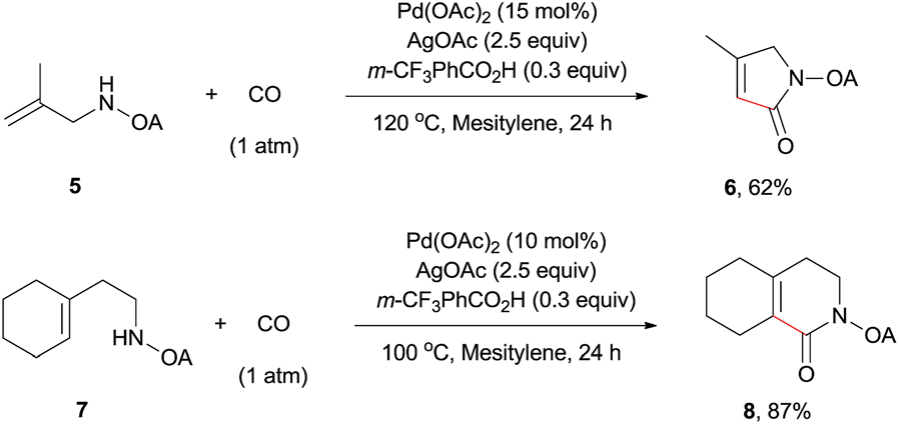
Carbonylation of allylamine reaction.

The scope of γ-carbonylation of oxalyl amide protected primary amine could be further extended to allylamine substrates. Substrates **5** and **7** smoothly reacted with CO to give the corresponding carbonylated products **6** and **8** in acceptable yield respectively, which could be used as synthons for a variety of further elaborations (Scheme 2).^26^

**Scheme 3:**
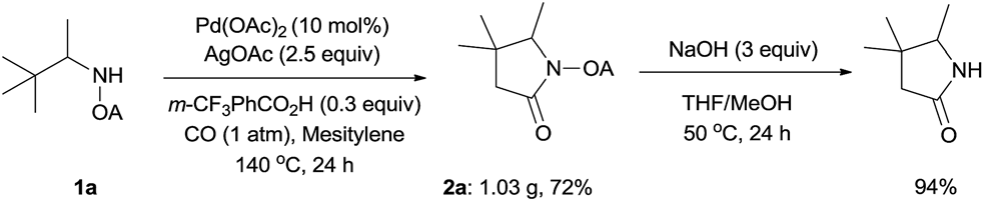
Scaling up and auxiliary removal.

To demonstrate the powerful use of this protocol, a gram scale reaction involving substrate **1a** was carried out and the corresponding pyrrolidone was isolated in 72% yield (Scheme 3). The oxalyl amide directing group could be removed under basic conditions at 50 °C, thus underlining the potential utility of this newly developed synthetic method.

## Conclusion

In conclusion, we have developed the first Pd-catalyzed γ-carbonylation of oxalyl amide protected aliphatic amine substrates by using oxalyl amide as a bidentate directing group with AgOAc, and ArCO_2_H under carbon monoxide atmosphere. This method is a novel strategy for the preparation of pyrrolidones. The substrate scope can be extended to benzylamine and allyl amine derivatives, giving products in good to excellent yields. The use of 3-(trifluoromethyl)benzoic acid is important for this transformation, and a detailed mechanistic study is now being undertaken in our laboratory.

## Supplementary Material

Supplementary information

Supplementary information

Crystal structure data
