# Supplementary material for: Oxalyl amide assisted palladium-catalyzed synthesis of pyrrolidones via carbonylation of γ-C(sp3)–H bonds of aliphatic amine substrates
Source: Chem Sci. 2015 May 19;6(8):4610–4. doi: 10.1039/c5sc00519a (PMC5861525; doi:10.1039/c5sc00519a)
Supplement: Supplementary file 1 [file SC-006-C5SC00519A-s001.pdf]

Electronic Supplementary Material (ESI) for Chemical Science.
This journal is © The Royal Society of Chemistry 2015

Oxalyl Amide Assisted-Palladium-Catalyzed Synthesis of Pyrrolidones via Carbonylation of γ-C(sp^3)–H Bonds of Aliphatic Amine Substrates

Chao Wang, Li Zhang, Changpeng Chen, Jian Han, Yingming Yao* and Yingsheng Zhao*

College of Chemistry, Chemical Engineering and Materials Science, Soochow University, Suzhou 215123, P. R. China

Supporting information

Table of Contents

1. Reagents **S2**

2. Instruments **S2**

3. Mechanism study **S2**

4. Optimization of reaction conditions **S4**

5. Carbonylation with equivalent of $Pd(OAc)_2$ **S4**

6. Gram scale reaction **S5**

7. Removal of the directing group **S5**

8. Preparation of other directing groups protected aliphatic amine **S6**

9. Preparation of oxalamide substrates **S7**

10. Palladium-catalyzed carbonylation of γ-C(sp^3)–H bonds **S16**

11. Palladium-catalyzed carbonylation of γ-C(sp^2)–H bonds **S23**

12. Allylamine reaction (Scheme 2) **S25**

13. References **S26**

Appendix (Proton and Carbon NMR Spectra) **S27**

1. Reagents: Unless otherwise noted, all reagents were purchased from commercial suppliers and used without further purification. Column chromatography purifications were performed using 300–400 mesh silica gel.

2. Instruments: NMR spectra were recorded on Varian Inova–400 MHz, Inova–300 MHz, Bruker DRX–400 or Bruker DRX–500 instruments and calibrated using residual solvent peaks as internal reference. Multiplicities are recorded as: s = singlet, d = doublet, t = triplet, dd = doublet of doublets, br = broad singlet, m = multiplet. HRMS analyses were carried out using a Bruker micrOTOF–Q instrument or a TOF–MS instrument.

3. Mechanism study

3.1. NMR tube reaction

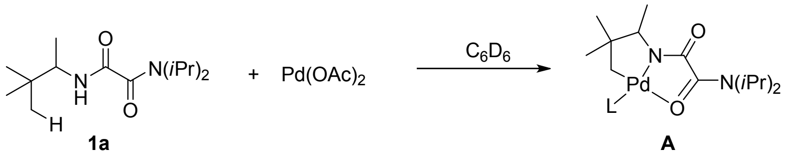

A mixture of **1a** (10.2 mg, 0.04 mmol, 1.0 eq), $Pd(OAc)_2$ (9.0 mg, 0.04 mmol, 1 equiv) and Benzene–d_6 (0.5 mL) in NMR tube was heated in an oil bath at 60 °C and 100 °C respectively for 10 h. The reaction mixtures were cooled to room temperature and further analyzed by ^{1}H NMR. From the NMR analysis we can speculated that the C-H activation could be happened at 100 °C. Thus, the palladium intermediate **A** might be formed during the catalytic cylcle. However, the palladium black was observed in the NMR tube reactions at 100 °C. We had tried to added nitrile compounds or benzoic acid derivaties, no one of them could prevent the decompsition of the palladium intermediates.

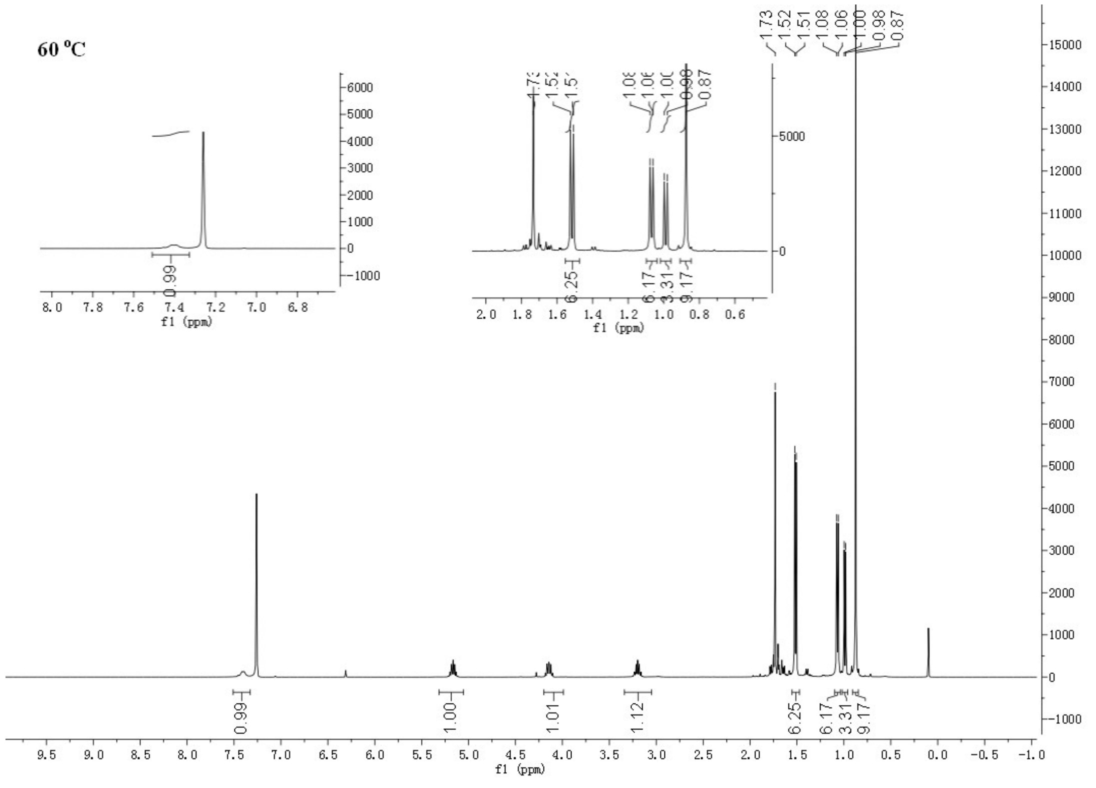

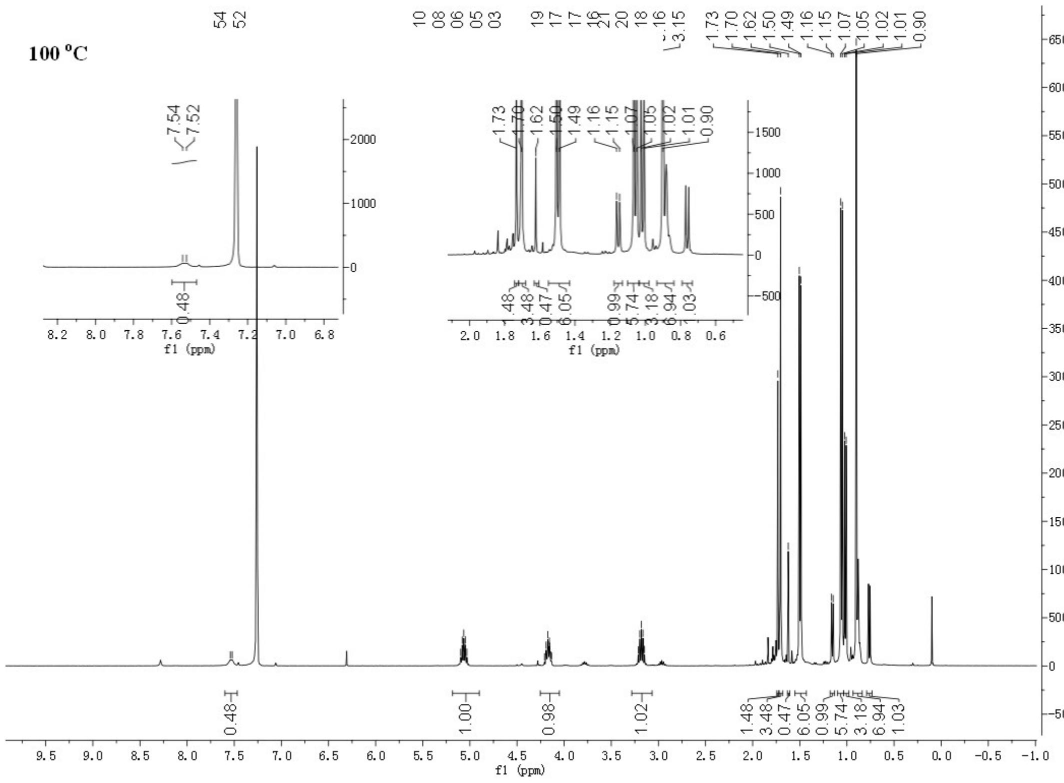

3.2. Reaction of Oxalyl Amide protected *tert*-Butylamine

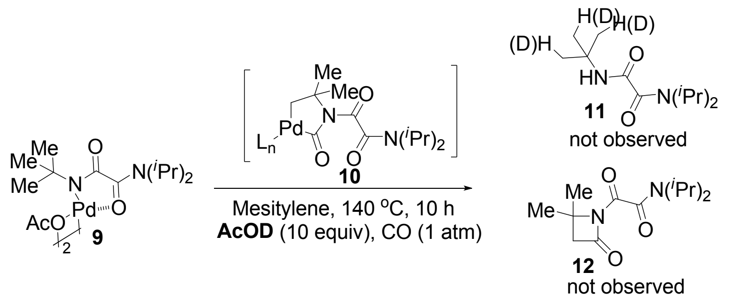

Oxalyl amide protected *tert*-butylamine**,** which lack γ C-H bonds, was treated with $Pd(OAc)_2$ (1 equiv), AcOD (10 equiv) under CO atmosphere in mesitylene at 140 °C for 10 h. If the carbon monoxide could insert into Pd–N bonds, the complex **10** might be formed. Then, either **11** or **12** could be formed through reductive elimination from the complex **10**. However, proton NMR analysis revealed that neither **11** nor **12** were generated. Based on this result, the palladium intermediate **B** might be the key intermediate druing the catalytic cycle, the palladium intermediate **10** could not be formed in the catalytic cycle.

3.3. Proposed Mechanism

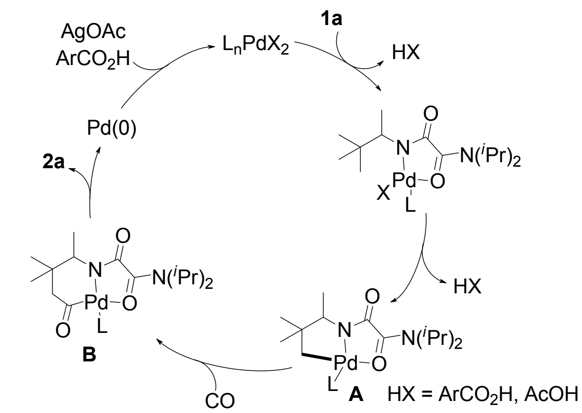

Although the mechanistic details were unclear, based on our previously studies and pioneering reports,[1] we proposed a plausible mechanism for this γ-C(sp^3)–H carbonylation reaction. The palladium complex **A** could be generated through a concerted metalation-deprotonation (CMD) pathway. One molecule of CO combined with Pd(II) center, followed by a 1,1-migratory insertion of CO into the Pd-C bonds, giving the key palladium intermediate **B**. The six-membered palladacycle **B** then underwent reductive elimination affording the desired product.

4. Optimization of reaction conditions

Table S1. Optimization of Reaction Conditions

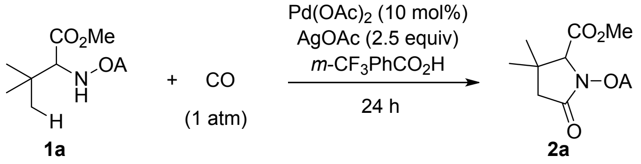

Entry	m-CF_3PhCO_2H (equiv)	Temp. (°C)	Solvent	Yield of **2a** (%)[a]
1	0.3	140	*m*-xylene	38
2	0.3	140	DCE	80
3	0.3	140	HFIP	69
3	0.3	140	mesitylene	87
4	0.2	140	mesitylene	82
5	0.5	140	mesitylene	85
6	0.3	120	mesitylene	52

[a]Reactions were carried out on a 0.2 mmol scale under CO (1 atm), using 0.3 mL solvent; yield was based on GC using tridecane as the internal standard.

5. Carbonylation with equivalent of $Pd(OAc)_2$

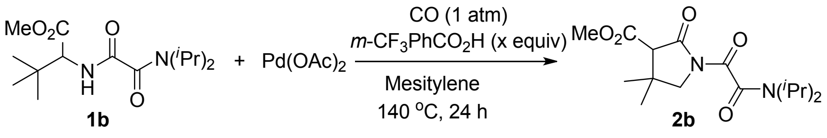

a x= 0, yield of **2b**: 36%
b x= 1, yield of **2b**: 44%

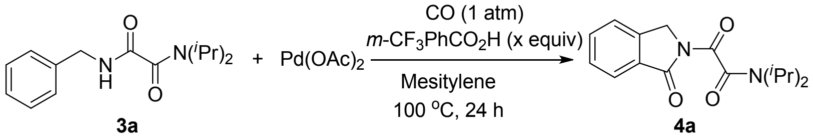

a x= 0, yield of **4a**: 84%
b x= 1, yield of **4a**: 82%

6. Gram scale reaction

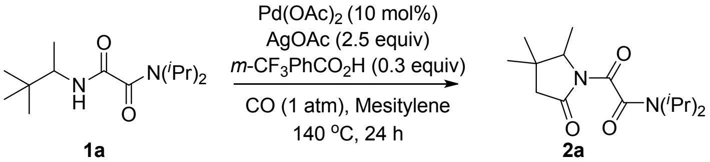

1a **1.28 g, 5 mmol,** **2a** **1.03 g, 72%**

A 20 mL Schlenk-type tube (with a Teflon high pressure valve and side arm) was charged with **1a** (1.28 g, 5 mmol, 1.0 eq), $Pd(OAc)_2$ (112.2 mg, 0.1 eq), AgOAc (2g, 2.5 eq), *m*-CF_3PhCO_2H (28.5 mg, 0.3 eq) and mesitylene (1.5 mL). The reaction tube was evacuated and back-filled with CO (3-times), and connected with a 100 mL CO balloon. The vial was heated at 140 °C in an oil bath for 24 hours, then the reaction mixture was cooled to rt, and concentrated in vacuo. The resulting residue was purified by column chromatography on silica gel to give **2a** as white solid in 72% yield.

7. Removal of the Directing Group[2]

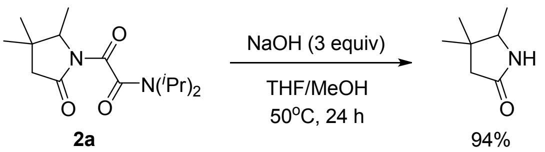

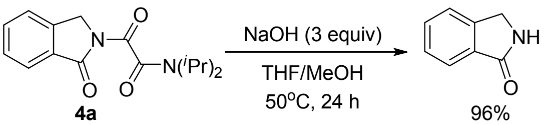

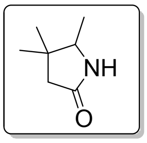

Compound **2a** (55.3 mg, 0.2 mmol, 1.0 eq) was dissolved in a mixture of THF/MeOH (0.8/0.2 mL); NaOH (24 mg, 0.6 mmol, 3.0 eq) was then added. The mixture was heated to 50 °C and stirred for 24 hours. Water was added and the mixture was extracted with DCM. The combined organic layers was washed with water and brine, dried over anhydrous Na_2SO_4, and concentrated in vacuo. The residue was purified by column chromatography on silica gel to give the product in

94% yield. ^1H NMR (400 MHz, $CDCl_3$) δ 6.21 (br, 1H), 3.39 (m, 1H), 2.21-2.07 (m, 2H), 1.15-1.05 (m, 6H), 0.99 (s, 3H); ^{13}C NMR (101 MHz, $CDCl_3$) δ 177.28, 59.21, 45.98, 38.59, 27.11, 22.51, 15.61; HRMS Calcd for $C_7H_{13}NNaO$ [M+Na$^+$]: 150.0895; Found: 150.0896.

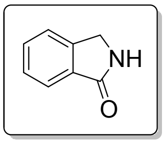

Compound **4a** (55.3 mg, 0.2 mmol, 1.0 eq) was dissolved in a mixture of THF/MeOH (0.8/0.2 mL); NaOH (24 mg, 0.6 mmol, 3.0 eq) was then added. The mixture was heated to 50 °C and stirred for 24 hours. Water was added and the mixture was extracted with DCM. The combined organic layers was washed with water and brine, dried over anhydrous Na_2SO_4, and concentrated in vacuo. The residue was purified by column chromatography on silica gel to give the product in 96 % yield. ^1H NMR (400 MHz, $CDCl_3$) δ 8.16 (br, 1H), 7.92-7.80 (m, 1H), 7.61-7.52 (m, 1H), 7.51-7.42 (m, 2H), 4.46 (s, 2H); ^{13}C NMR (101 MHz, $CDCl_3$) δ 172.45, 143.84, 132.33, 131.79, 128.06, 123.75, 123.28, 45.94.

8. Preparation of other directing groups protected aliphatic amine

8.1. Preparation of benzyloxycarbonyl amide

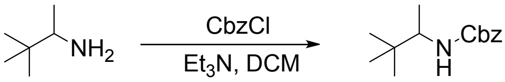

To a solution of 3,3-dimethylbutan-2-amine (20 mmol) in dichloromethane (40 mL) was added triethylamine (2.8 mL, 20 mmol, 1.0 equiv) at 0 °C. After stirring for 5 min, benzyl carbonochloridate (8.8 mL, 22 mmol, 1.1 equiv) was added dropwise and then the mixture was stirred for 6 h at rt overnight. Water was added and the mixture was extracted with DCM. The combined organic layers was washed with water and brine, dried over anhydrous Na_2SO_4, and concentrated in vacuo. The resulting residue was purified by column chromatography on silica gel to give the desired picolinamide product in 93% yield.

8.2. Preparation of trifluoromethanesulfonamide[3]

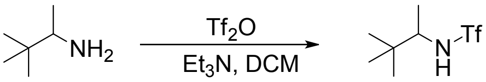

To a solution of 3,3-dimethylbutan-2-amine (50 mmol) in dichloromethane (100 mL) was added triethylamine (7.0 mL, 50 mmol, 1.0 equiv) at –78 °C under nitrogen. After stirring for 5 min at –78 °C, trifluoromethanesulfonic anhydride (8.8 mL, 52.5 mmol, 1.05 equiv) was added dropwise and then the mixture was stirred for 1 h at that temperature before being quenched by ice water (100 mL). The organic layer was separated and the aqueous layer was extracted with dichloromethane (50 mL × 2). The combined organic phase was washed with brine (30 mL), and then dried over anhydrous Na_2SO_4. Evaporation and column chromatography on silica gel afforded the product as colorless oil in 92% yield.

8.3. Preparation of picolinamide[4]

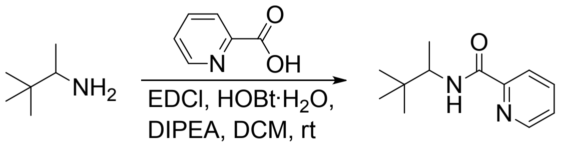

A mixture of 3,3-dimethylbutan-2-amine (1.0 eq), picolinic acid (1.1 eq), EDCI (1.1 eq), HOBt·H_2O (1.1 eq), and DIPEA (3.0 eq) in anhydrous DCM (0.2 M) was stirred at rt overnight. Water was added and the mixture was extracted with DCM. The combined organic layers was washed with water and brine, dried over anhydrous Na_2SO_4, and concentrated in vacuo. The resulting residue was purified by column chromatography on silica gel to give the desired picolinamide product in 91% yield.

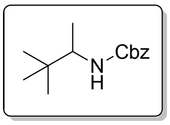

^{1}H NMR (400 MHz, $CDCl_3$) δ 7.42-7.27 (m, 5H), 5.19-5.04 (m, 2H), 4.64 (br, 1H), 3.59 (m, 1H), 1.08 (d, J = 6.8 Hz, 3H), 0.90 (s, 9H); ^{13}C NMR (101 MHz, $CDCl_3$) δ 156.21, 136.81, 128.62, 128.21, 128.18, 66.67, 55.20, 34.45, 26.21, 16.50.

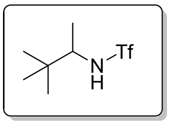

^{1}H NMR (400 MHz, $CDCl_3$) δ 4.70 (br, 1H), 3.40 (m, 1H), 1.24 (d, J = 6.8 Hz, 3H), 0.96 (s, 9H); ^{13}C NMR (101 MHz, $CDCl_3$) δ 124.55, 121.36, 118.17, 114.98, 61.00, 34.63, 26.16, 17.33.

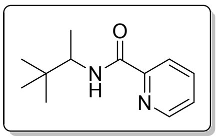

^{1}H NMR (400 MHz, $CDCl_3$) δ 8.62-8.44 (m, 1H), 8.20 (d, J = 7.8 Hz, 1H), 8.03 (br, 1H), 7.83 (m, 1H), 7.40 (m, 1H), 4.05 (m, 1H), 1.18 (d, J = 6.8 Hz, 3H), 0.97 (s, 9H); ^{13}C NMR (101 MHz, $CDCl_3$) δ 163.60, 150.31, 148.10, 137.42, 126.06, 122.39, 53.03, 34.62, 26.39, 16.25.

9. Preparation of oxalamide substrates

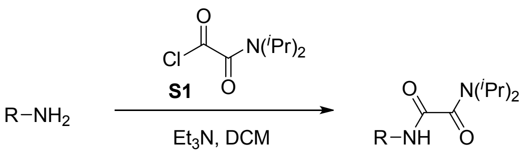

9.1. Preparation of N, N–Diisopropyloxamoyl chloride S1[5]

A solution of Diisopropylamine (7.01 mL, 50 mmol, 1.0 equiv) in CH_2Cl_2 (50 mL) was added dropwise to a solution of oxalyl chloride (6.44 ml, 75 mmol, 1.5 equiv) in CH_2Cl_2 (100 mL) at 0 °C, after stirring for 5 min, triethylamine (7.30 mL, 52.5 mmol, 1.05 equiv) was added dropwise. The solution was warmed to room temperature and stirred for 6 hours. The excess of oxalyl

chloride and the solvent were removed under reduce pressure and CH_2Cl_2 (30 mL) was added and evaporated. This operation was performed twice to give **1a** as a pale yellow solid. The crude product was used in the next step without any purification.

9.2. General procedures for the preparation of oxalamide substrates 1a, 1d, 1g, 1j–m, 1p, 3a–3h, 5, 7[6]

A solution of amine (20 mmol, 1.0 eq) in CH_2Cl_2 (40 mL) was added dropwise to a solution of N,N–Diisopropyloxamoyl chloride **S1** (25 mmol, 1.25 equiv) in CH_2Cl_2 (50 mL) at 0 °C, after stirring for 5 min, triethylamine (2.92 ml, 21 mmol, 1.05 equiv) was added dropwise and then the mixture was stirred for 6 hours at room temperature before quenched by water (50 mL). The organic layer was separated and the aqueous layer was extracted with CH_2Cl_2 (20 mL × 2). The combined organic phase was washed with brine (30 mL), and then dried over anhydrous Na_2SO_4. Evaporation and column chromatography on silica gel afforded corresponding amide substrates as white solid or colourless liquid in >90% yield.

9.3. Preparation of 1b, 1h, 1i, 1n, 1o,1s, 1t, 1u

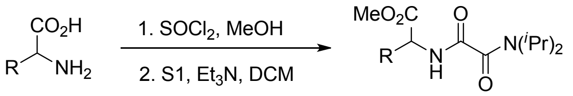

To a solution of amino acid (20 mmol, 1.0 eq) in MeOH (30 mL), at 0 °C, was added $SOCl_2$ (4.35 mL, 60 mmol, 3.0 eq) dropwise. The resulting mixture was allowed to stir from 0 °C to room temperature overnight. The solvent was removed under reduced pressure to afford a white solid, which was used directly for next step. The second step followed the general oxalamide coupling procedure, to give the product in about 75% yield.

9.4. Preparation of 1c[7]

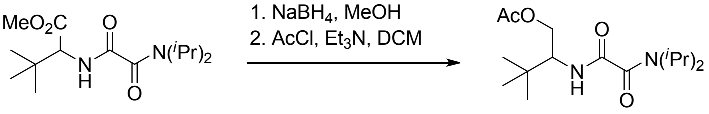

1b **1c**

To a solution of **1a** (3.00 g, 10 mmol, 1.0 eq) in MeOH (10 ml) at room temperature, was added $NaBH_4$ (0.76 g, 20 mmol, 2.0 eq) in portions. Water was added after the reaction was determined by TLC, and the mixture was extracted with DCM. The combined organic layers was washed with water and brine, dried over anhydrous Na_2SO_4, the resulting solution was used directly for next step. The solution was treated with AcCl (0.78 mL, 11 mmol, 1.1 eq) and Et_3N (2.78 mL, 20 mmol, 2.0 eq) at room temperature overnight. Water was added and the mixture was extracted with DCM. The combined organic layers was washed with water and brine, dried over anhydrous Na_2SO_4, and concentrated in vacuo. The resulting residue was purified by column chromatography on silica gel to give the product **1b** 2.74 g, 87%.

9.5. Preparation of 1e[2]

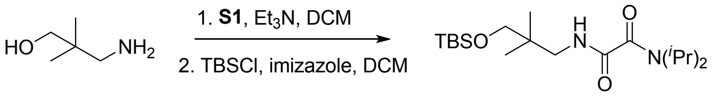

1e

The first step using 3-amino-2,2-dimethylpropan-1-ol (2.06 g, 20 mmol, 1.0 eq) as starting material followed the general oxalamide coupling procedure, affording a white solid. The solid was dissolved in DCM (30 mL), and then added imidazole (2.72 g, 40 mmol, 2.0 eq) at rt. When the solution turned clear again, it was cooled to 0 °C and TBSCl (4.52 g, 30 mmol, 1.5 eq) was added in small portions. Then the mixture was stirred for 6 hours at room temperature before quenched by water (20 mL). The organic layer was separated and the aqueous layer was extracted with CH_2Cl_2 (20 mL × 2). The combined organic phase was washed with brine (15 mL), and then dried over anhydrous Na_2SO_4. Evaporation and column chromatography on silica gel to give the product **1e** 5.81 g, 78%.

9.6. Preparation of 1f

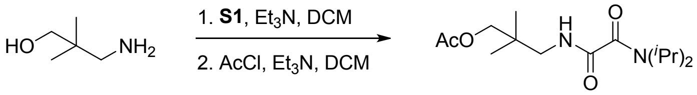

1f

The first step using 3-amino-2,2-dimethylpropan-1-ol (2.06 g, 20 mmol, 1.0 eq) as starting material followed the general oxalamide coupling procedure, affording a white solid. The solid was dissolved in DCM (30 mL), and the solution was treated with AcCl (1.56 mL, 22 mmol, 1.1 eq) and Et_3N (2.78 mL, 20 mmol, 1.0 eq) at room temperature overnight. Water was added and the mixture was extracted with DCM. The organic layer was separated and the aqueous layer was extracted with CH_2Cl_2 (20 mL × 2). The combined organic phase was washed with brine (15 mL), and then dried over anhydrous Na_2SO_4. Evaporation and column chromatography on silica gel to give the product **1f** 4.92 g, 81%.

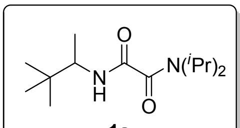

1a

^{1}H NMR (400 MHz, $CDCl_3$) δ 6.79 (br, 1H), 4.72 (m, 1H), 3.83 (m, 1H), 3.50 (m, 1H), 1.42 (m, 6H), 1.29-1.17 (m, 6H), 1.09 (d, J = 6.8 Hz, 3H), 0.92 (d, J = 4.8 Hz, 9H); ^{13}C NMR (101 MHz, $CDCl_3$) δ 163.61, 162.74, 53.03, 49.76, 46.61, 34.45, 26.26, 21.05, 20.93, 20.32, 20.23, 15.92; HRMS Calcd for $C_{14}H_{28}N_2NaO_2$ [M+Na$^+$]: 279.2048; Found: 279.2043.

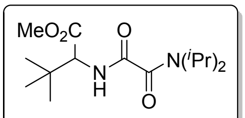

1b

^{1}H NMR (400 MHz, $CDCl_3$) δ 7.30 (br, 1H), 4.67 (m, 1H), 4.40 (d, J = 9.6 Hz, 1H), 3.74 (s, 3H), 3.51 (m, 1H), 1.43 (dd, J = 6.8, 2.1 Hz, 6H), 1.22 (dd, J = 6.6, 4.3 Hz, 6H), 1.01 (s, 9H); ^{13}C NMR (101 MHz, $CDCl_3$) δ 171.24, 162.88, 162.56, 60.24, 52.03, 49.74, 46.75, 35.13, 26.66, 20.99, 20.95, 20.22, 20.19; HRMS Calcd for $C_{15}H_{28}N_2NaO_4$ [M+Na$^+$]: 323.1947; Found: 323.1951.

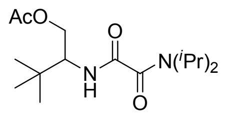

1c

^{1}H NMR (400 MHz, $CDCl_3$) δ 6.86 (br, 1H), 4.64 (m, 1H), 4.27 (m, 1H), 4.15-3.96 (m, 2H), 3.52 (m, 1H), 2.03 (s, 3H), 1.43 (d, J = 6.8 Hz, 6H), 1.23 (t, J = 7.0 Hz, 6H), 0.99 (s, 9H); ^{13}C NMR (101 MHz, $CDCl_3$) δ 171.13, 163.62, 163.43, 63.74, 55.89, 49.86, 46.65, 34.12, 26.82, 21.02, 21.00, 20.34, 20.22; HRMS Calcd for $C_{16}H_{30}N_2NaO_4$ [M+Na$^+$]: 337.2103; Found: 337.2101.

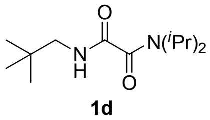

1d

^{1}H NMR (400 MHz, $CDCl_3$) δ 7.05 (br, 1H), 4.83-4.54 (m, 1H), 3.49 (m, 1H), 3.07 (d, J = 6.6 Hz, 2H), 1.41 (d, J = 6.8 Hz, 6H), 1.21 (d, J = 6.7 Hz, 6H), 0.92 (t, J = 3.0 Hz, 9H); ^{13}C NMR (101 MHz, $CDCl_3$) δ 163.61, 163.58, 50.47, 49.79, 46.56, 32.17, 27.30, 20.94, 20.22; HRMS Calcd for $C_{13}H_{26}N_2NaO_2$ [M+Na$^+$]: 265.1892; Found: 265.1890.

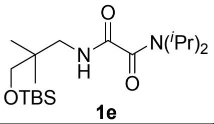

1e

^{1}H NMR (400 MHz, $CDCl_3$) δ 7.21 (br, 1H), 4.49 (m, 1H), 3.47 (m, 1H), 3.37 (s, 2H), 3.20 (d, J = 5.9 Hz, 2H), 1.41 (d, J = 6.8 Hz, 6H), 1.20 (d, J = 6.7 Hz, 6H), 0.88 (d, J = 1.4 Hz, 15H), 0.04 (s, 6H); ^{13}C NMR (101 MHz, $CDCl_3$) δ 164.02, 164.01, 72.17, 49.84, 48.15, 46.34, 35.95, 26.04, 22.53, 20.99, 20.24, 18.33, -5.51; HRMS Calcd for $C_{19}H_{41}N_2O_3Si$ [M+H$^+$]: 373.2886; Found: 373.2888.

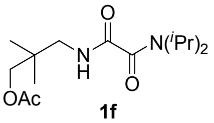

1f

^{1}H NMR (400 MHz, $CDCl_3$) δ 7.26 (br, 1H), 4.63 (m, 1H), 3.83 (s, 2H), 3.48 (m, 1H), 3.17 (d, J = 6.7 Hz, 2H), 2.06 (s, 3H), 1.39 (d, J = 6.8 Hz, 6H), 1.20 (d, J = 6.7 Hz, 6H), 0.93 (s, 6H); ^{13}C NMR (101 MHz, $CDCl_3$) δ 171.26, 163.69, 163.40, 70.44, 49.79, 46.56, 46.08, 35.60, 22.58, 20.94, 20.92, 20.17; HRMS Calcd for $C_{15}H_{29}N_2O_4$ [M+H$^+$]: 301.2127; Found: 301.2131.

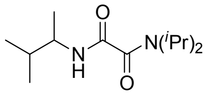

1g

^{1}H NMR (400 MHz, $CDCl_3$) δ 6.81 (br, 1H), 4.72 (m, 1H), 3.81 (m, 1H), 3.49 (m, 1H), 1.73 (d, J = 7.8 Hz, 1H), 1.41 (m, 6H), 1.21 (m, 6H), 1.11 (d, J = 6.8 Hz, 3H), 0.91 (m, 6H); ^{13}C NMR (101

MHz, $CDCl_3$) δ 163.72, 162.83, 50.23, 49.75, 46.45, 32.96, 20.92, 20.84, 20.20, 20.13, 18.62, 18.57, 17.28; HRMS Calcd for $C_{13}H_{27}N_2O_2$ [M+H$^+$]: 243.2073; Found: 243.2072.

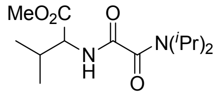

1h

^{1}H NMR (400 MHz, $CDCl_3$) δ 7.23 (d, J = 8.4 Hz, 1H), 4.68–4.61 (m, 1H), 4.49–4.46 (dd, J = 8.9, 5.0 Hz, 1H), 3.74 (s, 3H), 3.54–3.47 (m, 1H), 2.25–2.17 (m, 1H), 1.42 (d, J = 6.8 Hz, 6H), 1.21 (dd, J = 6.6, 3.9 Hz, 6H), 0.95 (dd, J = 9.7, 6.9 Hz, 6H); ^{13}C NMR (101 MHz, $CDCl_3$) δ 171.55, 163.19, 162.64, 57.24, 52.19, 49.68, 46.53, 31.22, 20.83, 20.14, 20.02, 18.95, 17.85; HRMS Calcd for $C_{14}H_{27}N_2O_4$ [M+H$^+$]: 286.1893; Found: 286.1898.

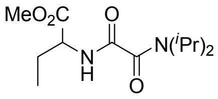

1i

^{1}H NMR (400 MHz, $CDCl_3$) δ7.29 (d, J = 7.2 Hz, 1H), 4.65–4.68 (m, 1H), 4.51–4.46 (m, 1H), 3.73 (s, 3H), 3.52-3.45 (m, 1H), 1.94–1.88 (m, 1H), 1.80–1.73 (m, 1H), 1.44–1.39 (m, 6H), 1.21–1.19 (m, 6H), 0.93 (t, J = 7.5 Hz, 3H); ^{13}C NMR (101 MHz, $CDCl_3$) δ 172.07, 163.09, 162.62, 53.45, 52.48, 49.75, 46.66, 25.49, 20.95, 20.92, 20.21, 20.11, 9.74; HRMS Calcd for $C_{13}H_{25}N_2O_4$ [M+H$^+$]: 273.1814; Found: 273.1818.

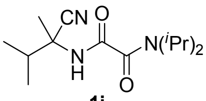

1j

^{1}H NMR (400 MHz, $CDCl_3$) δ 7.63 (br, 1H), 4.80 (m, 1H), 3.53 (m, 1H), 2.36 (m, 1H), 1.63 (s, 3H), 1.40 (d, J = 6.8 Hz, 6H), 1.23 (d, J = 6.7 Hz, 6H), 1.15 (d, J = 6.8 Hz, 3H), 1.06 (d, J = 6.8 Hz, 3H); ^{13}C NMR (101 MHz, $CDCl_3$) δ 162.14, 161.96, 119.19, 54.94, 49.95, 46.92, 34.93, 21.11, 20.96, 20.90, 20.13, 20.07, 17.79, 16.56; HRMS Calcd for $C_{14}H_{25}N_3NaO_2$ [M+Na$^+$]: 290.1844; Found: 290.1839.

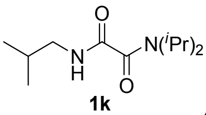

1k

^{1}H NMR (400 MHz, $CDCl_3$) δ 7.08 (br, 1H), 4.76-4.65 (m, 1H), 3.49 (m, 1H), 3.09 (t, J = 6.5 Hz, 2H), 1.81 (m, 1H), 1.40 (d, J = 6.8 Hz, 6H), 1.21 (d, J = 6.7 Hz, 6H), 0.92 (d, J = 6.7 Hz, 6H); ^{13}C NMR (101 MHz, $CDCl_3$) δ 163.47, 49.76, 46.71, 46.59, 28.49, 20.95, 20.20; HRMS Calcd for $C_{12}H_{24}N_2NaO_2$ [M+Na$^+$]: 251.1735; Found: 251.1732.

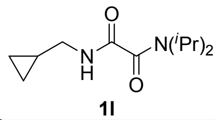

1l

^{1}H NMR (400 MHz, $CDCl_3$) δ 7.26 (br, 1H), 4.64 (m, 1H), 3.47 (m, 1H), 3.10 (dd, *J* = 7.0, 5.8 Hz, 2H), 1.38 (d, *J* = 6.8 Hz, 6H), 1.19 (d, *J* = 6.7 Hz, 6H), 0.95 (m, 1H), 0.53-0.43 (m, 2H), 0.25-0.13 (m, 2H); ^{13}C NMR (101 MHz, $CDCl_3$) δ 163.53, 163.25, 49.74, 46.50, 44.18, 20.90, 20.13, 10.43, 3.55; HRMS Calcd for $C_{12}H_{23}N_2O_2$ [M+H$^+$]: 227.1760; Found: 227.1750.

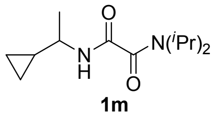

1m

^{1}H NMR (400 MHz, $CDCl_3$) δ 6.91 (br, 1H), 4.76 (m, 1H), 3.51 (m, 1H), 3.40-3.30 (m, 1H), 1.42 (dd, *J* = 6.8, 1.9 Hz, 6H), 1.25-1.20 (m, 9H), 0.86 (m, 1H), 0.59-0.41 (m, 2H), 0.35 (m, 1H), 0.22 (m, 1H); ^{13}C NMR (101 MHz, $CDCl_3$) δ 163.37, 162.55, 49.92, 49.70, 46.66, 21.00, 20.98, 20.25, 20.22, 20.04, 17.23, 3.39, 3.16; HRMS Calcd for $C_{13}H_{24}N_2NaO_2$ [M+Na$^+$]: 263.1735; Found: 263.1730.

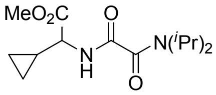

1n

^{1}H NMR (400 MHz, $CDCl_3$) δ 7.49 (br, 1H), 4.54 (m, 1H), 3.89-3.79 (m, 1H), 3.70 (s, 3H), 3.45 (m, 1H), 1.37 (t, *J* = 6.3 Hz, 6H), 1.16 (d, *J* = 6.5 Hz, 6H), 1.12-1.03 (m, 1H), 0.60-0.42 (m, 3H), 0.38 (m, 1H); ^{13}C NMR (101 MHz, $CDCl_3$) δ 171.50, 163.02, 162.68, 56.10, 52.29, 49.62, 46.40, 20.77, 20.71, 20.05, 19.94, 13.34, 3.41, 3.26; HRMS Calcd for $C_{14}H_{24}N_2NaO_4$ [M+Na$^+$]: 307.1634; Found: 307.1618.

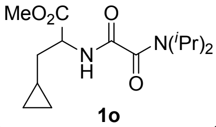

1o

^{1}H NMR (400 MHz, $CDCl_3$) δ 7.43 (br, 1H), 4.71-4.39 (m, 2H), 3.70 (d, *J* = 1.1 Hz, 3H), 3.46 (m), 1.69 (m, 2H), 1.38 (m, 6H), 1.17 (dd, *J* = 6.5, 3.2 Hz, 6H), 0.76-0.59 (m, 1H), 0.52-0.31 (m, 2H), 0.04 (m, 2H); ^{13}C NMR (101 MHz, $CDCl_3$) δ 172.04, 162.92, 162.68, 52.68, 52.33, 49.68, 46.50, 36.80, 20.85, 20.80, 20.13, 19.97, 7.01, 4.24, 4.20; HRMS Calcd for $C_{15}H_{26}N_2NaO_4$ [M+Na]: 321.1790; Found: 321.1775.

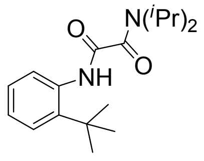

1p

^{1}H NMR (400 MHz, $CDCl_3$) δ 9.13 (br, 1H), 7.77 (dd, *J* = 7.9, 1.3 Hz, 1H), 7.41 (dd, *J* = 7.9, 1.4 Hz, 1H), 7.25–7.23 (m, 2H), 5.19–5.12 (m, 1H), 3.62–3.57 (m, 1H), 1.48 (d, *J* = 6.8 Hz, 6H), 1.46 (s, 9H), 1.28 (d, *J* = 6.7 Hz, 6H); ^{13}C NMR (101 MHz, $CDCl_3$) δ 162.44, 160.23, 142.18, 134.56, 126.85, 126.72, 126.14, 125.83, 49.69, 47.21, 34.67, 30.71, 21.10, 20.19. This compound was known.[5]

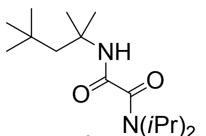

1q

^{1}H NMR (400 MHz, $CDCl_3$) δ 6.75 (br, 1H), 4.76 (m, 1H), 3.47 (m, 1H), 1.75 (s, 2H), 1.41 (s, 6H), 1.39 (d, *J* = 6.8 Hz, 6H), 1.20 (d, *J* = 6.7 Hz, 6H), 0.99 (s, 9H); ^{13}C NMR (101 MHz, $CDCl_3$) δ 163.79, 163.41, 49.72, 46.32, 37.91, 37.55, 25.76, 22.38, 20.80, 20.06. This compound was known.[5]

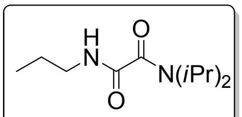

1r

^{1}H NMR (400 MHz, $CDCl_3$) δ 7.28 (br, 1H), 4.62–4.58 (m, 1H), 3.49–3.42 (m, 1H), 3.19 (dd, *J* = 13.6, 6.7 Hz, 2H), 1.57–1.47 (m, 2H), 1.36 (d, *J* = 6.8 Hz, 6H), 1.17 (d, *J* = 6.7 Hz, 6H), 0.89 (t, *J* = 7.4 Hz, 3H); ^{13}C NMR (101 MHz, $CDCl_3$) δ 163.70, 163.47, 49.75, 46.42, 41.02, 22.51, 20.86, 20.11, 11.42; HRMS Calcd for $C_{11}H_{21}N_2O_2$ [M-H$^+$]: 213.1603; Found: 213.1608.

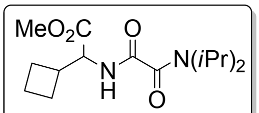

1s

White solid (4.41 g, 74% yield). ^{1}H NMR (400 MHz, $CDCl_3$) δ 7.25 (br, 1H), 4.63–4.57 (m, 1H), 4.47 (t, *J* = 8.2 Hz, 1H), 3.70 (s, 3H), 3.52–3.45 (m, 1H), 2.71–2.62 (m, 1H), 2.01–1.76 (m, 6H), 1.40 (d, *J* = 6.8 Hz, 6H), 1.19 (dd, *J* = 6.6, 2.2 Hz, 6H); ^{13}C NMR (101 MHz, $CDCl_3$) δ 171.37, 163.28, 162.67, 55.89, 52.29, 49.75, 46.60, 37.66, 25.07, 24.82, 20.89, 20.18, 20.11, 17.96; HRMS Calcd for $C_{15}H_{26}N_2NaO_4$ [M+Na$^+$]: 321.1790; Found: 321.1790.

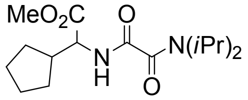

1t

White solid (4.37 g, 70% yield). ^{1}H NMR (400 MHz, $CDCl_3$) δ 7.20 (d, *J* = 8.0 Hz, 1H), 4.70–4.64 (m, 1H), 4.51–4.47 (m, 1H), 3.74 (d, *J* = 1.8 Hz, 3H), 3.54–3.48 (m, 1H), 2.34–2.24 (m, 1H), 1.76–1.53 (m, 6H), 1.42 (d, *J* = 6.8 Hz, 6H), 1.40–1.33 (m, 2H), 1.22 (dd, *J* = 6.6, 3.4 Hz, 6H); ^{13}C NMR (101 MHz, $CDCl_3$) δ 171.11, 162.05, 161.53, 54.40, 51.38, 48.76, 45.75, 41.71, 28.13, 27.62, 24.32, 24.07, 19.99, 19.25, 19.18; HRMS Calcd for $C_{16}H_{28}N_2NaO_4$ [M+Na$^+$]: 335.1947;

Found: 335.1951.

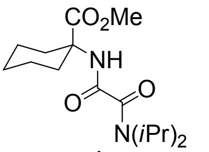

1u

White solid (4.99 g, 80% yield). ^{1}H NMR (400 MHz, CDCl$_3$) δ 7.06 (br, 1H), 4.72–4.65 (m, 1H), 3.71 (s, 3H), 3.54–3.47 (m, 1H), 2.07 (d, J = 13.5 Hz, 2H), 1.89–1.82 (m, 2H), 1.68–1.60 (m, 3H), 1.53–1.46 (m, 2H), 1.42 (d, J = 6.8 Hz, 6H), 1.33–1.27 (m, 1H), 1.21 (d, J = 6.7 Hz, 6H); ^{13}C NMR (101 MHz, CDCl$_3$) δ 174.09, 162.86, 162.68, 59.05, 52.48, 49.62, 46.67, 32.24, 25.17, 21.44, 20.97, 20.22; HRMS Calcd for $C_{16}H_{28}N_2NaO_4$ [M+Na$^+$]: 335.1947; Found: 31951.

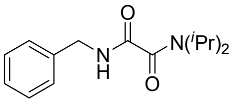

3a

^{1}H NMR (400 MHz, CDCl$_3$) δ 7.39-7.27 (m, 5H), 7.20 (br, 1H), 4.87–4.80 (m, 1H), 4.46 (d, J = 6.0 Hz, 2H), 3.56–3.49 (m, 1H), 1.42 (d, J = 6.8 Hz, 6H), 1.24 (d, J = 6.7 Hz, 6H); ^{13}C NMR (101 MHz, CDCl$_3$) δ 163.16, 163.04, 137.53, 128.84, 127.90, 127.70, 49.75, 46.69, 43.41, 20.97, 20.15. HRMS Calcd for $C_{15}H_{22}N_2O_2$ [M+Na$^+$]: 285.1579; Found: 285.1579.

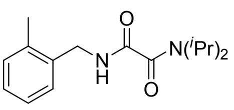

3b

^{1}H NMR (400 MHz, CDCl$_3$) δ 7.29–7.26 (m, 1H), 7.23–7.17 (m, 3H), 7.13 (br, 1H), 4.83– 4.76 (m, 1H), 4.47 (d, J = 5.7 Hz, 2H), 3.57–3.50 (m, 1H), 2.36 (s, 3H), 1.43 (d, J = 6.8 Hz, 6H), 1.26 (d, J = 6.7 Hz, 6H); ^{13}C NMR (101 MHz, CDCl$_3$) δ 163.02, 162.98, 136.51, 135.18, 130.65, 128.62, 128.01, 126.38, 49.76, 46.70, 41.59, 20.99, 20.17, 19.12. HRMS Calcd for $C_{16}H_{24}N_2O_2$ [M+Na$^+$]: 299.1735; Found: 299.1729.

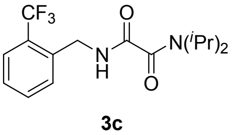

3c

^{1}H NMR (400 MHz, CDCl$_3$) δ 7.66 (d, J = 7.9 Hz, 1H), 7.57-7.52 (m, 2H), 7.40 (m, 1H), 7.20 (br, 1H), 4.82-4.75 (m, 1H), 4.66 (d, J = 6.3 Hz, 2H), 3.56–3.49 (m, 1H), 1.42 (d, J = 6.8 Hz, 6H), 1.24 (d, J = 6.7 Hz, 6H); ^{13}C NMR (101 MHz, CDCl$_3$) δ 163.16 , 162.87, 161.10 (d, J_{C-F} = 245.0 Hz) , 130.19 (d, J_{C-F} = 5.0 Hz), 129.57 (d, J_{C-F} = 8.1 Hz), 124.56 (d, J_{C-F} = 15.0, Hz), 124.44 (d, J_{C-F} = 4.0 Hz). 115.55 (d, J_{C-F} = 21.0 Hz), 49.73, 46.71, 37.38 (d, J_{C-F} = 4.0 Hz), 20.96, 20.15; HRMS Calcd for $C_{16}H_{21}F_3N_2O_2$ [M+Na$^+$]: 353.1453; Found: 353.1459.

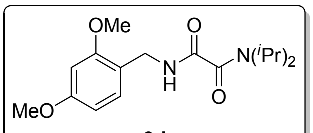

3d

^{1}H NMR (400 MHz, $CDCl_3$) δ 7.18 (m, 2H), 6.43 (m, 2H), 4.80 – 4.73 (m, 1H), 4.38 (d, J = 6.0 Hz, 2H), 3.82 (s, 3H), 3.79 (s, 3H), 3.52 – 3.45 (m, 1H), 1.40 (d, J = 6.8 Hz, 6H), 1.21 (d, J = 6.7 Hz, 6H); ^{13}C NMR (101 MHz, $CDCl_3$) δ 163.20, 162.85, 160.73, 158.70, 130.50, 118.09, 103.98, 98.59, 55.44, 55.42, 49.59, 46.49, 38.76, 20.90, 20.12; HRMS Calcd for $C_{17}H_{26}N_2O_4$ [M+Na^+]: 345.1790; Found: 345.1783.

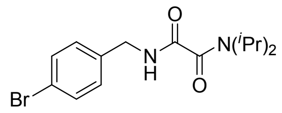

3e

^{1}H NMR (400 MHz, $CDCl_3$) δ 7.45–7.43 (m, 3H), 7.17 (d, J = 8.3 Hz, 2H), 4.78–4.71 (m, 1H), 4.38 (d, J = 6.1 Hz, 2H), 3.55–3.48 (m, 1H), 1.40 (d, J = 6.8 Hz, 6H), 1.23 (d, J = 6.7 Hz, 6H); ^{13}C NMR (101 MHz, $CDCl_3$) δ 163.23, 162.98, 136.71, 131.90, 129.58, 121.57, 49.81, 46.73, 42.72, 20.96, 20.14; HRMS Calcd for $C_{15}H_{21}BrN_2O_2$ [M+Na^+]: 363.0684; Found: 363.0685.

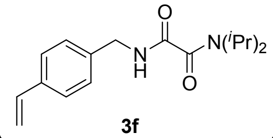

3f

^{1}H NMR (400 MHz, $CDCl_3$) δ 7.43–7.33 (m, 3H), 7.28–7.22 (m, 2H), 6.69 (dd, J = 17.6, 10.9 Hz, 1H), 5.73 (d, J = 17.6, 1H), 5.24 (d, J = 10.9, 1H), 4.80–4.73 (m,1H), 4.43 (d, J = 6.0 Hz, 2H), 3.55–3.48 (m, 1H), 1.41 (d, J = 6.8 Hz, 6H), 1.23 (d, J = 6.7 Hz, 6H); ^{13}C NMR (101 MHz, $CDCl_3$) δ 163.23, 137.17, 136.96, 136.44, 128.06, 126.57, 113.99, 49.77, 46.57, 43.01, 20.90, 20.10. HRMS Calcd for $C_{17}H_{24}N_2O_2$ [M+Na^+]; 311.1735; Found:311.1731.

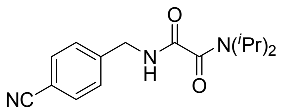

3g

^{1}H NMR (400 MHz, $CDCl_3$) δ 7.78 (br, 1H), 7.60 (d, J = 8.3 Hz, 2H), 7.40 (d, J = 8.3 Hz, 2H), 4.67 (m, 1H), 4.47 (d, J = 6.3 Hz, 2H), 3.51 (m, 1H), 1.39 (d, J = 6.8 Hz, 6H), 1.22 (d, J = 6.7 Hz, 6H); ^{13}C NMR (101 MHz, $CDCl_3$) δ 163.38, 162.72, 143.17, 132.62, 128.31, 118.76, 111.54, 49.87, 46.82, 42.87, 20.96, 20.13; HRMS Calcd for $C_{16}H_{21}N_3NaO_2$ [M+Na^+]: 310.1531; Found: 310.1505.

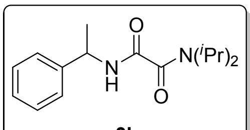

3h

^{1}H NMR (400 MHz, $CDCl_3$) δ 7.73 (br, 1H), 7.37–7.30 (m, 4H), 7.28–7.21 (m, 1H), 5.09–5.02 (m, 1H), 4.65 (s, 1H), 3.55–3.43 (m, 1H), 1.50 (d, *J* = 7.0 Hz, 3H), 1.41 (dd, *J* = 9.7, 6.8 Hz, 6H), 1.20 (dd, *J* = 9.9, 6.7 Hz, 6H); ^{13}C NMR (101 MHz, $CDCl_3$) δ 163.36, 162.42, 143.05, 128.70, 127.38, 126.18, 49.70, 49.11, 46.53, 22.03, 20.85, 20.12. HRMS Calcd for $C_{16}H_{24}N_2O_2$ [M+Na$^+$]: 299.1735; Found: 299.1734.

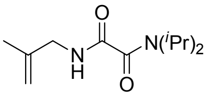

5

^{1}H NMR (400 MHz, $CDCl_3$) δ 7.06 (br, 1H), 4.93-4.81 (m, 2H), 4.73 (m, 1H), 3.82 (d, *J* = 6.3 Hz, 2H), 3.51 (m, 1H), 1.75 (d, *J* = 0.5 Hz, 3H), 1.42 (d, *J* = 6.8 Hz, 6H), 1.22 (d, *J* = 6.7 Hz, 6H); ^{13}C NMR (101 MHz, $CDCl_3$) δ 163.42, 141.18, 111.25, 49.75, 46.42, 44.68, 20.82, 20.35, 20.07; HRMS Calcd for $C_{12}H_{22}N_2NaO_2$ [M+Na$^+$]: 249.1579; Found: 249.1590.

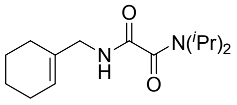

7

^{1}H NMR (400 MHz, $CDCl_3$) δ 6.81 (br, 1H), 5.48 (s, 1H), 4.70 (m, 1H), 3.50 (m, 1H), 3.35 (m, 2H), 2.16 (t, *J* = 6.8 Hz, 2H), 1.97 (m, 4H), 1.65-1.58 (m, 2H), 1.57-1.50 (m, 2H), 1.41 (d, *J* = 6.8 Hz, 6H), 1.22 (d, *J* = 6.7 Hz, 6H); ^{13}C NMR (101 MHz, $CDCl_3$) δ 163.32, 163.27, 134.38, 123.73, 49.77, 46.60, 37.52, 37.36, 28.10, 25.36, 22.94, 22.44, 21.01, 20.22; HRMS Calcd for $C_{15}H_{26}N_2NaO_2$ [M+Na$^+$]: 289.1892; Found: 289.1901.

10. Palladium-catalyzed carbonylation of γ-C(sp^3)–H bonds

A mixture of oxalamide (0.2 mmol, 1.0 eq), $Pd(OAc)_2$ (4.5 mg, 0.1 eq), AgOAc (83.4 mg, 2.5 eq), *m*-CF_3PhCO_2H (11.4 mg, 0.3 eq) and mesitylene (0.3 mL) in a 20 mL glass vial was purged with CO (3-times,), and sealed with a teflon septa. The vial was heated at 140 ^{o}C in an oil bath for 24 hours, then the reaction mixture was cooled to rt, and concentrated in vacuo. The resulting residue was purified by column chromatography on silica gel to give the product **2**.

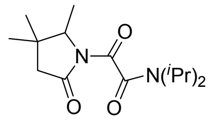

2a

^{1}H NMR (400 MHz, $CDCl_3$) δ 3.96 (d, *J* = 5.5 Hz, 1H), 3.50 (m, 2H), 2.51 (s, 1H), 2.20 (d, *J* = 16.2 Hz, 1H), 1.48 (d, *J* = 6.2 Hz, 6H), 1.25 (d, *J* = 6.6 Hz, 3H), 1.20 (d, *J* = 6.6 Hz, 6H), 1.13 (d, *J* = 9.5 Hz, 6H); ^{13}C NMR (101 MHz, $CDCl_3$) δ 174.12, 164.25, 163.12, 61.05, 50.94, 45.80, 44.84, 36.06, 28.77, 22.65, 20.21, 14.53; HRMS Calcd for $C_{15}H_{26}N_2NaO_3$ [M+Na$^+$]: 305.1841; Found: 305.1833.

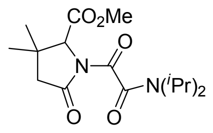

2b

^{1}H NMR (400 MHz, $CDCl_3$) δ 4.31 (s, 1H), 3.78 (s, 3H), 3.71-3.55 (m, 1H), 3.48 (m, 1H), 2.59 (d, *J* = 17.3 Hz, 1H), 2.31 (d, *J* = 17.4 Hz, 1H), 1.49 (m, 6H), 1.29 (s, 3H), 1.20 (d, *J* = 6.6 Hz, 3H), 1.18-1.10 (m, 6H); ^{13}C NMR (101 MHz, $CDCl_3$) δ 173.36, 170.37, 164.11, 162.33, 66.51, 52.54, 50.65, 45.93, 45.35, 36.16, 29.52, 23.91, 20.73, 20.30, 20.01, 19.47; HRMS Calcd for $C_{16}H_{26}N_2NaO_5$ [M+Na$^+$]: 349.1739; Found: 349.1753.

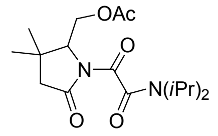

2c

^{1}H NMR (400 MHz, $CDCl_3$) δ 4.67 (d, *J* = 149.4 Hz, 1H), 4.22-3.88 (m, 2H), 3.51 (m, 2H), 2.63 (d, *J* = 16.3 Hz, 1H), 2.25 (d, *J* = 17.6 Hz, 1H), 2.03 (s, 3H), 1.49 (t, *J* = 7.5 Hz, 6H), 1.20 (m, 12H); ^{13}C NMR (101 MHz, $CDCl_3$) δ 174.44, 170.10, 164.26, 162.71, 61.70, 50.95, 46.09, 45.89, 36.10, 30.02, 22.78, 20.86, 20.59, 20.29, 19.51; HRMS Calcd for $C_{17}H_{28}N_2NaO_5$ [M+Na$^+$]: 363.1896; Found: 363.1912.

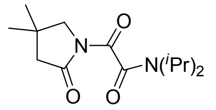

2d

^{1}H NMR (400 MHz, $CDCl_3$) δ 3.59-3.40 (m, 4H), 2.38 (s, 2H), 1.46 (d, *J* = 6.7 Hz, 6H), 1.25-1.12 (m, 12H); ^{13}C NMR (101 MHz, $CDCl_3$) δ 174.39, 164.28, 162.98, 56.17, 50.96, 47.09, 45.85, 33.19, 27.17, 20.38, 19.84; HRMS Calcd for $C_{14}H_{24}N_2NaO_3$ [M+Na$^+$]: 291.1685; Found: 291.1688.

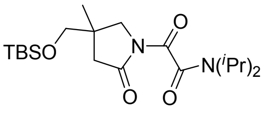

2e

^{1}H NMR (400 MHz, $CDCl_3$) δ 3.74 (d, *J* = 11.2 Hz, 1H), 3.51 (m, 2H), 3.46-3.37 (m, 3H), 2.66 (d, *J* = 17.6 Hz, 1H), 2.28 (d, *J* = 19.0 Hz, 1H), 1.49 (d, *J* = 6.7 Hz, 6H), 1.20 (d, *J* = 6.6 Hz, 6H), 1.15 (s, 3H), 0.87 (s, 9H), 0.04 (s, 6H); ^{13}C NMR (101 MHz, $CDCl_3$) δ 174.37, 164.38, 163.04, 68.72, 52.01, 50.98, 45.91, 42.12, 37.95, 25.93, 23.12, 20.46, 18.37, -5.45; HRMS Calcd for $C_{20}H_{38}N_2NaO_4Si$ [M+Na$^+$]: 421.2499; Found: 421.2496.

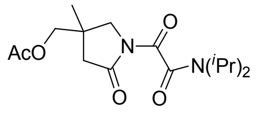

2f

^{1}H NMR (400 MHz, $CDCl_3$) δ 3.98 (s, 2H), 3.74 (d, *J* = 11.7 Hz, 1H), 3.61-3.41 (m, 3H), 2.61 (d, *J* = 17.2 Hz, 1H), 2.40 (d, *J* = 17.6 Hz, 1H), 2.09 (s, 3H), 1.48 (d, *J* = 6.8 Hz, 6H), 1.30-1.16 (m, 9H); ^{13}C NMR (101 MHz, $CDCl_3$) δ 173.27, 170.81, 164.12, 162.73, 69.32, 52.37, 51.02, 45.95, 42.63, 36.06, 23.25, 20.81, 20.48, 19.85; HRMS Calcd for $C_{16}H_{26}N_2NaO_5$ [M+Na$^+$]: 349.1739; Found: 349.1744.

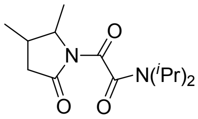

2g

^{1}H NMR (400 MHz, $CDCl_3$, ratio of rotamers: 2.9:1) δ 4.36 (s, 0.25H), 3.93 (m, 0.75H), 3.50 (m, 2H), 2.82 (m, 0.76H), 2.53 (d, *J* = 12.1 Hz, 0.54H), 2.37 (d, *J* = 8.2 Hz, 0.27H), 2.12 (m, 1.54H), 1.48 (d, *J* = 6.6 Hz, 6H), 1.37 (d, *J* = 6.4 Hz, 2.29H), 1.24-1.16 (m, 6.96H), 1.12 (d, *J* = 6.9 Hz, 2.28H), 1.08 (d, *J* = 6.5 Hz, 0.80H); ^{13}C NMR (101 MHz, $CDCl_3$) δ 174.66, 174.36, 164.60, 163.82, 163.13, 59.30, 57.29, 50.96, 45.85, 38.67, 38.24, 33.73, 31.02, 20.50, 20.08, 18.10, 14.29; HRMS Calcd for $C_{14}H_{25}N_2O_3$ [M+H$^+$]: 269.1865; Found: 269.1873.

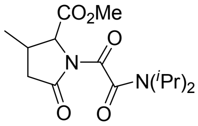

2h

^{1}H NMR (400 MHz, $CDCl_3$, ratio of rotamers: 2.3:1) δ 4.67 (d, *J* = 8.6 Hz, 0.28H), 4.33 (d, *J* = 2.8 Hz, 0.65H), 3.83-3.76 (m, 3H), 3.76-3.67 (m, 0.81H), 3.65-3.56 (m, 0.65H), 3.54-3.40 (m, 1H), 2.78 (m, 1H), 2.62 (m, 0.32H), 2.58-2.48 (m, 0.81H), 2.35 (m, 0.30H), 2.22 (m, 0.70H), 1.60-1.38 (m, 6H), 1.27 (d, *J* = 7.0 Hz, 2H), 1.24-1.13 (m, 6H), 1.10 (d, *J* = 6.9 Hz, 1H); ^{13}C NMR (101 MHz, $CDCl_3$) δ 173.58, 173.34, 163.92, 163.71, 162.35, 162.25, 63.21, 60.74, 52.94, 52.47, 50.66, 50.60, 45.90, 38.85, 38.42, 30.79, 29.73, 20.94, 20.71, 20.30, 20.18, 20.02, 19.42, 15.21; HRMS Calcd for $C_{15}H_{25}N_2O_5$ [M+H$^+$]: 313.1763; Found: 313.1758.

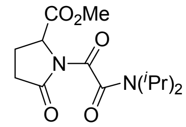

2i

^{1}H NMR (400 MHz, $CDCl_3$) δ 4.74 (m, 1H), 3.77 (s, 3H), 3.73 (d, *J* = 4.9 Hz, 1H), 3.48 (m, 1H), 2.72-2.51 (m, 2H), 2.48-2.36 (m, 1H), 2.23-2.09 (m, 1H), 1.47 (m, 6H), 1.24-1.12 (m, 6H); ^{13}C NMR (101 MHz, $CDCl_3$) δ 173.76, 171.22, 163.70, 162.28, 56.27, 52.98, 50.63, 45.88, 30.64,

22.34, 20.69, 20.29, 19.98, 19.36; HRMS Calcd for $C_{14}H_{22}N_2NaO_5$ [M+H$^+$]: 321.1426; Found: 321.1425.

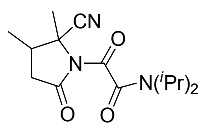

2j

^{1}H NMR (400 MHz, $CDCl_3$, ratio of rotamers: 4.2:1) δ 3.54 (m, 2H), 3.00-2.81 (m, 0.39H), 2.81-2.59 (m, 1H), 2.50-2.24 (m, 1.65H), 1.89 (s, 2H), 1.68 (s, 1H), 1.56-1.43 (m, 6H), 1.42-1.35 (m, 2H), 1.27 (d, J = 6.9 Hz, 1H), 1.23 (dd, J = 6.6, 2.0 Hz, 6H); ^{13}C NMR (101 MHz, $CDCl_3$) δ 172.43, 171.50, 163.80, 163.05, 161.77, 161.59, 116.91, 60.46, 57.40, 51.27, 51.23, 46.13, 38.79, 37.87, 37.29, 37.00, 22.69, 20.69, 20.15, 20.05, 19.42, 17.79, 14.86, 13.57; HRMS Calcd for $C_{15}H_{23}N_3NaO_3$ [M+Na$^+$]: 316.1637; Found: 316.1641.

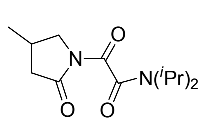

2k

^{1}H NMR (400 MHz, $CDCl_3$) δ 3.97 (dd, J = 11.3, 7.8 Hz, 1H), 3.61-3.42 (m, 2H), 3.33 (m, 1H), 2.71 (m, 1H), 2.62-2.47 (m, 1H), 2.24 (m, 1H), 2.04 (d, J = 10.5 Hz, 1H), 1.48 (d, J = 6.7 Hz, 6H), 1.19 (t, J = 7.3 Hz, 9H); ^{13}C NMR (101 MHz, $CDCl_3$) δ 174.66, 164.23, 163.02, 51.00, 50.72, 45.92, 40.45, 26.69, 20.46, 19.91, 19.23; HRMS Calcd for $C_{13}H_{22}N_2NaO_3$ [M+Na$^+$]: 277.1528; Found: 277.1530.

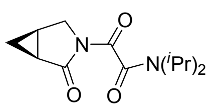

2l

^{1}H NMR (400 MHz, $CDCl_3$) δ 3.84-3.70 (m, 2H), 3.61-3.33 (m, 2H), 2.10-1.91 (m, 2H), 1.42 (t, J = 7.1 Hz, 6H), 1.29 (m, 1H), 1.23-1.06 (m, 6H), 0.94 (s, 1H); ^{13}C NMR (101 MHz, $CDCl_3$) δ 174.37, 164.71, 162.85, 50.91, 45.75, 45.53, 20.94, 20.47, 20.00, 19.48, 13.35, 13.31; HRMS Calcd for $C_{13}H_{20}N_2NaO_3$ [M+Na$^+$]: 275.1372; Found: 275.1379.

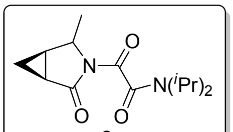

2m

^{1}H NMR (400 MHz, $CDCl_3$, ratio of rotamers: 1.7:1) δ 4.36 (m, 0.38H), 4.23 (s, 0.59H), 3.64-3.30 (m, 2H), 2.16 (m, 0.38H), 2.01 (d, J = 12.9 Hz, 1H), 1.81 (m, 0.76H), 1.55-1.37 (m, 9H), 1.32-1.15 (m, 7H), 1.02 (d, J = 28.5 Hz, 1H); ^{13}C NMR (101 MHz, $CDCl_3$) δ 165.46, 164.70, 163.16, 162.94, 53.63, 50.99, 50.91, 45.80, 45.78, 21.45, 20.68, 20.54, 20.35, 20.14, 19.47, 13.27, 9.95; HRMS Calcd for $C_{14}H_{22}N_2NaO_3$ [M+Na$^+$]: 289.1528; Found: 289.1538.

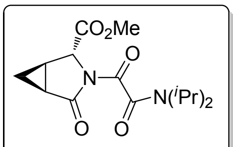

2n-trans

^1H NMR (400 MHz, $CDCl_3$) δ 4.64 (s, 1H), 3.81 (s, 4H), 3.47 (m, 1H), 2.14 (m, 1H), 2.10-2.03 (m, 1H), 1.49 (d, J = 6.8 Hz, 3H), 1.43 (d, J = 6.8 Hz, 3H), 1.34 (m, 1H), 1.24 (d, J = 6.6 Hz, 3H), 1.16 (d, J = 6.6 Hz, 3H), 1.06 (s, 1H); ^{13}C NMR (101 MHz, $CDCl_3$) δ 172.99, 169.79, 164.14, 162.37, 57.59, 53.11, 50.62, 45.88, 20.67, 20.26, 20.02, 19.89, 19.37, 16.66, 12.81; HRMS Calcd for $C_{15}H_{22}N_2NaO_5$ [$M+Na^+$]: 333.1426; Found: 333.1423.

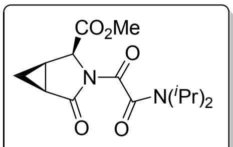

2n-cis

^1H NMR (400 MHz, $CDCl_3$) δ 4.76 (d, J = 6.4 Hz, 1H), 3.81 (s, 3H), 3.70 (s, 1H), 3.53-3.39 (m, 1H), 2.44-2.29 (m, 1H), 2.16 (s, 1H), 1.48 (t, J = 8.2 Hz, 6H), 1.27-1.11 (m, 8H); ^{13}C NMR (101 MHz, $CDCl_3$) δ 172.48, 168.91, 163.80, 161.83, 56.52, 52.88, 50.35, 45.88, 21.42, 21.12, 20.10, 19.83, 19.49, 16.46, 9.77; HRMS Calcd for $C_{15}H_{22}N_2NaO_5$ [$M+Na^+$]: 333.1426; Found: 333.1425.

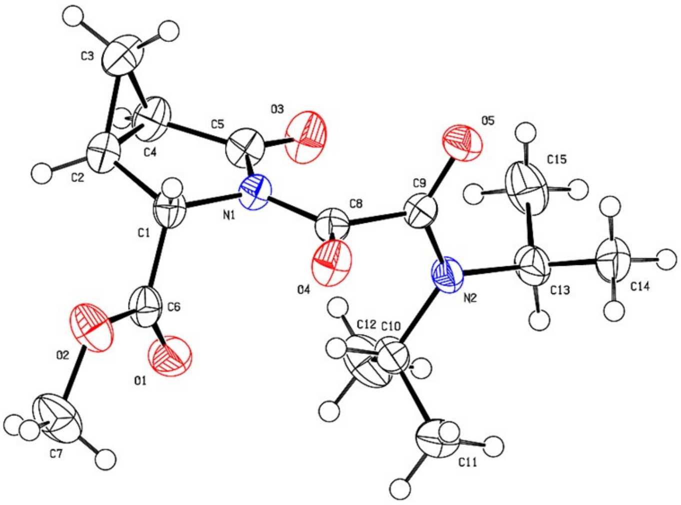

Figure S1. ORTEP diagram of **2n-trans** showing the atom–numbering scheme. Thermal ellipsoids are drawn at the 20% probability level.

X–Ray Data for 2n-trans: Intensity data were collected with a Rigaku Mercury CCD area detector in ω scan mode using Mo Kαradiation (λ = 0.71070 Å). The diffracted intensities were corrected for Lorentz polarization effects and empirical absorption corrections. Details of the intensity data collection and crystal data are given in Table 2. The structures were solved by direct methods and refined by fullmatrix least–squares procedures based on $|F|^2$. All the non–hydrogen atoms were refined anisotropically. All the H atoms were held stationaryand included in the

structure factor calculation in the final stage of full–matrix least–squares refinement. The structures were solved and refinedusing SHELEXL–97 programs.

Table S2. Crystal data and structure refinement for **2n-trans**.

Empirical formula	$C_{15}H_{22}N_2O_5$
Formula weight	310.34
Temperature/K	293(2)
Crystal system	orthorhombic
Space group	$P2_12_12_1$
a/Å	10.7542(4)
b/Å	11.2346(6)
c/Å	13.4066(6)
α/°	90
β/°	90
γ/°	90
Volume/Å^3	1619.78(13)
Z	4
ρ_{calc}g/cm^3	1.273
μ/mm^{-1}	0.096
F(000)	664.0
Crystal size/mm^3	0.3 × 0.29 × 0.19
Radiation	MoKα (λ = 0.71073)
2Θ range for data collection/°	6.062 to 50.986
Index ranges	-9 ≤ h ≤ 13, -13 ≤ k ≤ 10, -12 ≤ l ≤ 16
Reflections collected	5351
Independent reflections	2841 [R_{int} = 0.0285, R_{sigma} = 0.0458]
Data/restraints/parameters	2841/0/204
Goodness-of-fit on F^2	1.015
Final R indexes [I>=2σ (I)]	R_1 = 0.0407, wR_2 = 0.0827
Final R indexes [all data]	R_1 = 0.0536, wR_2 = 0.0885
Largest diff. peak/hole / e Å^{-3}	0.15/-0.20
Flack parameter	-1.0(8)

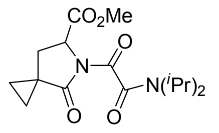

2o-1

^{1}H NMR (400 MHz, $CDCl_3$) δ 4.83 (m, 1H), 3.79 (d, *J* = 7.0 Hz, 4H), 3.49 (m, 1H), 2.82-2.60 (m, 1H), 2.00 (d, *J* = 13.1 Hz, 1H), 1.50 (m, 6H), 1.42 (s, 1H), 1.25 (s, 1H), 1.19 (m, 6H), 1.01-0.89 (m, 2H); ^{13}C NMR (101 MHz, $CDCl_3$) δ 175.92, 171.38, 163.64, 162.48, 54.24, 53.01, 50.58, 45.91, 30.69, 22.79, 20.77, 20.39, 19.99, 19.53, 18.68; HRMS Calcd for $C_{16}H_{24}N_2NaO_5$ [M+Na]: 347.1583; Found: 347.1585.

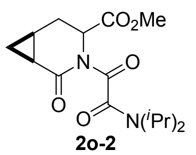

2o-2

^{1}H NMR (400 MHz, $CDCl_3$) δ 4.72 (t, *J* = 6.0 Hz, 1H), 3.78 (s, 3H), 3.73-3.60 (m, 1H), 3.47 (m, 1H), 2.63-2.45 (m, 1H), 2.20 (m, 1H), 1.91-1.79 (m, 1H), 1.77-1.69 (m, 1H), 1.46 (m, 6H), 1.37-1.32 (m, 1H), 1.28 (d, *J* = 6.5 Hz, 3H), 1.17 (d, *J* = 6.6 Hz, 4H); ^{13}C NMR (101 MHz, $CDCl_3$) δ 172.88, 171.06, 166.28, 163.01, 54.95, 53.01, 50.85, 45.71, 26.22, 20.49, 20.20, 19.66, 19.55, 18.14, 14.91; HRMS Calcd for $C_{16}H_{24}N_2NaO_5$ [M+Na]: 347.1583; Found: 347.1584.

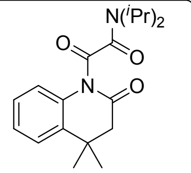

2p

^{1}H NMR (400 MHz, $CDCl_3$) δ 7.69 (m, 1H), 7.35 (m, 1H), 7.31-7.19 (m, 2H), 3.81-3.68 (m, 1H), 3.52 (m, 1H), 2.59 (s, 2H), 1.51 (d, *J* = 6.8 Hz, 6H), 1.37 (s, 6H), 1.27 (d, *J* = 6.6 Hz, 6H); ^{13}C NMR (101 MHz, $CDCl_3$) δ 172.63, 165.40, 163.27, 137.56, 133.99, 126.94, 126.58, 124.04, 122.92, 51.23, 47.70, 45.82, 33.62, 27.10, 20.09, 19.91; HRMS Calcd for $C_{19}H_{27}N_2O_3$ [M+H$^+$]: 331.2022; Found: 331.2025.

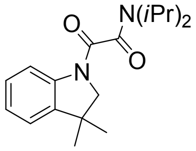

2p-1

^{1}H NMR (400 MHz, $CDCl_3$) δ 8.13 (d, *J* = 8.0 Hz, 0.69H), 7.24–7.05 (m, 3.19H), 4.00–3.93 (m, 1H), 3.79 (s, 1.52H), 3.75–3.72 (m, 0.27H), 3.56–3.49 (m, 1H), 1.56 (d, *J* = 6.8 Hz, 1.62H), 1.52 (d, *J* = 6.8 Hz, 4.53H), 1.35 (s, 6H), 1.25 (d, *J* = 6.6 Hz, 4.89H), 1.09 (s, 0.98H); ^{13}C NMR (151 MHz, $CDCl_3$) δ 164.61, 164.13, 162.74, 162.45, 142.25, 141.29, 140.49, 138.77, 127.91, 127.60,

125.16, 124.83, 123.02, 122.13, 117.44, 113.87, 62.64, 60.96, 51.11, 50.76, 46.12, 45.98, 40.72, 39.27, 28.06, 21.02, 20.71, 20.32, 20.07. This compound was known.[5]

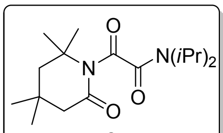

2q

^{1}H NMR (400 MHz, $CDCl_3$) δ 3.85–3.70 (m, 1H), 3.50–3.35 (m, 1H), 2.34 (s, 2H), 1.76 (s, 2H), 1.60 (s, 6H), 1.42 (d, *J* = 6.8 Hz, 6H), 1.24 (d, *J* = 6.6 Hz, 6H), 1.11 (s, 6H); ^{13}C NMR (101 MHz, $CDCl_3$) δ 174.88, 169.49, 163.94, 59.74, 52.64, 50.90, 47.65, 45.61, 30.07, 29.32, 20.01; HRMS Calcd for $C_{17}H_{31}N_2O_3$ [M+H^{+}]: 311.2335; Found: 311.2334.

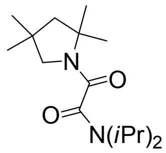

2q-1

^{1}H NMR (400 MHz, $CDCl_3$) δ 3.84 (m, 1H), 3.39 (m, 1H), 3.19 (s, 2H), 1.70 (s, 2H), 1.50 (d, *J* = 1.1 Hz, 6H), 1.40 (m, 6H), 1.17 (d, *J* = 6.7 Hz, 6H), 1.06 (s, 6H); ^{13}C NMR (101 MHz, $CDCl_3$) δ 165.06, 163.44, 62.53, 61.00, 55.92, 50.32, 45.48, 36.85, 27.37, 27.23, 27.22, 20.84, 20.17, 20.16. This compound was known.[5]

11. Palladium-catalyzed carbonylation of γ-C(sp^2)–H bonds

A mixture of oxalamide (0.2 mmol, 1.0 eq), $Pd(OAc)_2$ (4.5 mg, 0.1 eq), AgOAc (83.4 mg, 2.5 eq), *m*-CF_3PhCO_2H (11.4 mg, 0.3 eq) and mesitylene (0.3 mL) in a 20 mL glass vial was purged with CO (3-times), and sealed with a teflon septa. The vial was heated in an oil bath for 24 hours, then the reaction mixture was cooled to rt, and concentrated in vacuo. The resulting residue was purified by column chromatography on silica gel to give the product **4**.

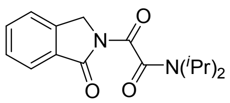

4a

^{1}H NMR (400 MHz, $CDCl_3$) δ 7.92 (d, *J* = 7.7 Hz, 1H), 7.74-7.65 (m, 1H), 7.59-7.46 (m, 2H), 4.85 (s, 2H), 3.68 (m, 1H), 3.55 (m, 1H), 1.57 (s, 6H), 1.24 (d, *J* = 6.6 Hz, 6H); ^{13}C NMR (101 MHz, $CDCl_3$) δ 167.13, 164.19, 162.85, 141.94, 134.67, 130.20, 129.04, 125.54, 123.86, 51.13, 47.01, 46.03, 20.52; HRMS Calcd for $C_{16}H_{21}N_2O_3$ [M+H^{+}]: 289.1552; Found: 289.1559.

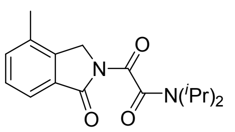

4b

^{1}H NMR (400 MHz, $CDCl_3$) δ 7.74 (d, *J* = 7.4 Hz, 1H), 7.47 (d, *J* = 7.3 Hz, 1H), 7.42 (m, 1H), 4.76 (s, 2H), 3.67 (m, 1H), 3.61–3.49 (m, 1H), 2.38 (s, 3H), 1.57 (s, 6H), 1.24 (d, *J* = 6.6 Hz, 6H); ^{13}C NMR (101 MHz, $CDCl_3$) δ 167.50, 164.24, 162.93, 140.93, 135.41, 133.73, 129.92, 129.29, 122.95, 51.15, 46.27, 46.04, 20.16, 17.61; HRMS Calcd for $C_{17}H_{22}N_2NaO_3$ [M+Na$^+$]: 325.1528; Found: 325.1520.

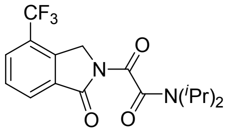

4c

^{1}H NMR (400 MHz, $CDCl_3$) δ 8.10 (d, *J* = 7.7 Hz, 1H), 7.93 (d, *J* = 7.7 Hz, 1H), 7.73-7.63 (m, 1H), 5.01 (s, 2H), 3.66 (m, 1H), 3.56 (m, 1H), 1.55 (s, 6H), 1.23 (d, *J* = 6.6 Hz, 6H); ^{13}C NMR (101 MHz, $CDCl_3$) δ 165.61, 163.82, 162.43, 139.22, 139.20, 132.00, 131.39, 131.35, 131.30, 131.26, 129.78, 129.04, 127.48, 127.24, 126.91, 126.57, 126.23, 124.77, 122.05, 119.34, 51.22, 46.46, 46.43, 46.14, 20.58, 19.94; HRMS Calcd for $C_{17}H_{20}F_3N_2O_3$ [M+H$^+$]: 357.1426; Found: 357.1411.

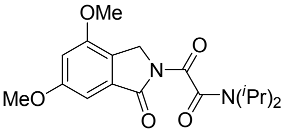

4d

^{1}H NMR (400 MHz, $CDCl_3$) δ 6.91 (s, 1H), 6.68 (d, *J* = 1.9 Hz, 1H), 4.67 (d, *J* = 7.2 Hz, 2H), 3.86 (s, 3H), 3.81 (s, 3H), 3.65 (m, 1H), 3.54 (m, 1H), 1.55 (s, 6H), 1.22 (d, *J* = 6.6 Hz, 6H); ^{13}C NMR (101 MHz, $CDCl_3$) δ 167.38, 164.07, 162.90, 162.35, 155.84, 132.00, 123.88, 105.28, 98.26, 55.99, 55.81, 51.06, 45.97, 44.70, 20.93, 20.15, 19.55; HRMS Calcd for $C_{18}H_{25}N_2O_5$ [M+H$^+$]: 349.1763; Found: 349.1757

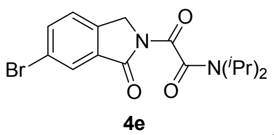

4e

^{1}H NMR (400 MHz, $CDCl_3$) δ 8.04 (s, 1H), 7.80 (dd, *J* = 8.1, 1.8 Hz, 1H), 7.43 (d, *J* = 8.1 Hz, 1H), 4.80 (s, 2H), 3.65 (m, 1H), 3.55 (m, 1H), 1.56 (d, *J* = 3.6 Hz, 6H), 1.24 (d, *J* = 6.6 Hz, 6H); ^{13}C NMR (101 MHz, $CDCl_3$) δ 165.74, 163.96, 162.60, 140.53, 137.65, 132.23, 128.52, 125.49, 123.08, 51.22, 46.86, 46.13, 20.96, 20.57, 20.13; HRMS Calcd for $C_{16}H_{19}BrN_2NaO_3$ [M+Na$^+$]: 389.0477; Found: 389.0467.

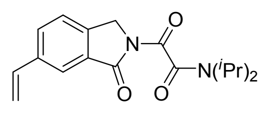

4f

^{1}H NMR (400 MHz, $CDCl_3$) δ 7.94 (s, 1H), 7.70 (dd, *J* = 7.9, 1.4 Hz, 1H), 7.49 (d, *J* = 8.0 Hz, 1H), 6.75 (dd, *J* = 17.6, 10.9 Hz, 1H), 5.84 (d, *J* = 17.6 Hz, 1H), 5.37 (d, *J* = 10.9 Hz, 1H), 4.83 (s, 2H), 3.66 (m, 1H), 3.62–3.50 (m, 1H), 1.57 (s, 6H), 1.24 (d, *J* = 6.6 Hz, 6H); ^{13}C NMR (101 MHz, $CDCl_3$) δ 167.12, 164.14, 162.84, 141.06, 138.90, 135.37, 132.83, 130.66, 123.98, 122.56, 116.26, 51.16, 46.94, 46.06, 20.95, 20.13; HRMS Calcd for $C_{18}H_{22}N_2NaO_3$ [M+Na$^+$]: 337.1528; Found:337.1525.

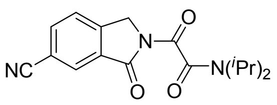

4g

^{1}H NMR (400 MHz, $CDCl_3$) δ 8.20 (d, *J* = 0.5 Hz, 1H), 7.96 (dd, *J* = 7.9, 1.5 Hz, 1H), 7.71 (dd, *J* = 8.0, 0.7 Hz, 1H), 4.93 (s, 2H), 3.66 (m, 1H), 3.57 (m, 1H), 1.56 (d, *J* = 6.6 Hz, 6H), 1.25 (d, *J* = 6.6 Hz, 6H); ^{13}C NMR (101 MHz, $CDCl_3$) δ 165.01, 163.82, 162.28, 145.82, 137.47, 131.68, 129.60, 125.24, 117.47, 113.71, 51.28, 47.32, 46.23, 20.60, 19.94; HRMS Calcd for $C_{17}H_{19}N_3NaO_3$ [M+Na$^+$]: 336.1324; Found: 336.1317.

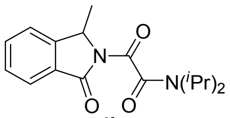

4h

^{1}H NMR (400 MHz, $CDCl_3$) δ 7.89 (d, *J* = 7.8 Hz, 1H), 7.68 (t, *J* = 7.5 Hz, 1H), 7.50 (t, *J* = 7.7 Hz, 2H), 5.21 (d, *J* = 6.1 Hz, 1H), 3.66 (m, 1H), 3.54 (m, 1H), 1.67 (s, 3H), 1.56 (d, *J* = 22.1 Hz, 6H), 1.24 (d, *J* = 6.6 Hz, 6H); ^{13}C NMR (101 MHz, $CDCl_3$) δ 167.11, 164.28, 163.05, 134.80, 130.11, 129.06, 128.43, 125.48, 122.93, 51.12, 45.97, 20.77, 20.31, 19.56, 18.85; HRMS Calcd for $C_{17}H_{22}N_2NaO_3$ [M+Na$^+$]: 325.1528; Found: 325.1537.

12. Allylamine reaction (Scheme 2)

A mixture of allylamine **5** or **7** (0.2 mmol, 1.0 eq), $Pd(OAc)_2$, AgOAc (83.4 mg, 2.5 eq), *m*-CF_3PhCO_2H (11.4 mg, 0.3 eq) and mesitylene (0.3 mL) in a 20 mL glass vial was purged with CO (3-times), and sealed with a teflon septa. The vial was heated in an oil bath for 24 hours, then the reaction mixture was cooled to rt, and concentrated in vacuo. The resulting residue was purified by column chromatography on silica gel to give the product **6** or **8**.

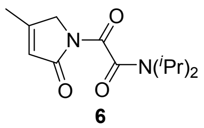

6

^{1}H NMR (400 MHz, $CDCl_3$) δ 5.87 (d, *J* = 1.4 Hz, 1H), 4.30 (s, 2H), 3.59 (m, 1H), 3.51 (m, 1H), 2.16 (s, 3H), 1.52 (d, *J* = 5.1 Hz, 6H), 1.21 (d, *J* = 6.6 Hz, 6H); ^{13}C NMR (101 MHz, $CDCl_3$) δ 169.60, 162.89, 161.74, 121.89, 52.03, 51.04, 45.97, 20.25, 16.23; HRMS Calcd for $C_{13}H_{20}N_2NaO_3$ [M+Na+]: 275.1372; Found: 275.1372.

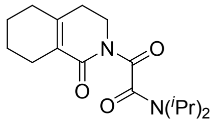

8

^{1}H NMR (400 MHz, $CDCl_3$) δ 4.23 (s, 1H), 3.68-3.39 (m, 3H), 2.52 (s, 1H), 2.26 (d, *J* = 40.5 Hz, 5H), 1.70 (s, 2H), 1.56 (d, *J* = 6.9 Hz, 2H), 1.48 (s, 6H), 1.22 (s, 6H); ^{13}C NMR (101 MHz, $CDCl_3$) δ 166.94, 165.05, 164.11, 153.00, 126.69, 51.03, 45.66, 39.69, 31.08, 29.32, 23.13, 22.08, 21.76, 20.60, 20.31, 19.87, 19.71; HRMS Calcd for $C_{17}H_{26}N_2NaO_3$ [M+Na+]: 329.1841; Found: 329.1846.

13. References

(1) (*a*) Z.-H. Guan, M. Chen, Z.-H. Ren, *J. Am. Chem. Soc.,* **2012**, *134*, 17490; (*b*) W. Li, Z. Duan, X. Zhang, H. Zhang, M. Wang, R. Jiang, H. Zeng, C. Liu, A. Lei, *Angew. Chem.; Int. Ed.,* **2015**, *54*, 1893; (*c*) B. D. Dangel, K. Godula, S. W. Youn, B. Sezen, D. Sames, *J. Am. Chem. Soc.,* **2002**, *124*, 11856; (*d*) E. Laga, A. García-Montero, F. J. Sayago, T. Soler, S. Moncho, C. Cativiela, M. Martínez, E. P. Urriolabeitia, *Chem. Eur. J.,* **2013**, *19*, 17398.

(2) He, G.; Zhao, Y.; Zhang, S.; Lu, C.; Chen, G. *J. Am. Chem. Soc.,* **2012**, *134*, 3.

(3) Mei, T.–S.; Wang, X.; Yu, J.–Q. *J. Am. Chem. Soc.,* **2009**, *131*, 10806.

(4) Zhao, Y.; Chen, G. *Org. Lett.,* **2011**, *13*, 4850.

(5) C. Wang, C. Chen, J. Zhang, J. Han, Q. Wang, K. Guo, P. Liu, M. Guan, Y. Yao, Y. Zhao, *Angew. Chem., Int. Ed.,* **2014**, *53*, 9884.

(6) He, G.; Lu, C.; Zhao, Y.; Nack, W. A.; Chen, G. *Org. Lett.,* **2012**, *14*, 2944.

(7) Kretschmer, M.; Menche, D. *Org, Lett.,* **2012**, *14*, 382.
