# Supplementary figures and images for: Oxalyl amide assisted palladium-catalyzed synthesis of pyrrolidones via carbonylation of γ-C(sp3)–H bonds of aliphatic amine substrates
Source: Chem Sci. 2015 May 19;6(8):4610–4. doi: 10.1039/c5sc00519a (PMC5861525; doi:10.1039/c5sc00519a)

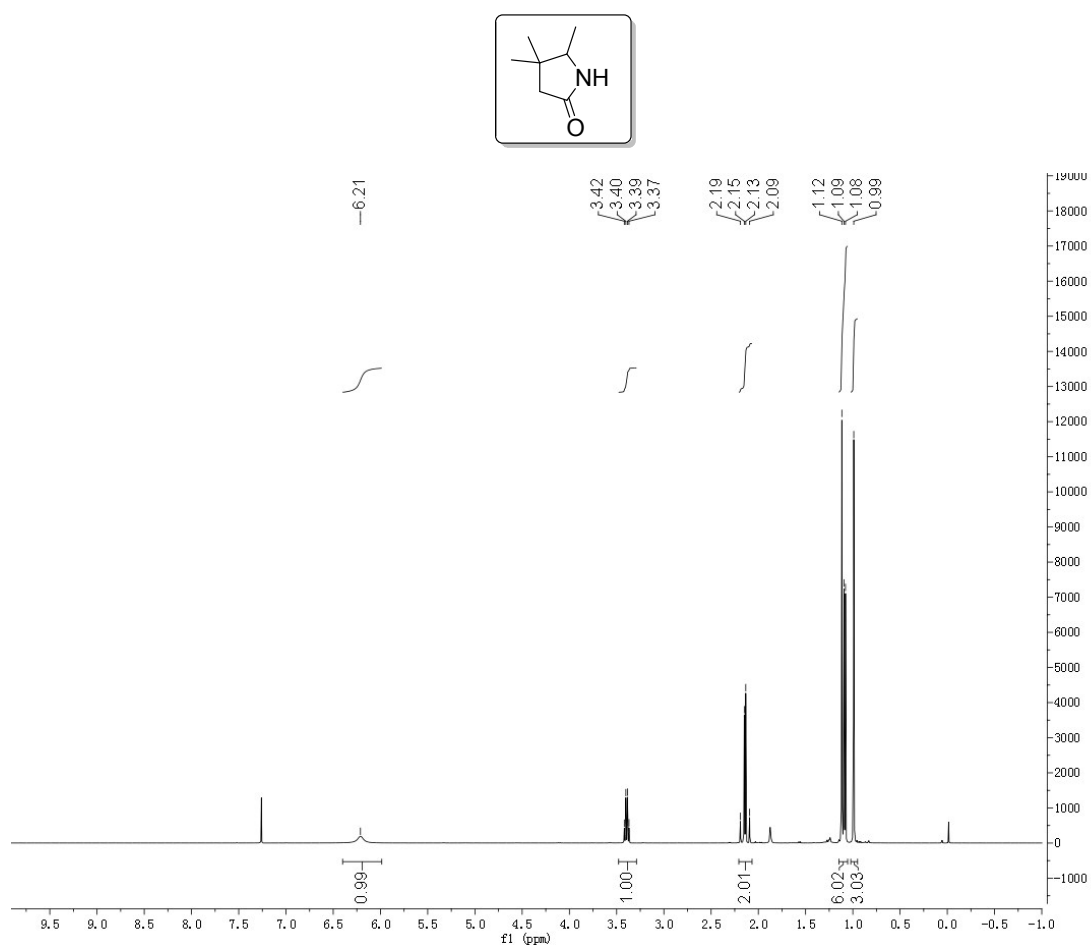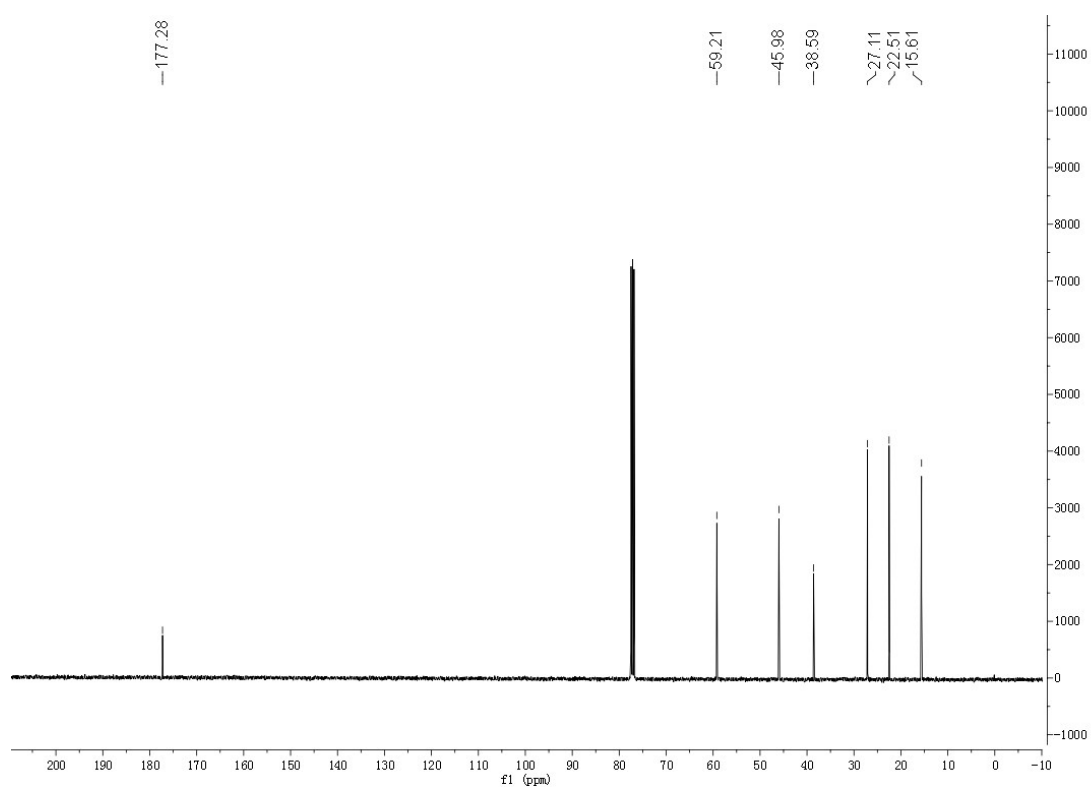

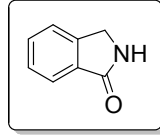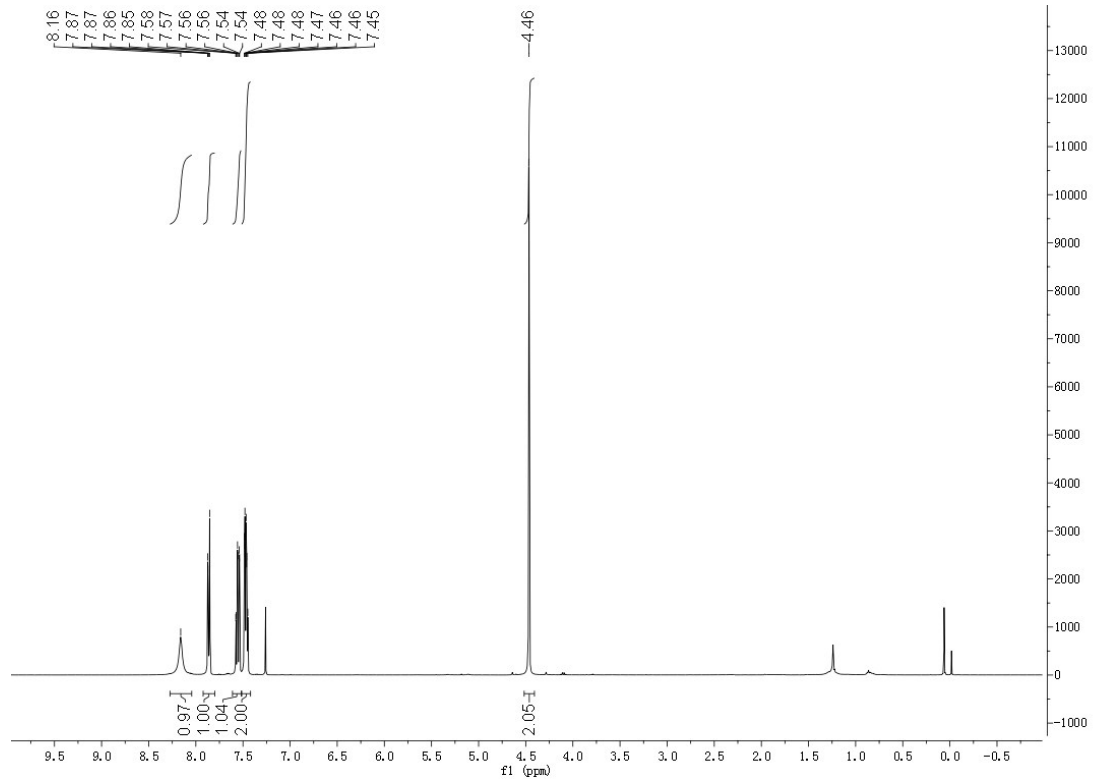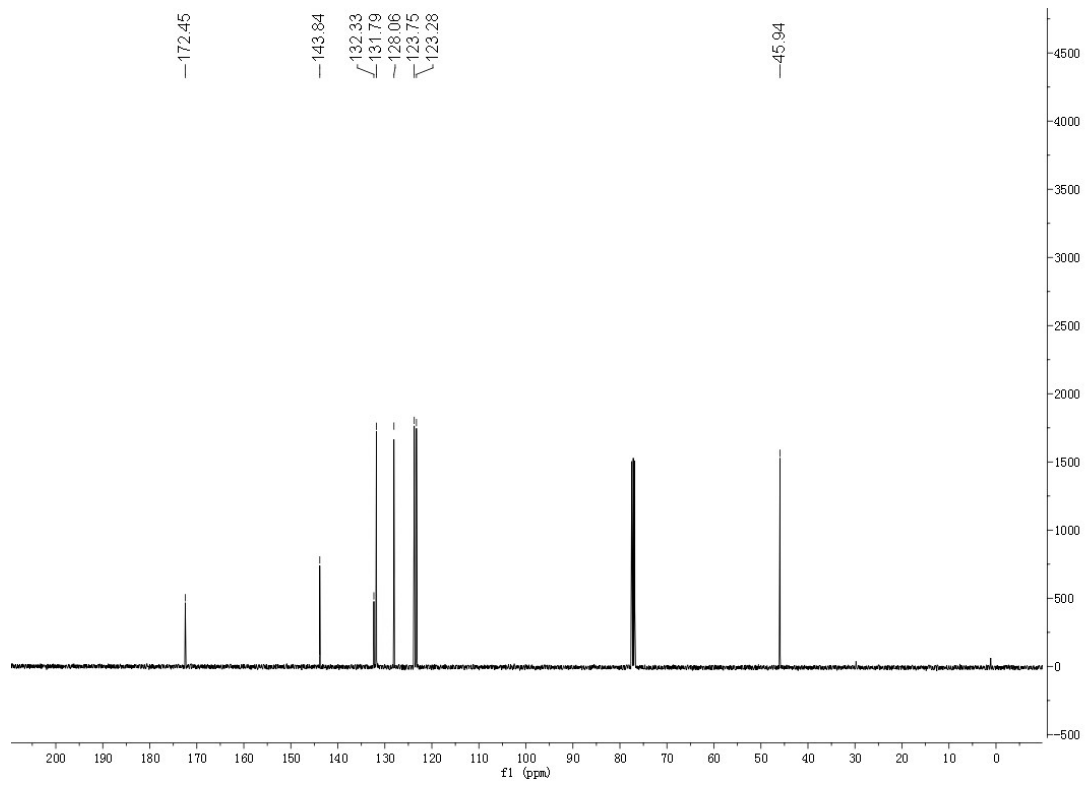

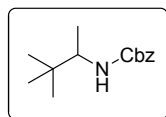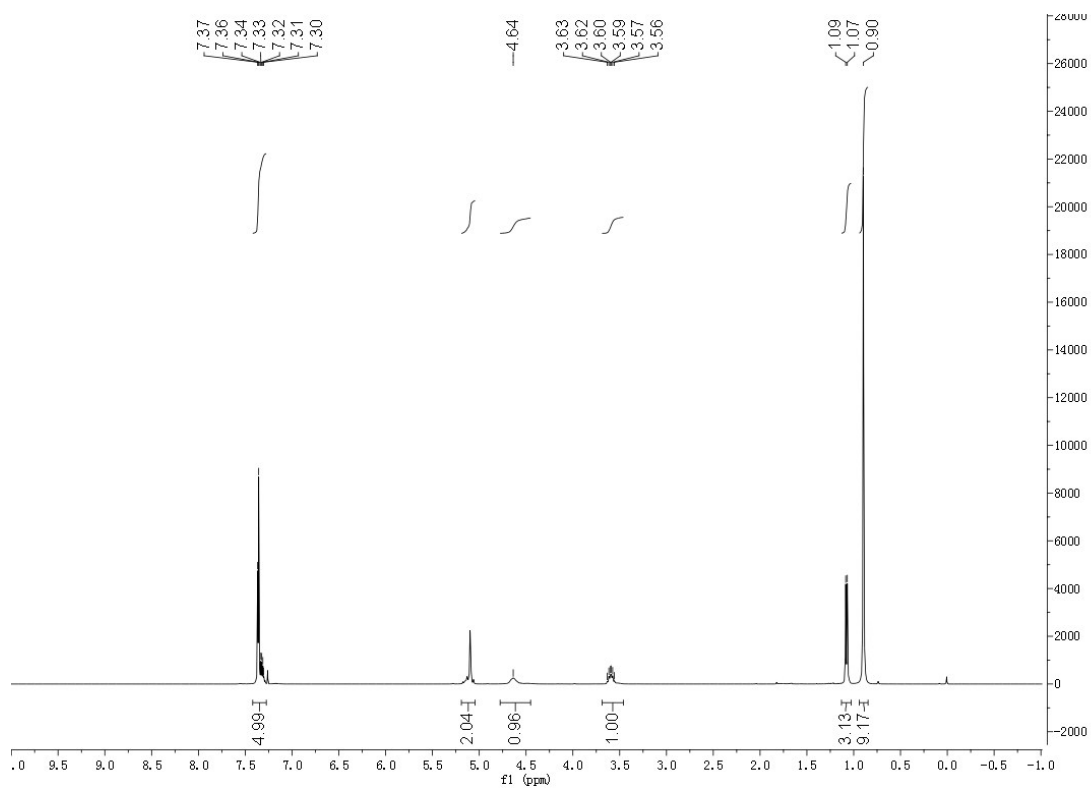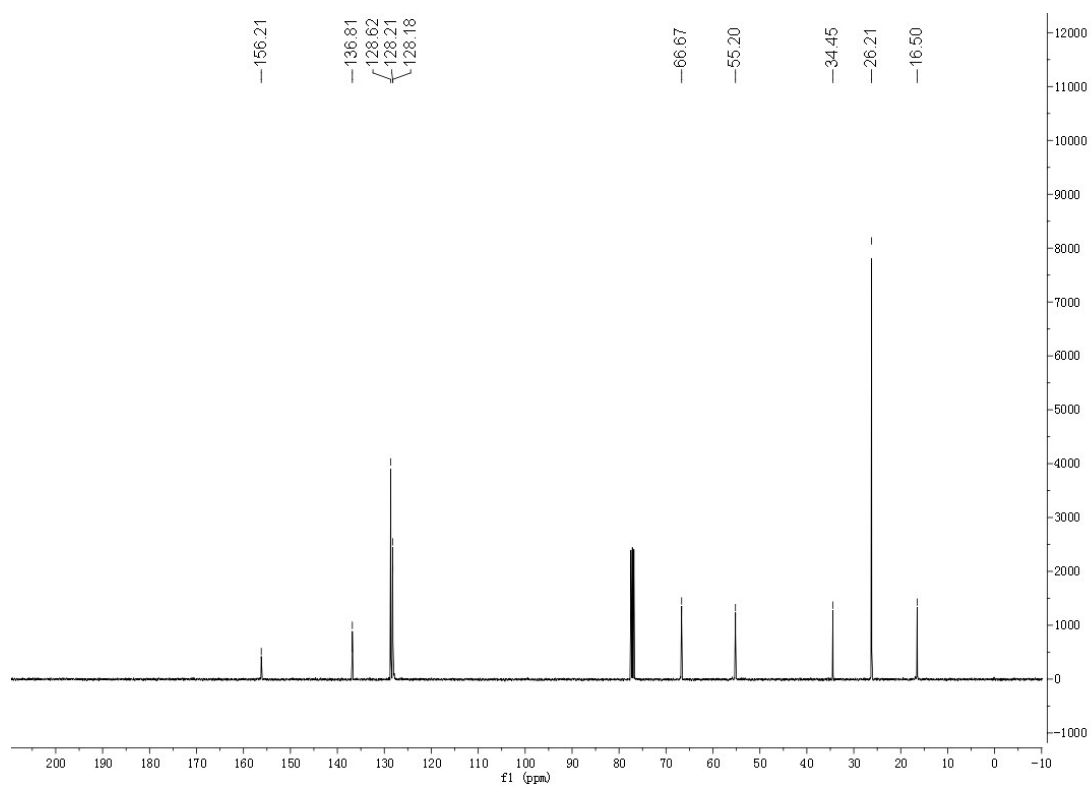

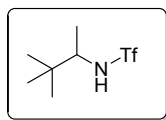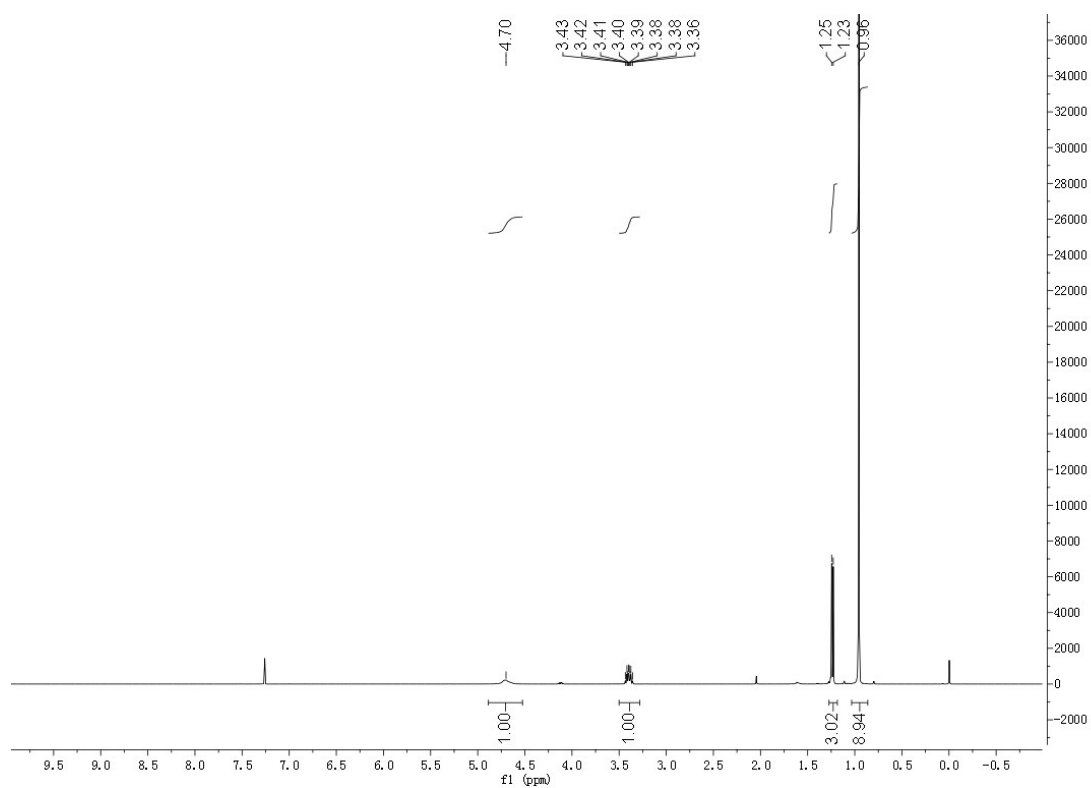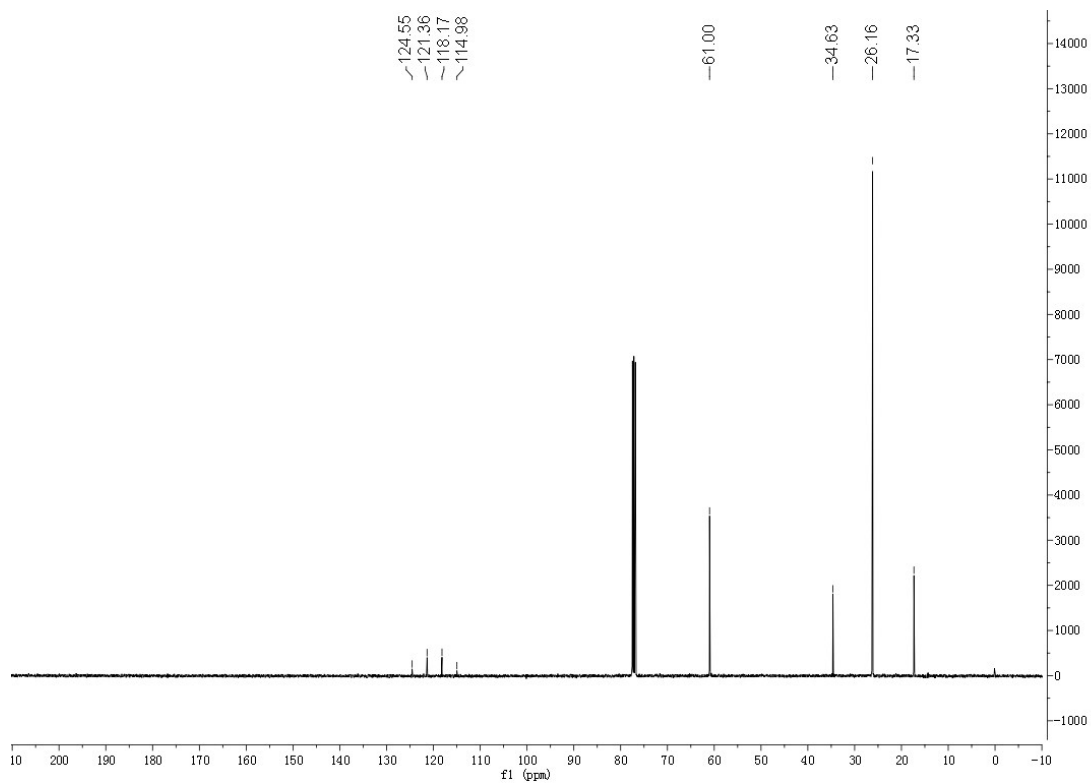

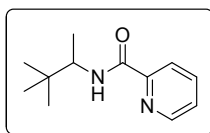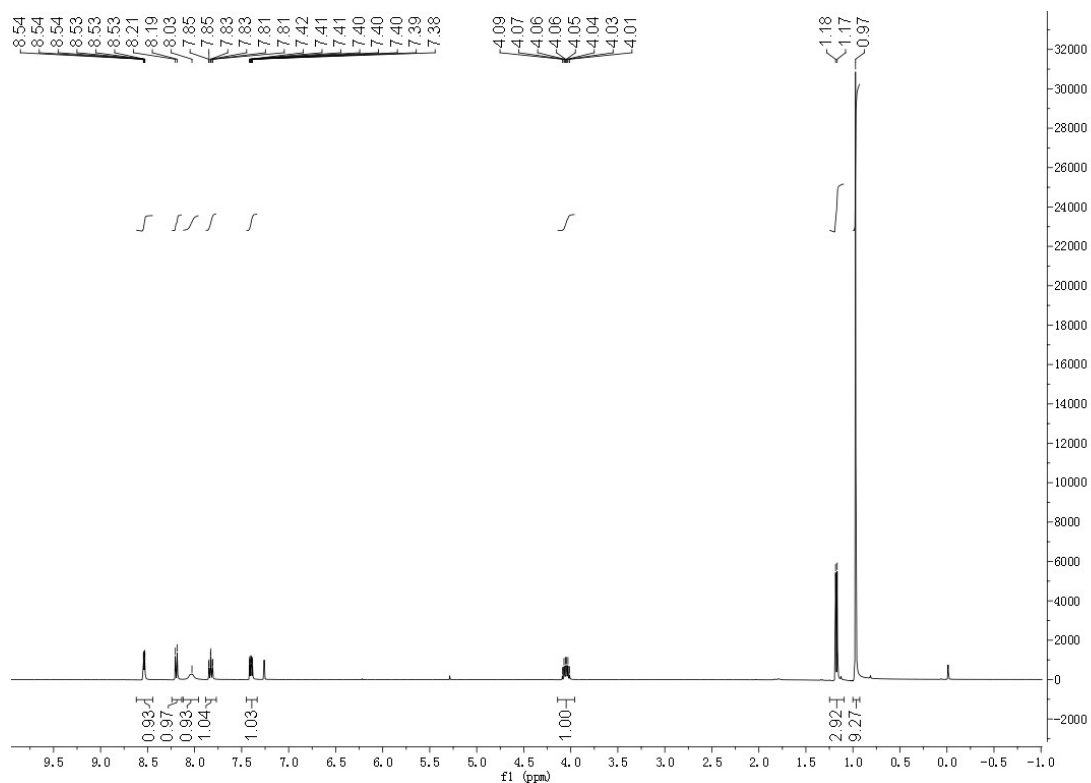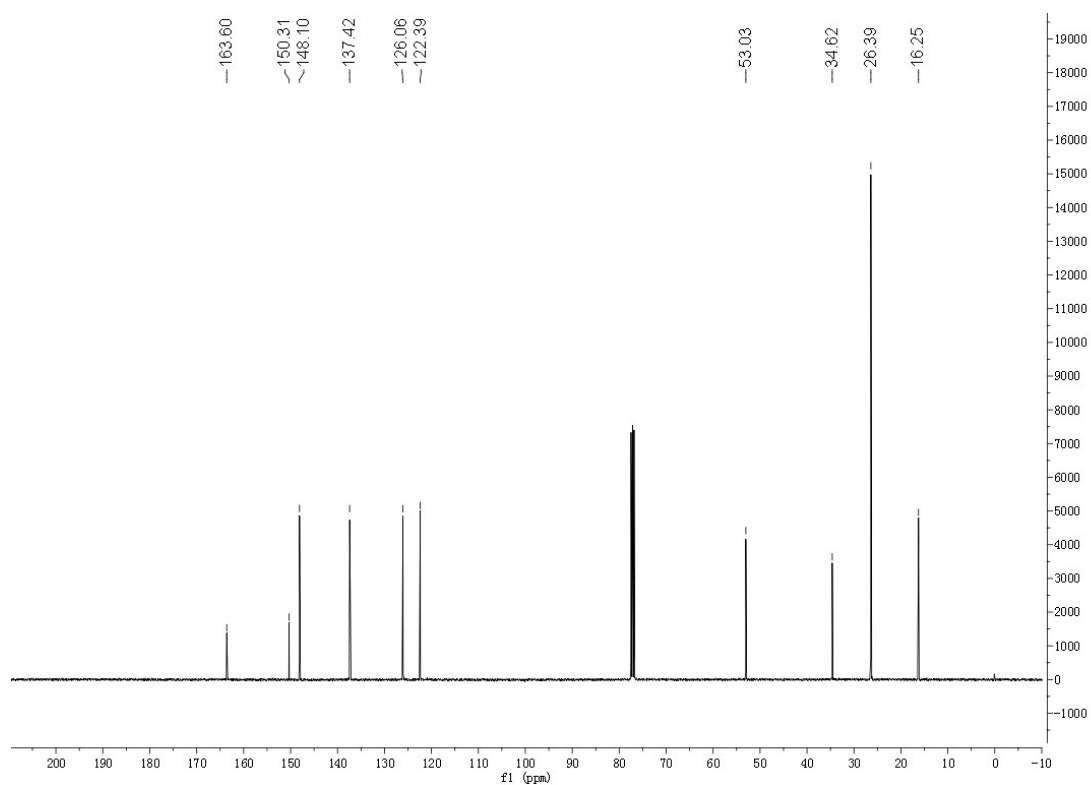

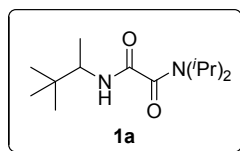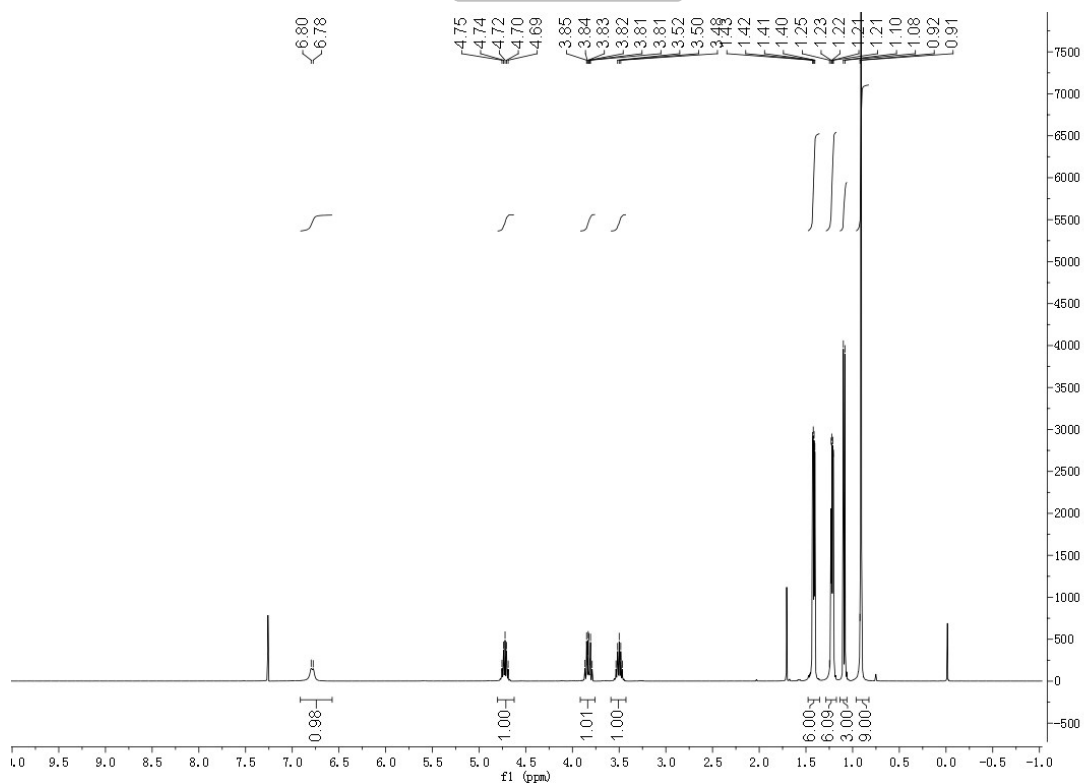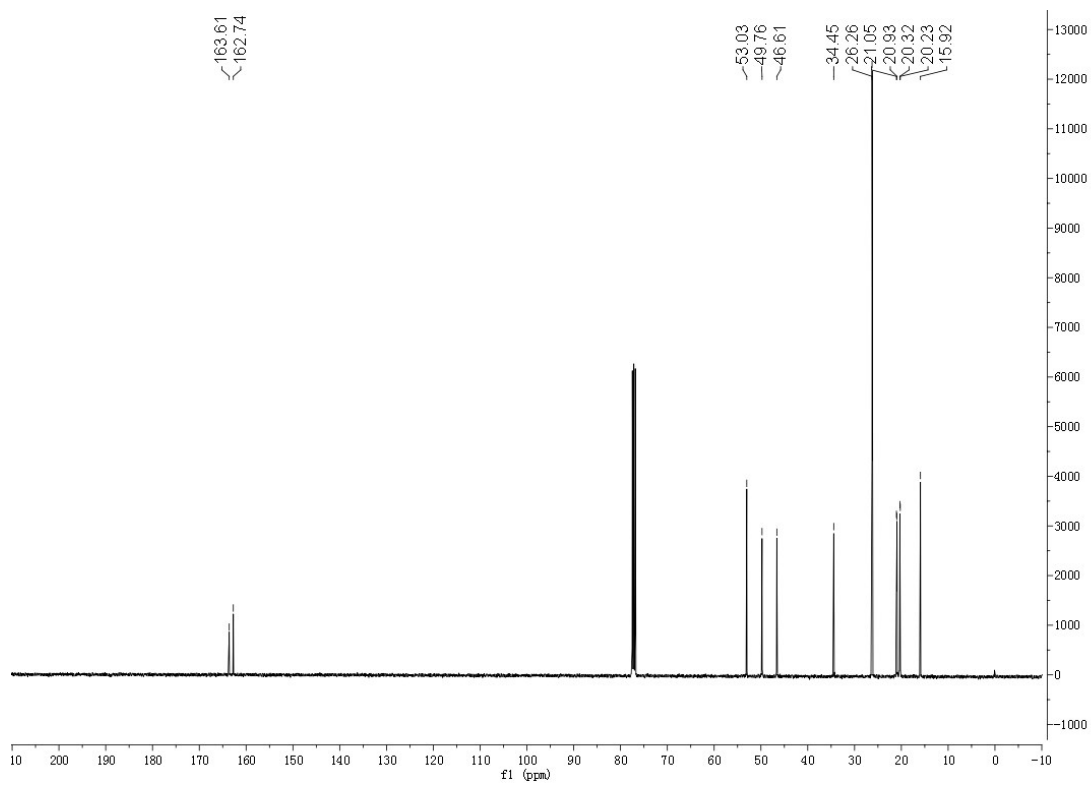

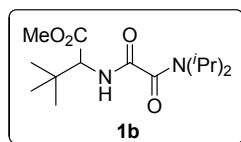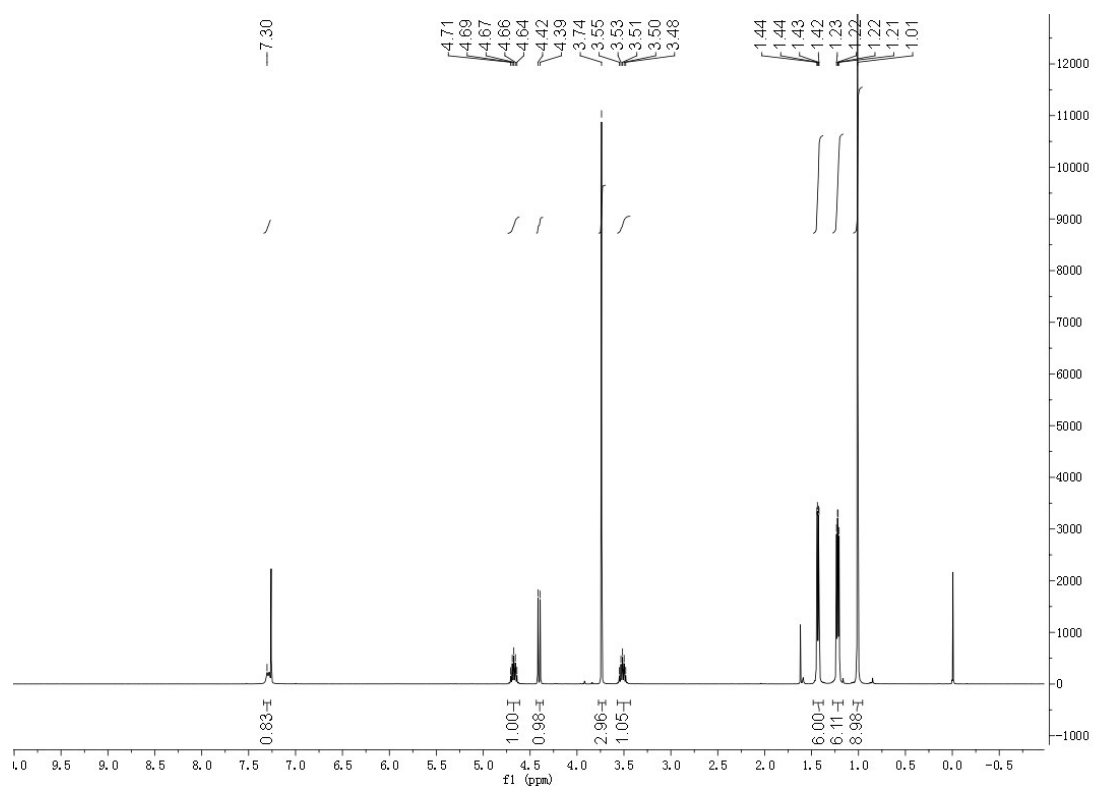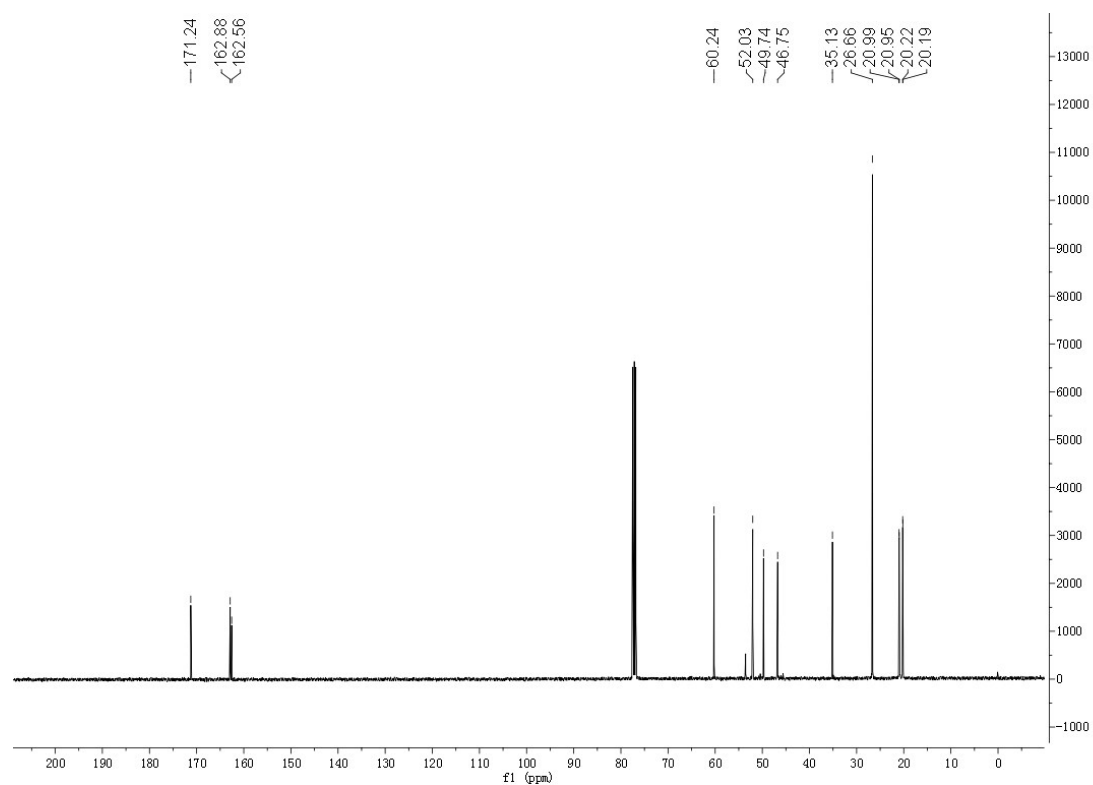

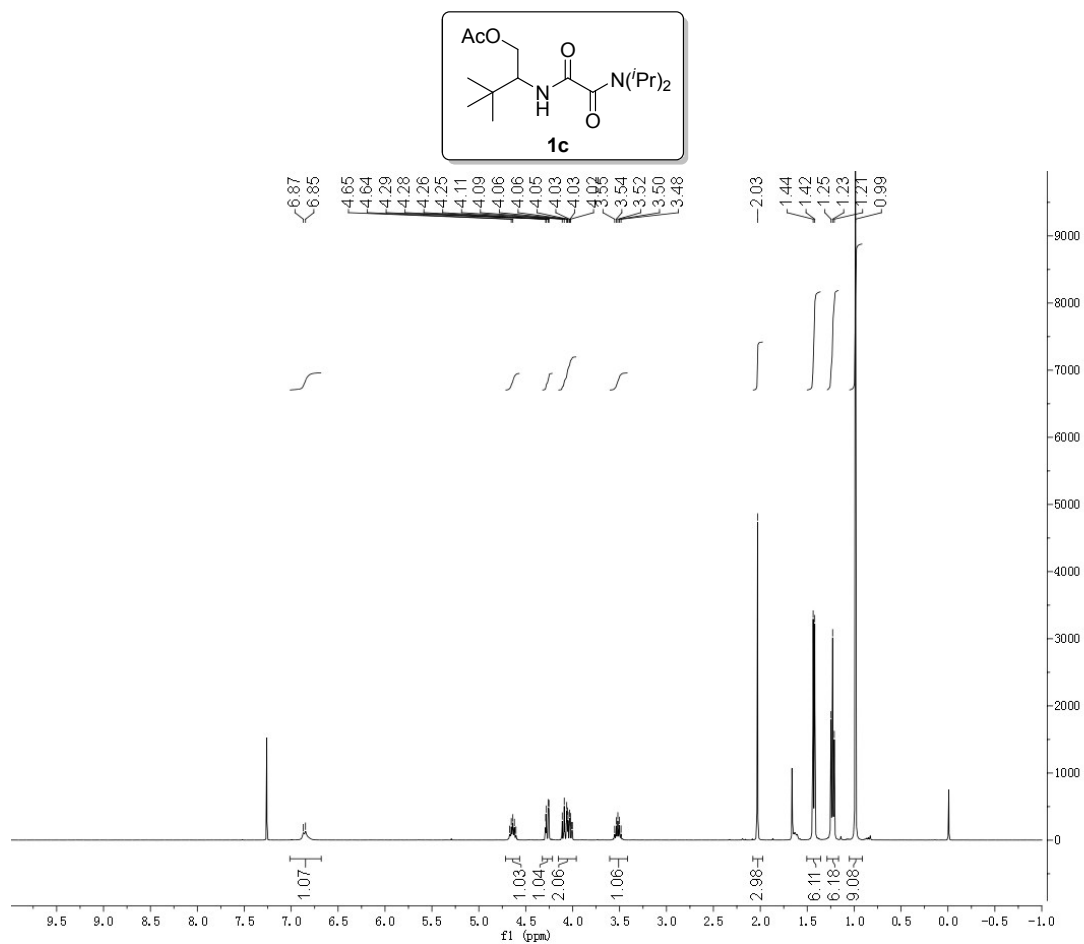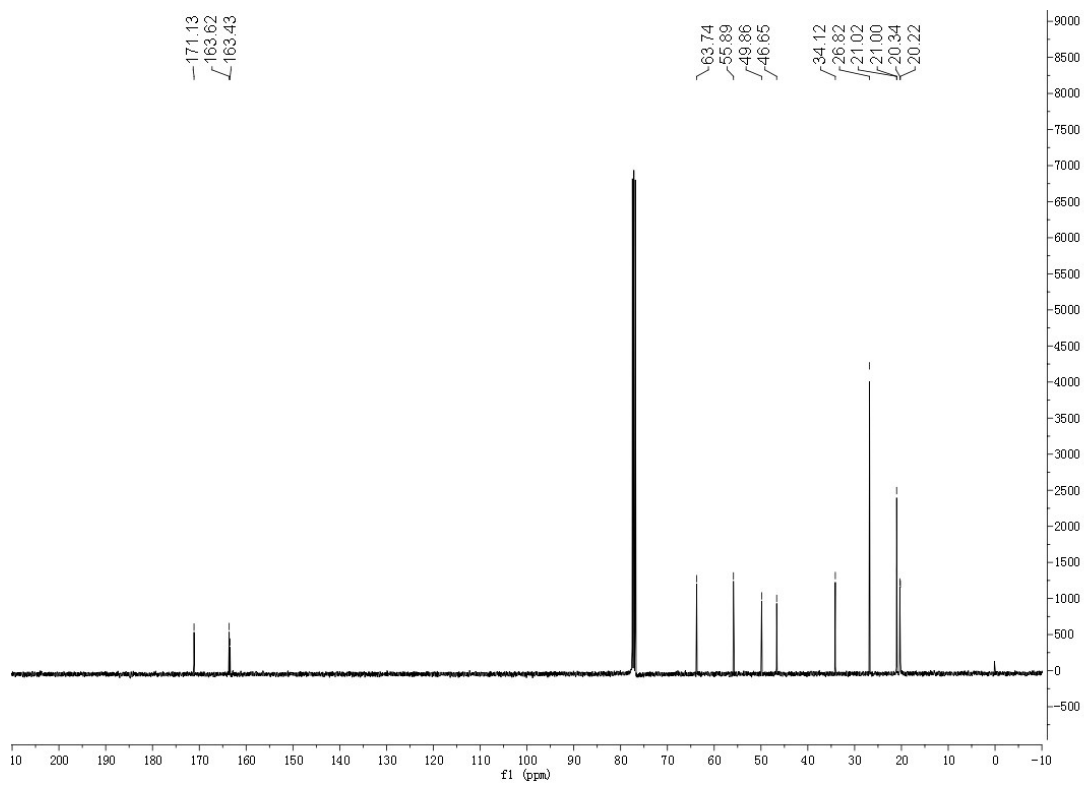

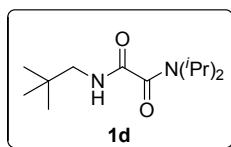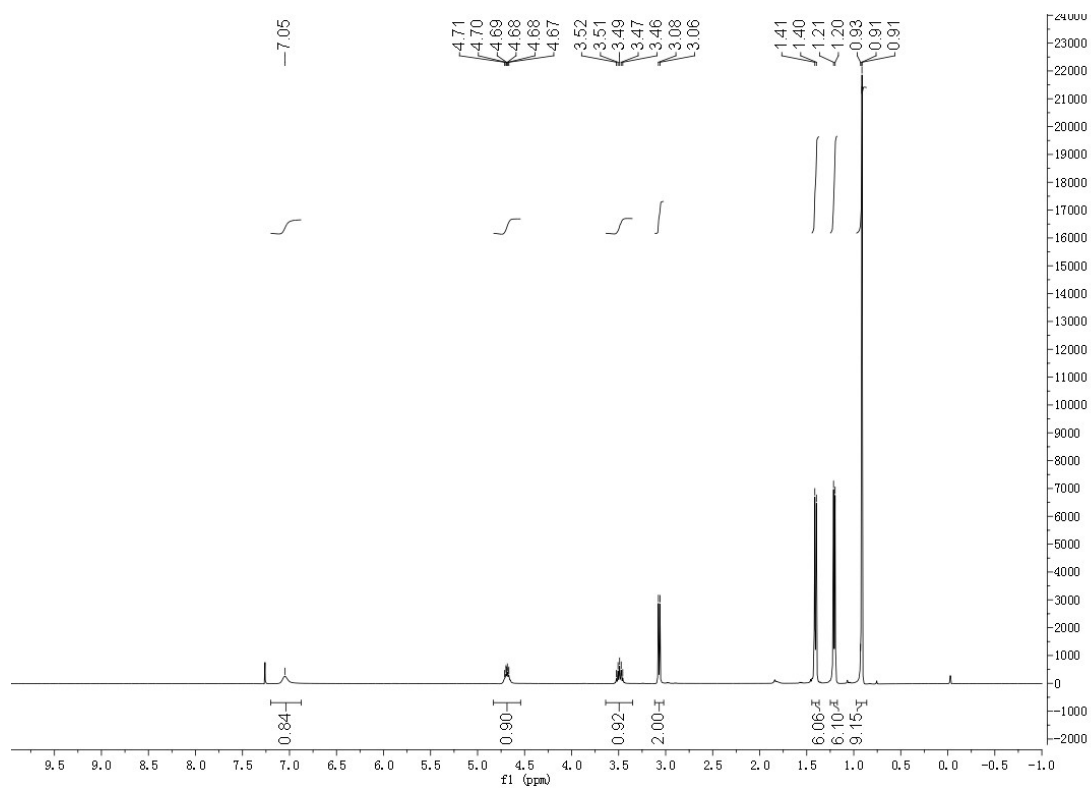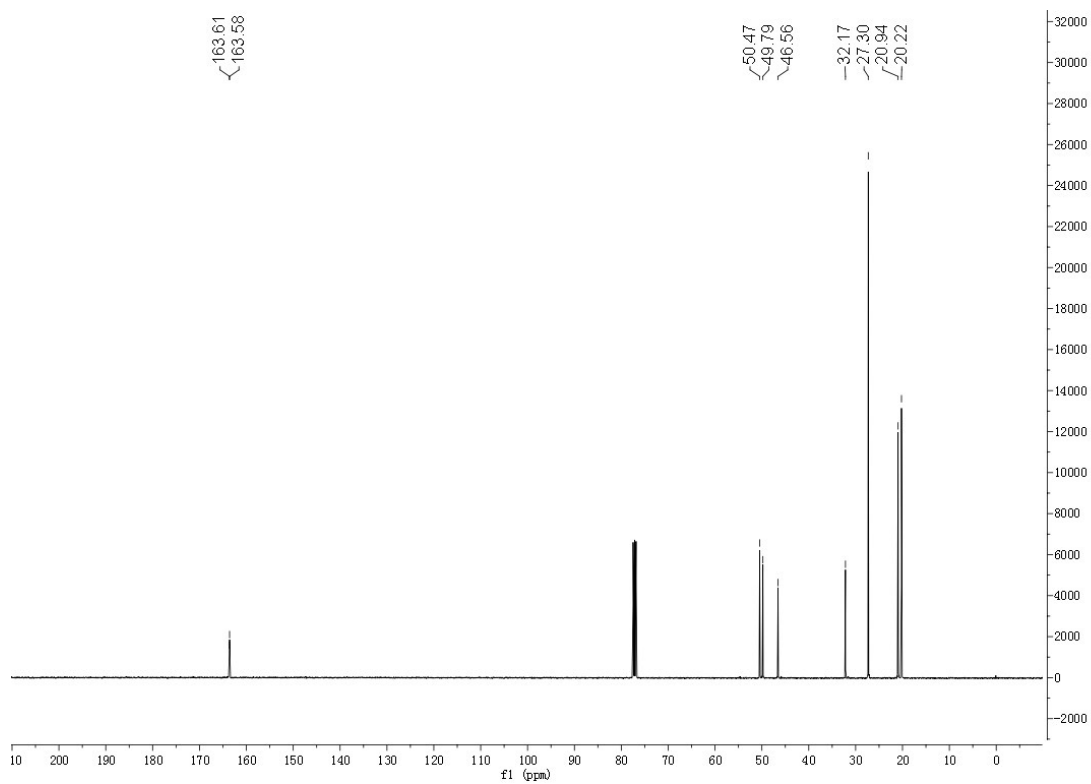



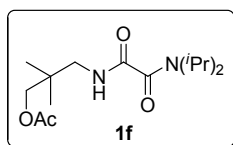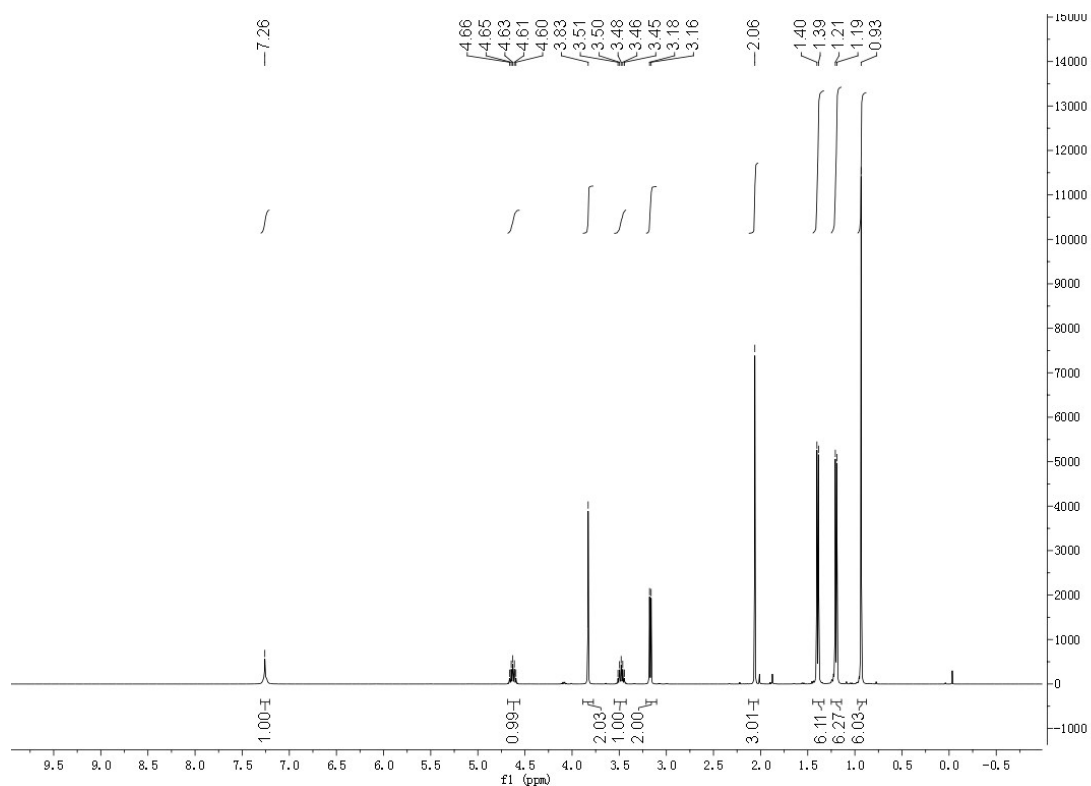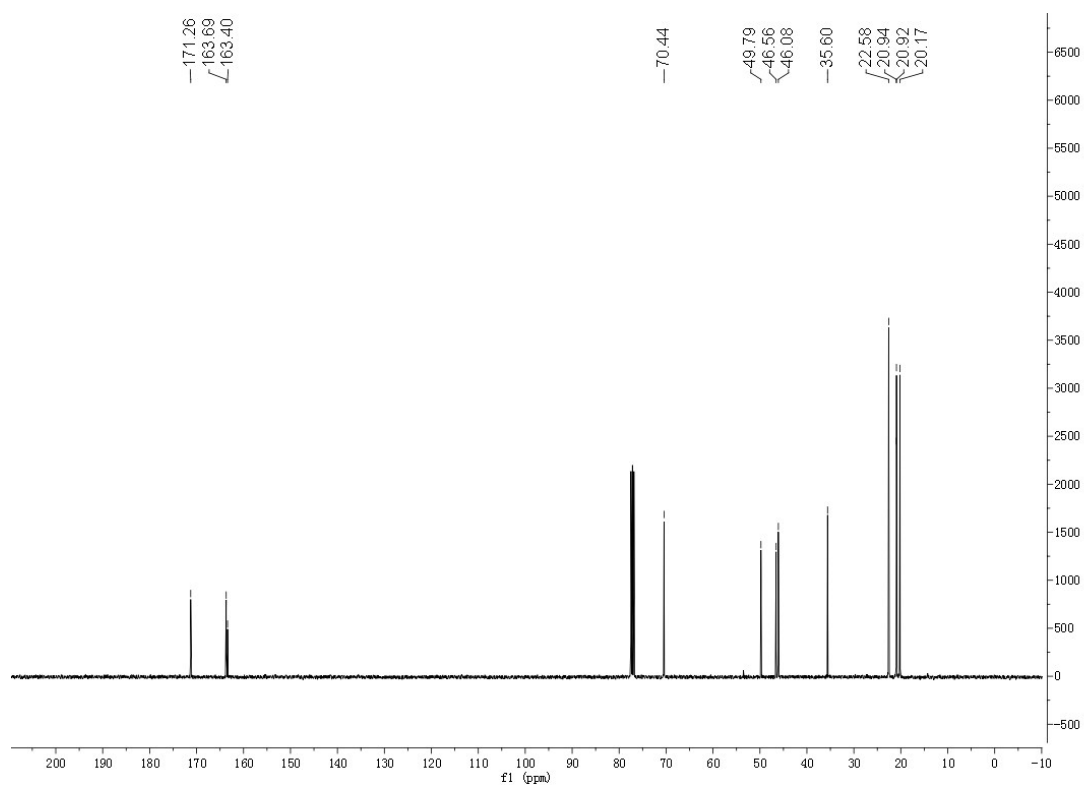

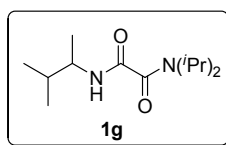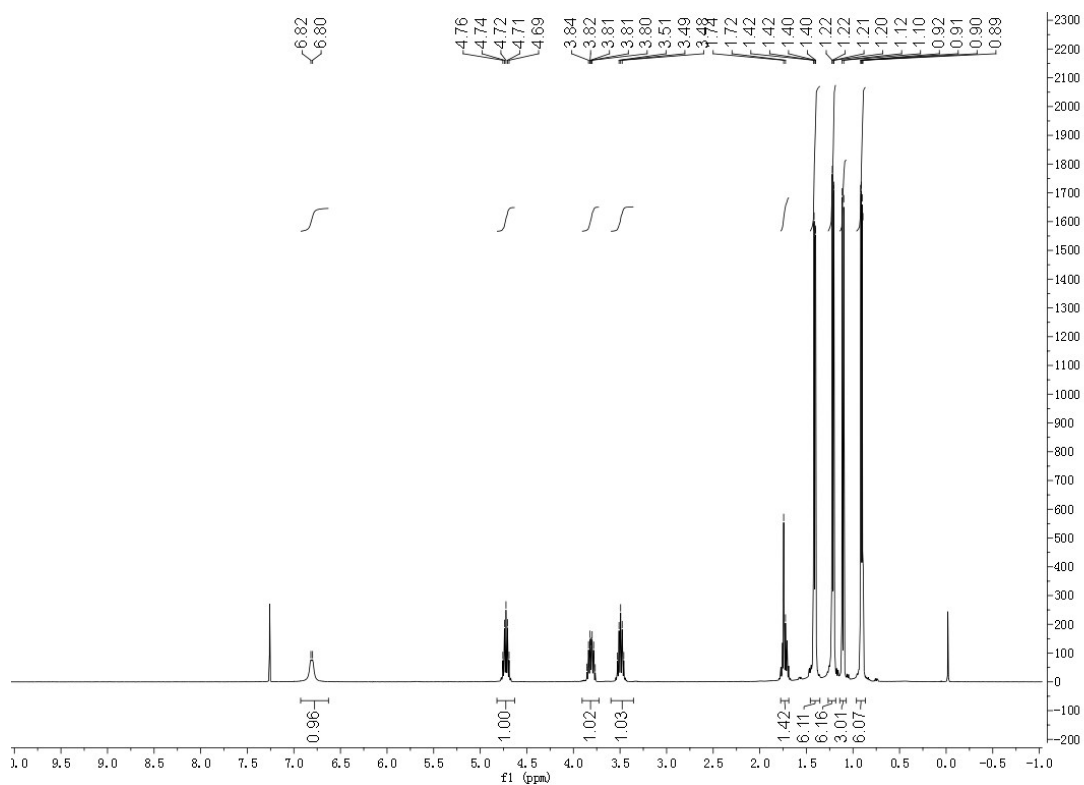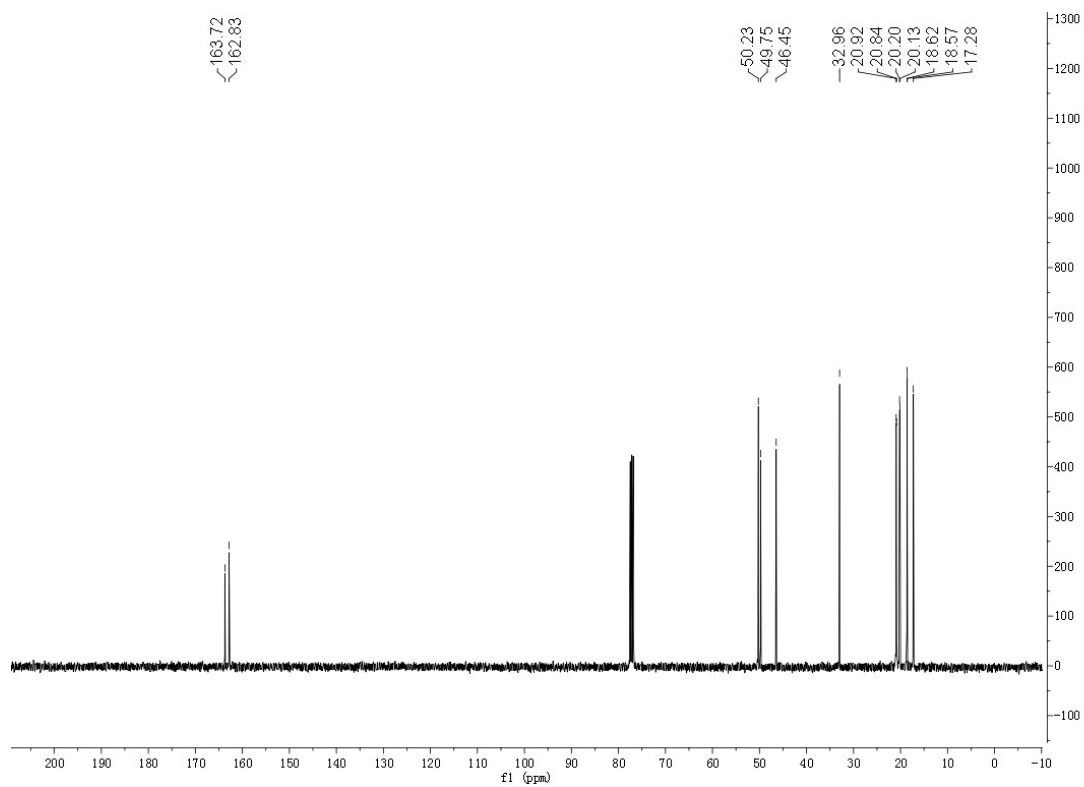

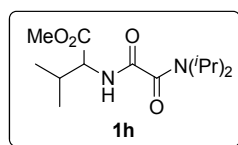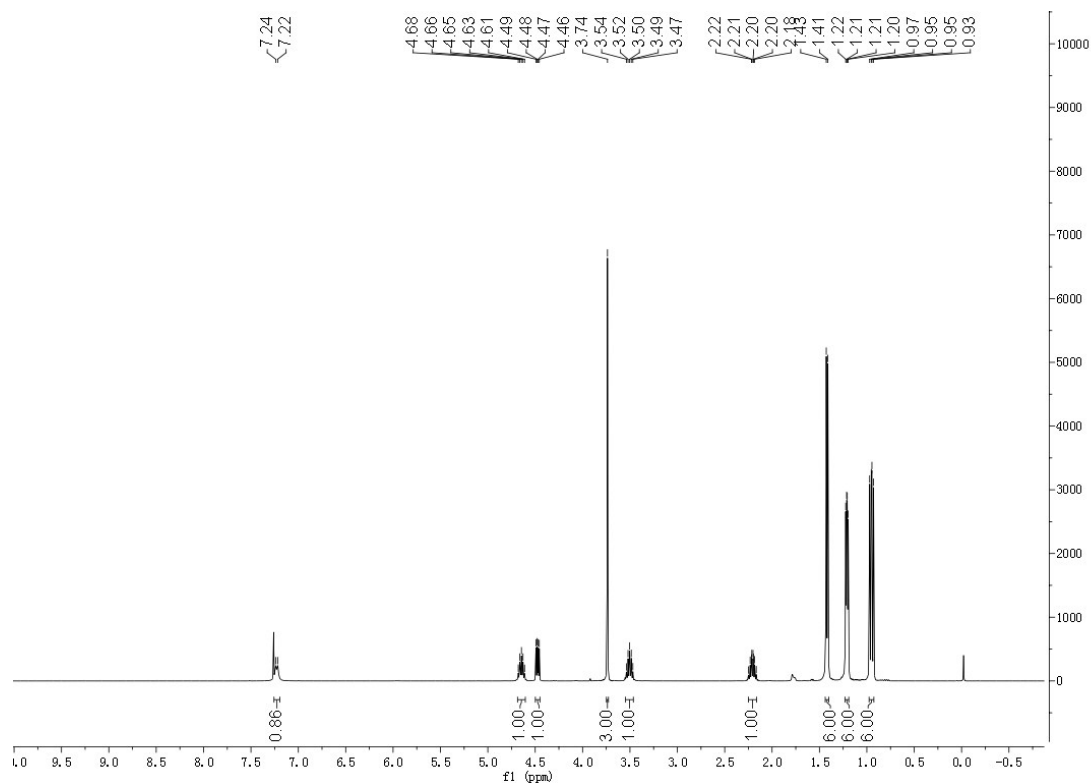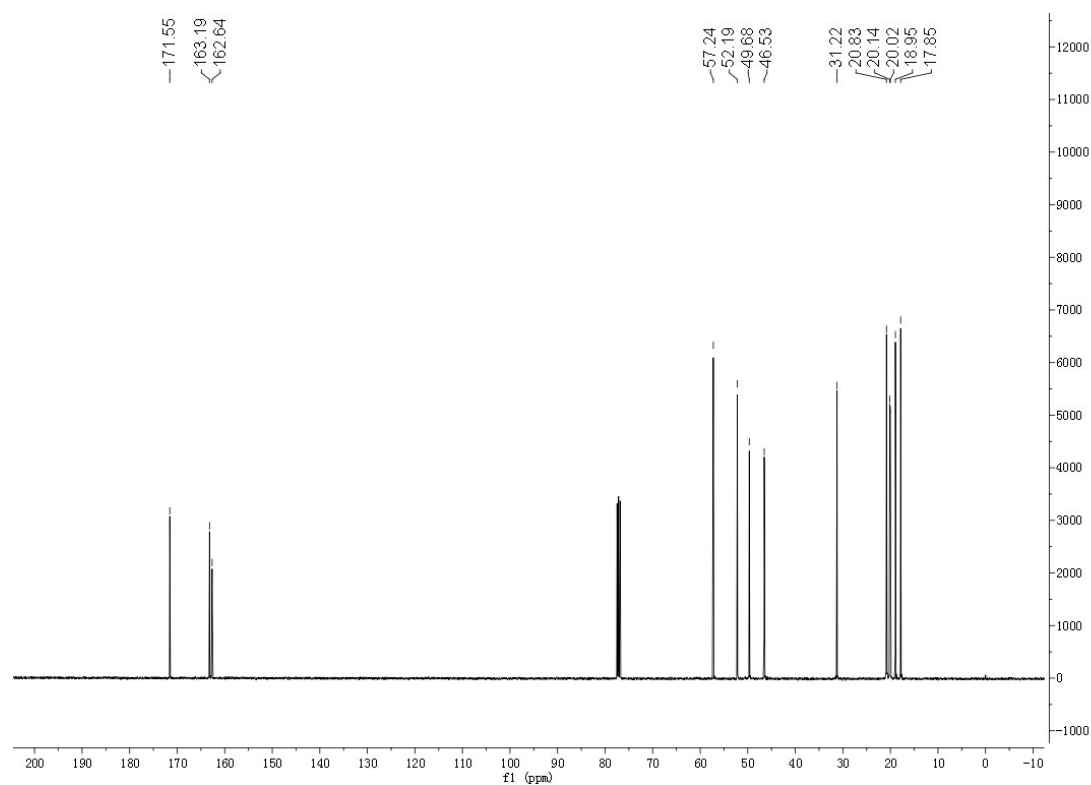

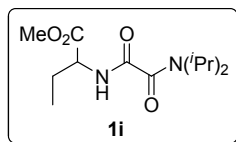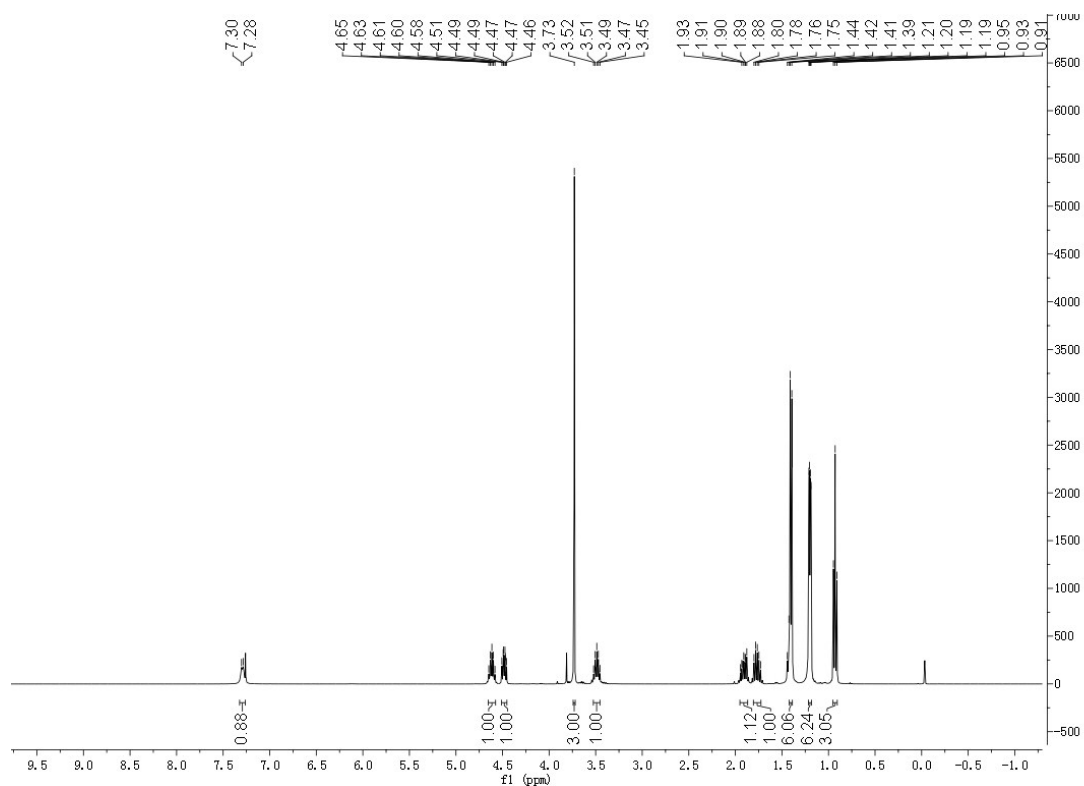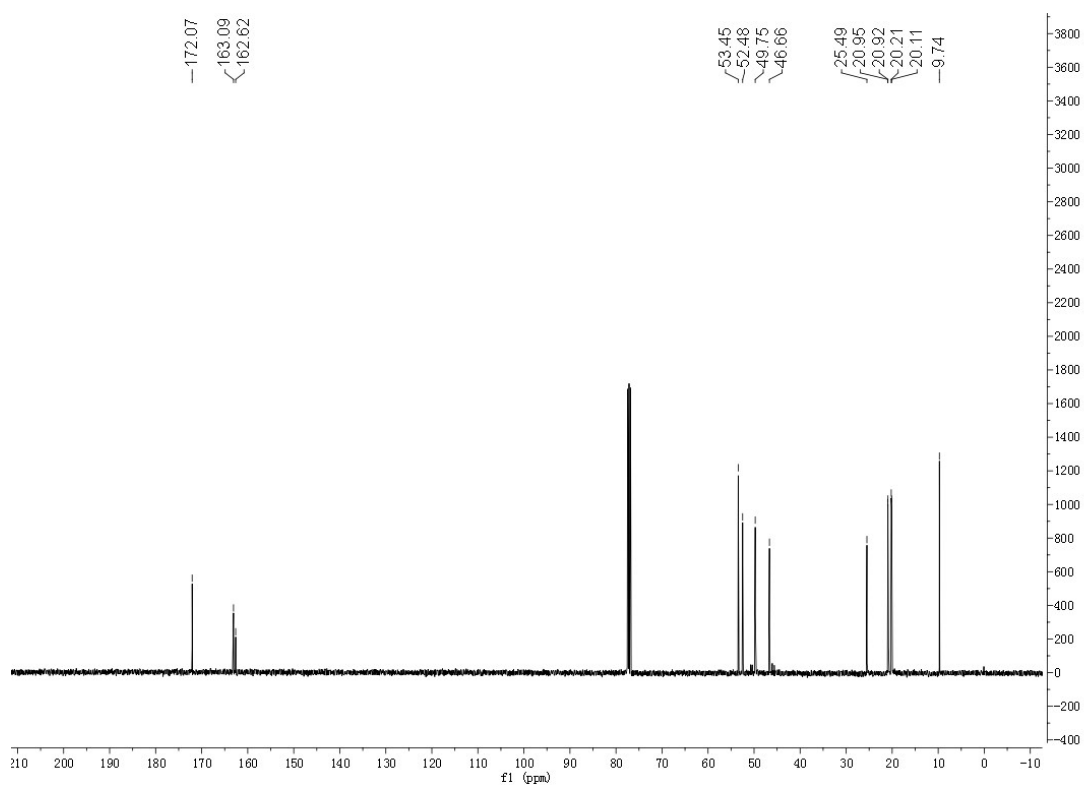

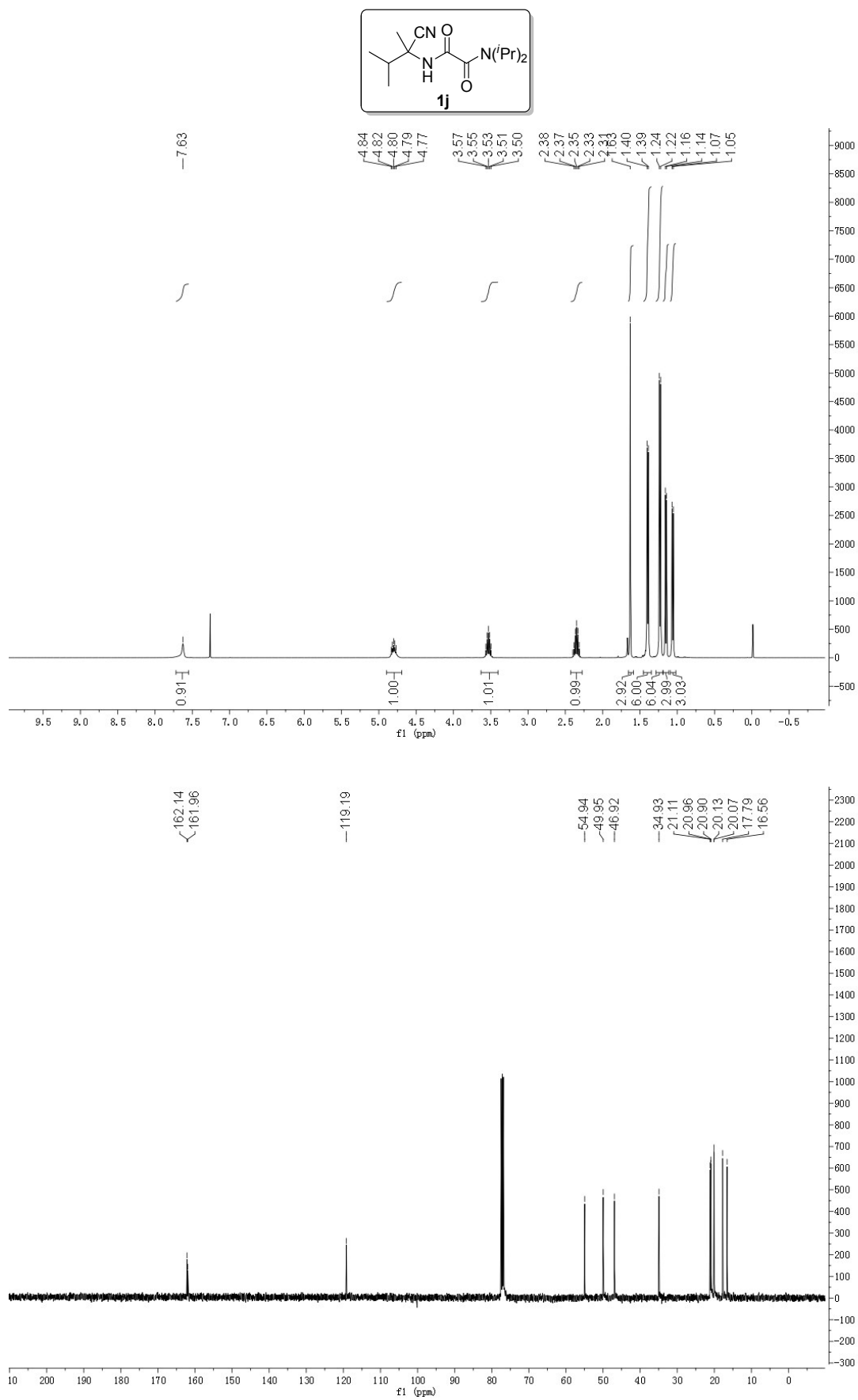

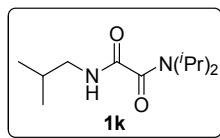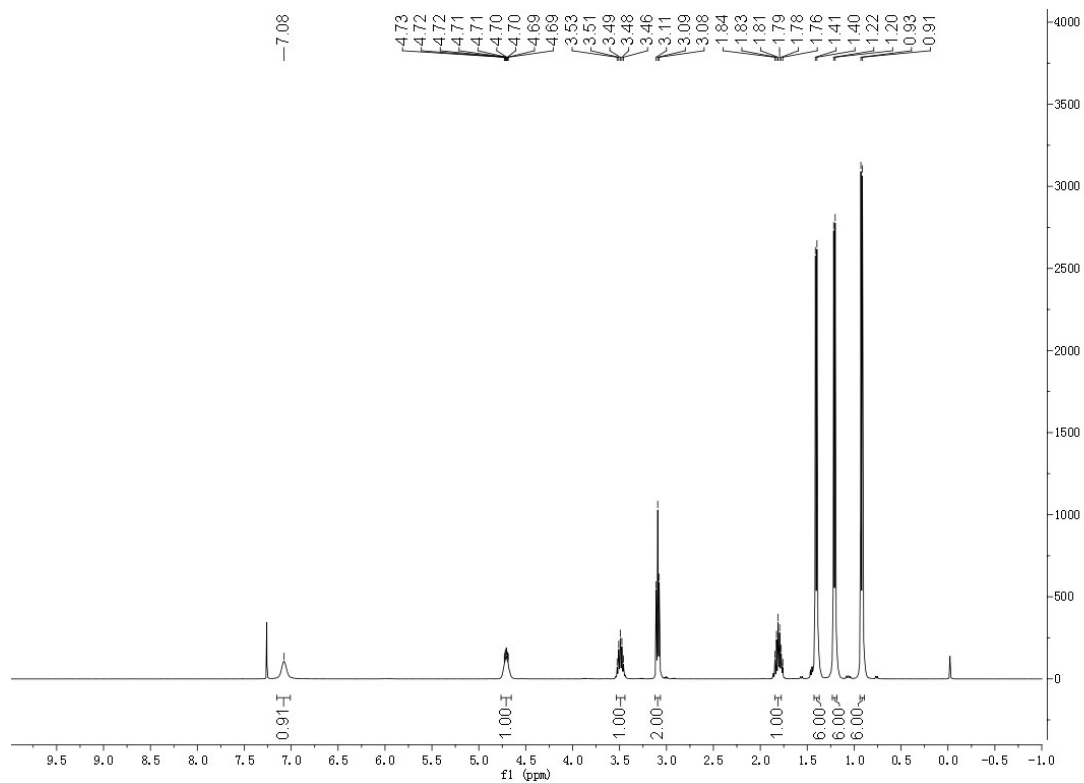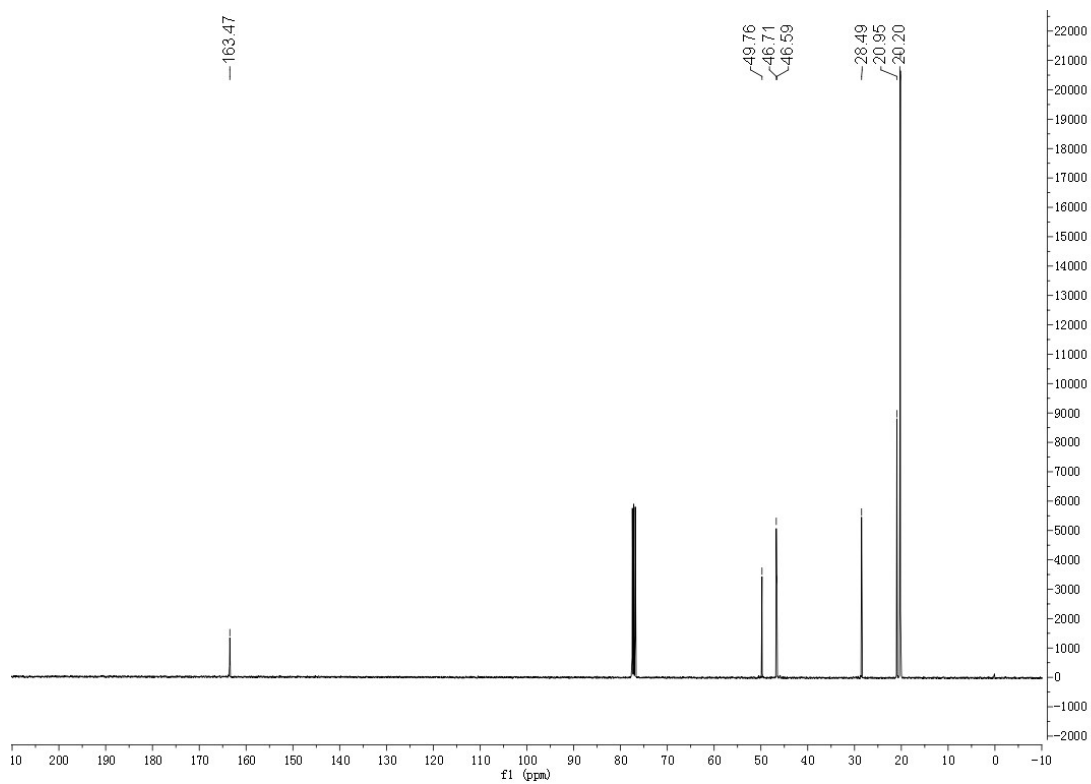

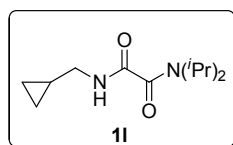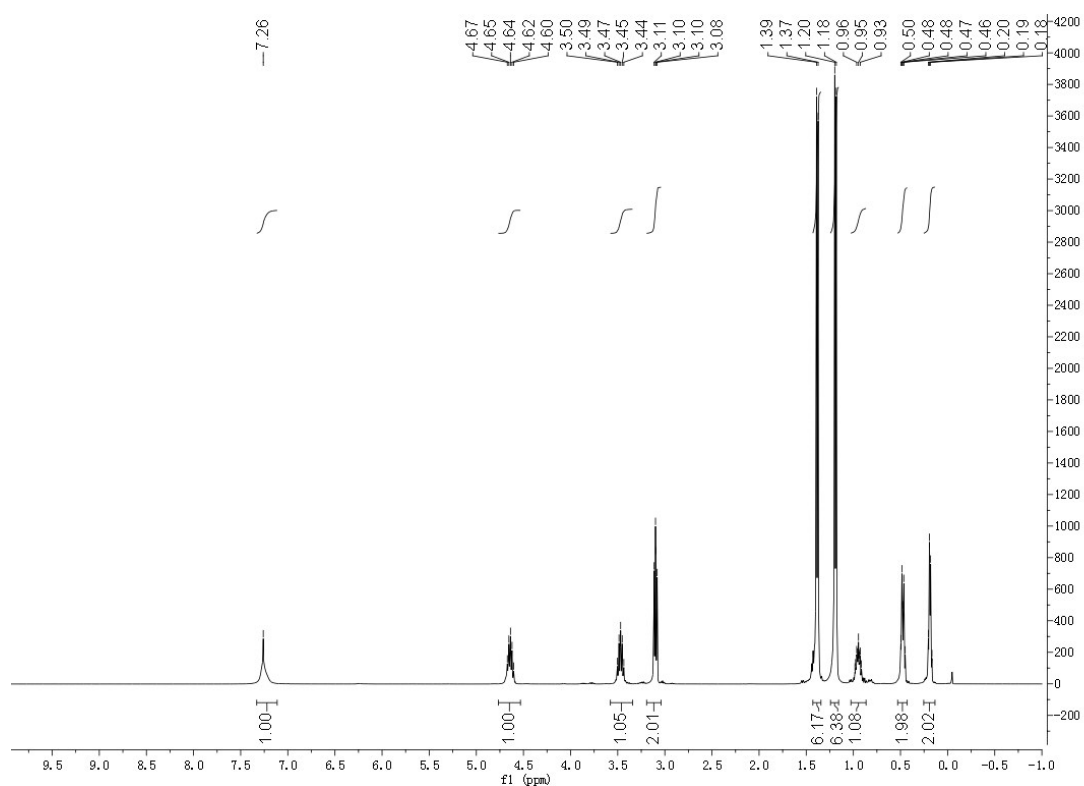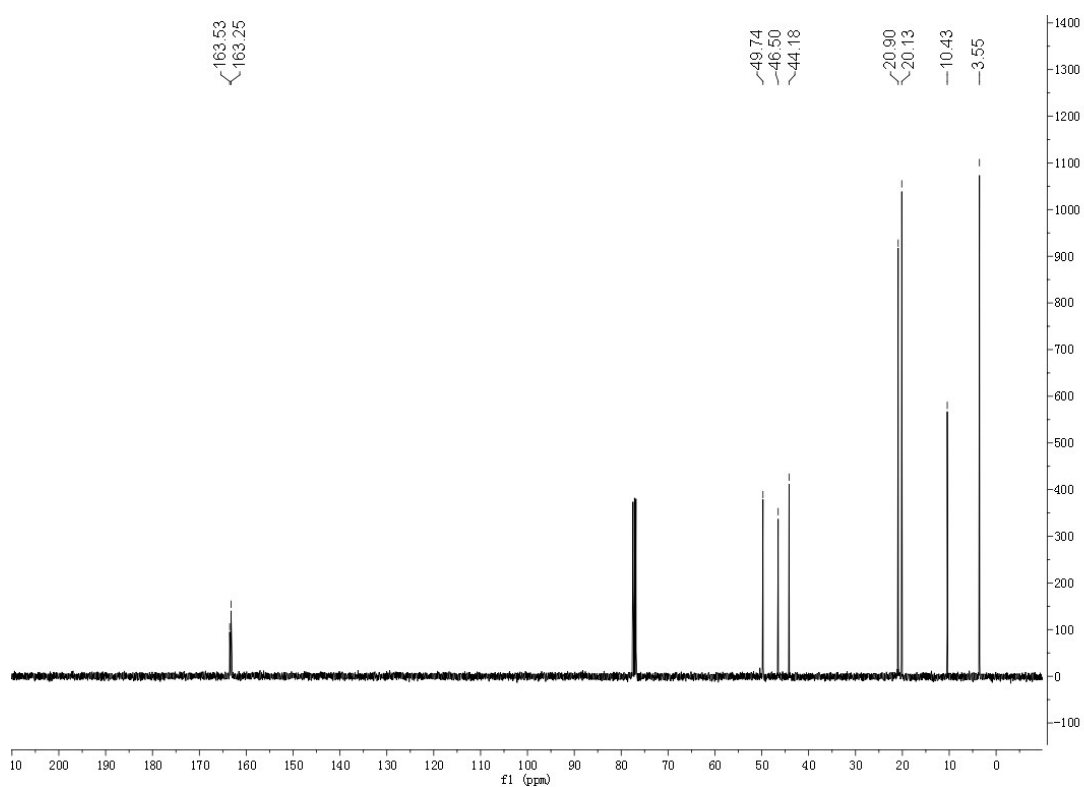

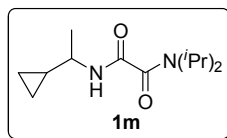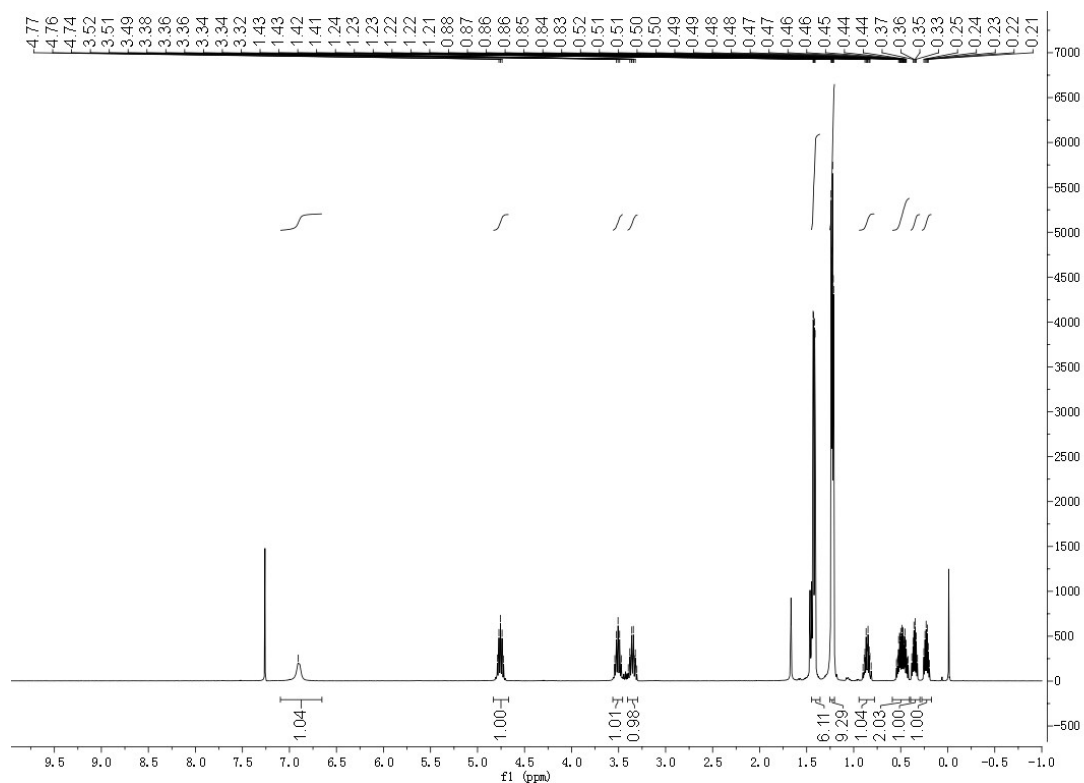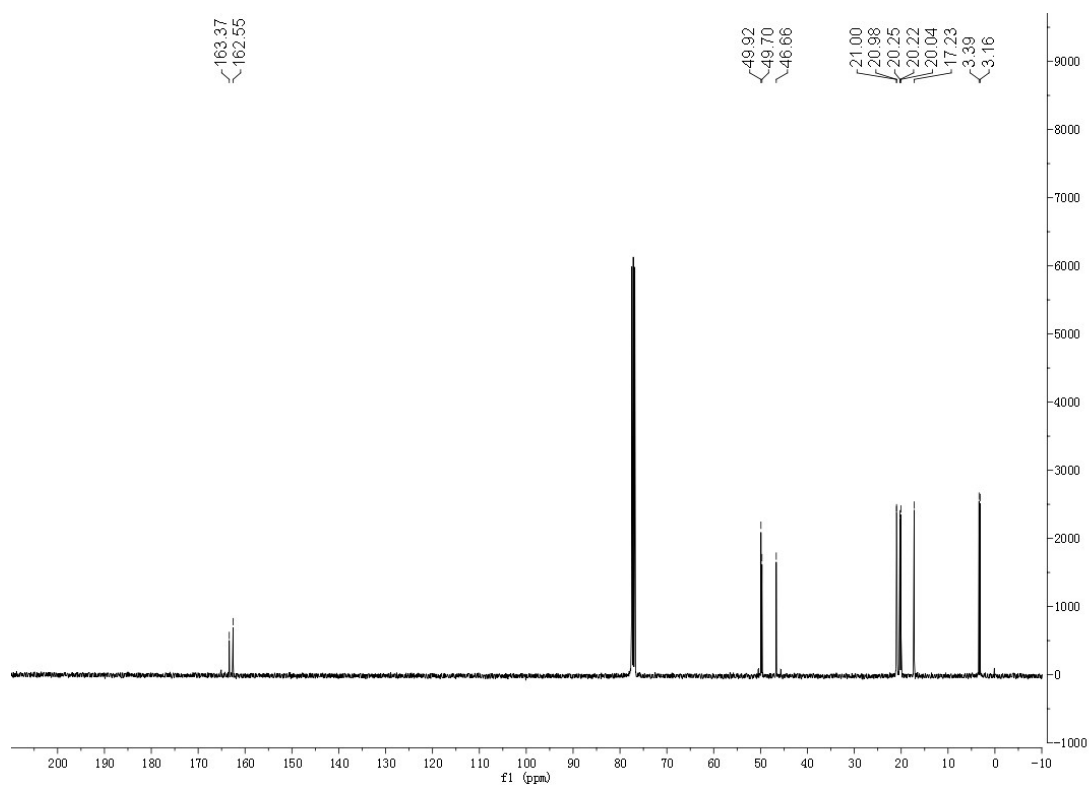

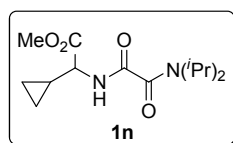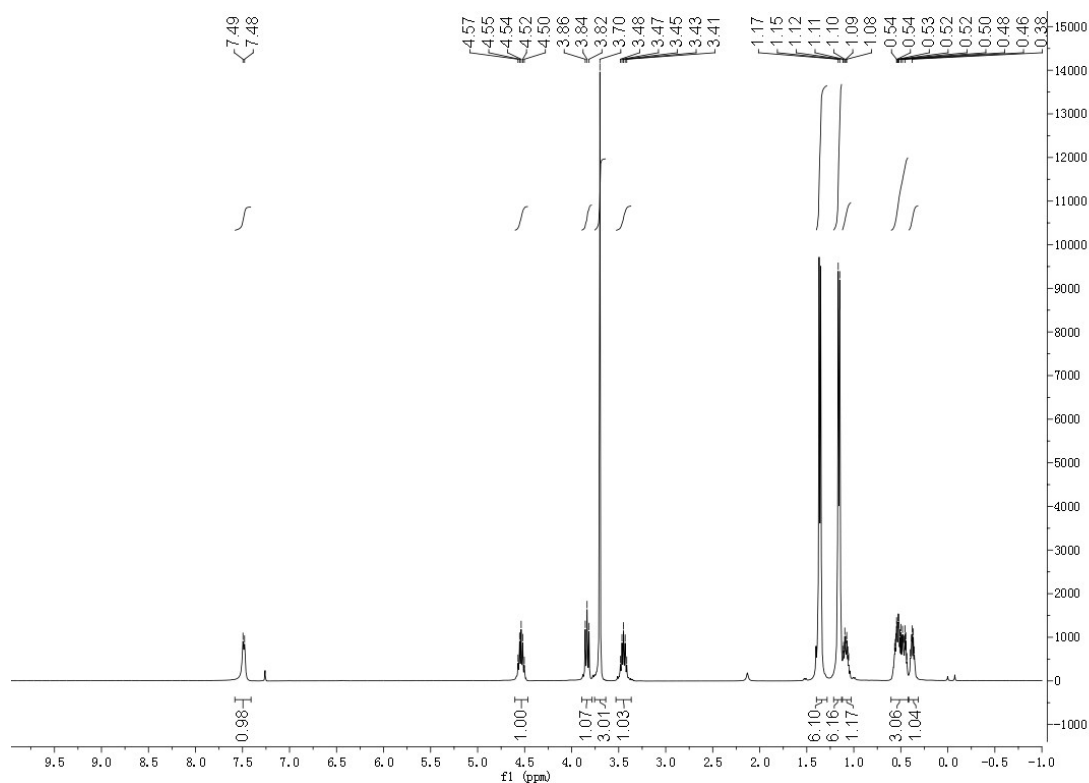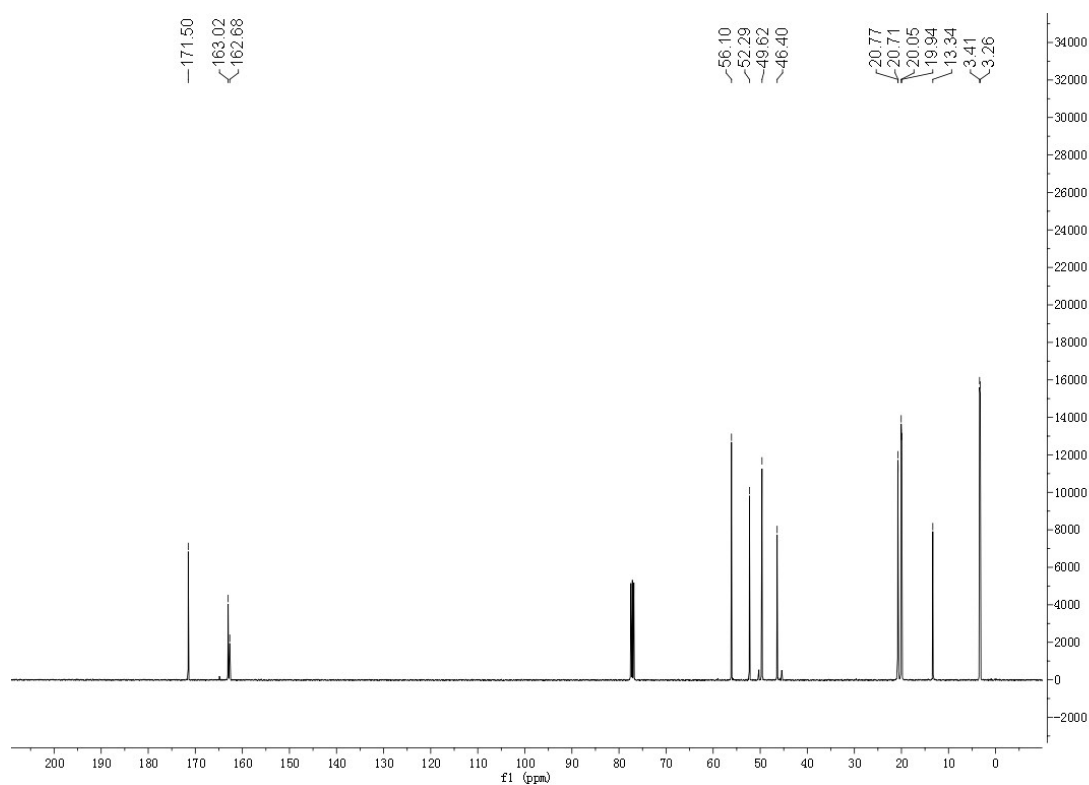

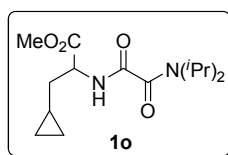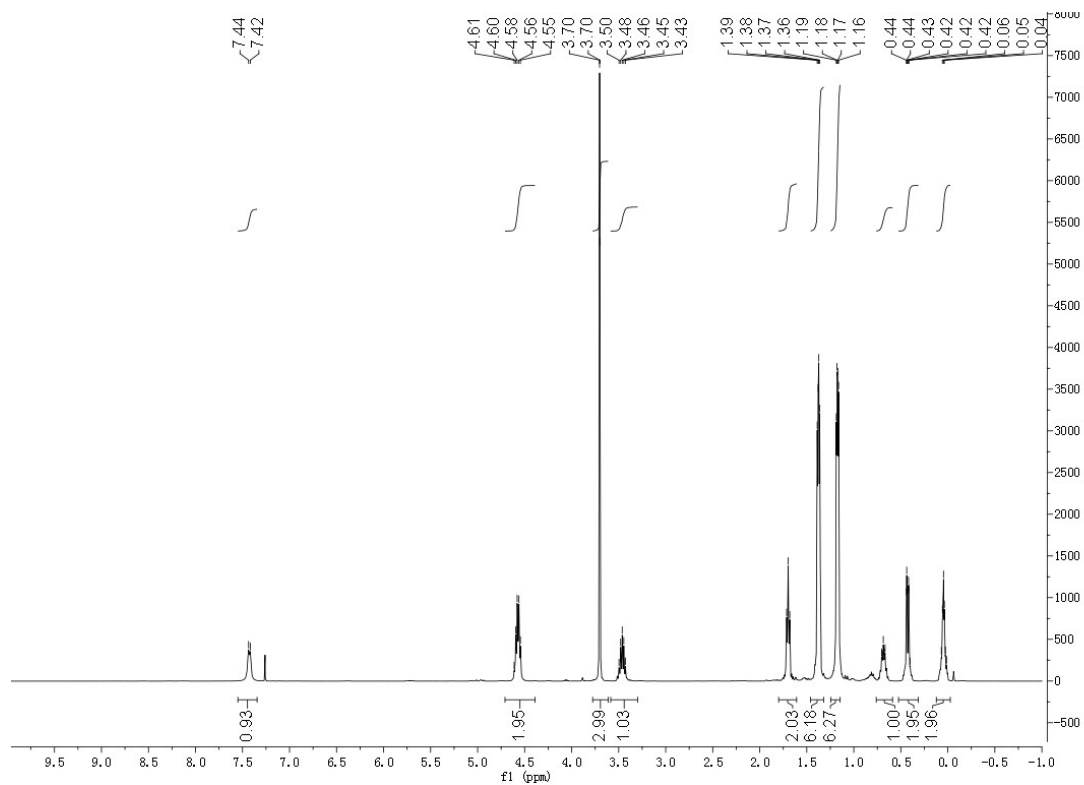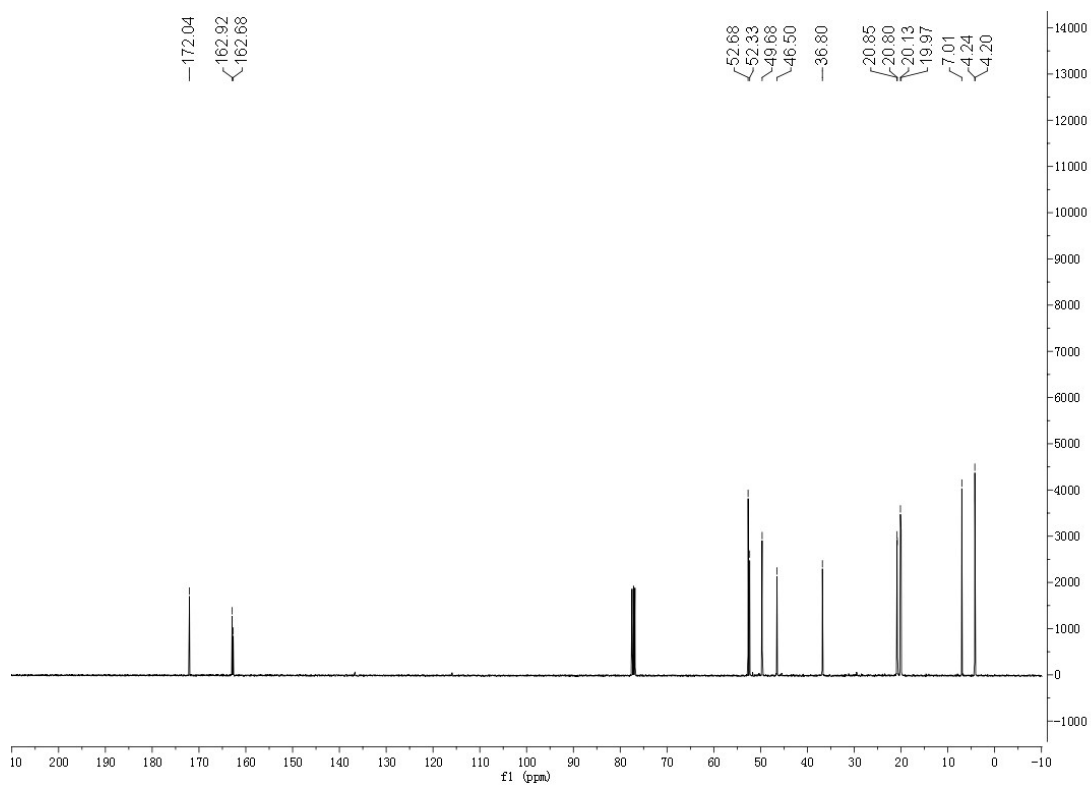

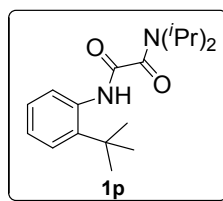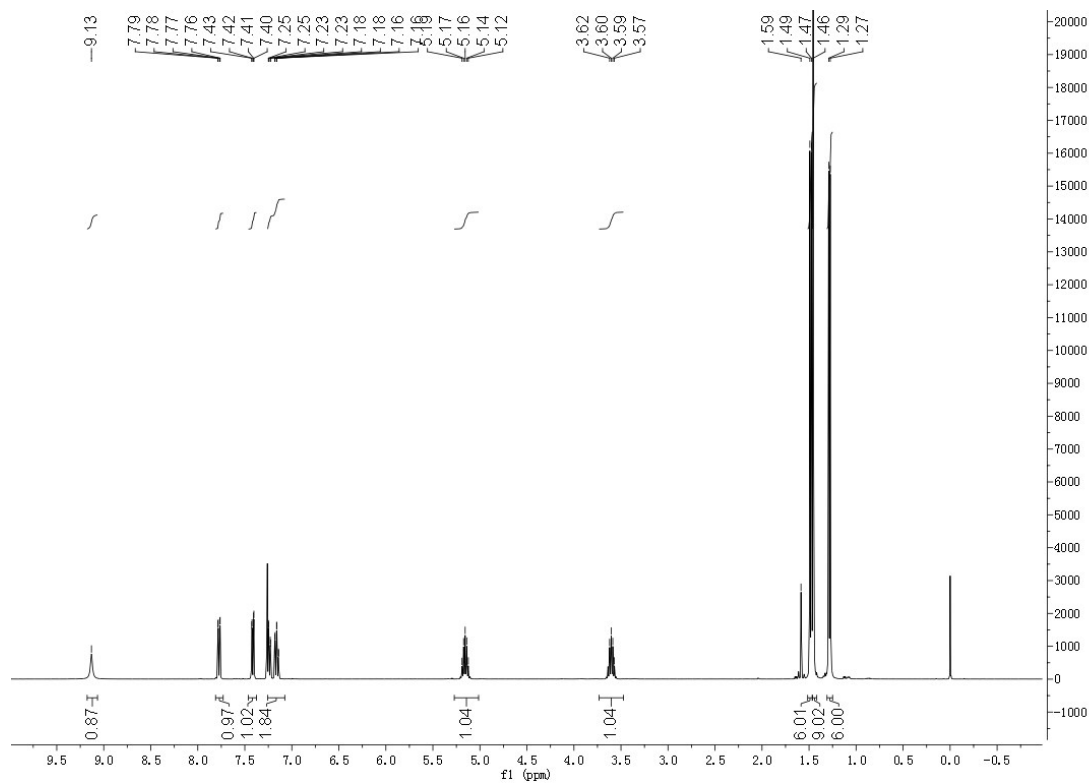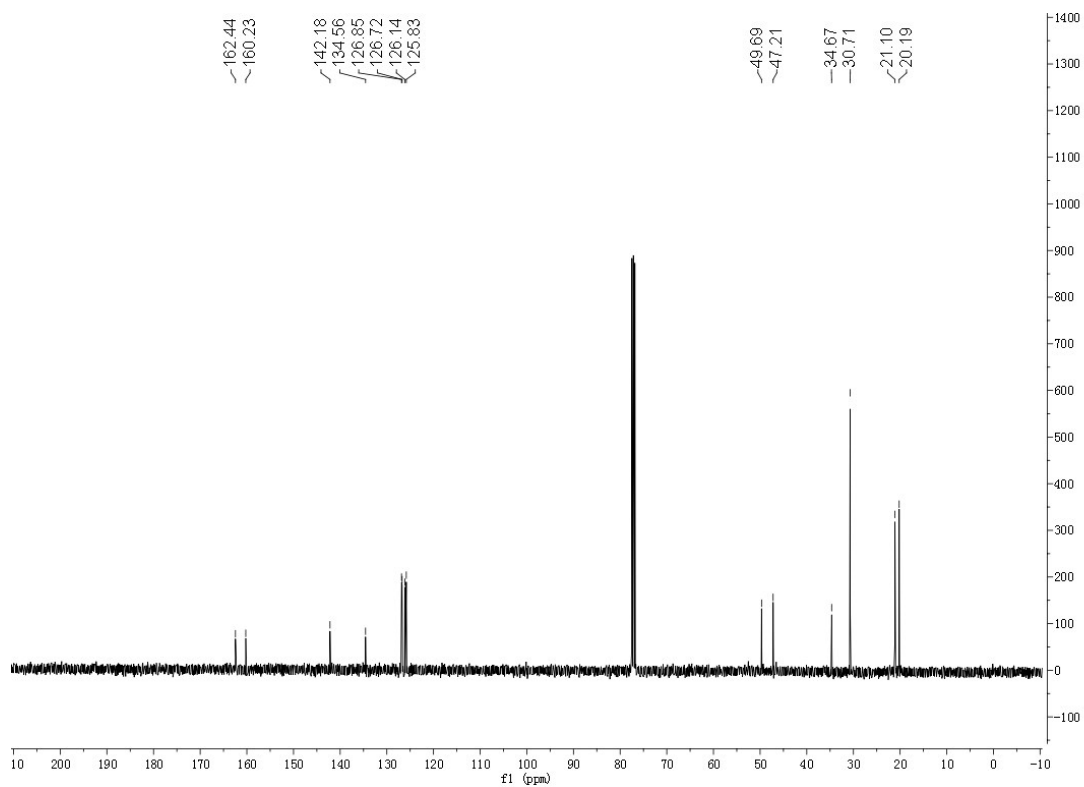

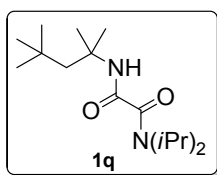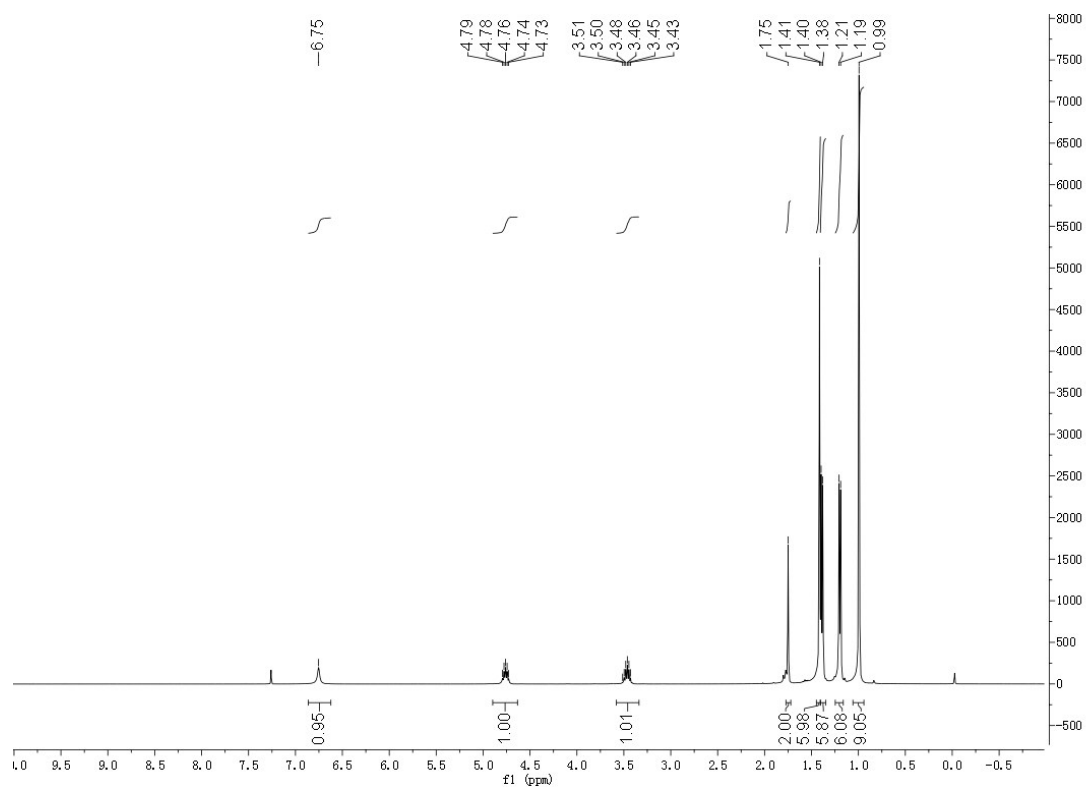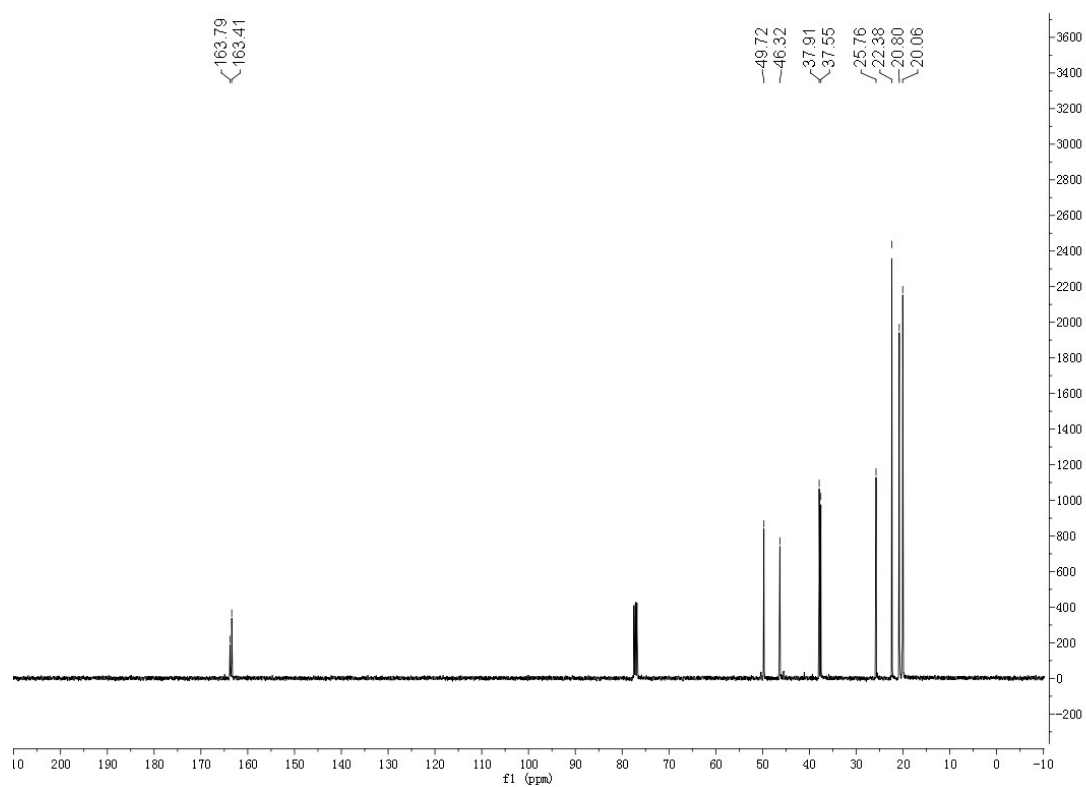

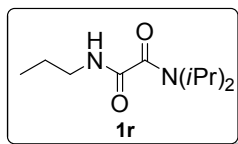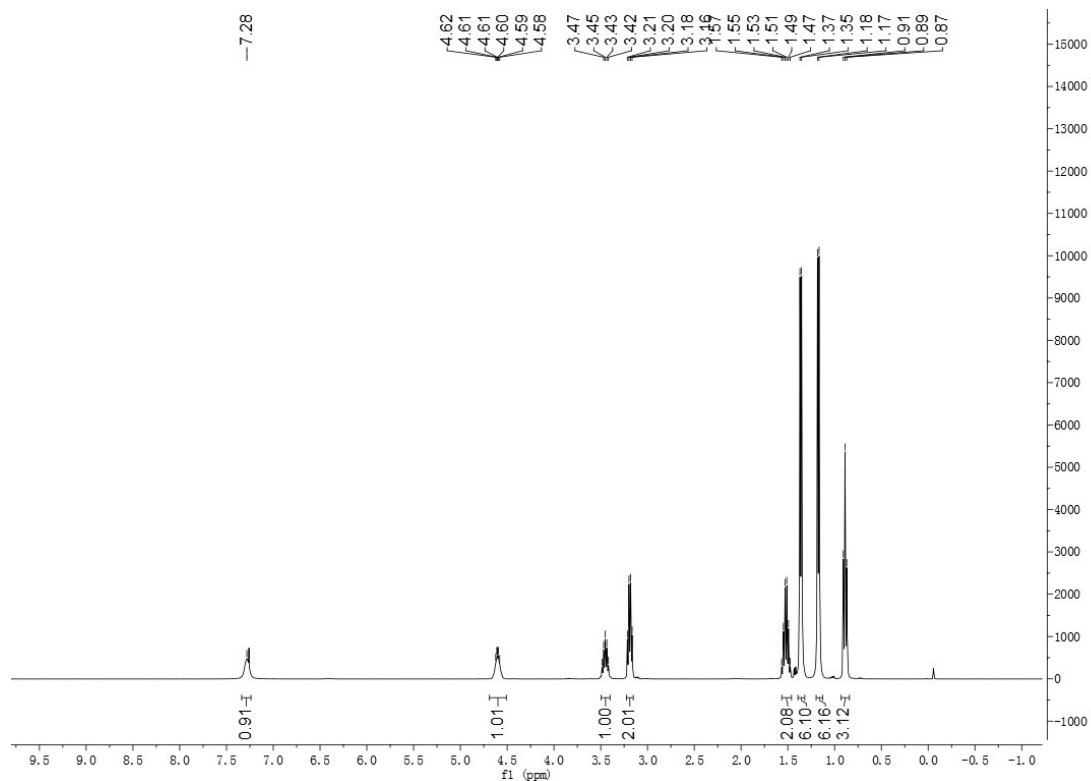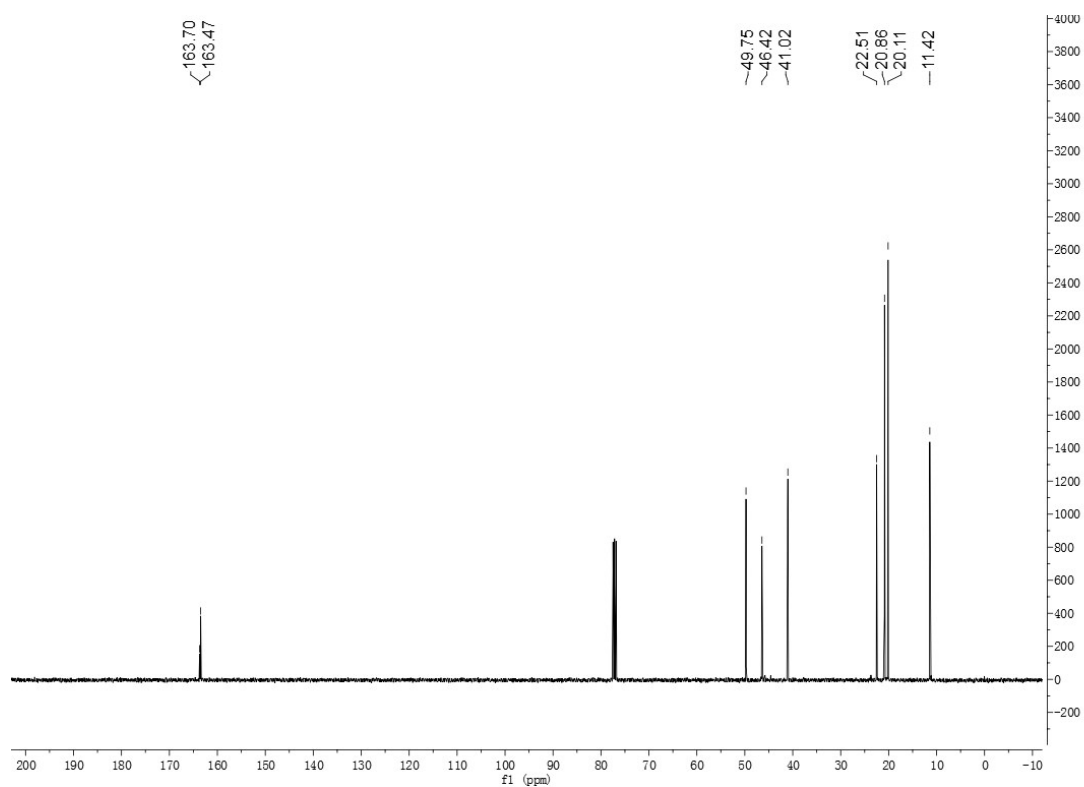

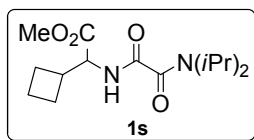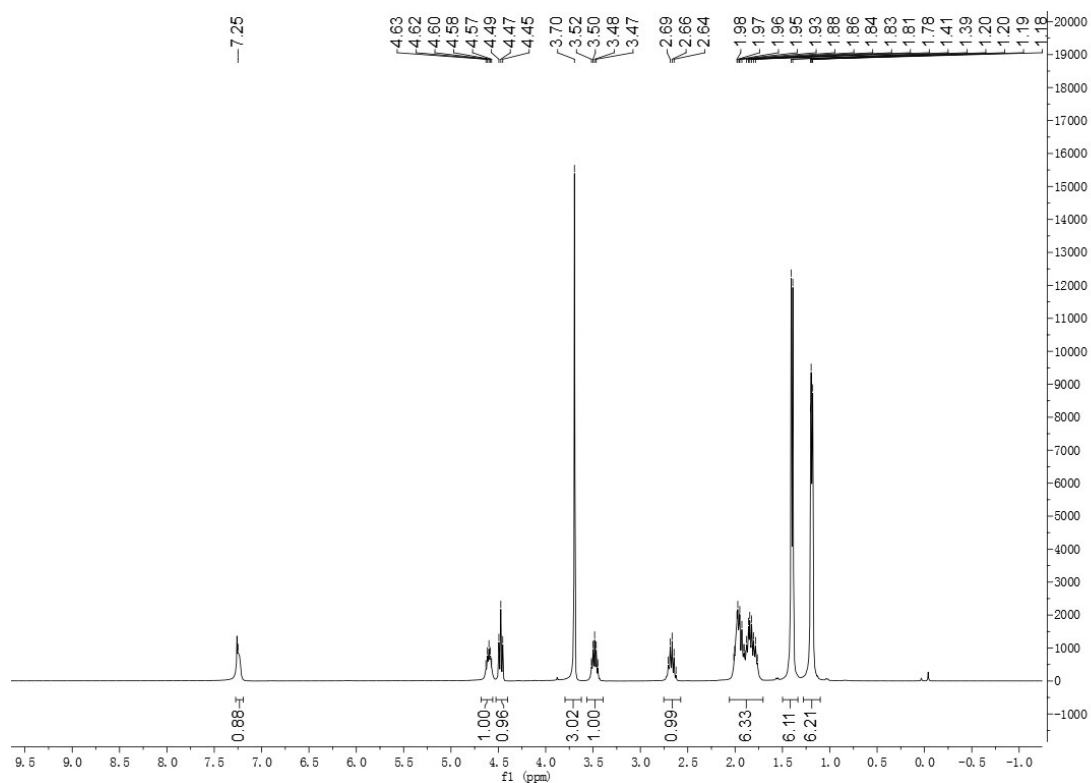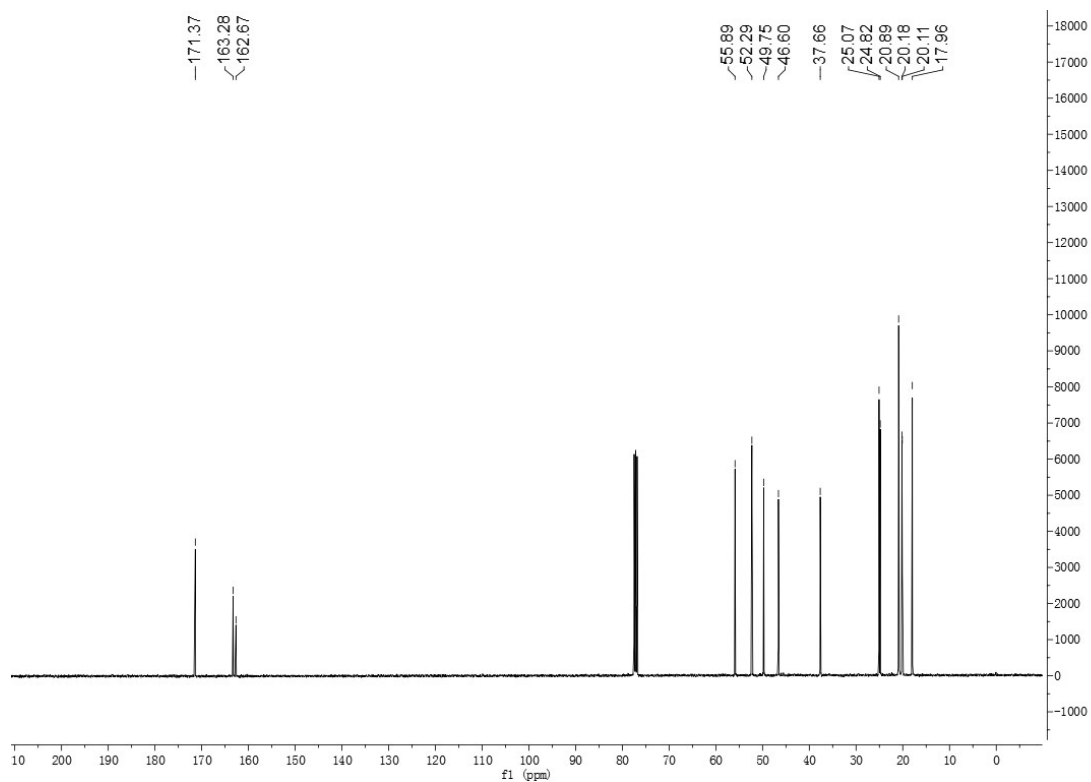

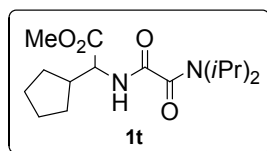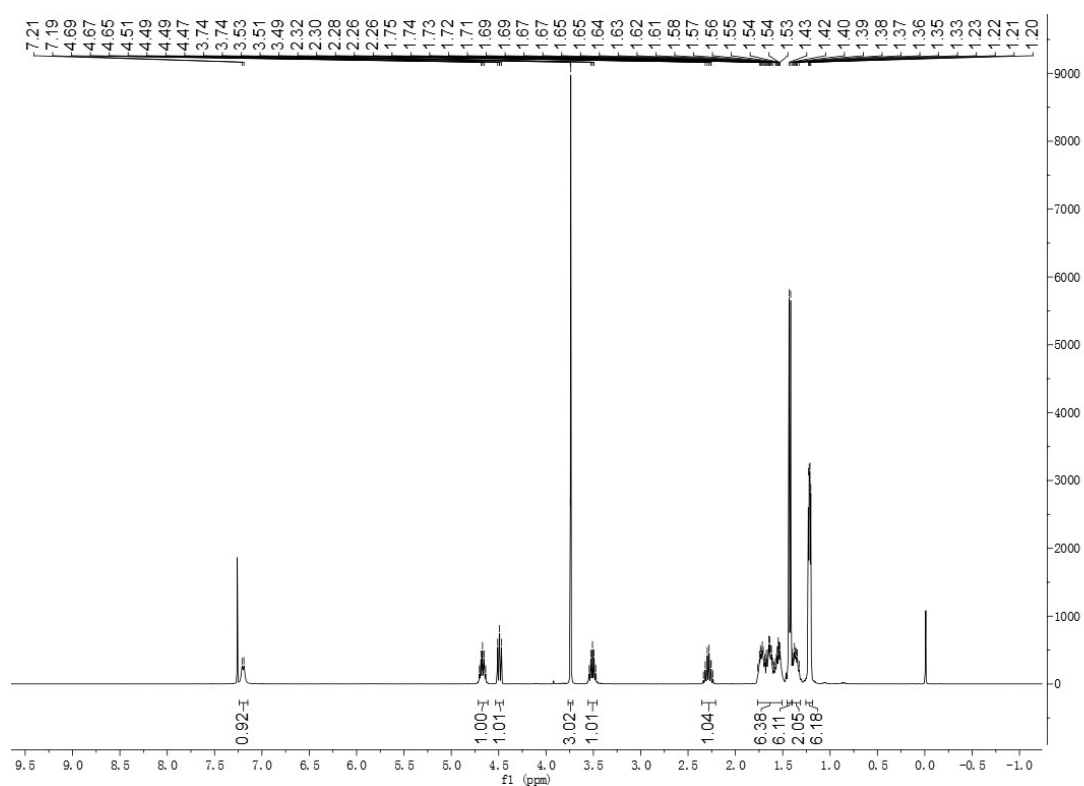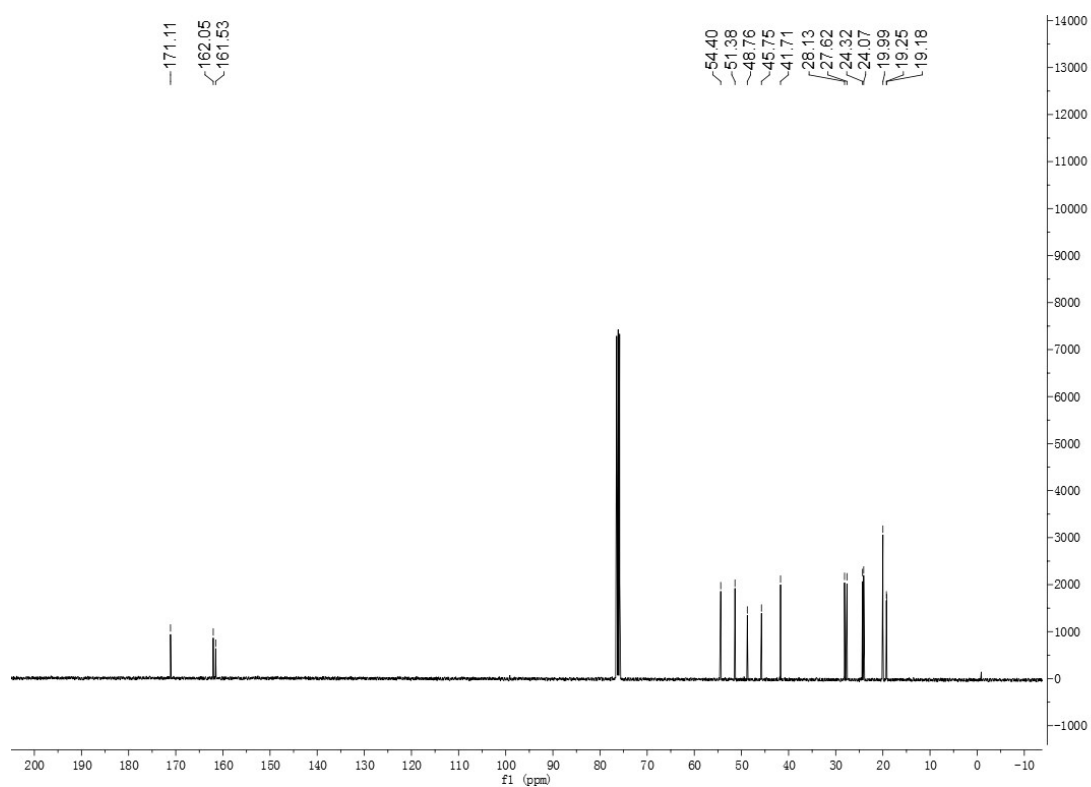

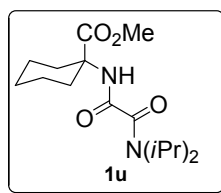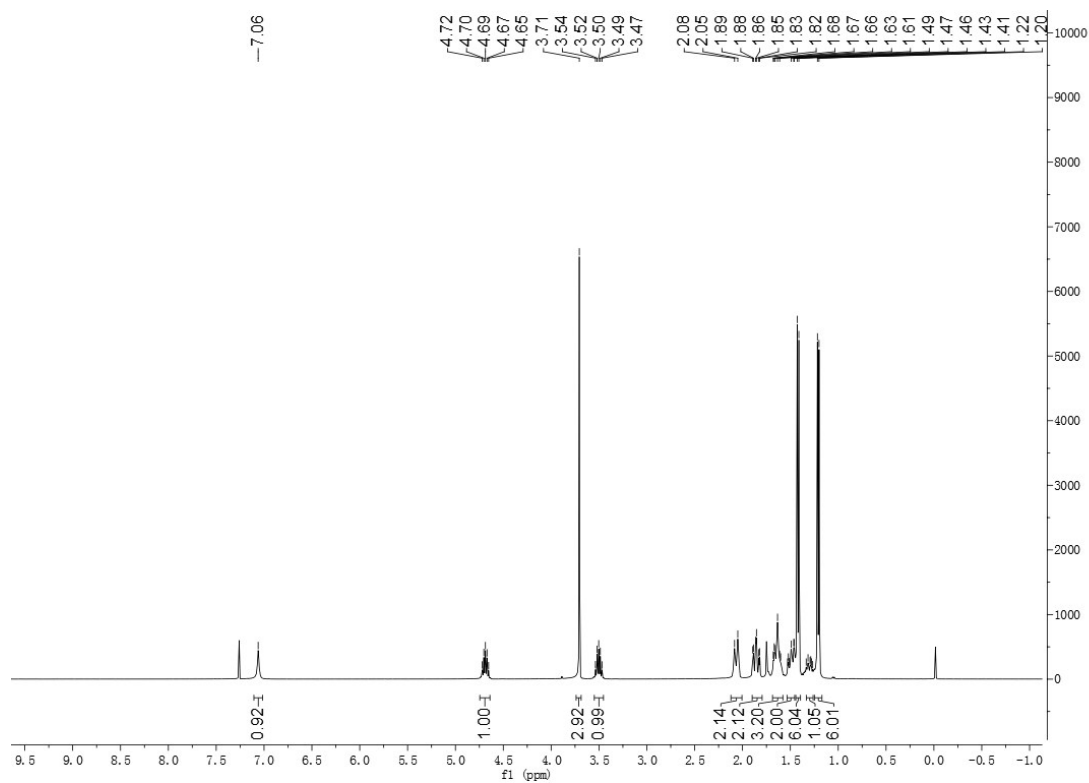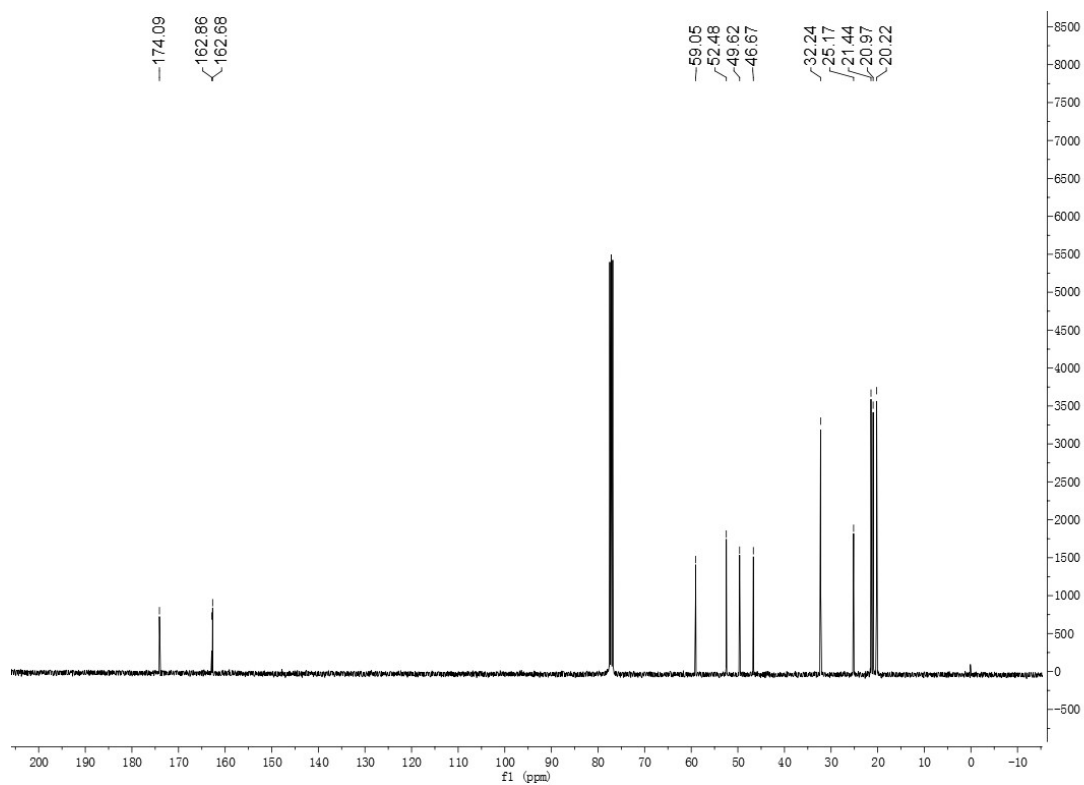

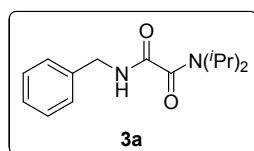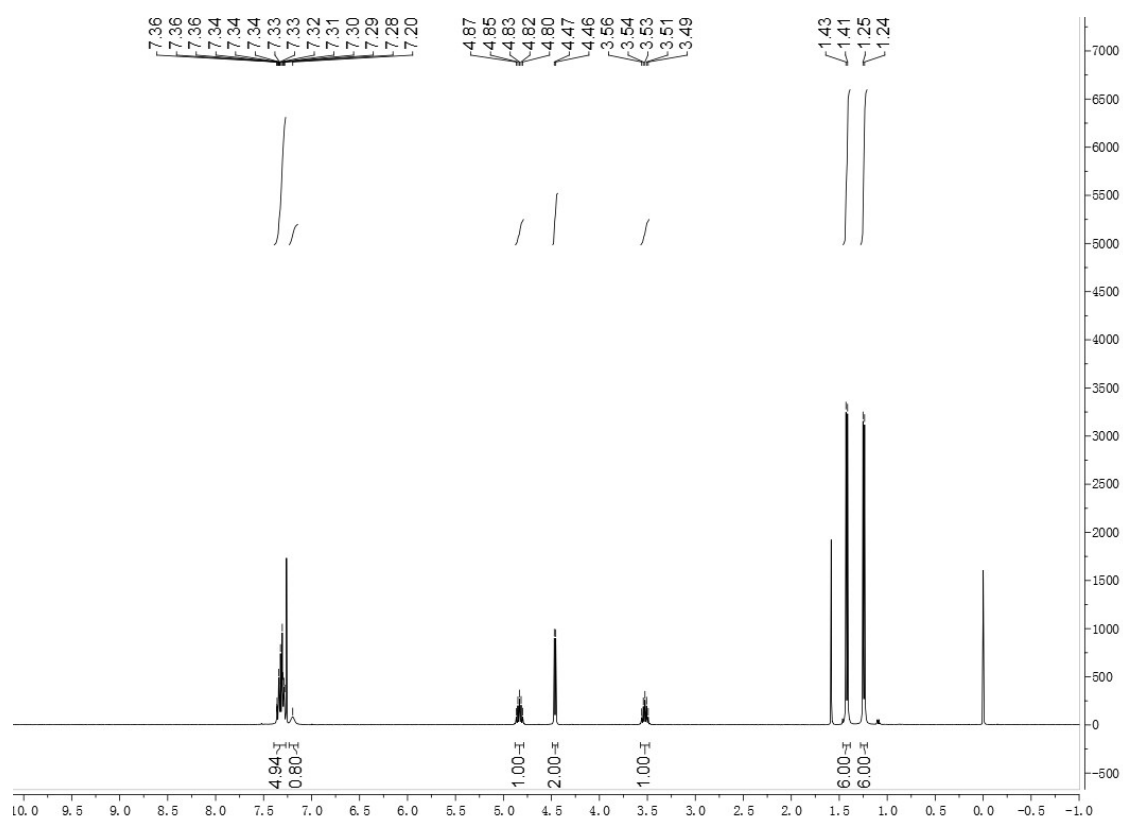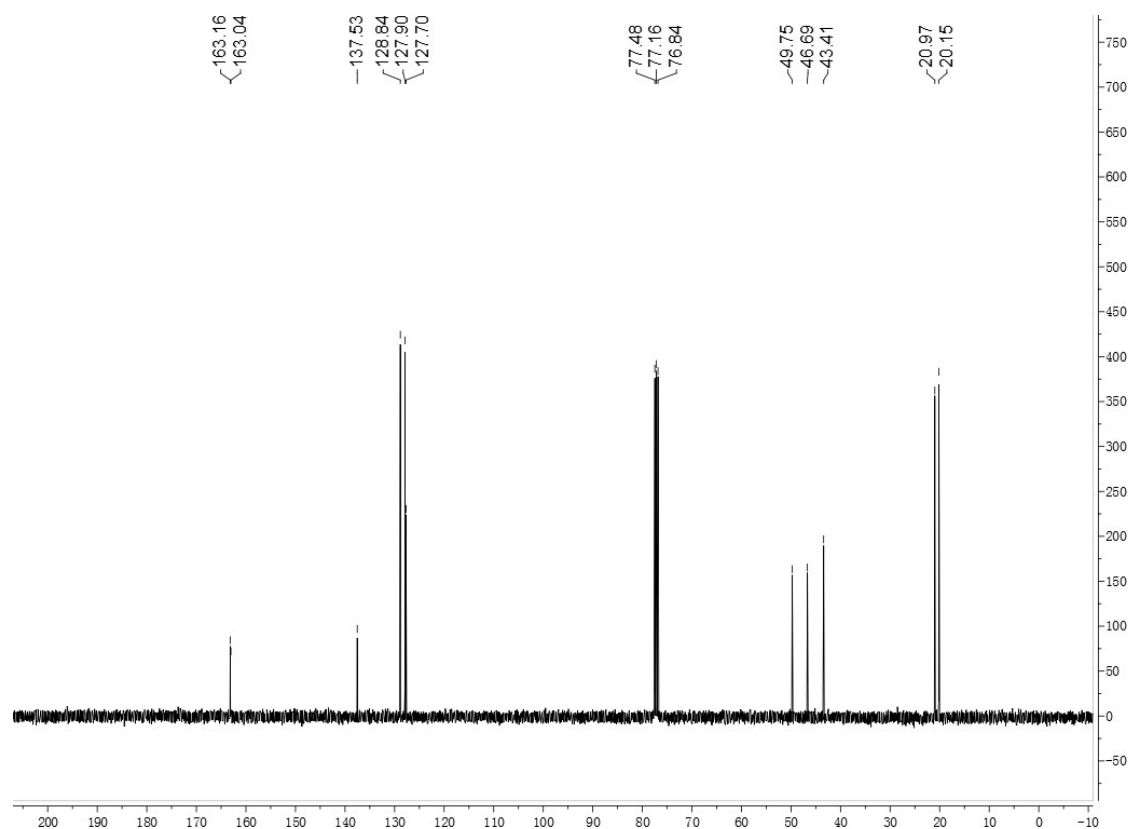

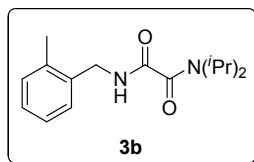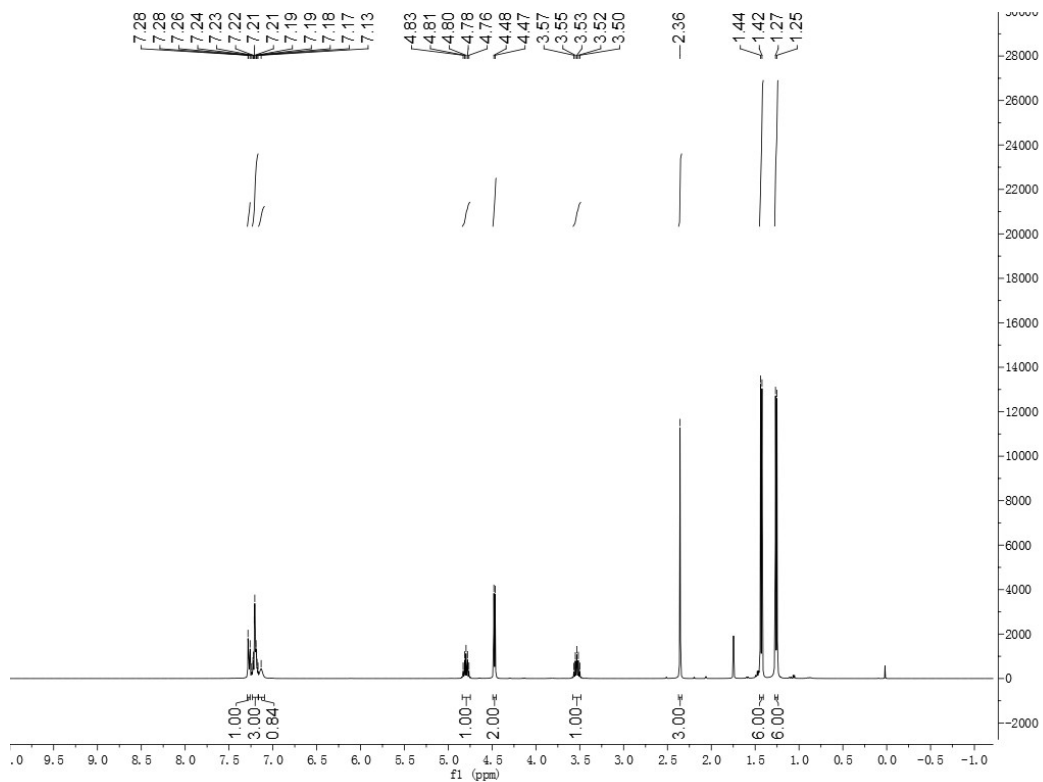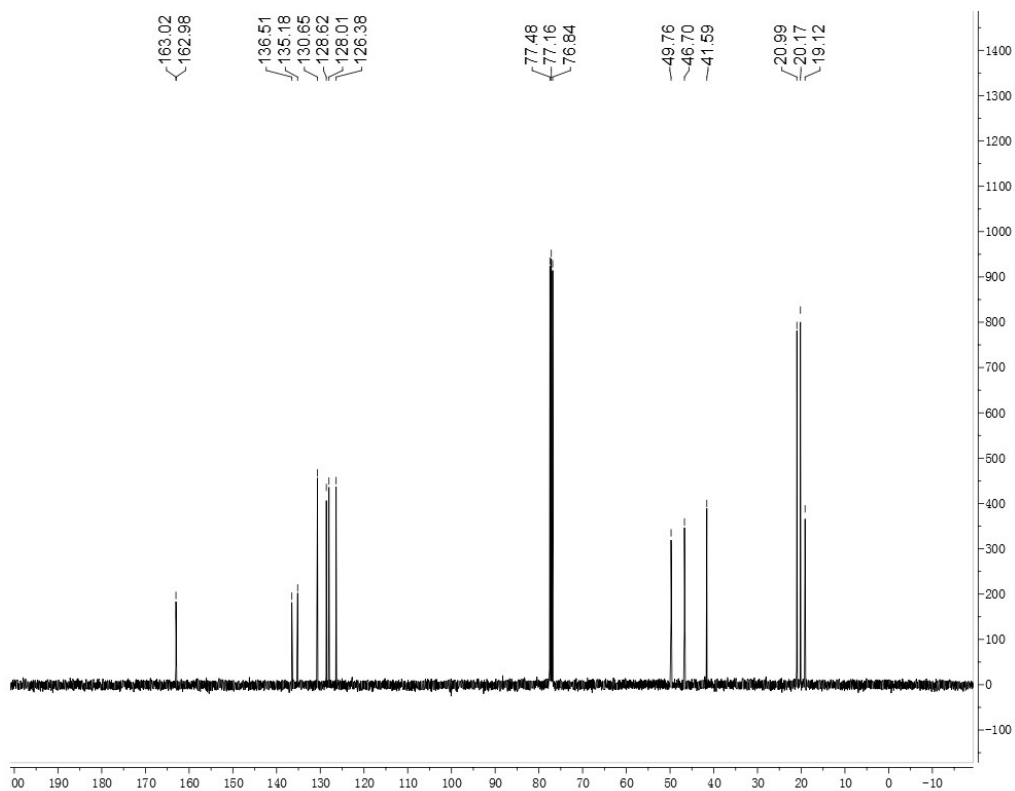

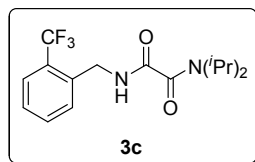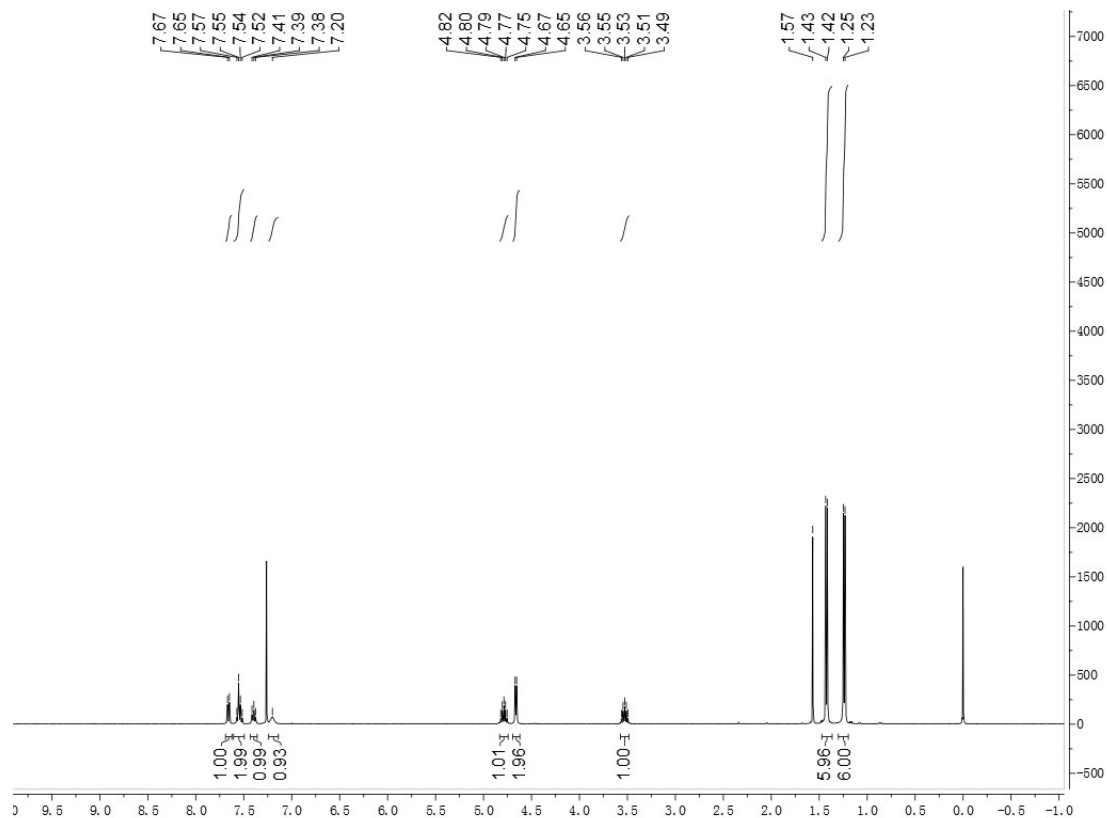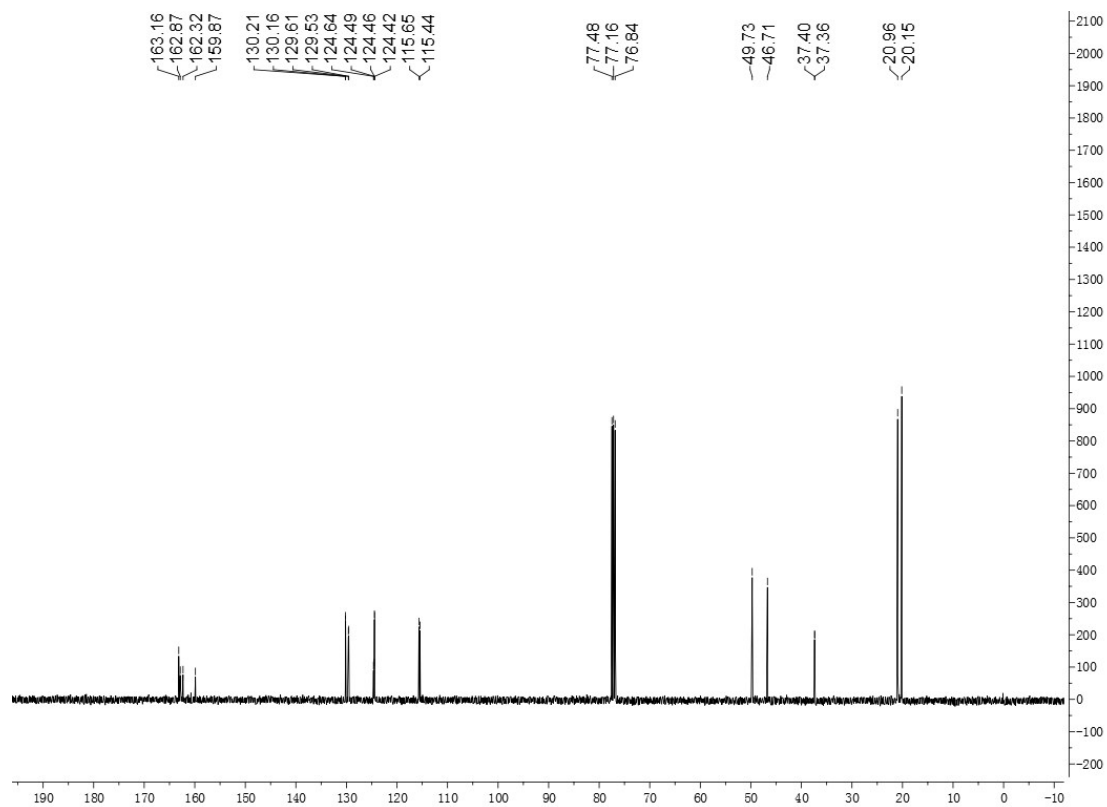

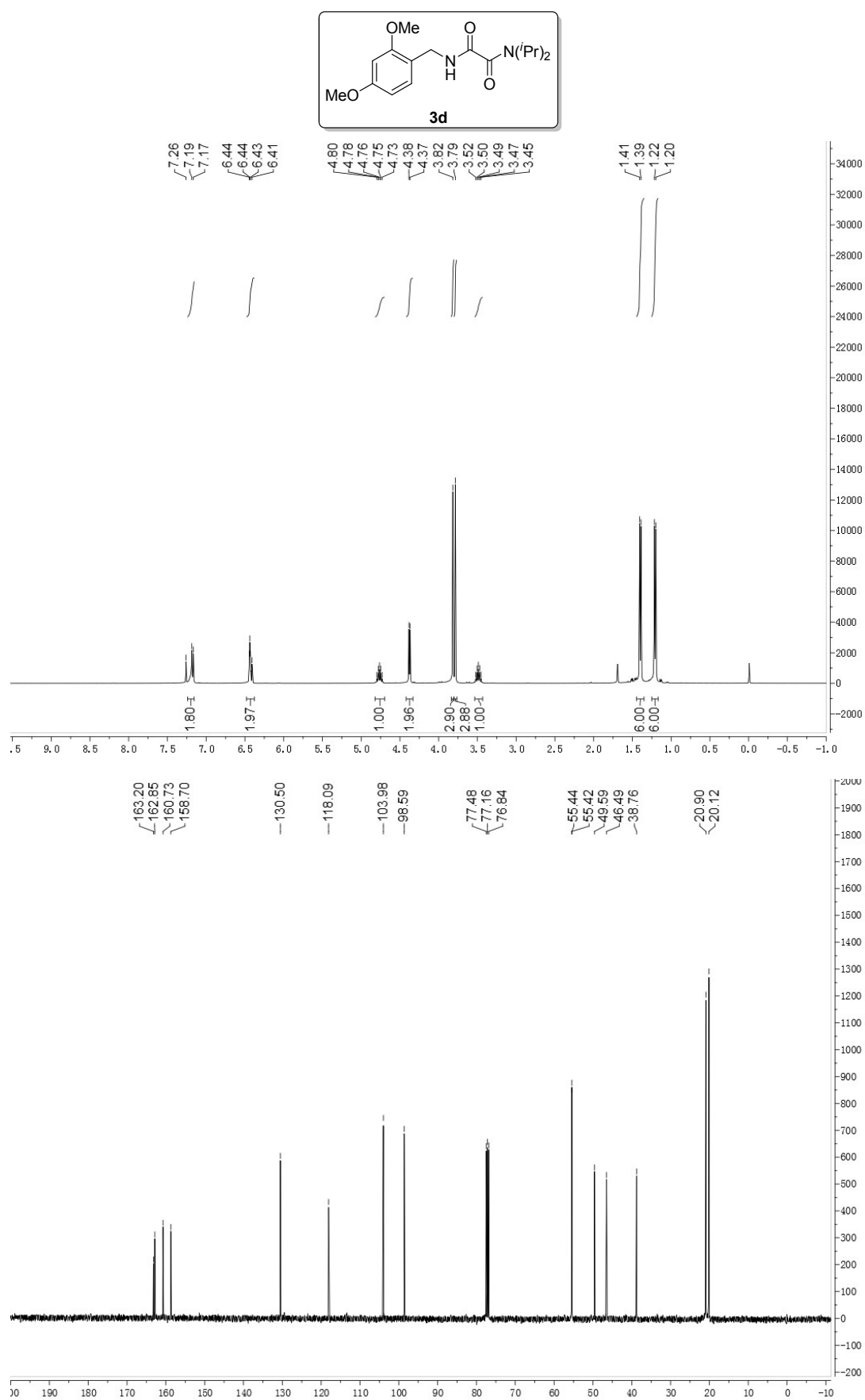

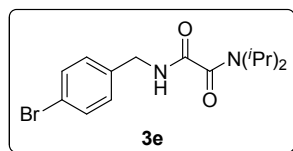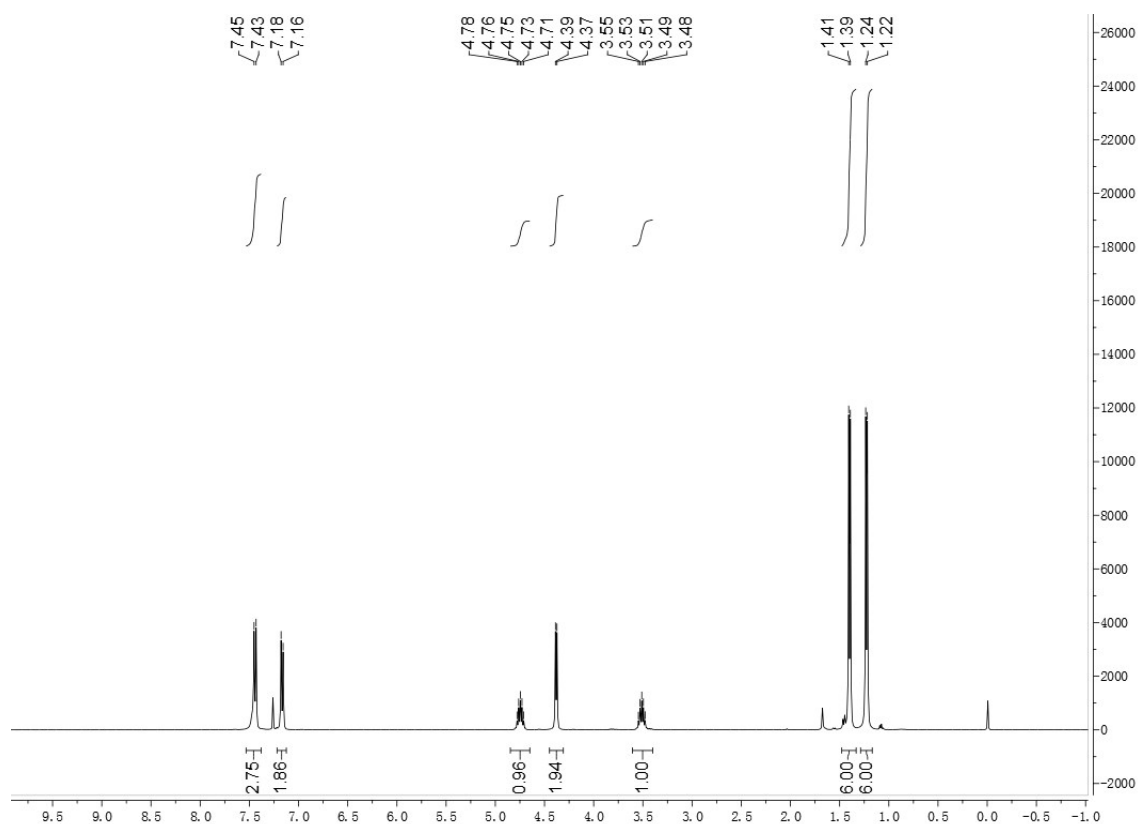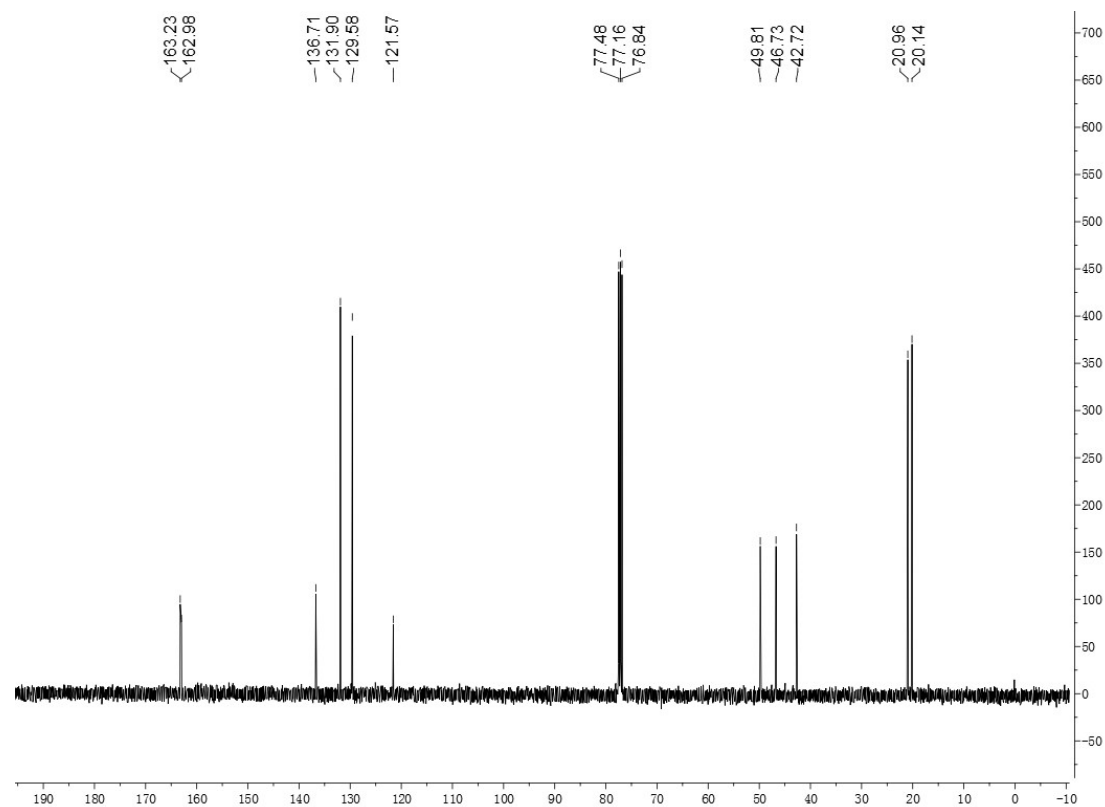

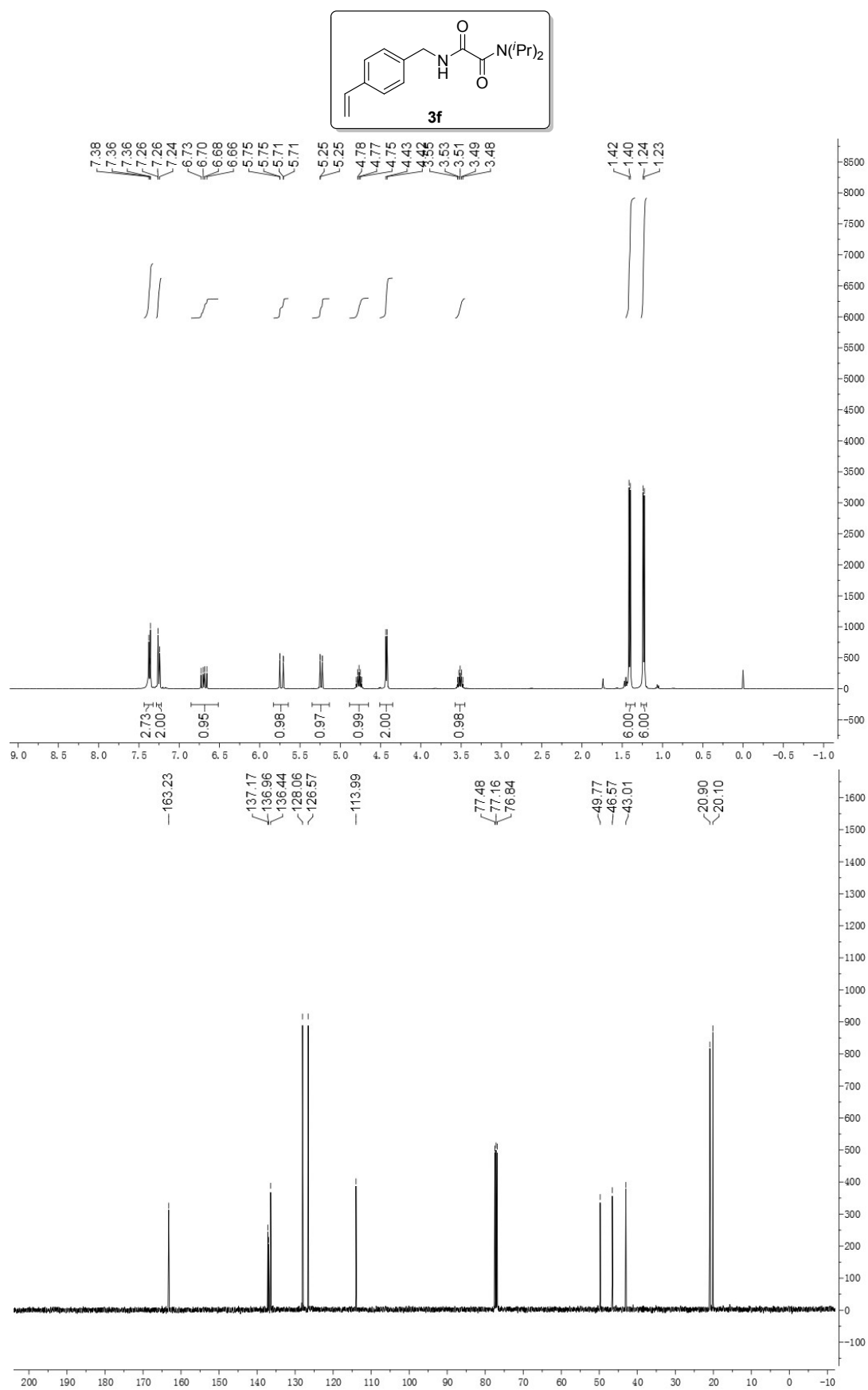

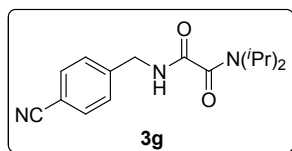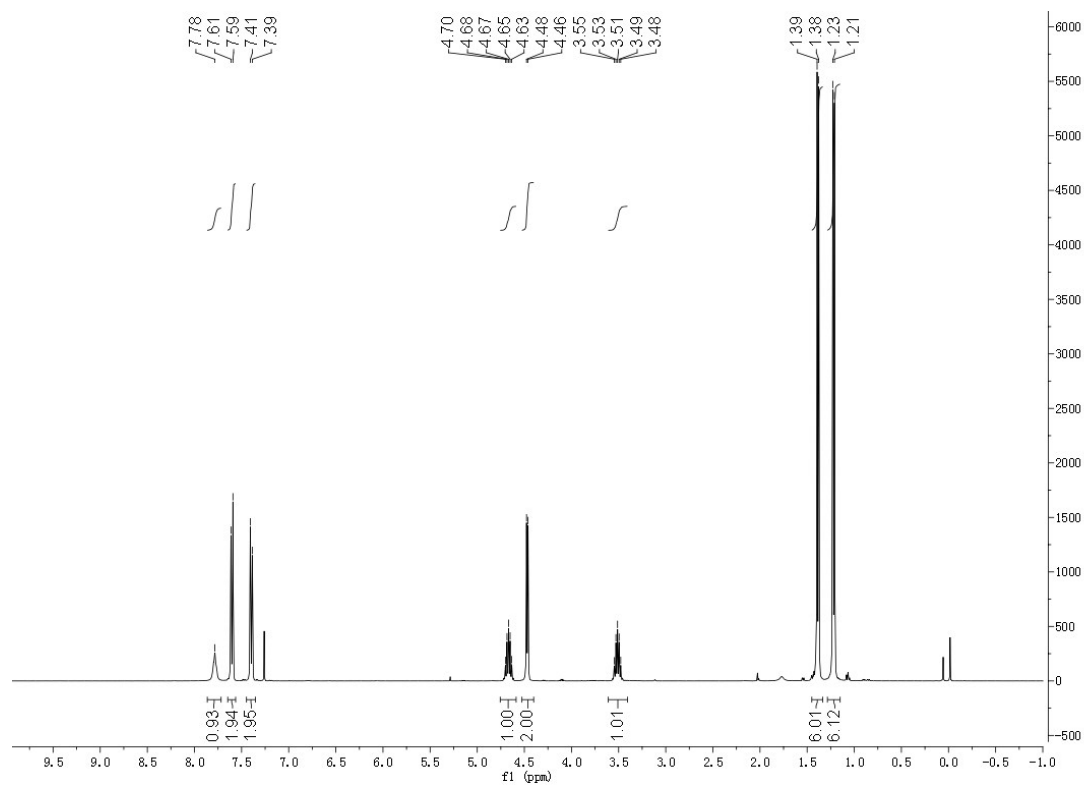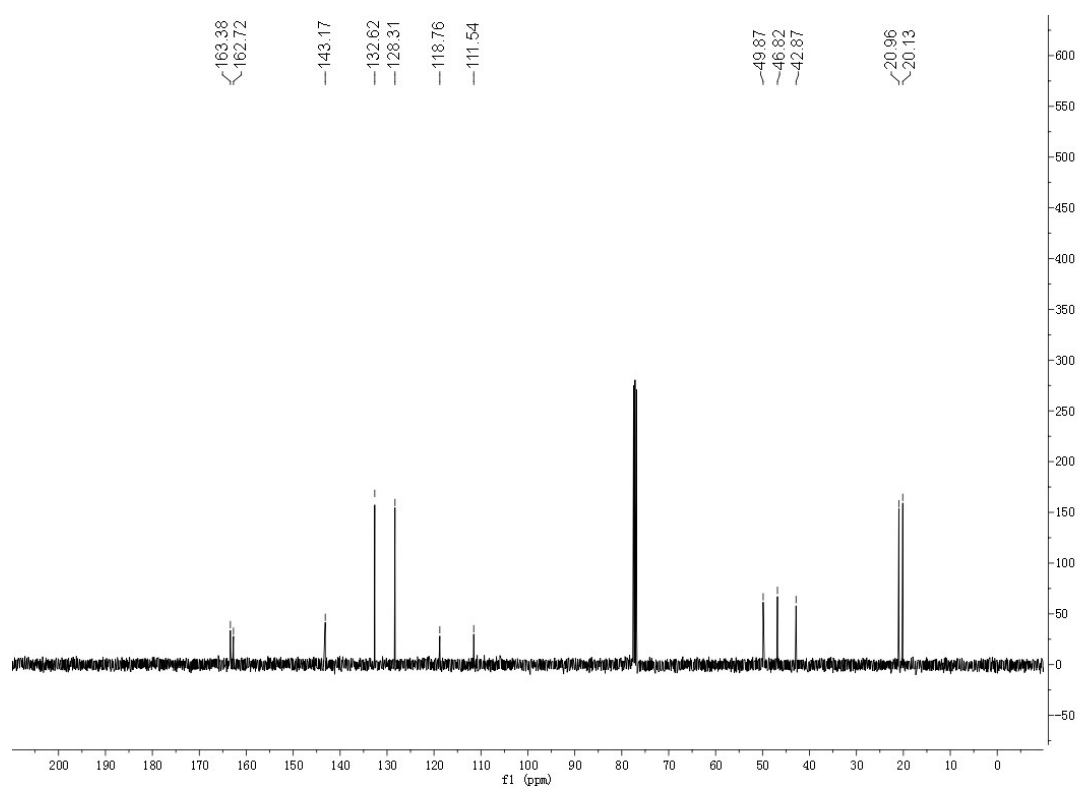

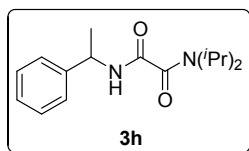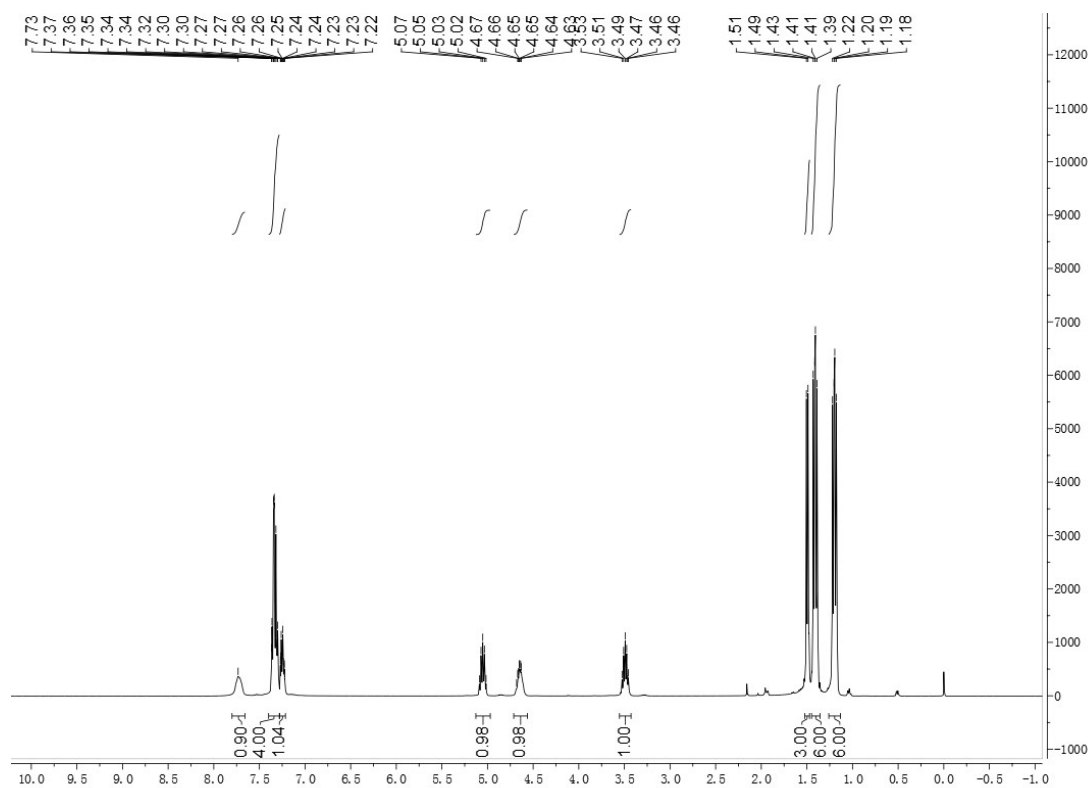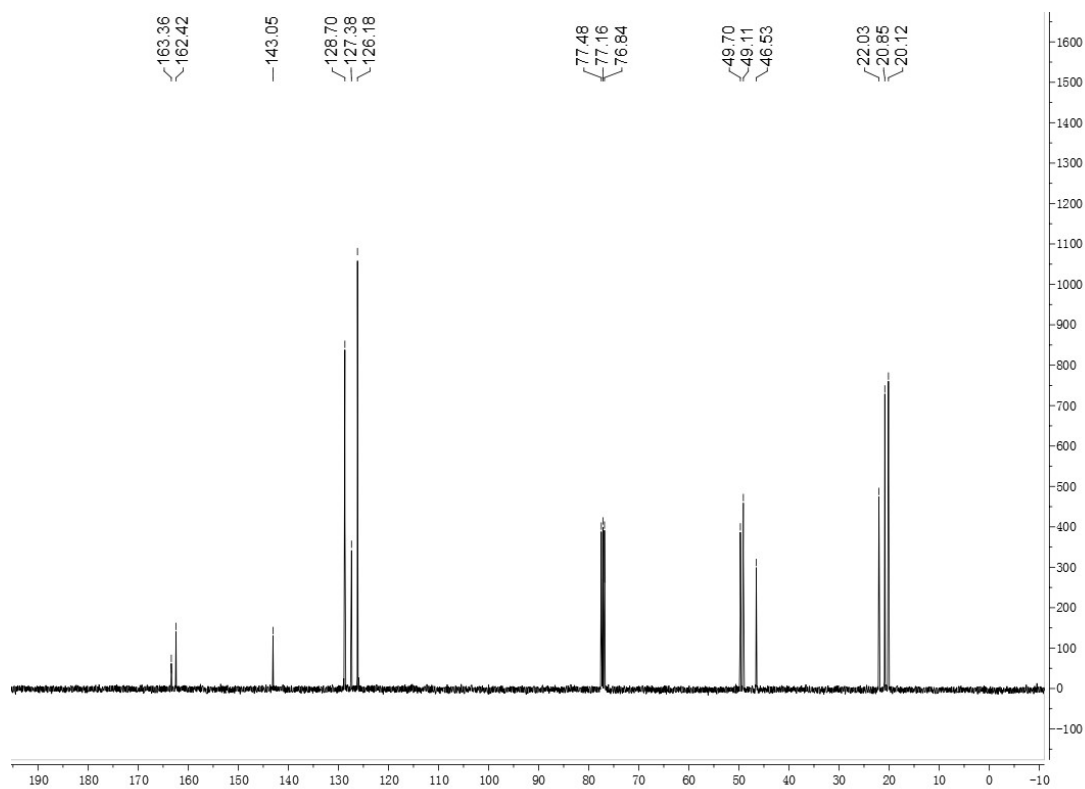

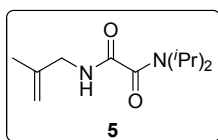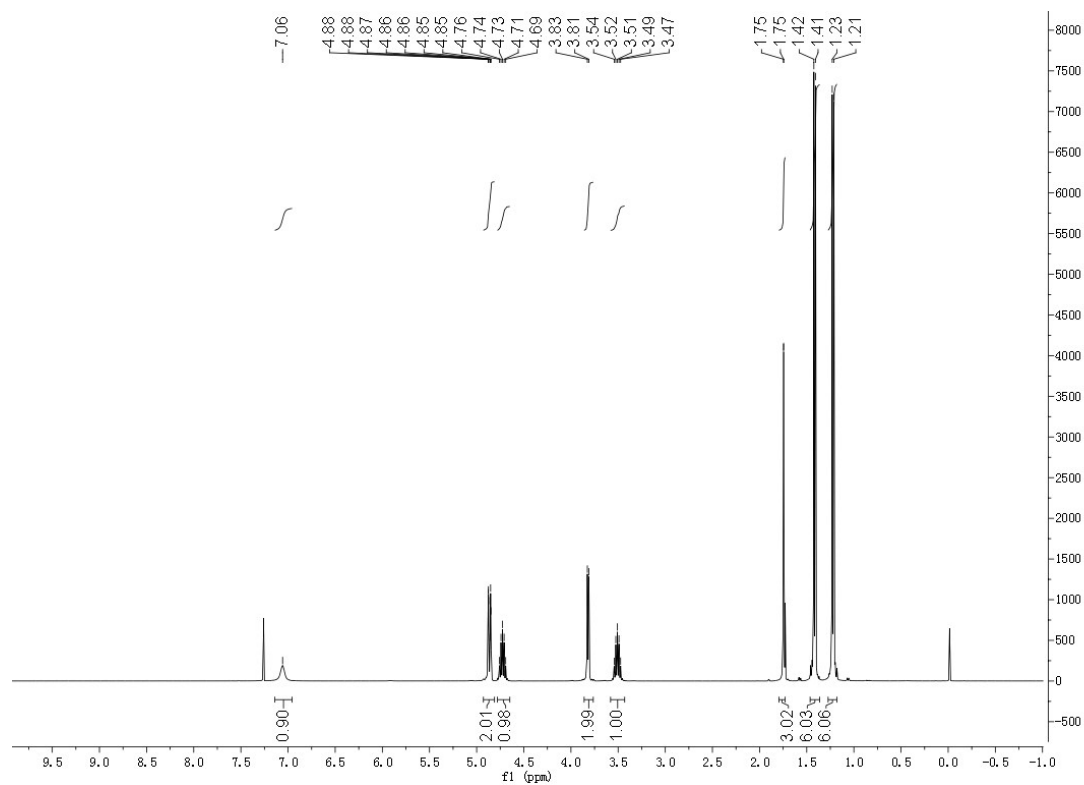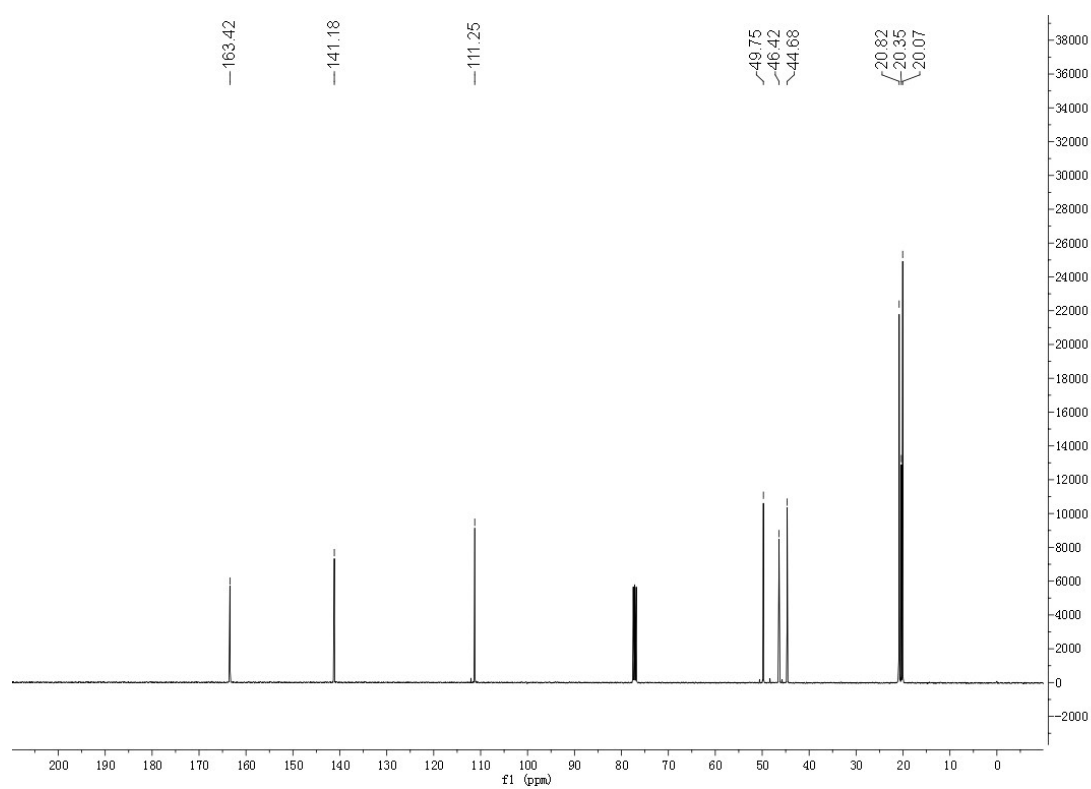

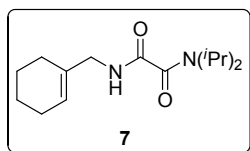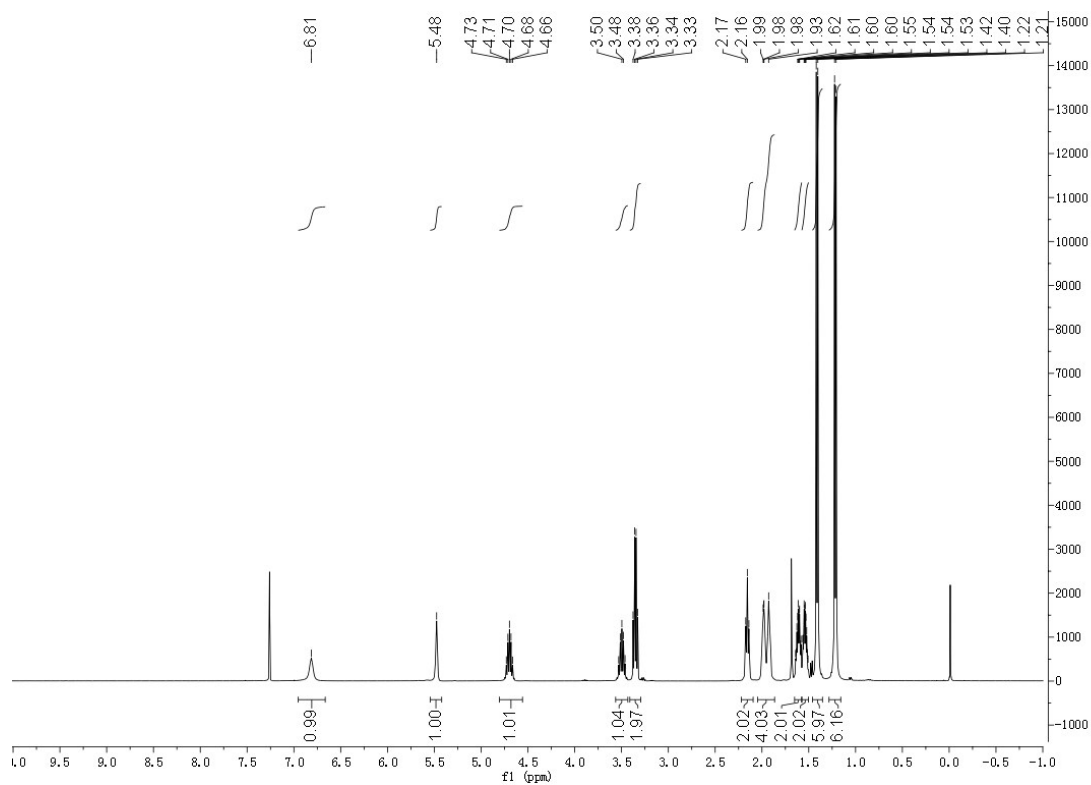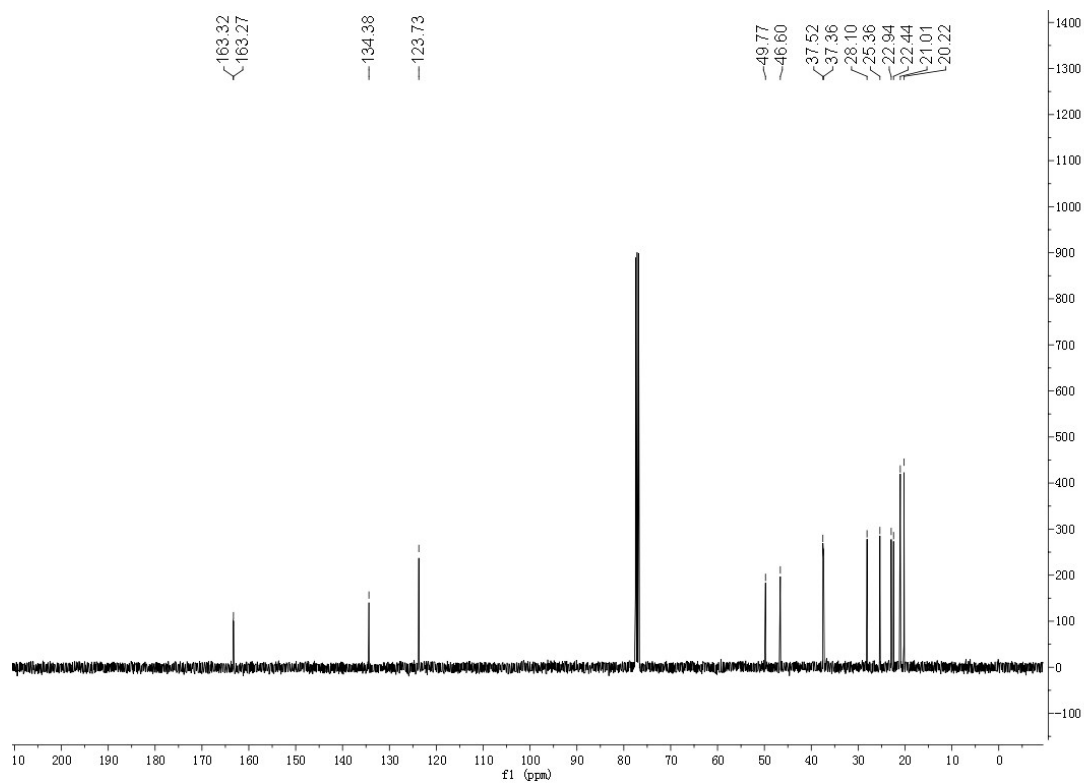

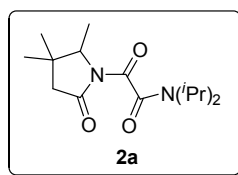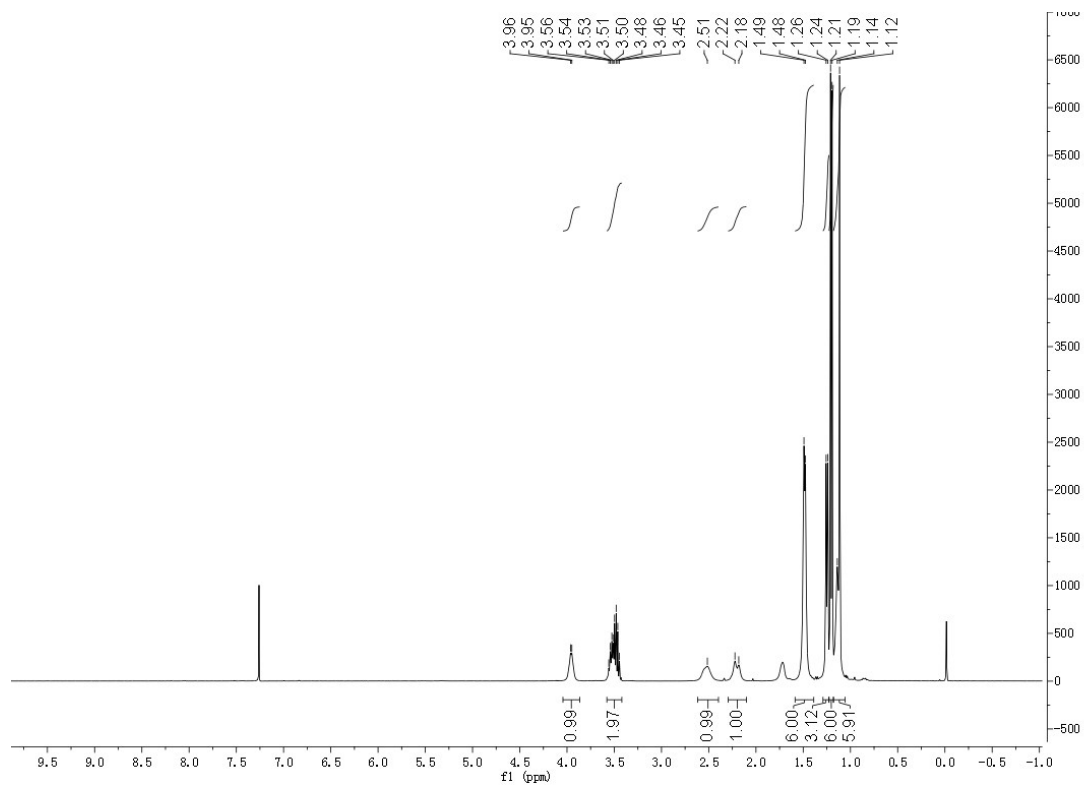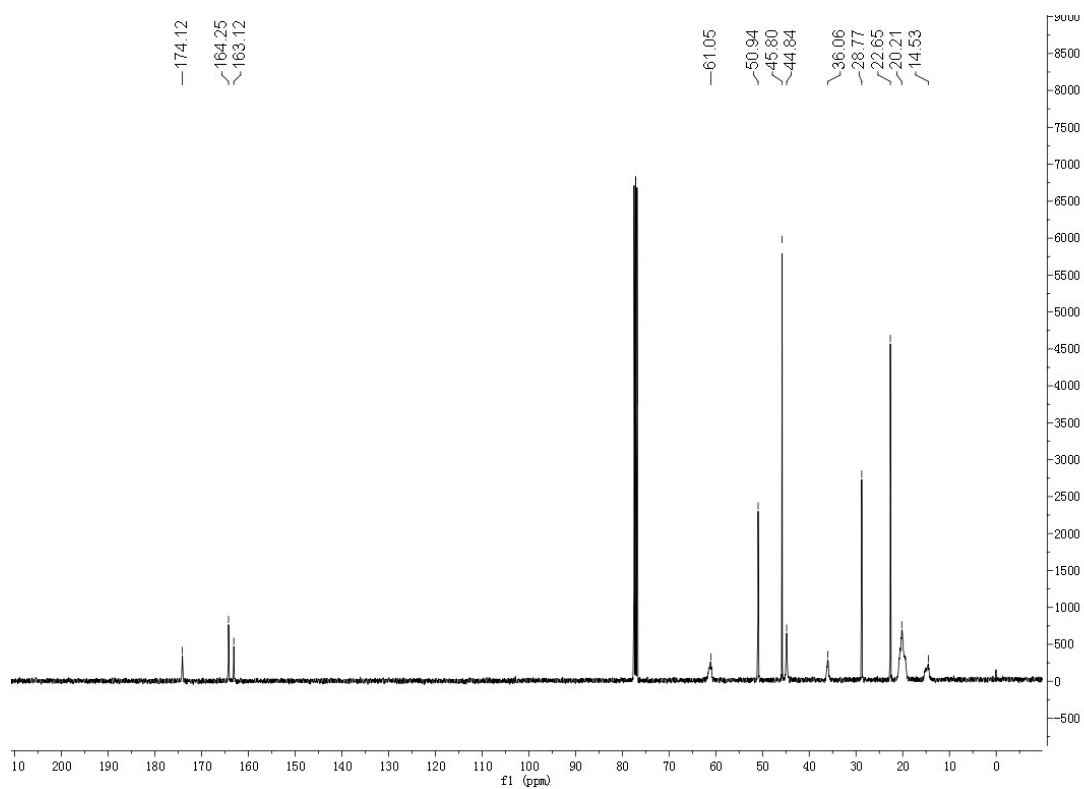

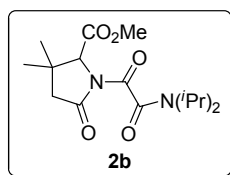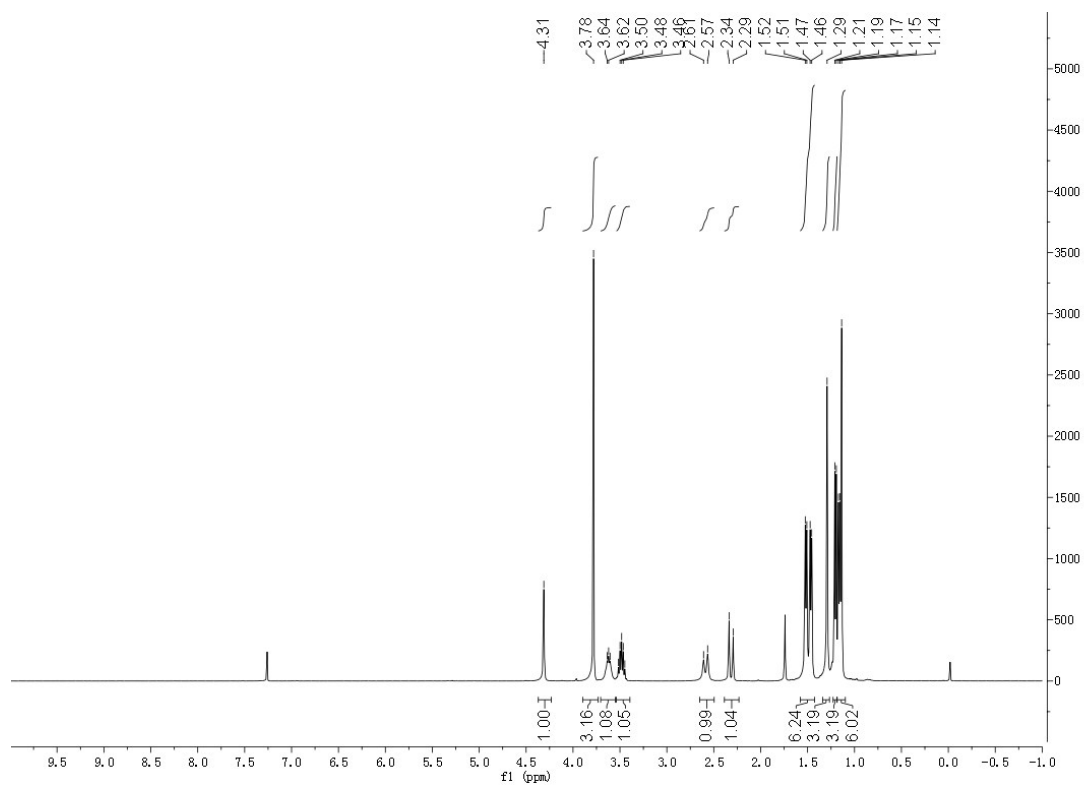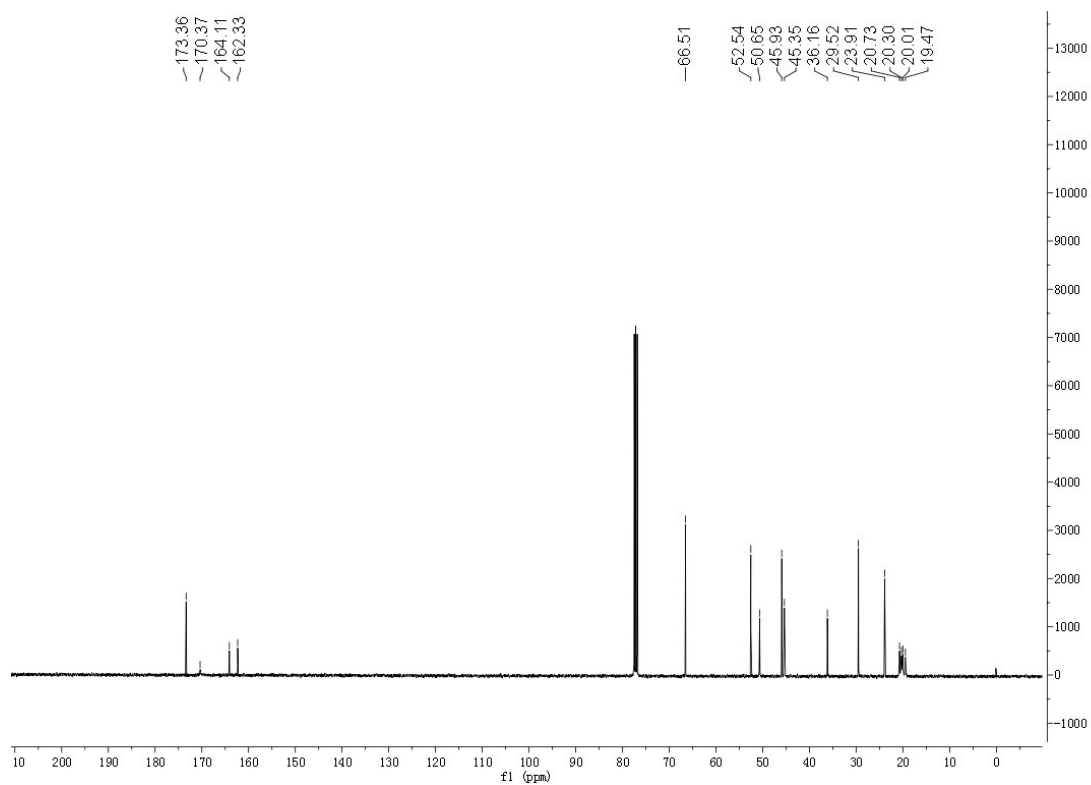

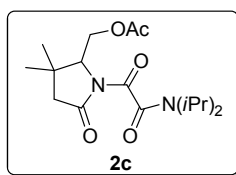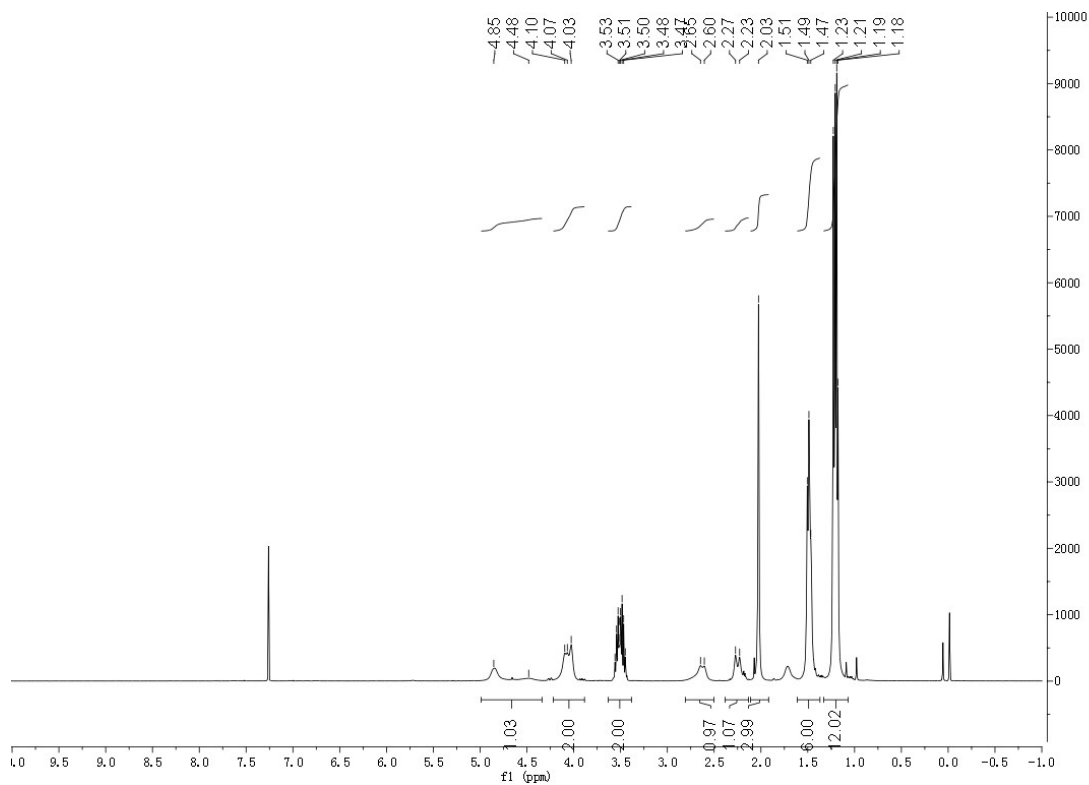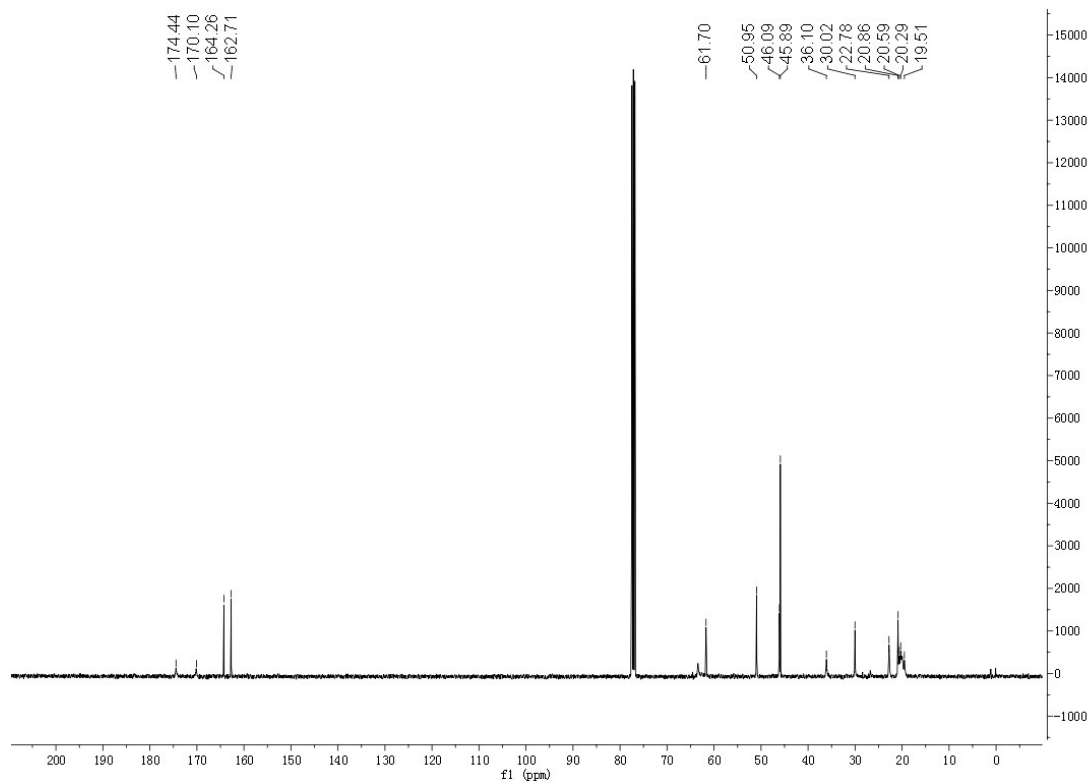

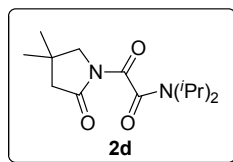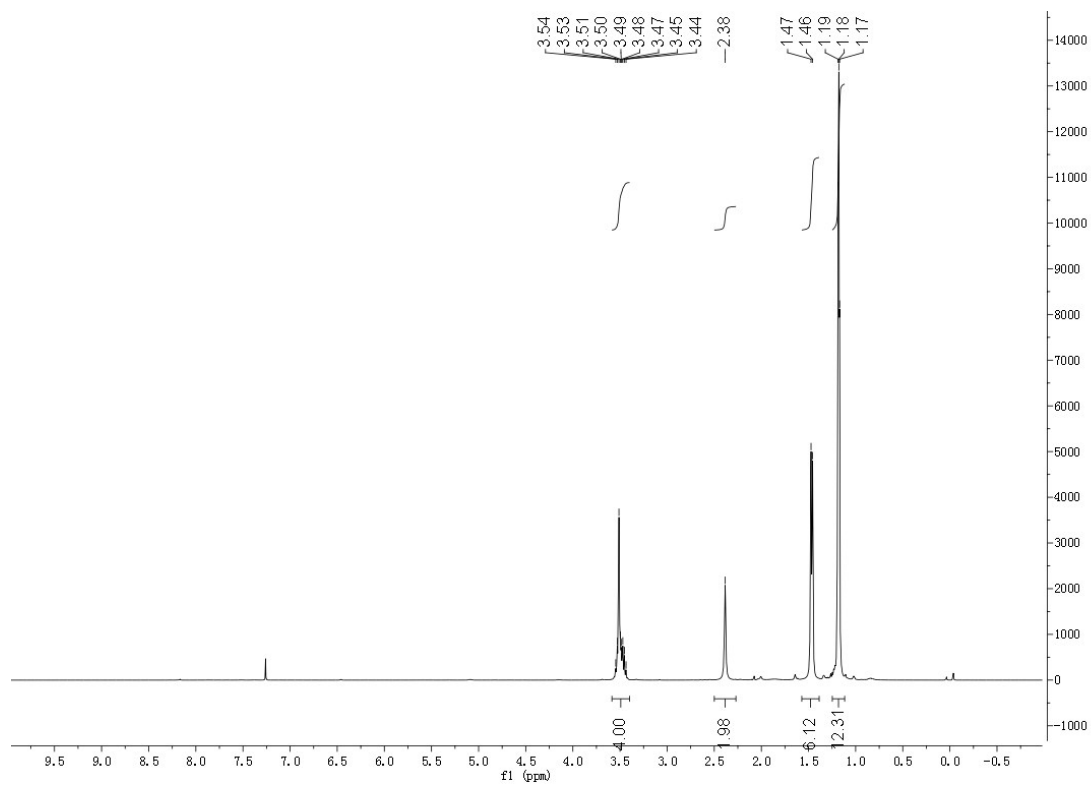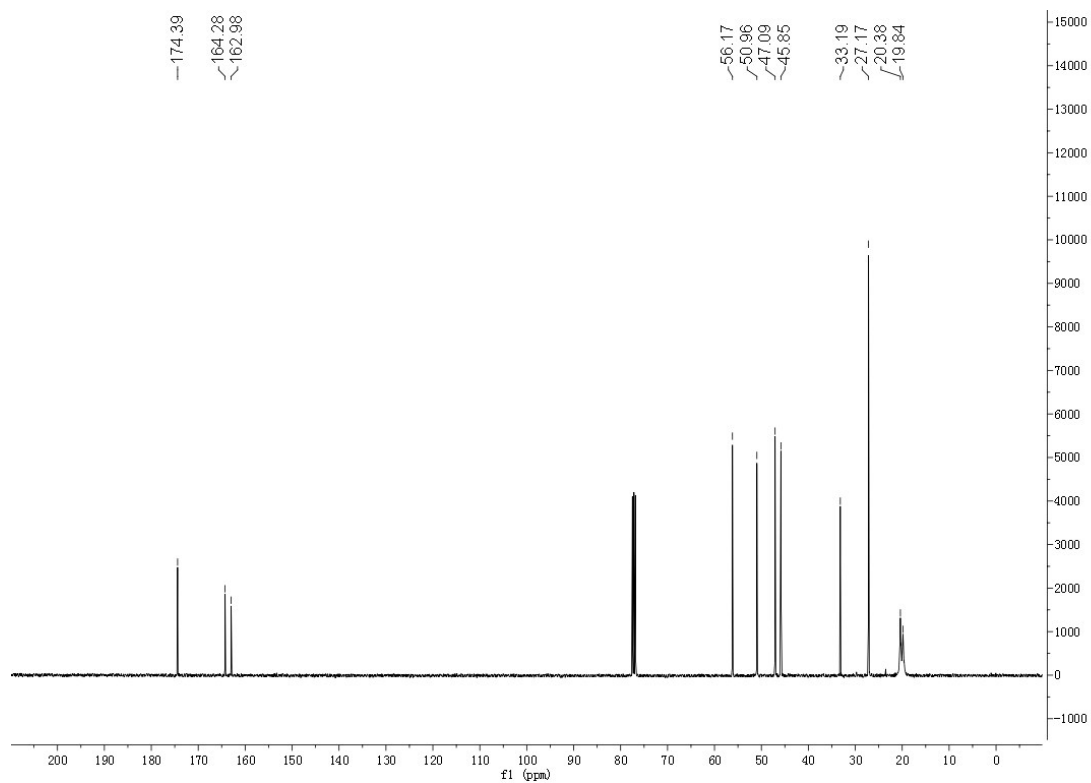

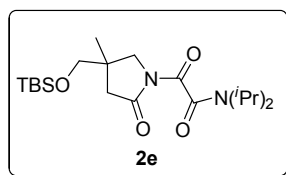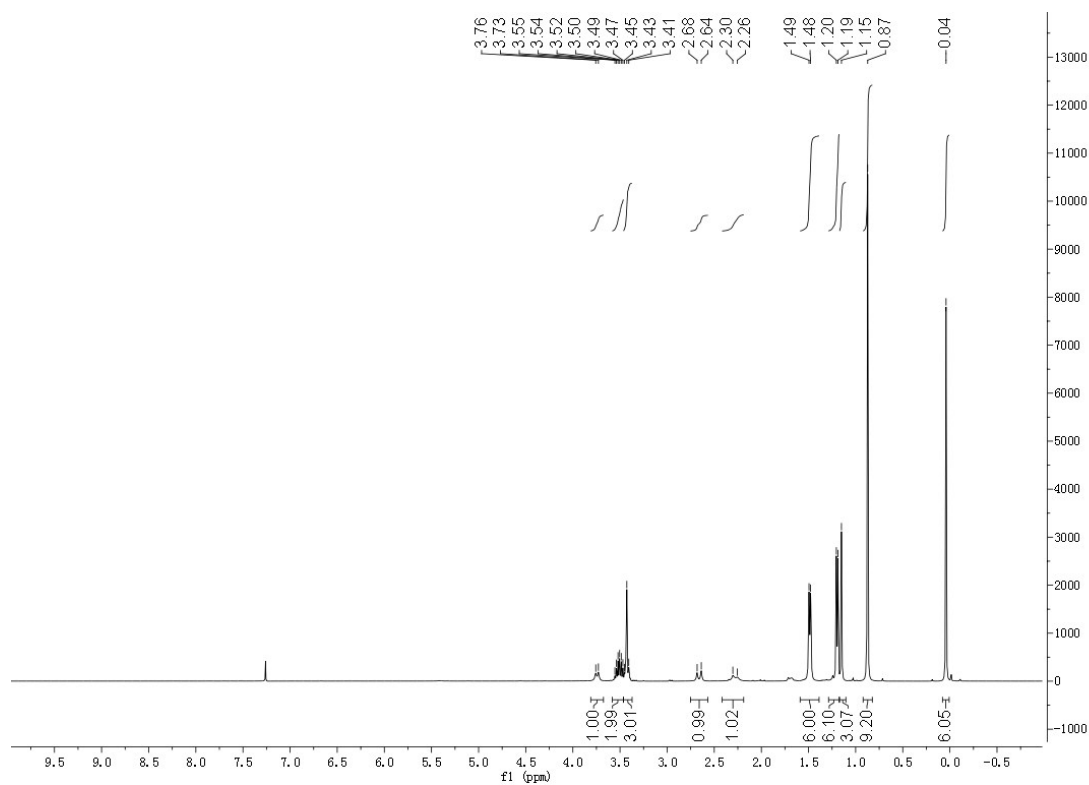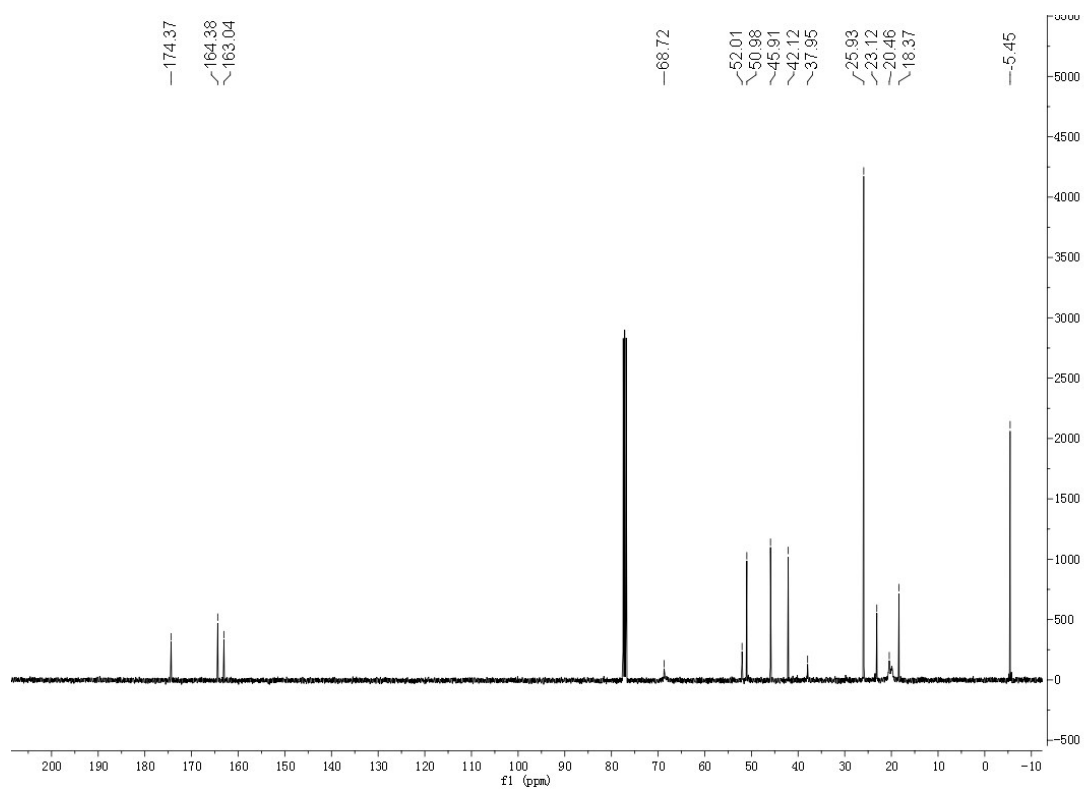

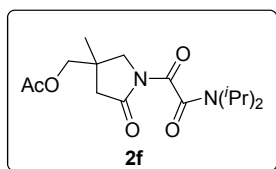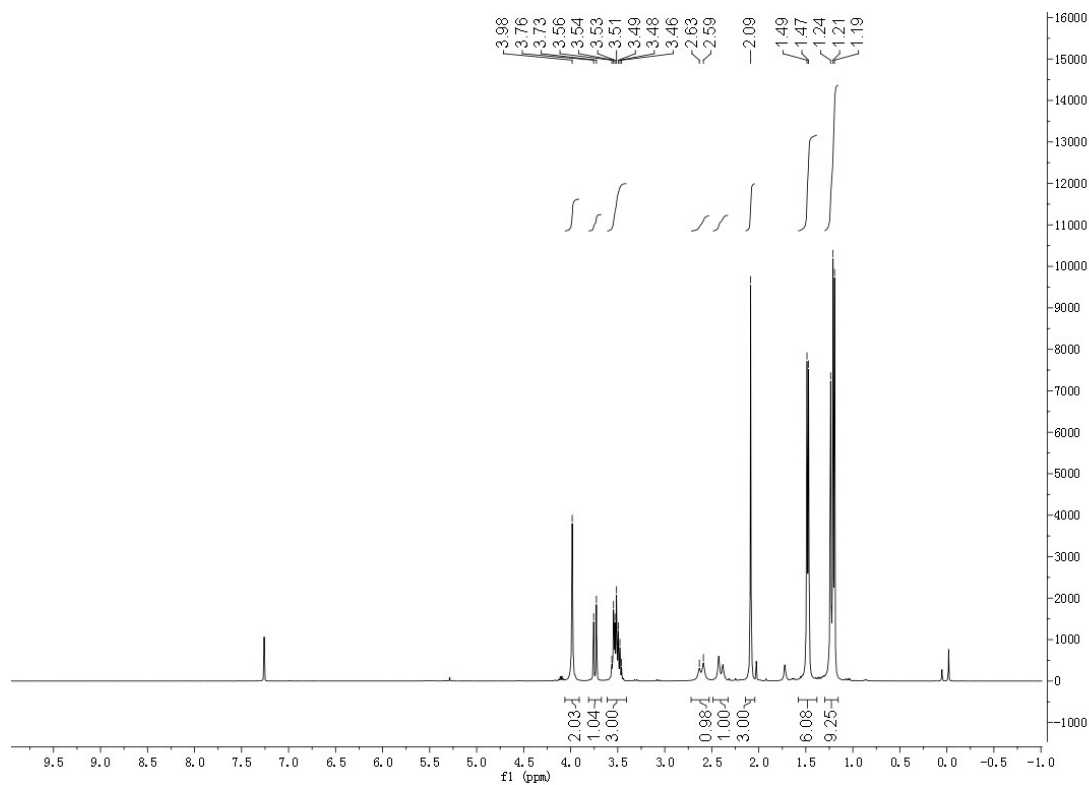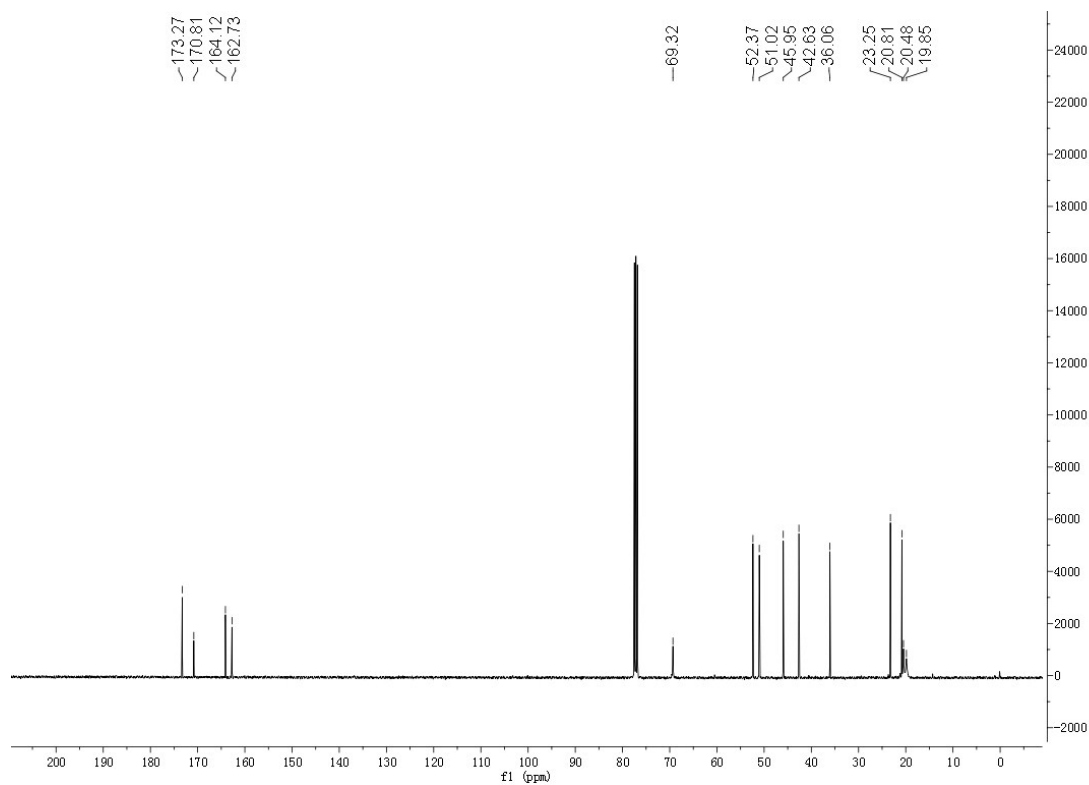

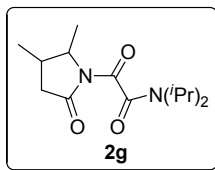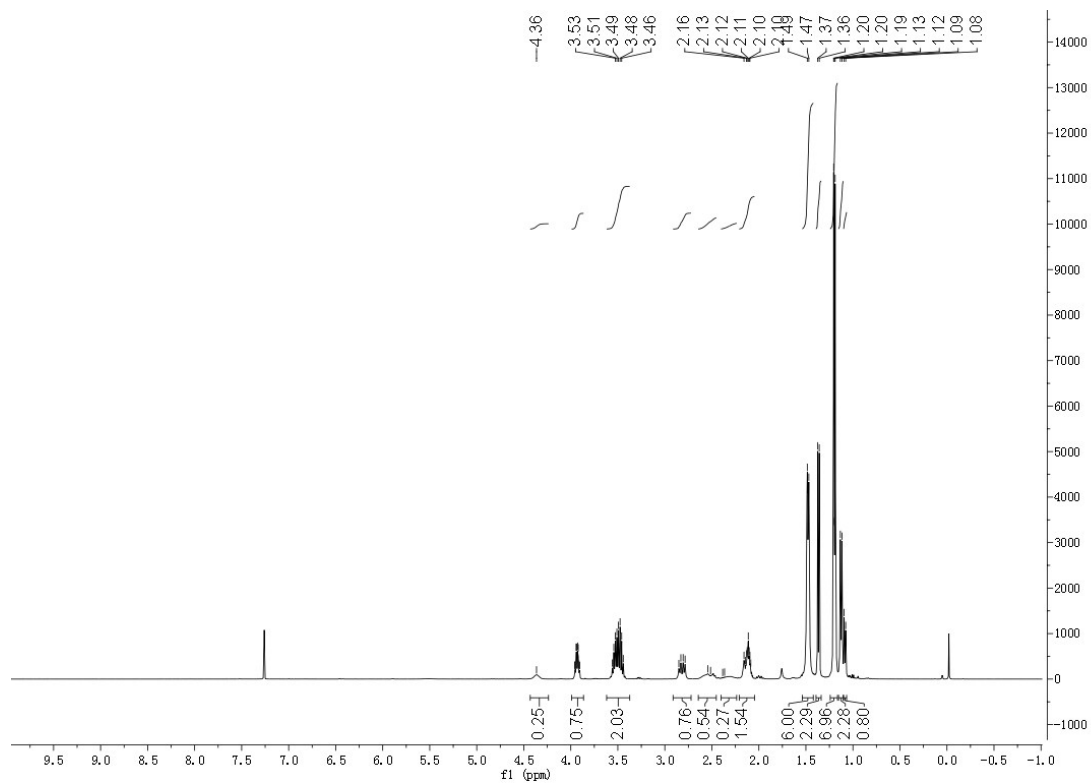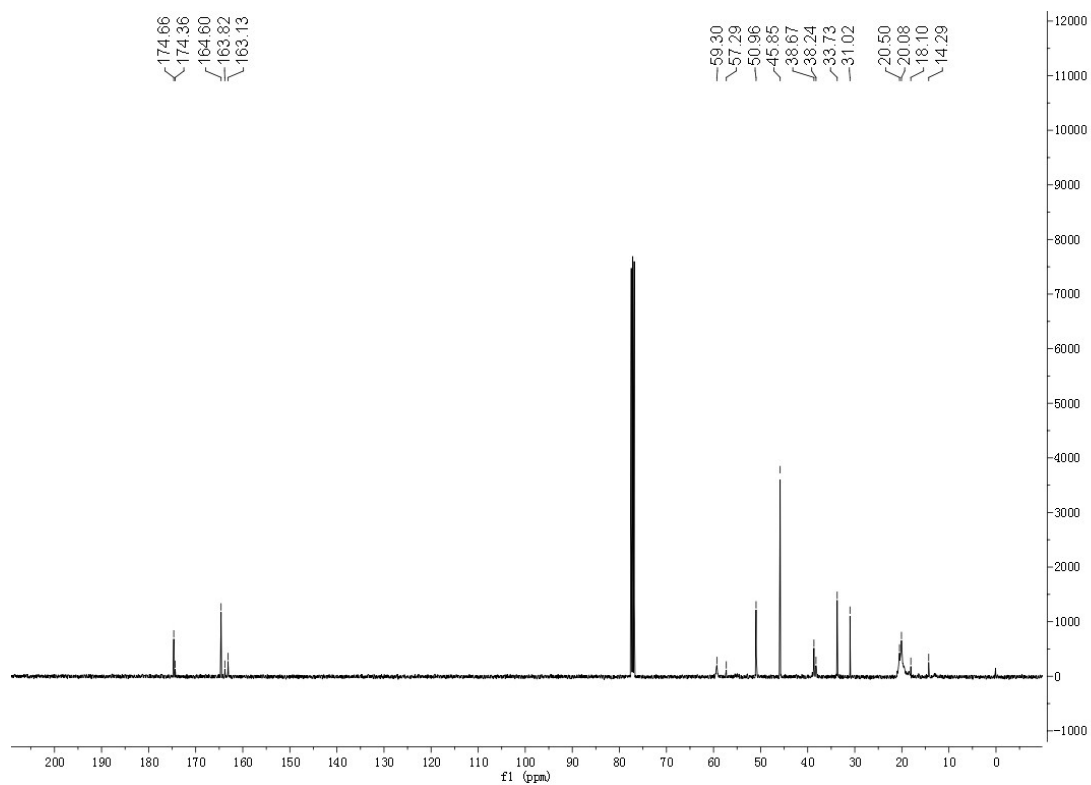

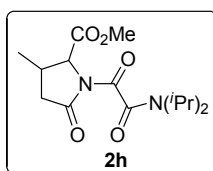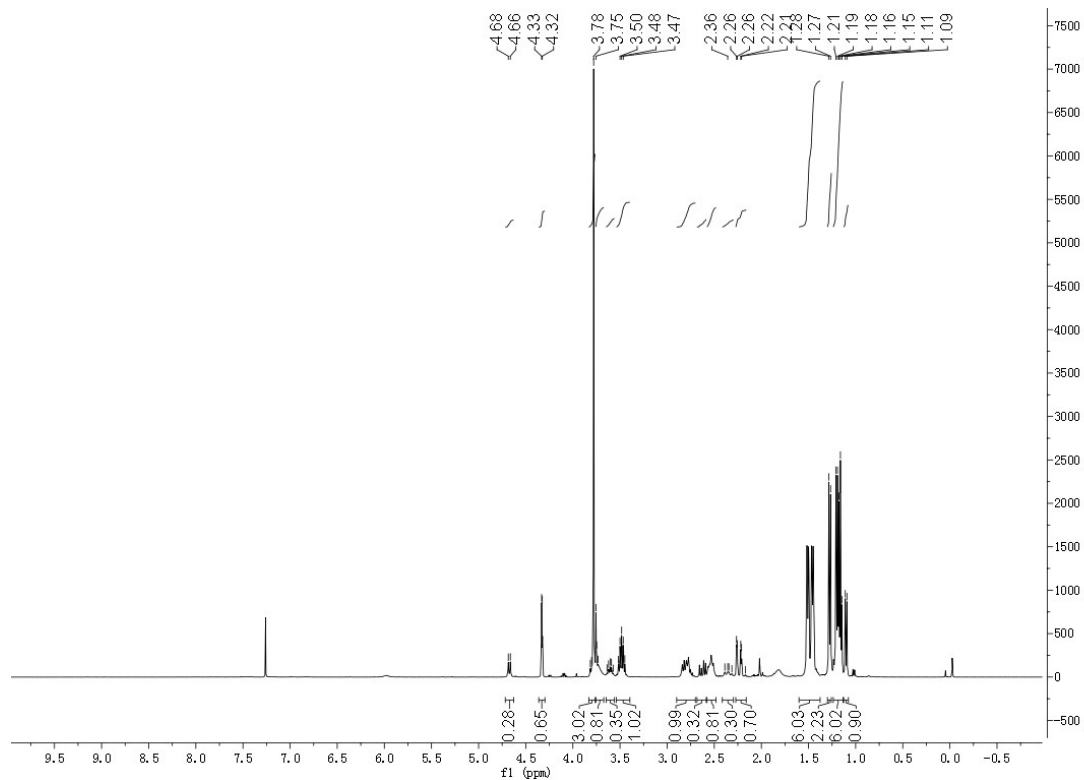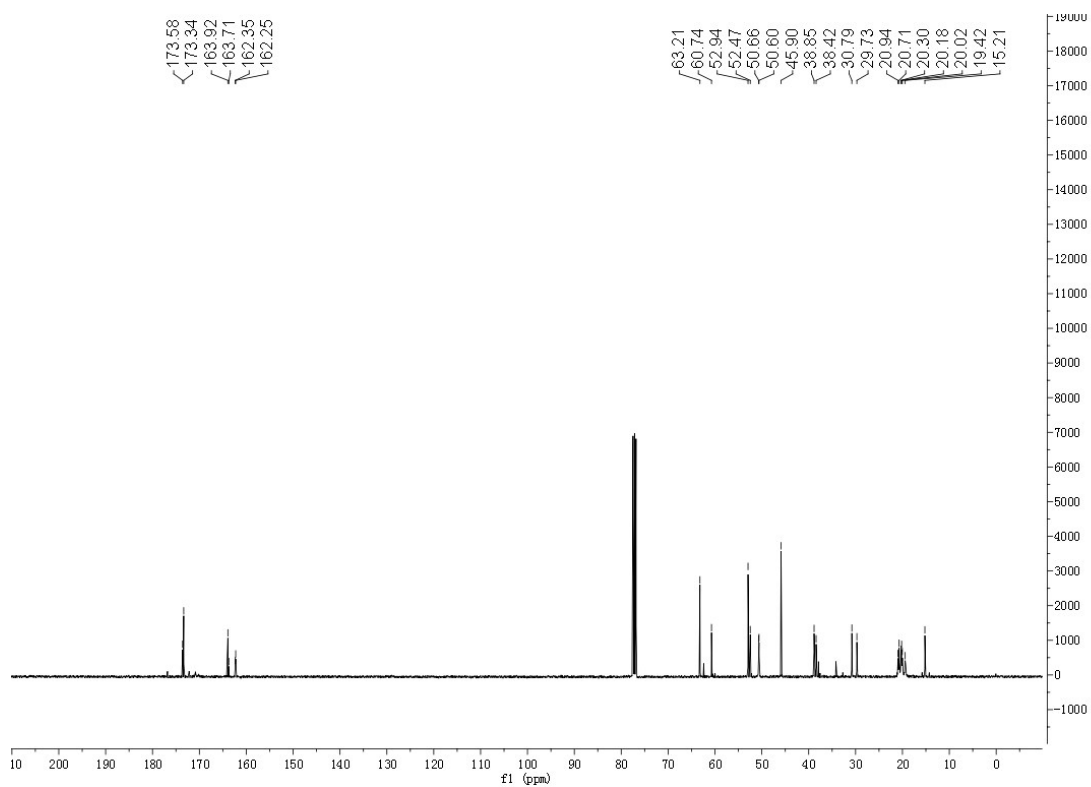

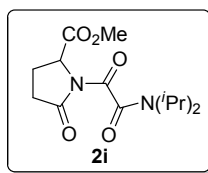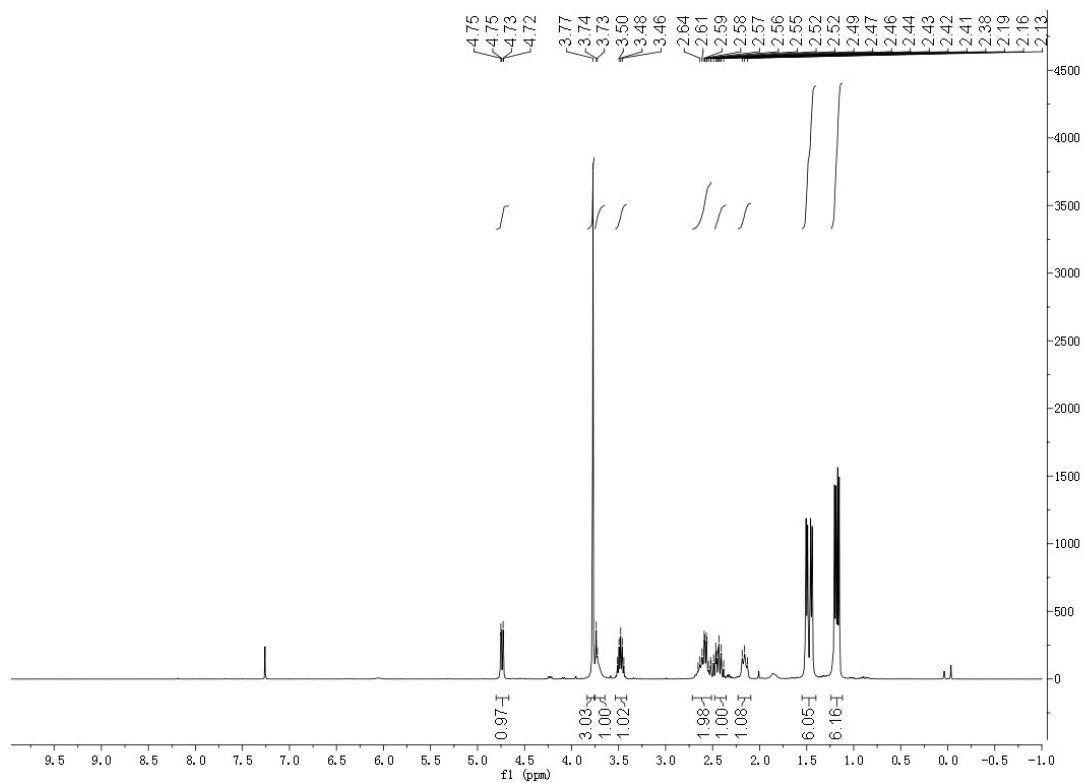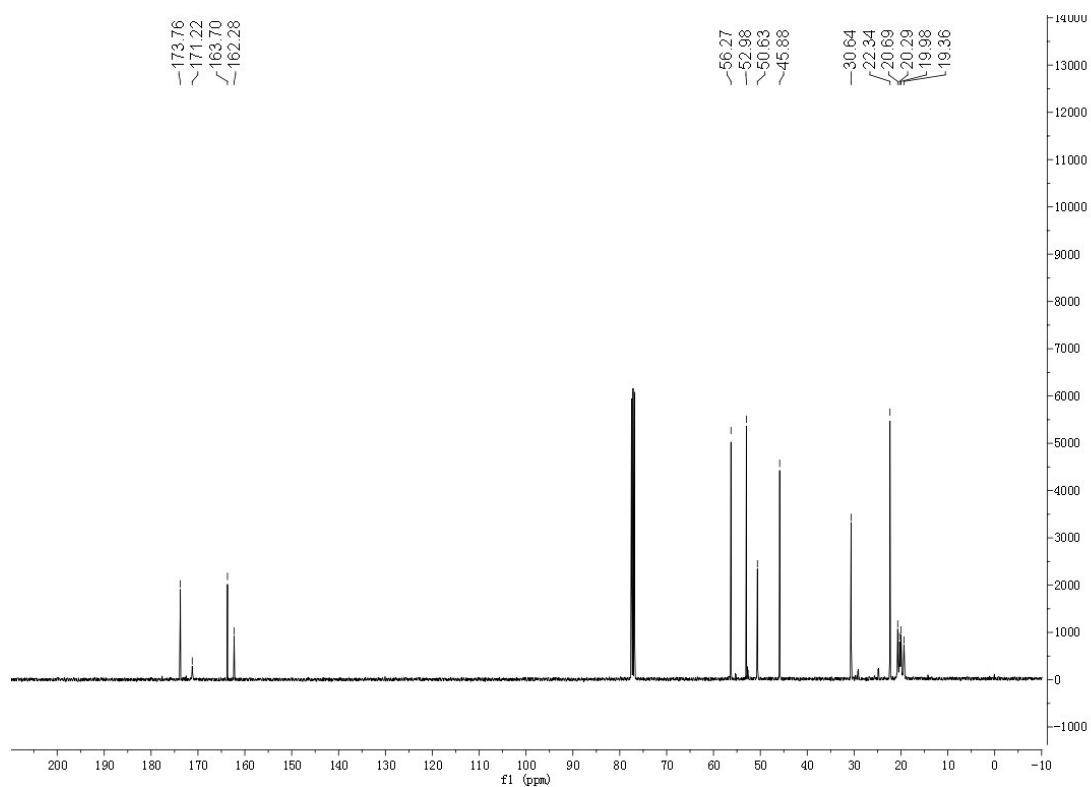

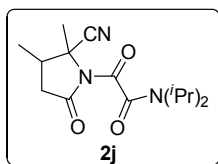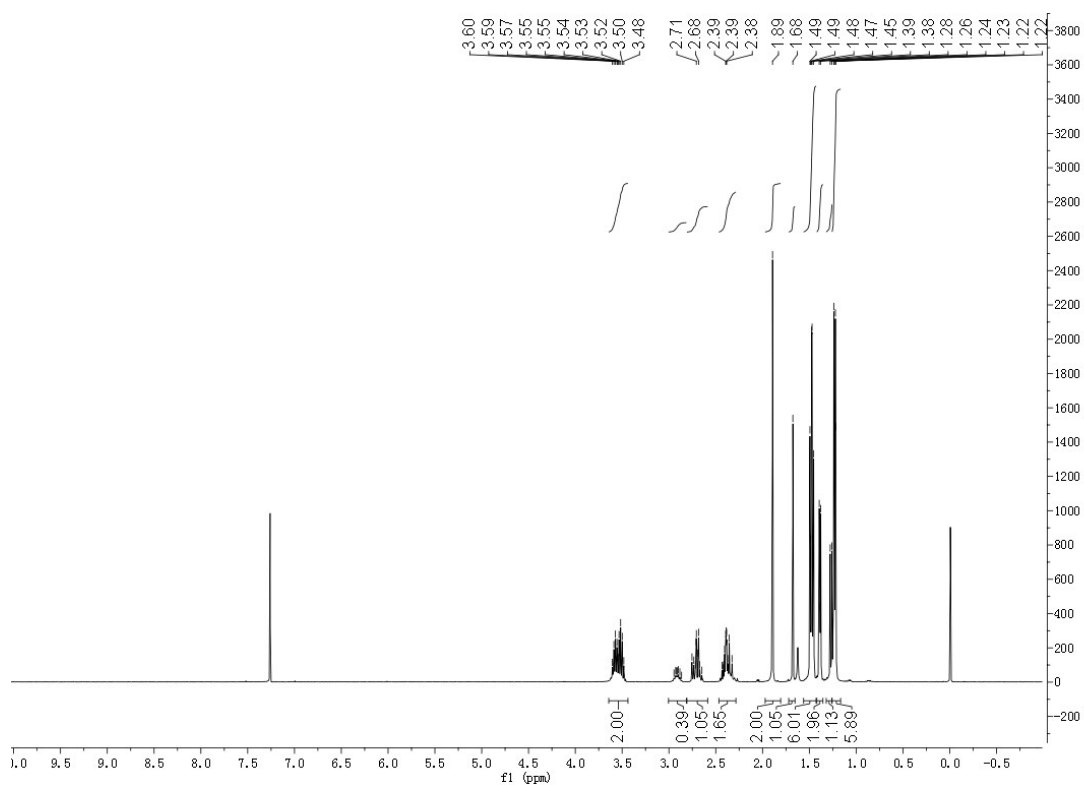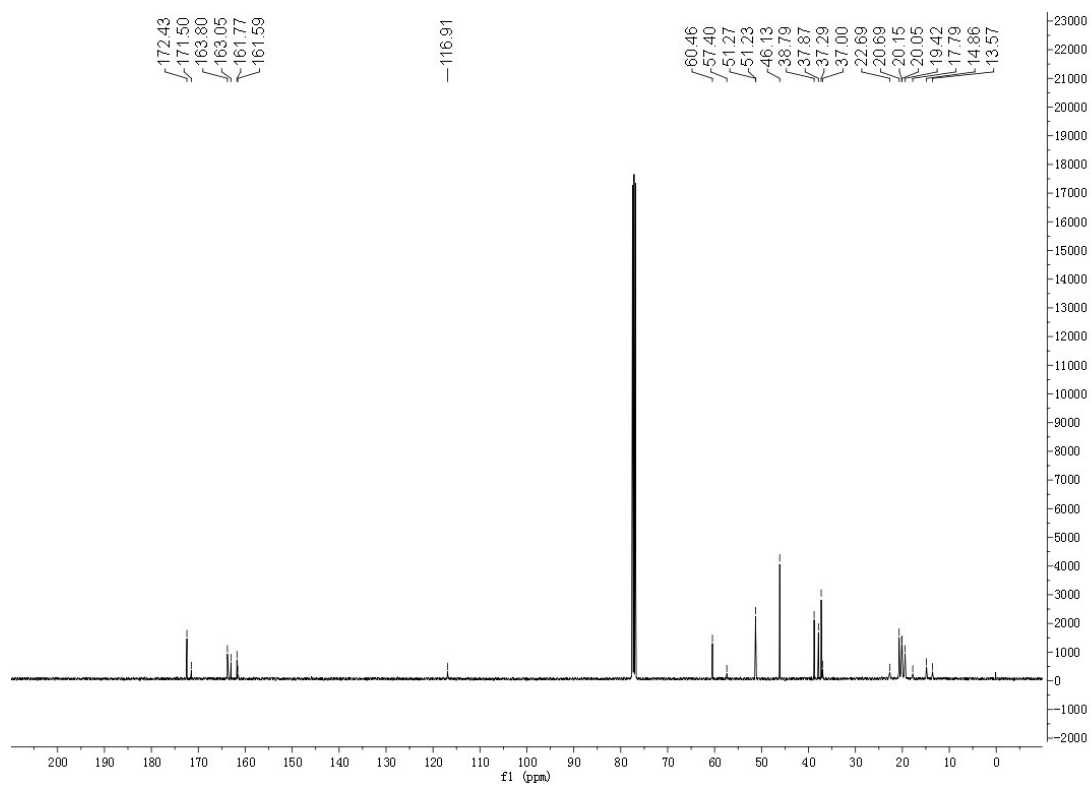

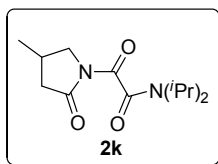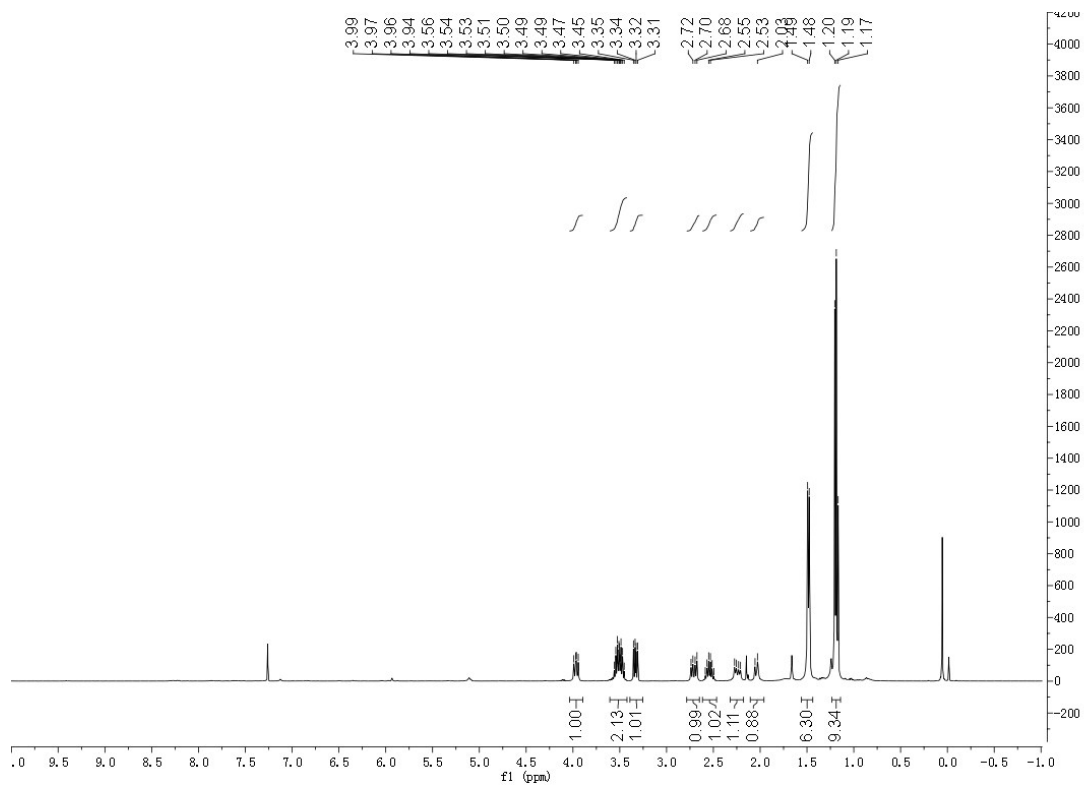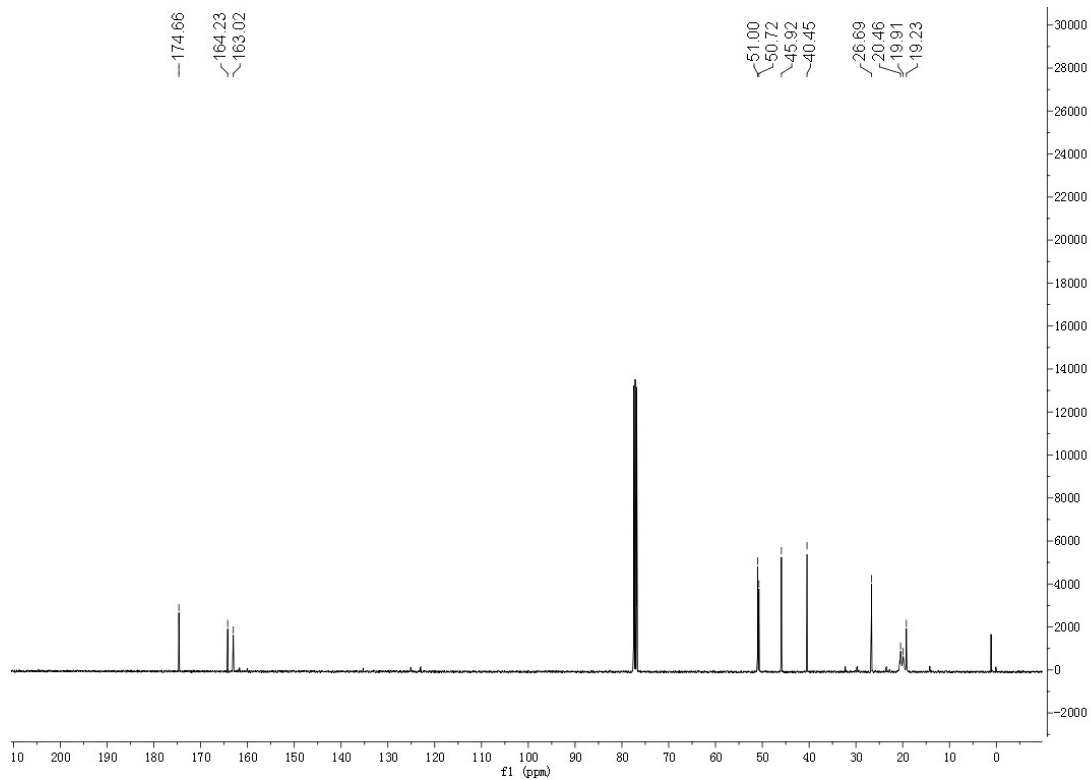

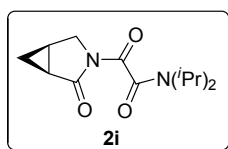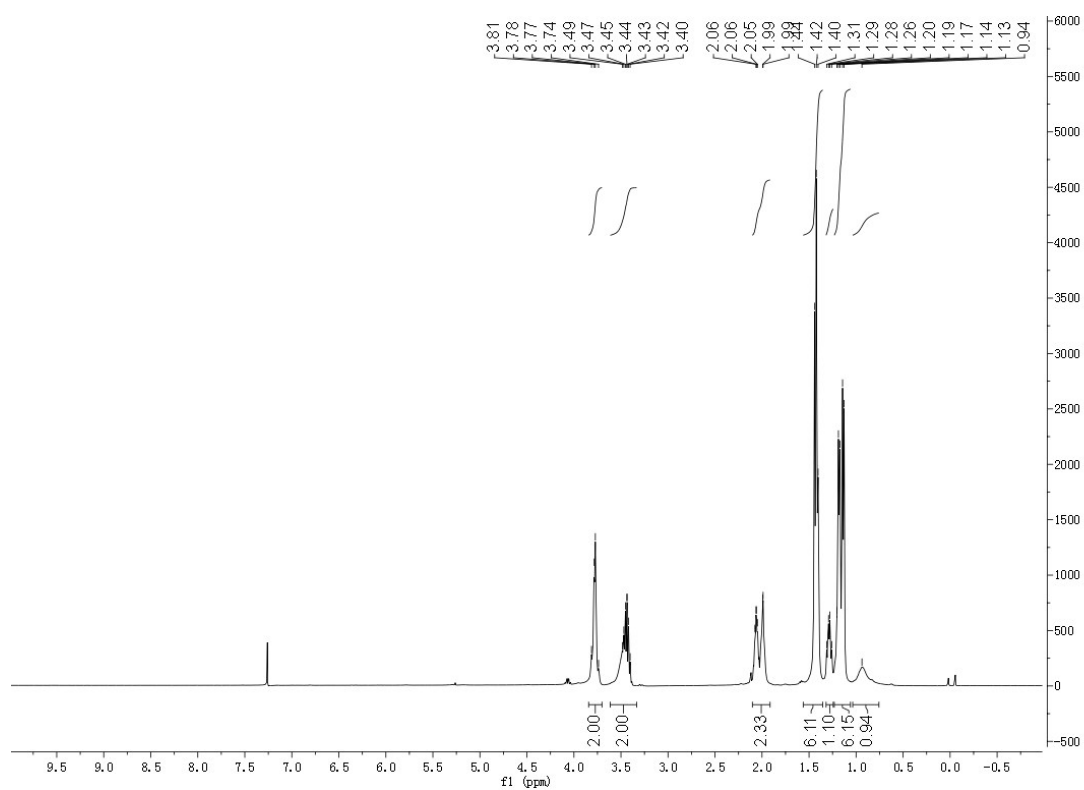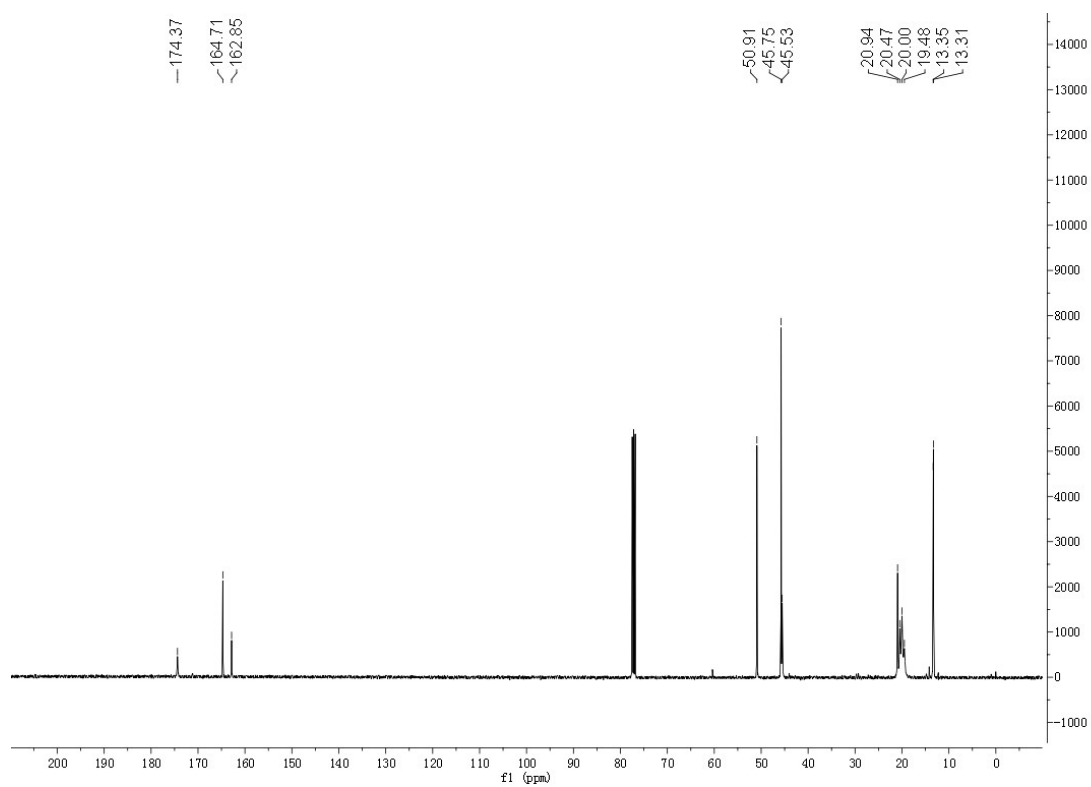

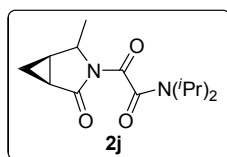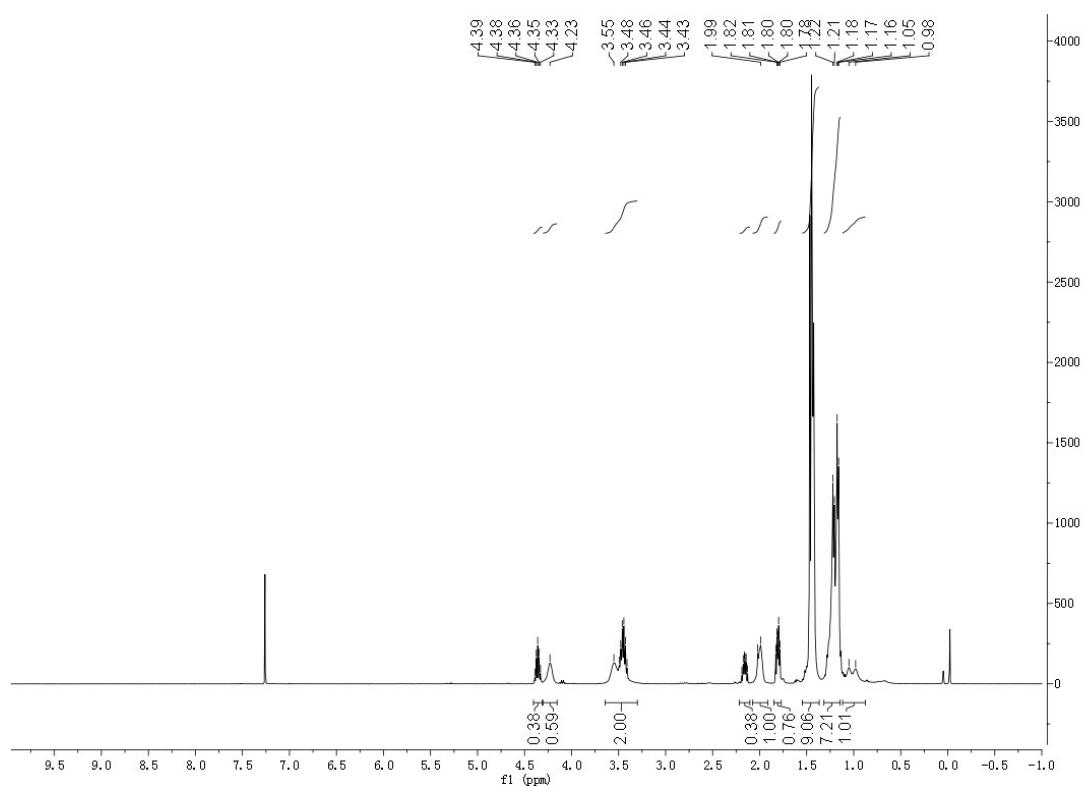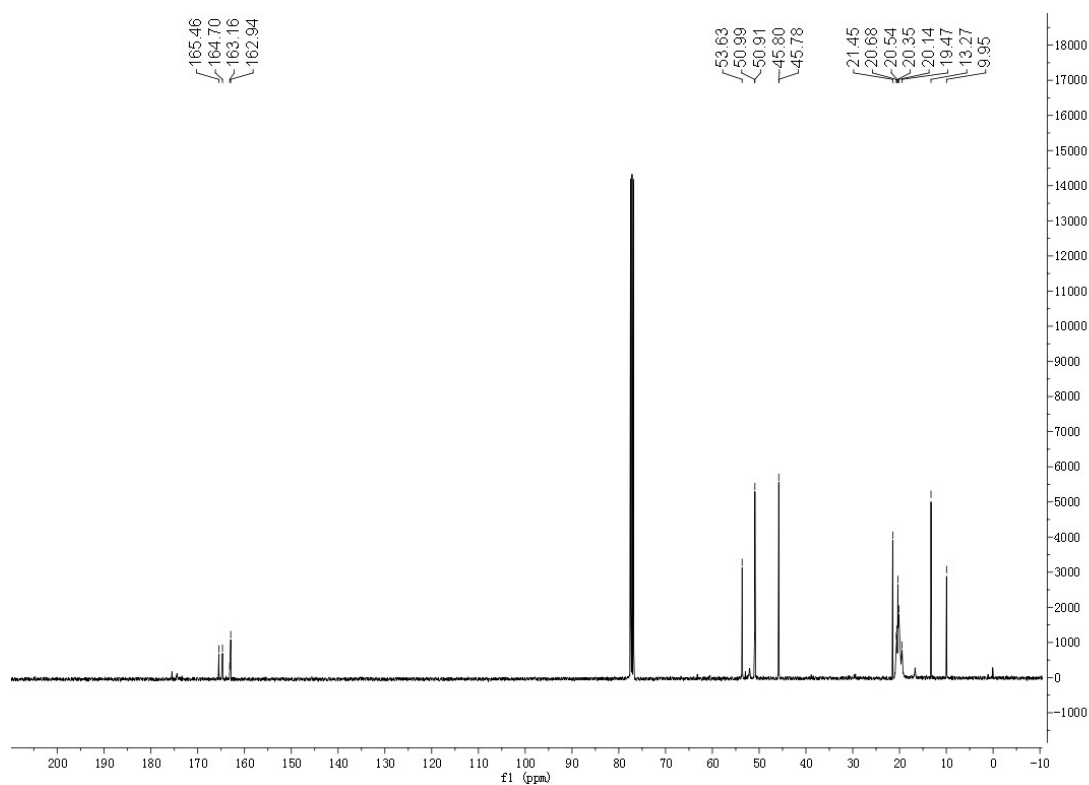

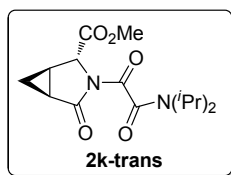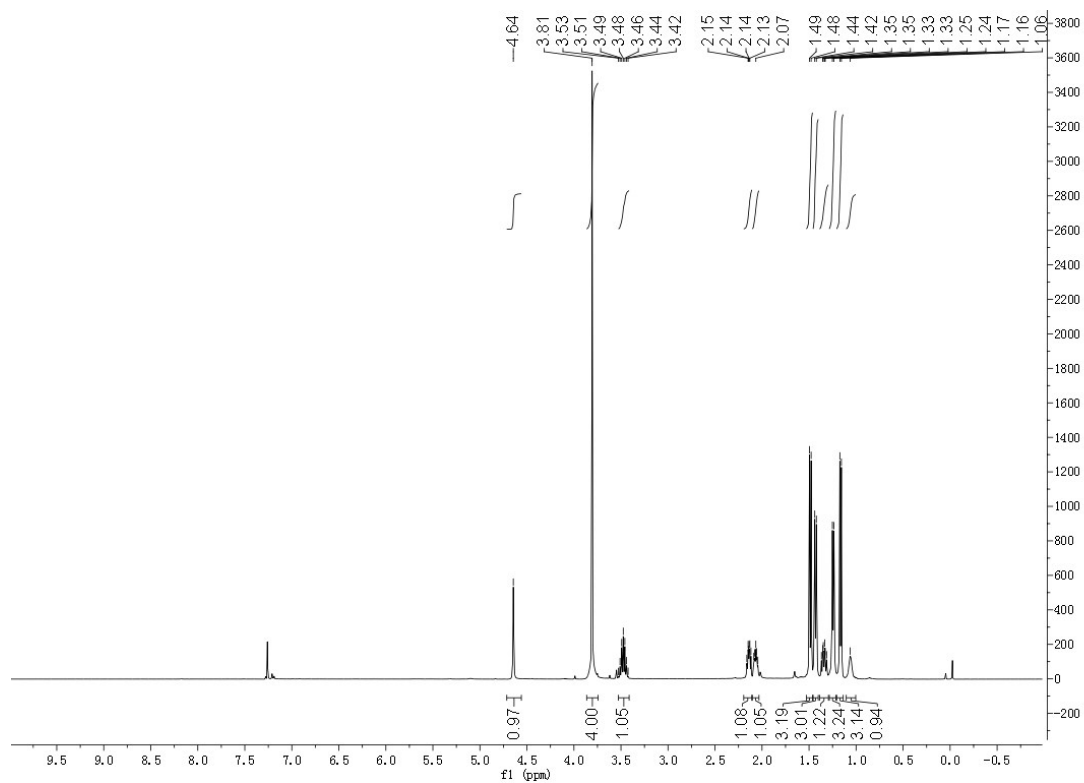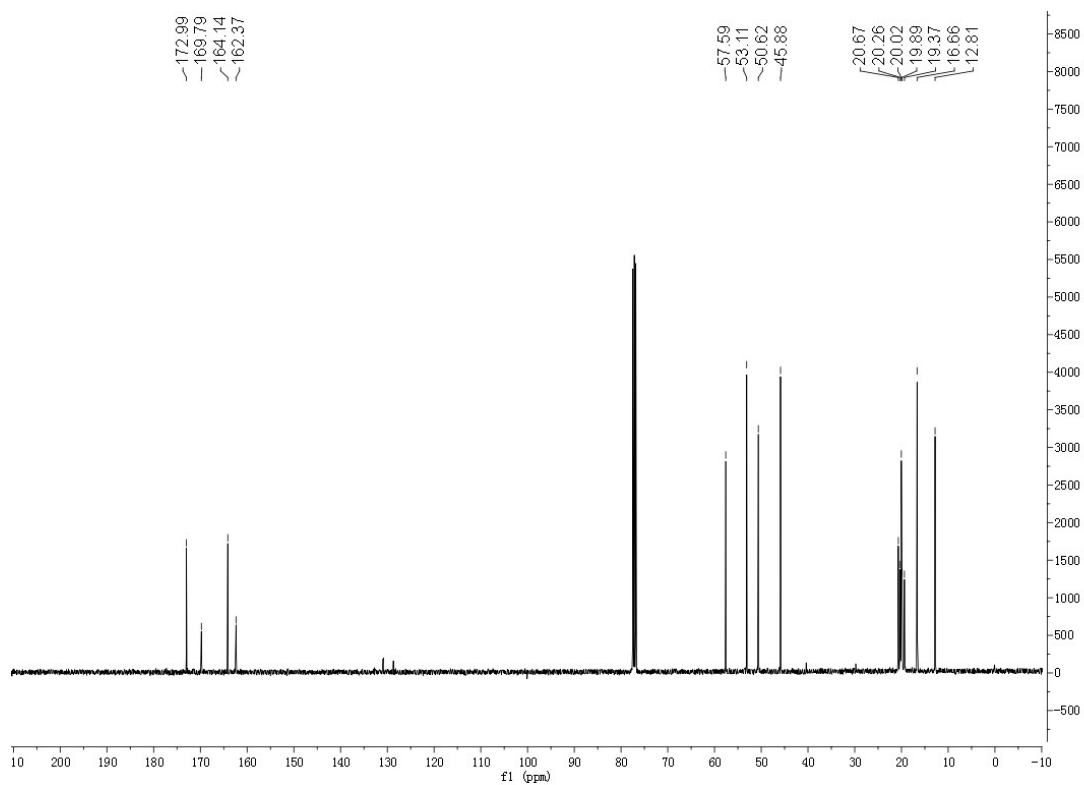

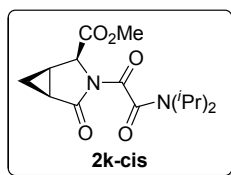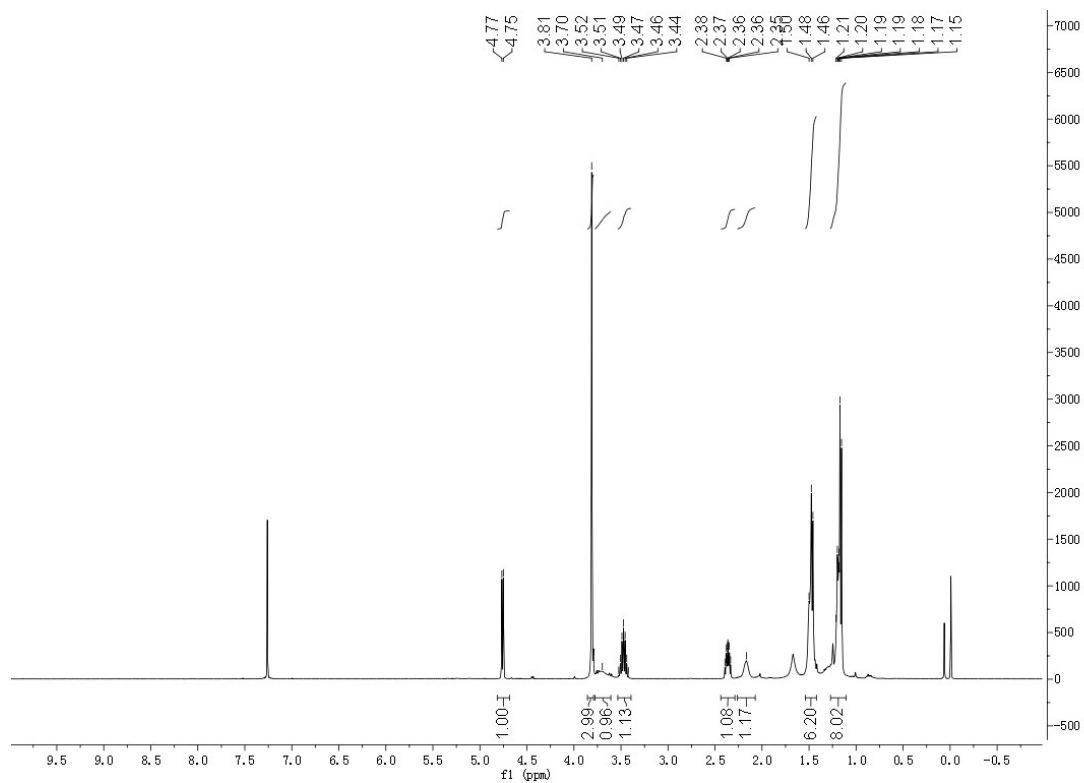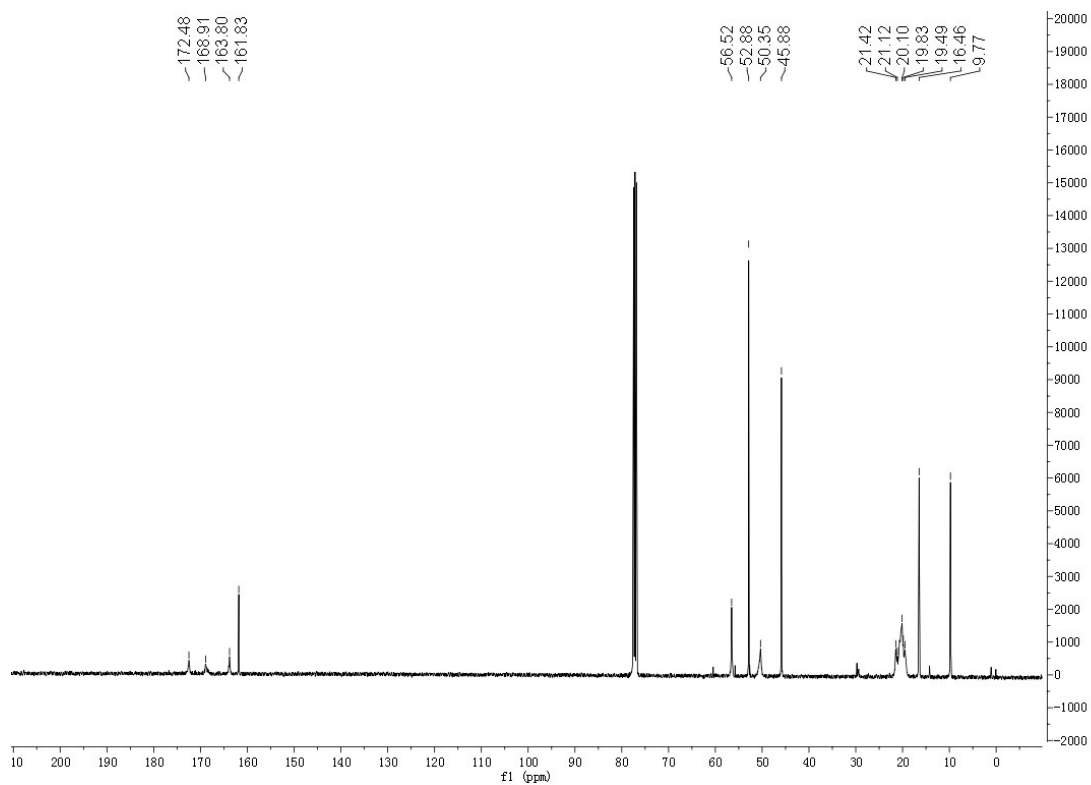

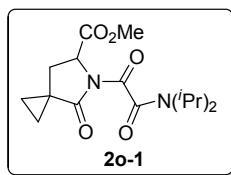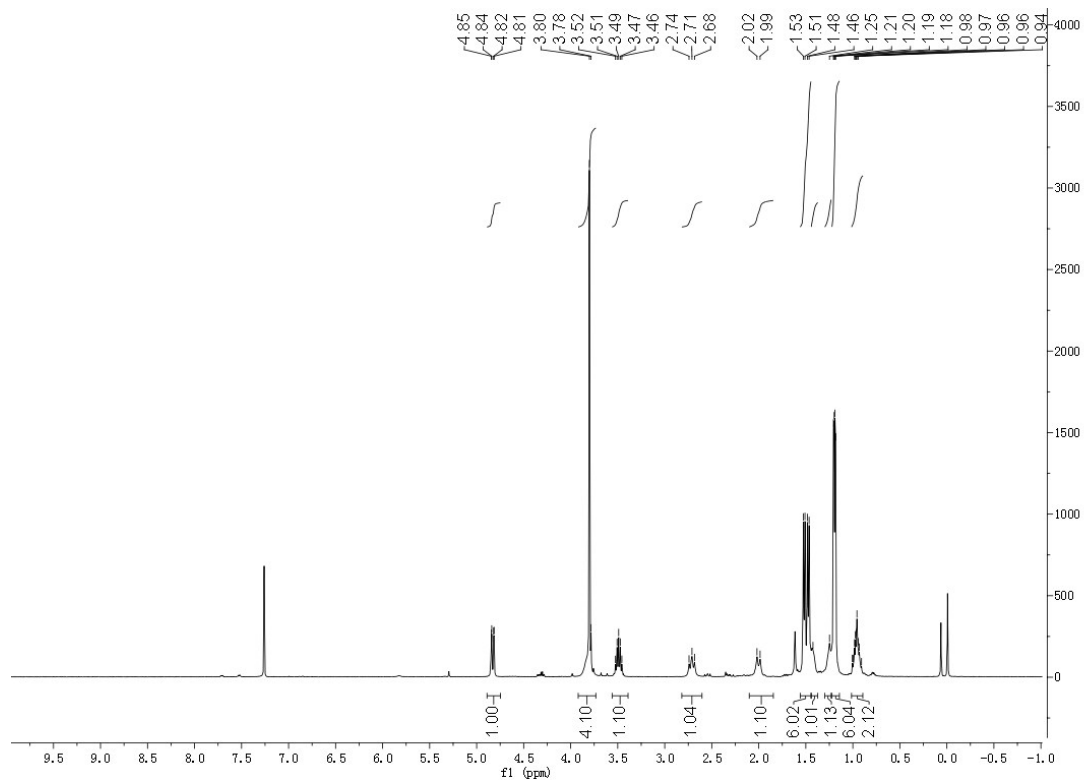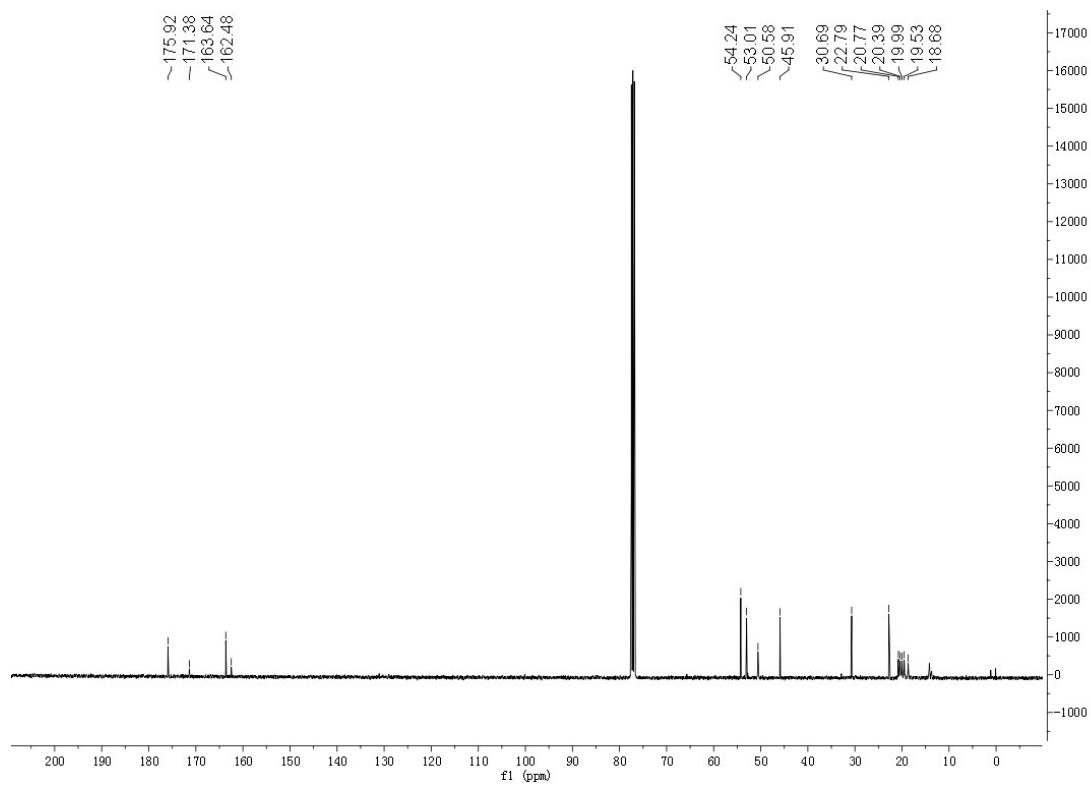

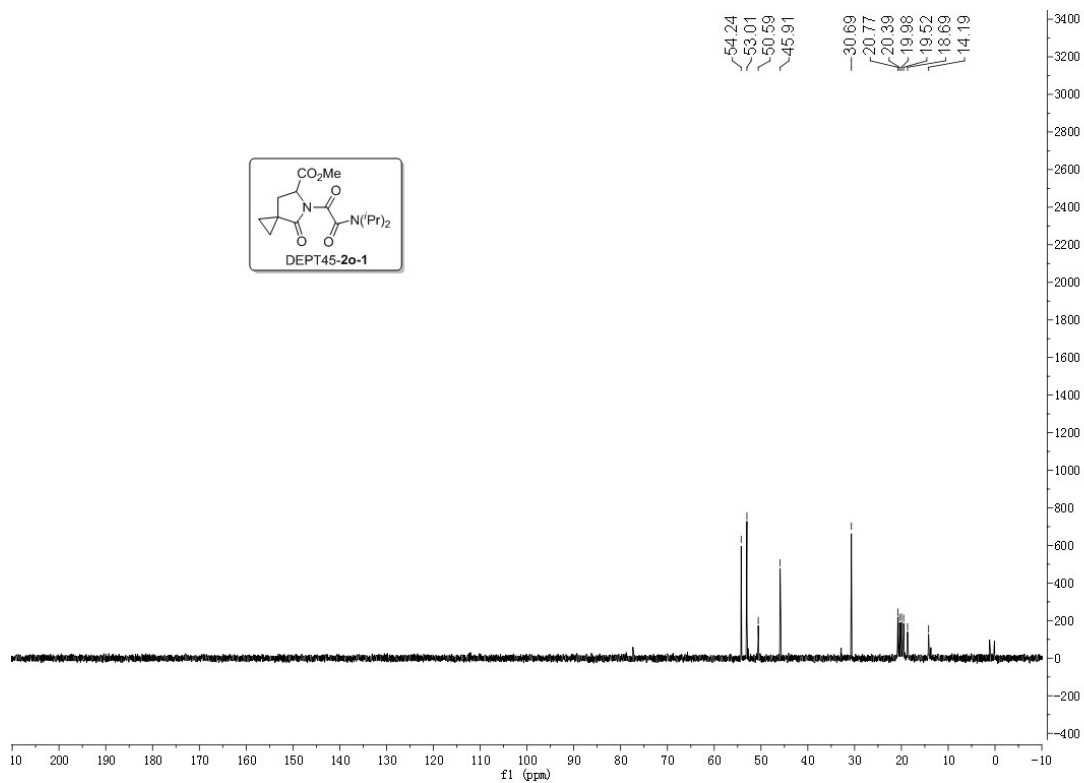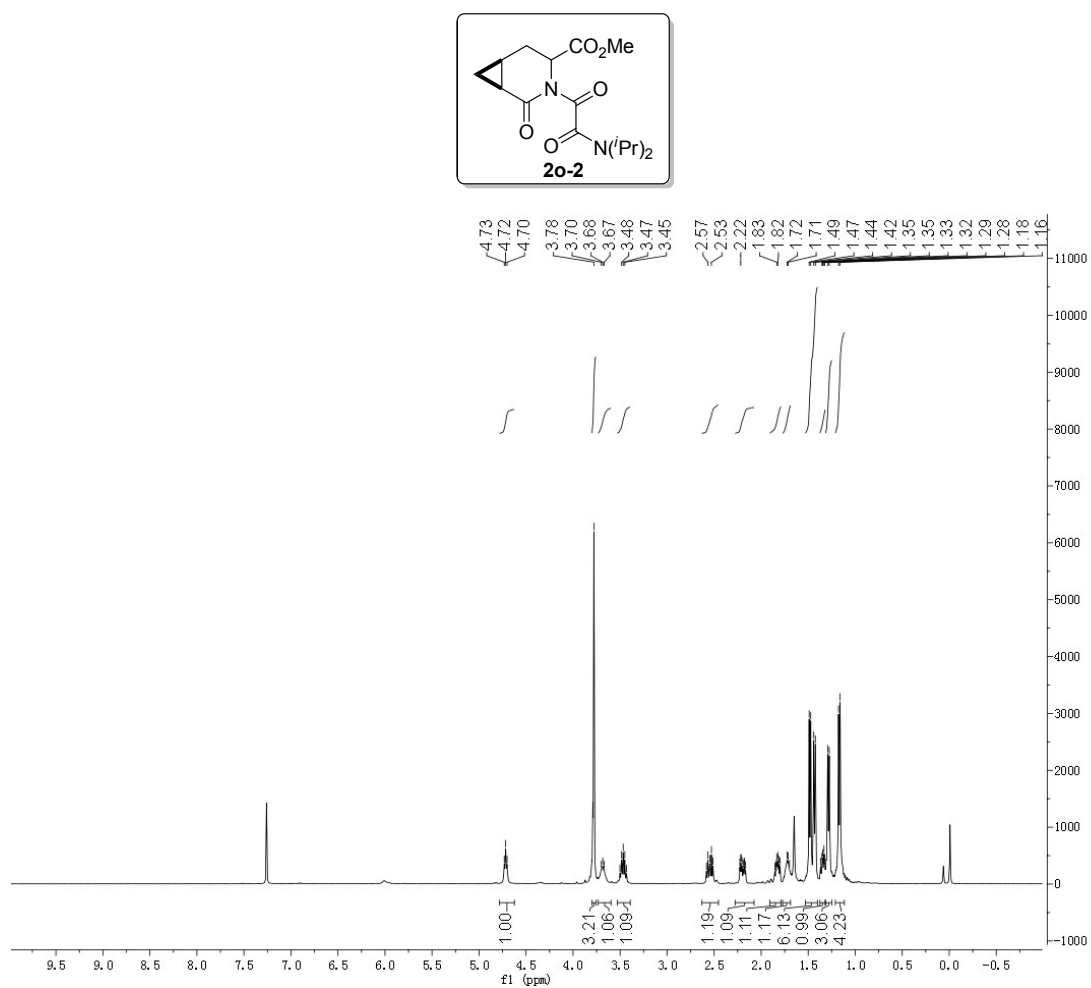

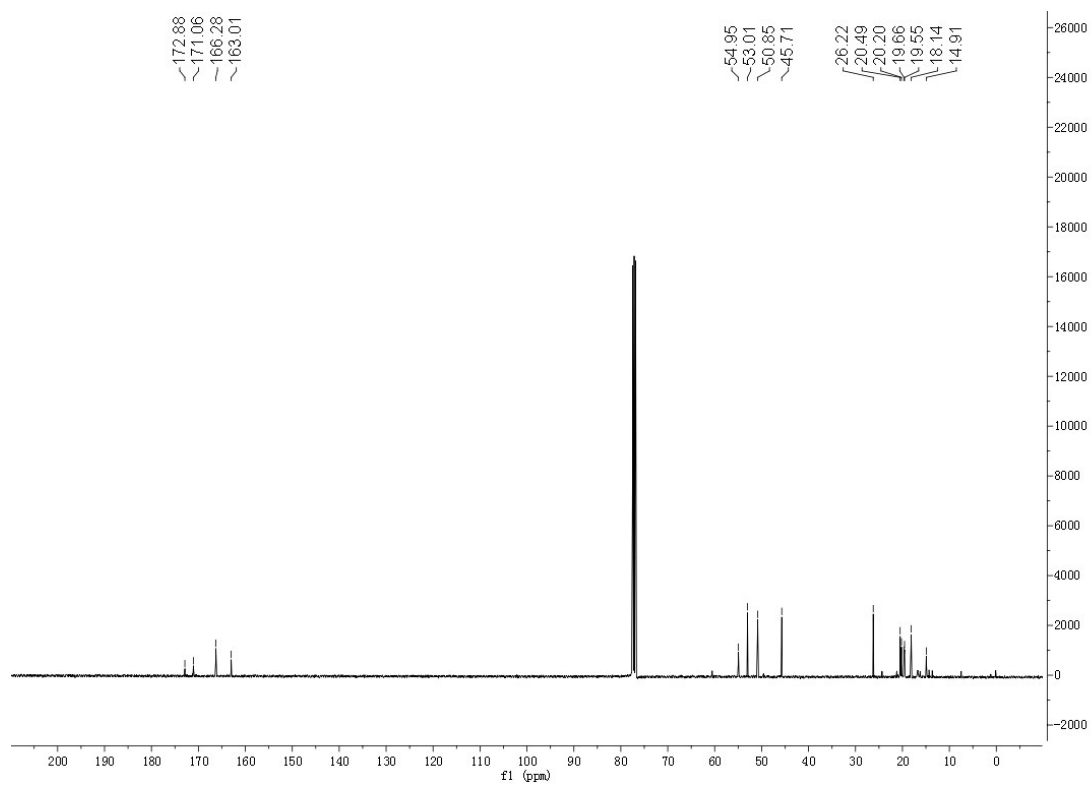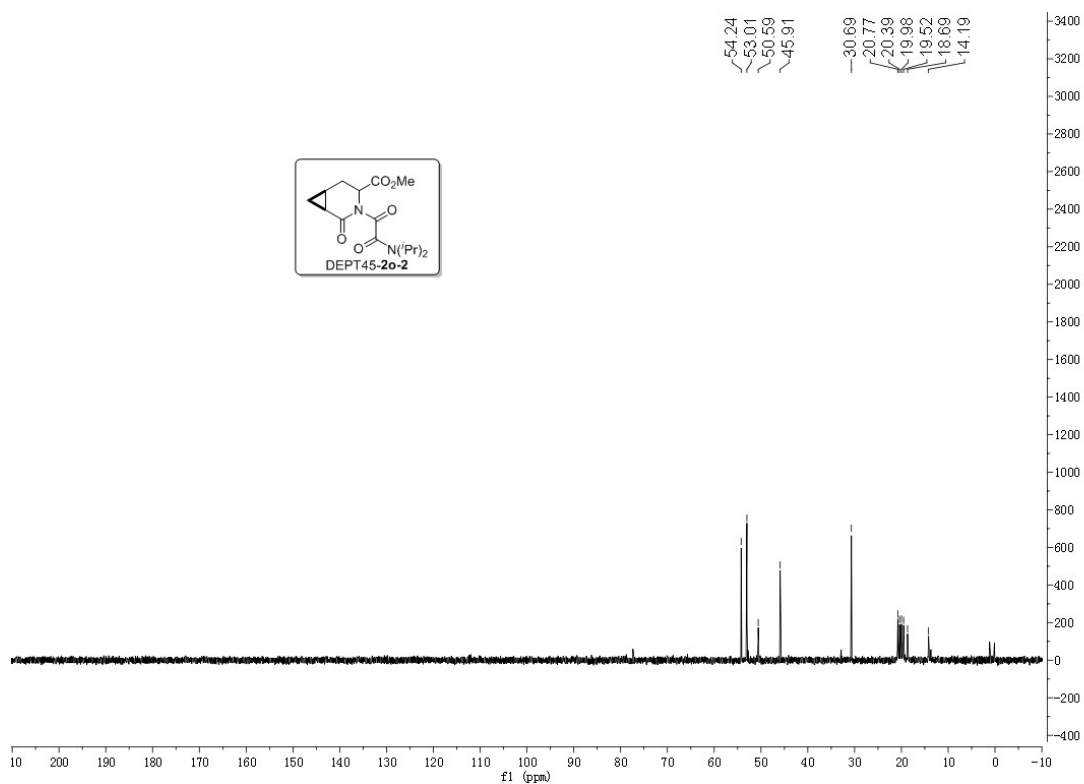

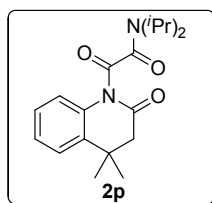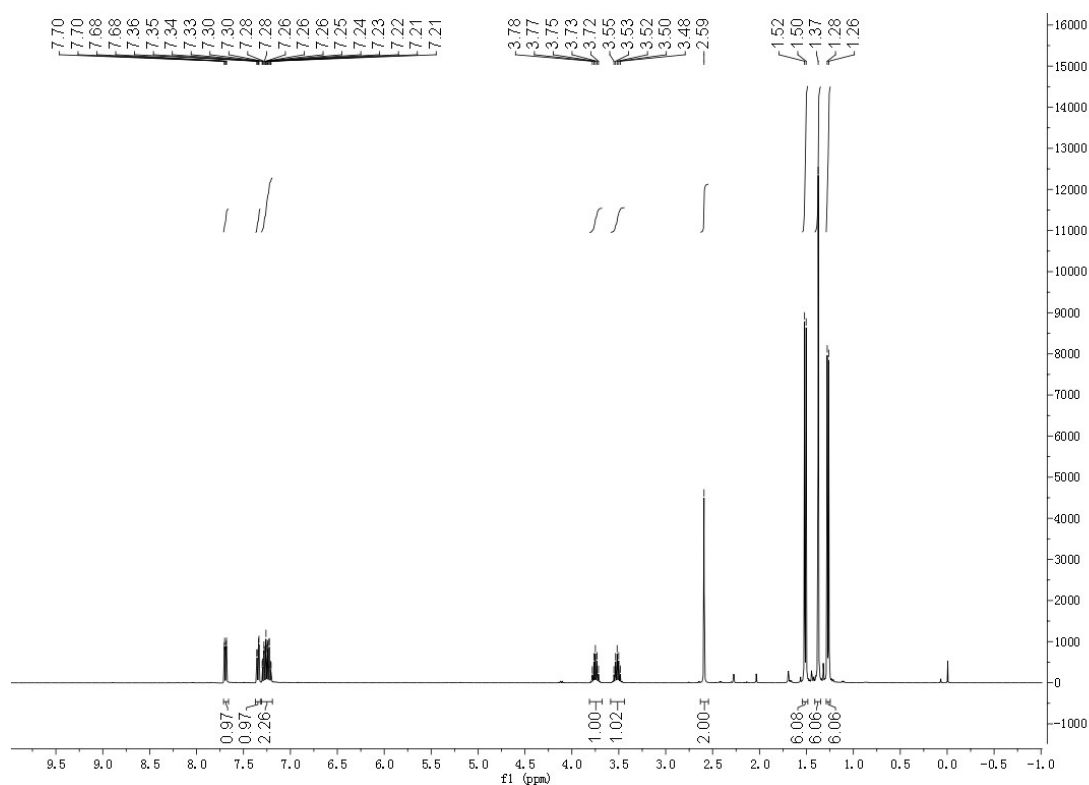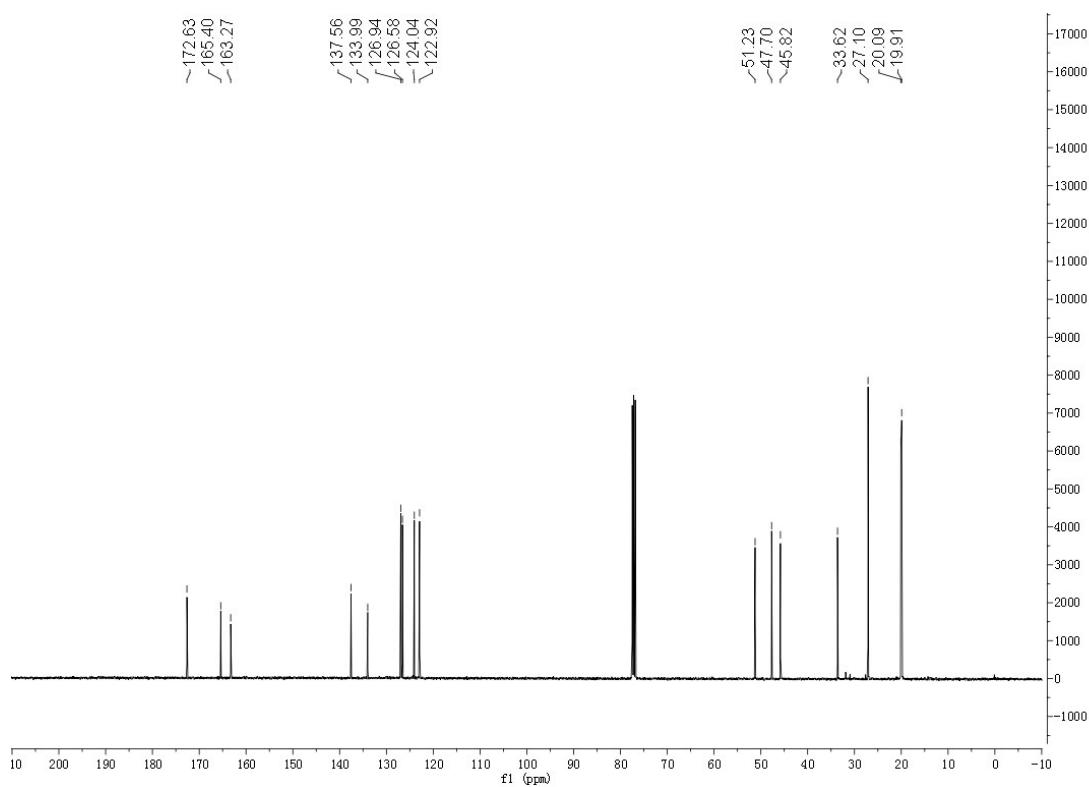

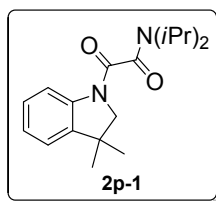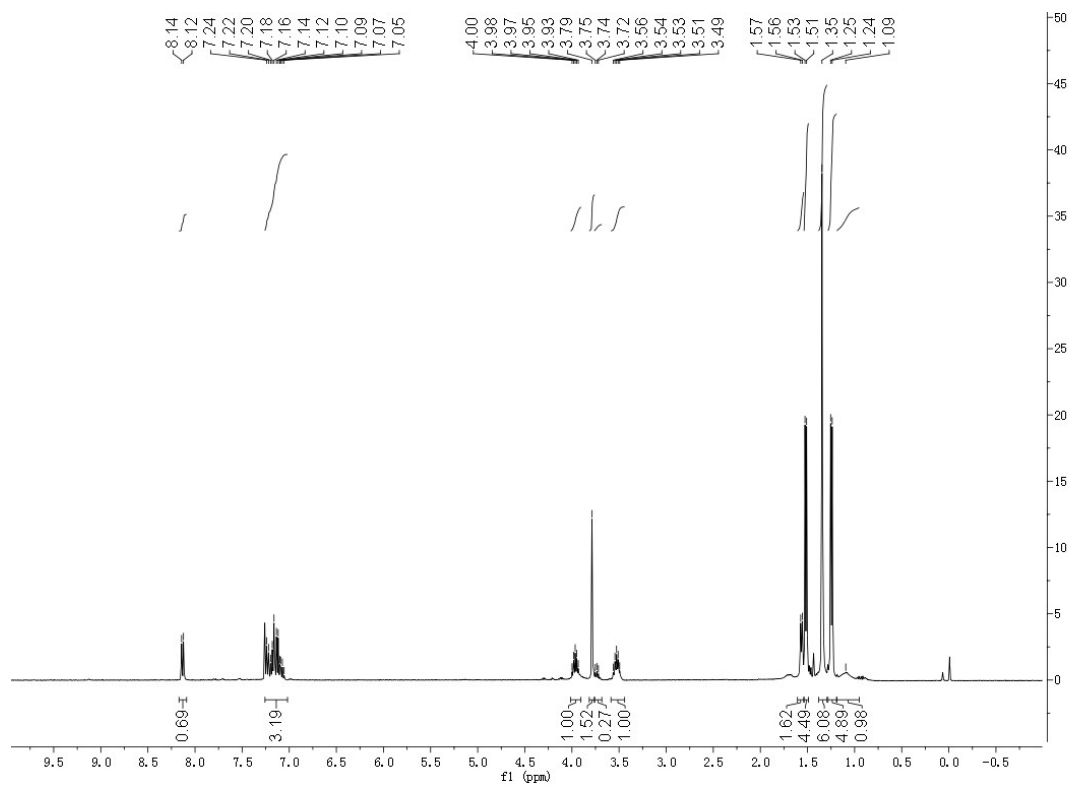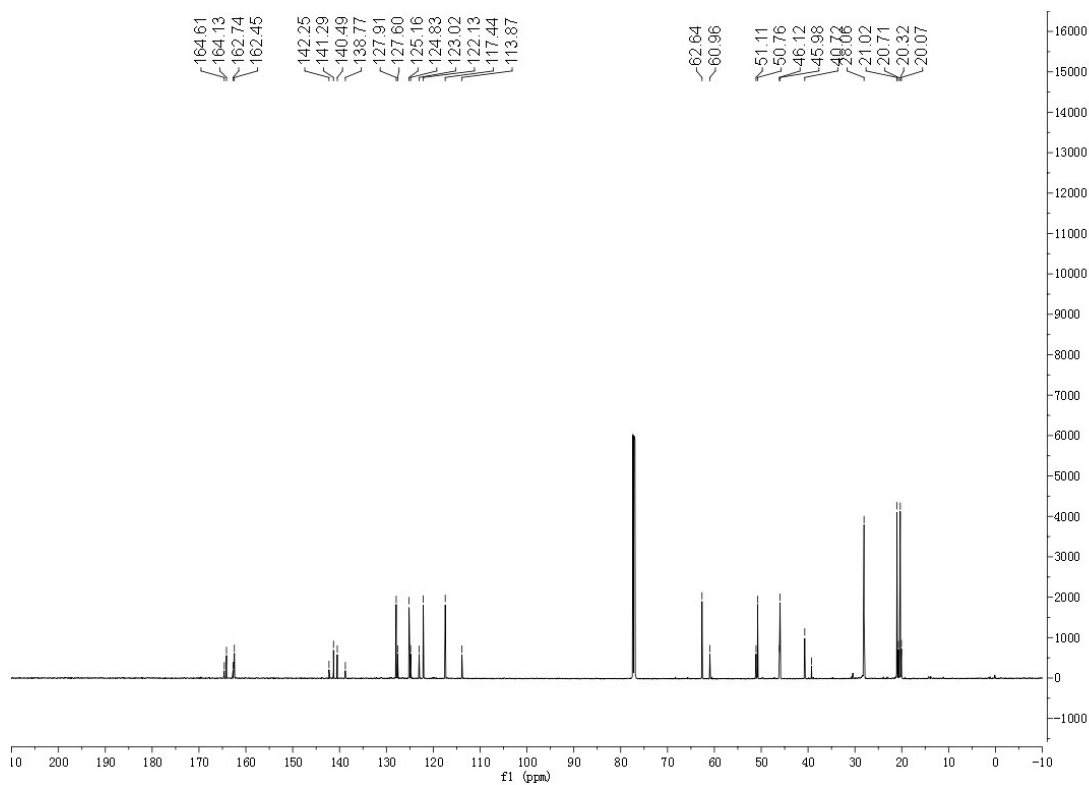

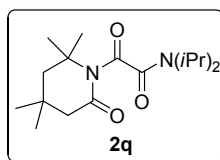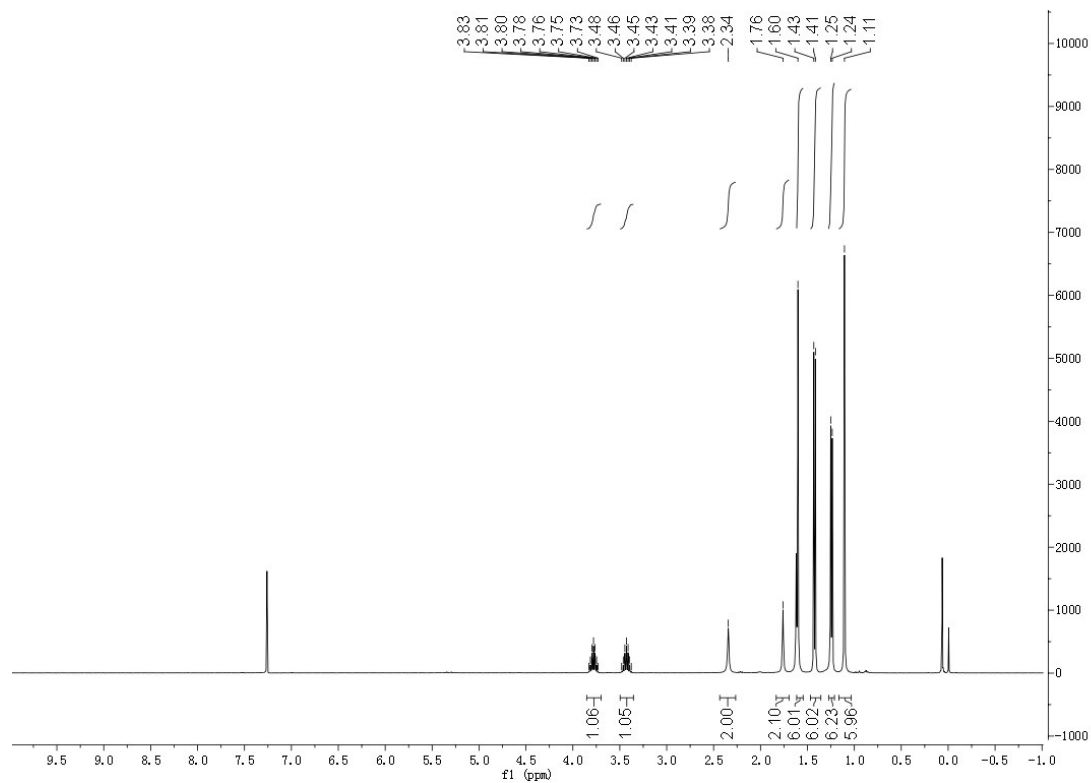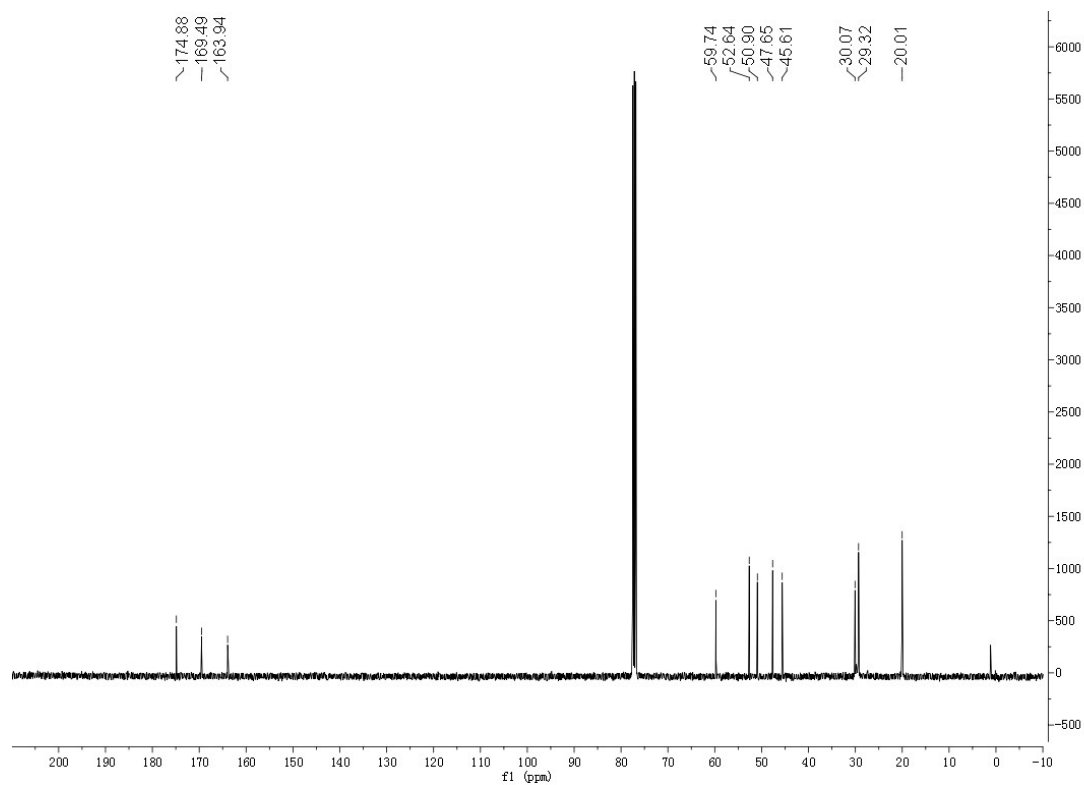

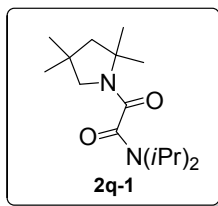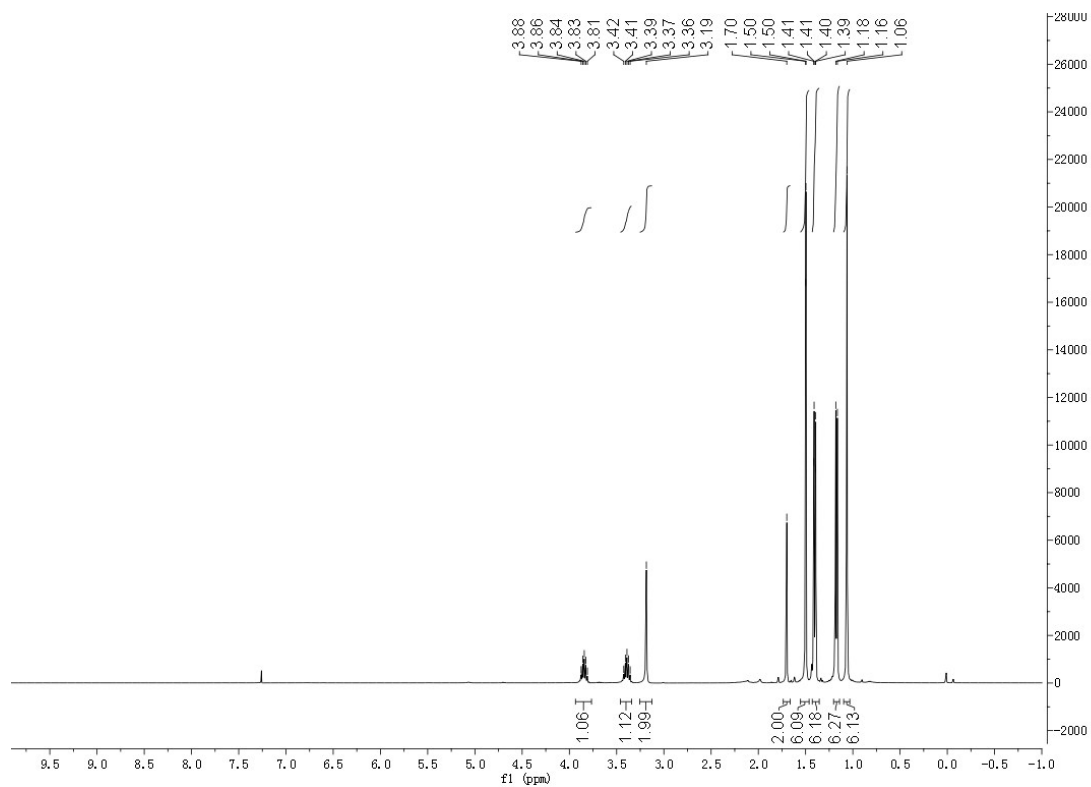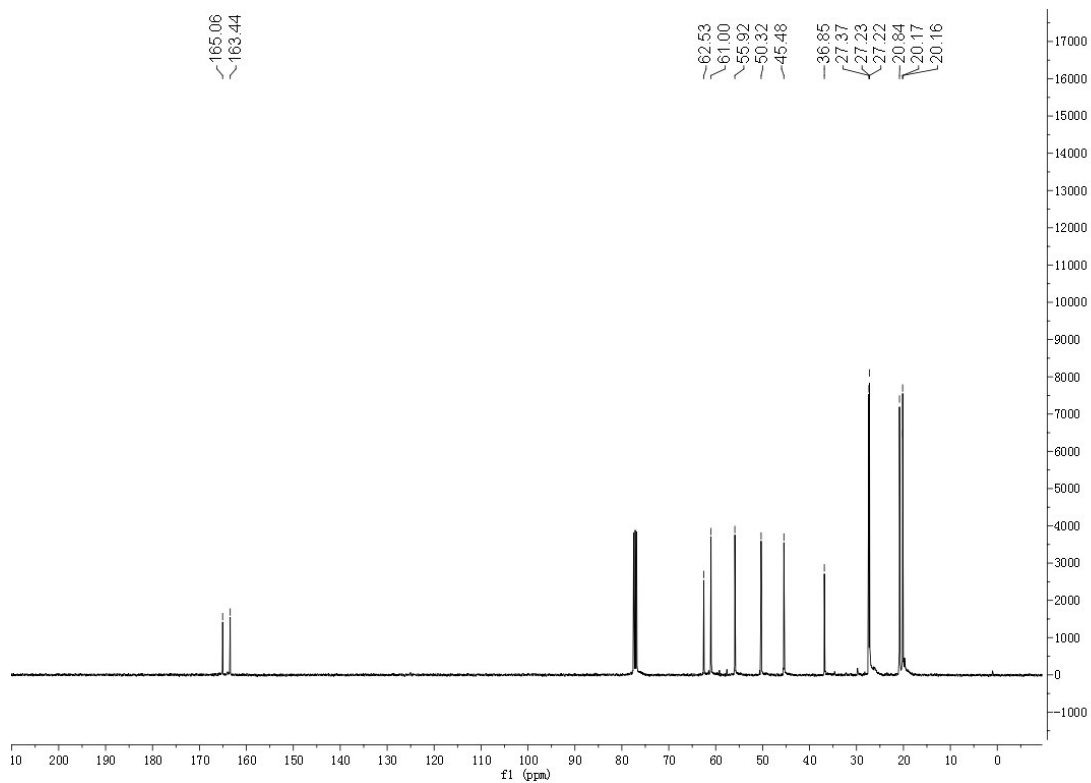

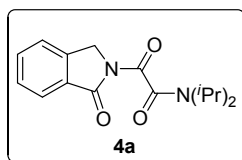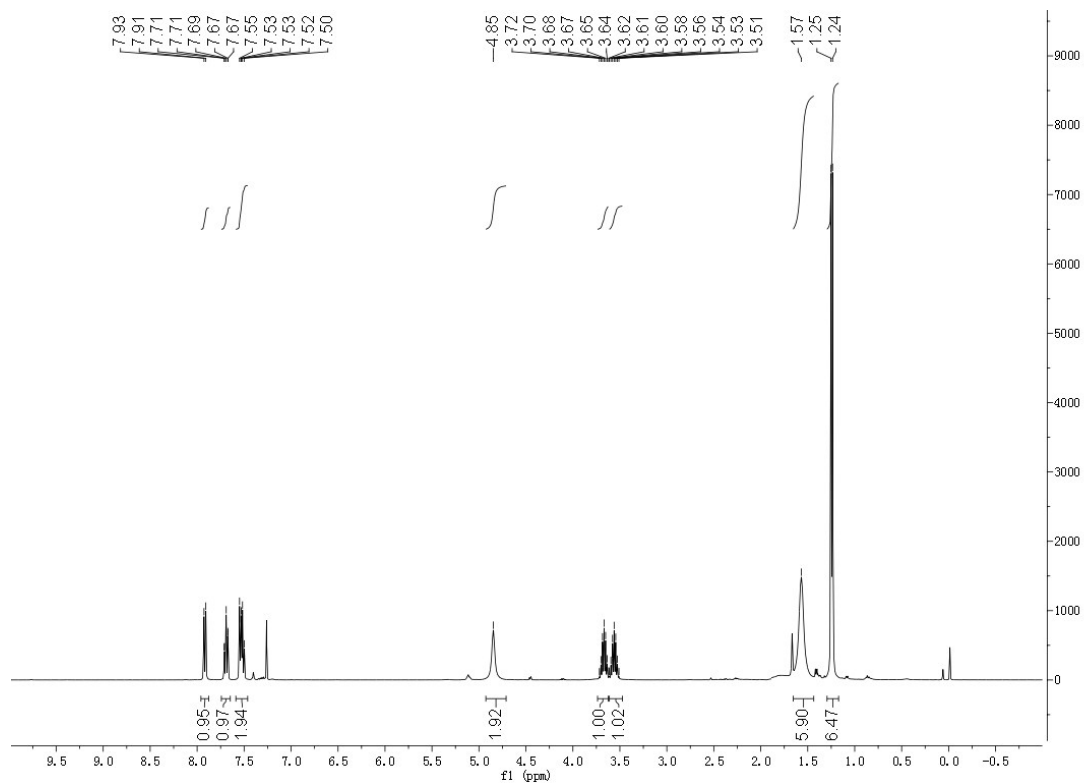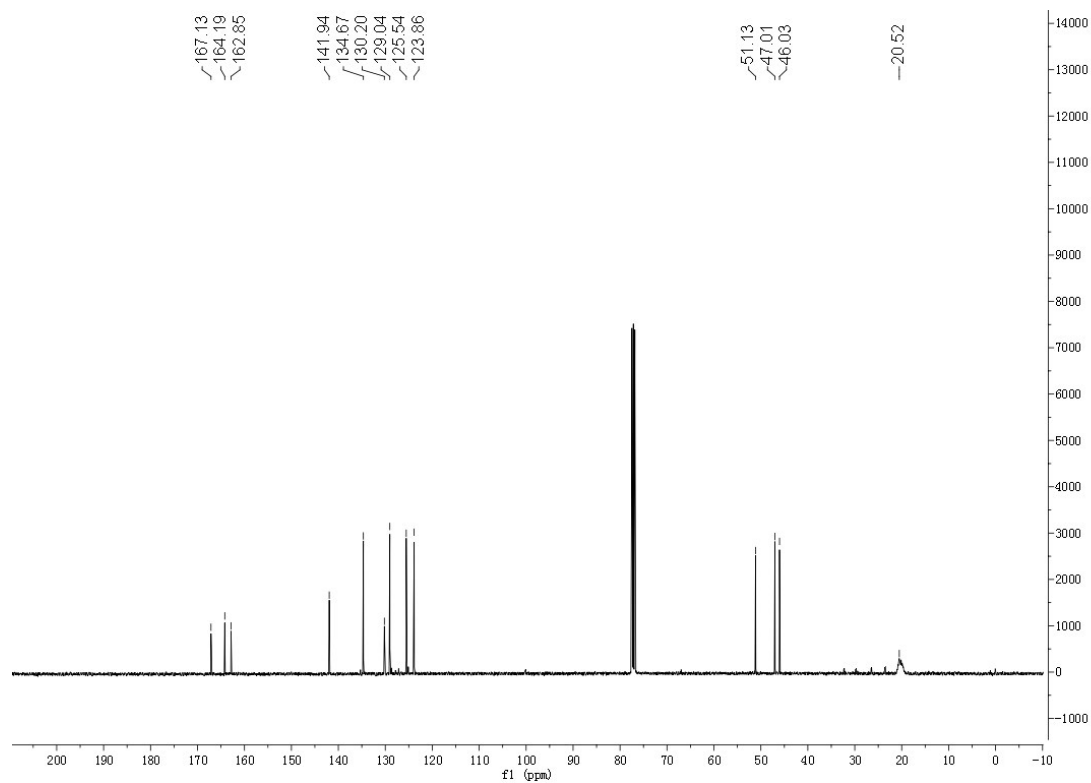

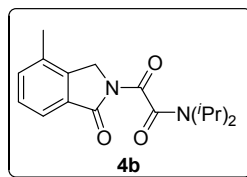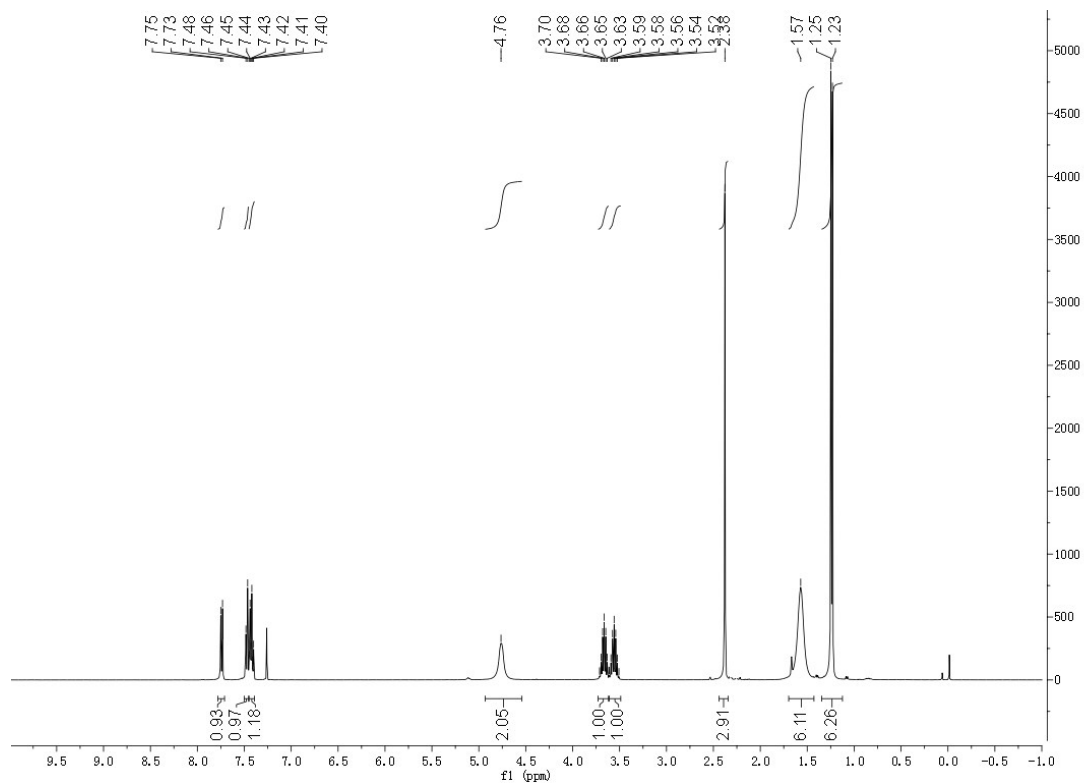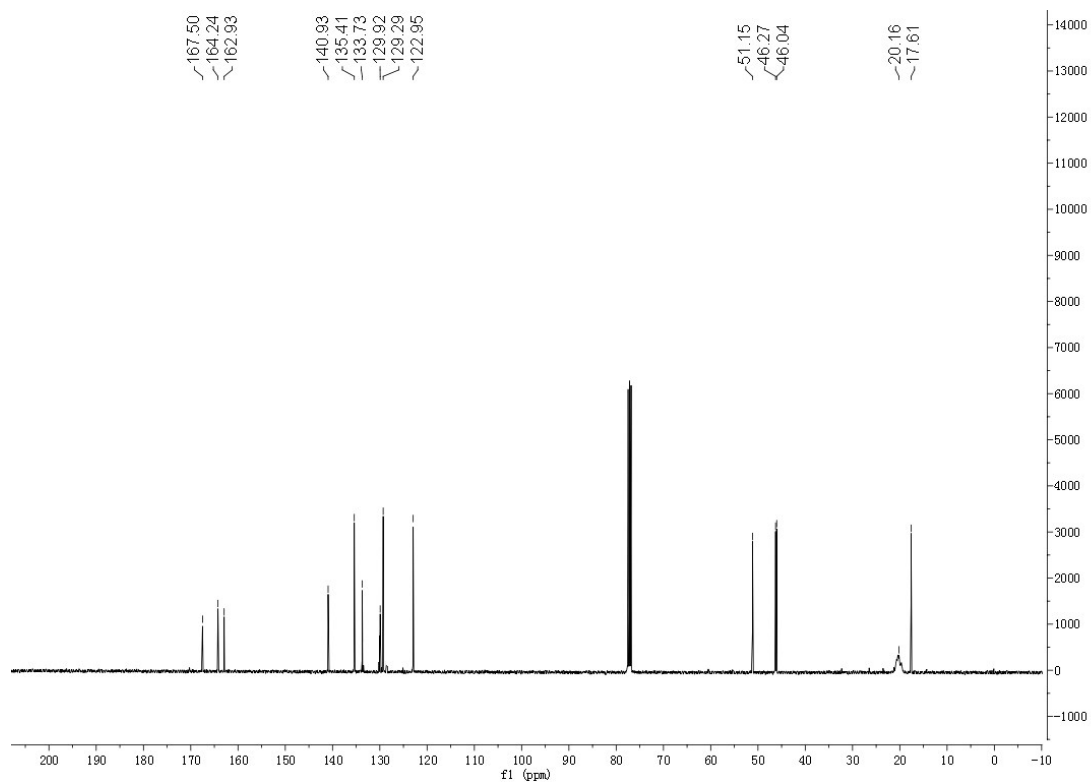

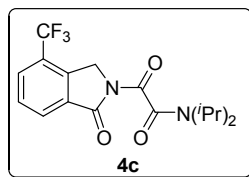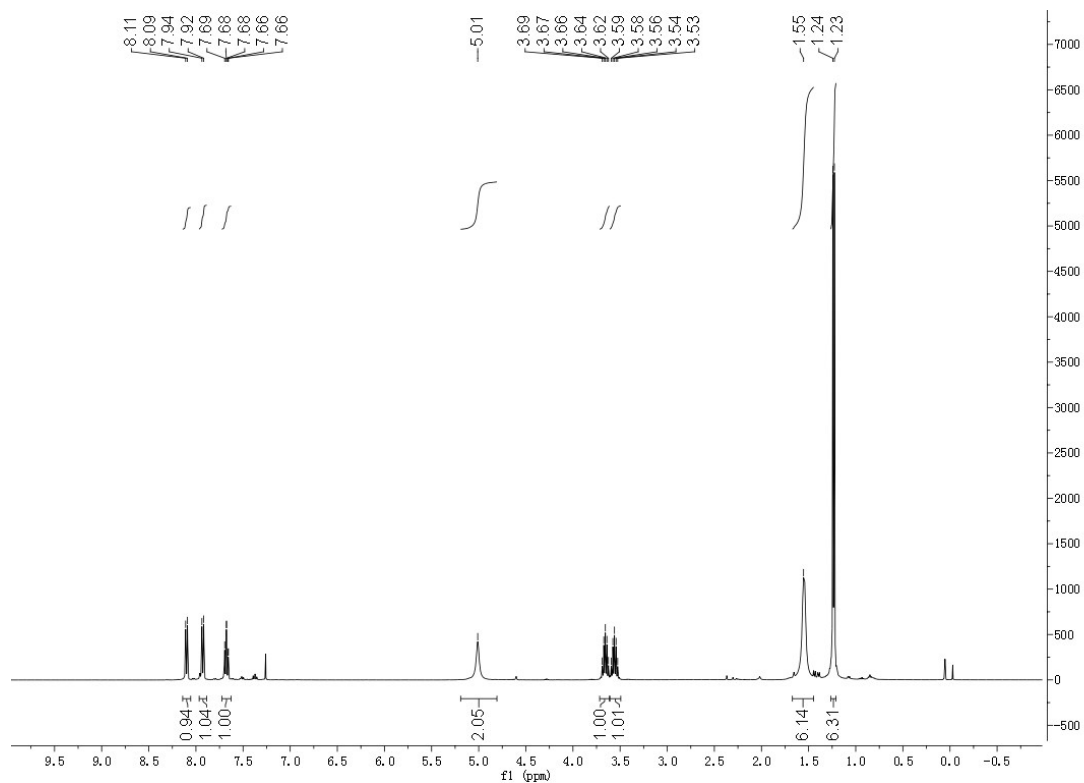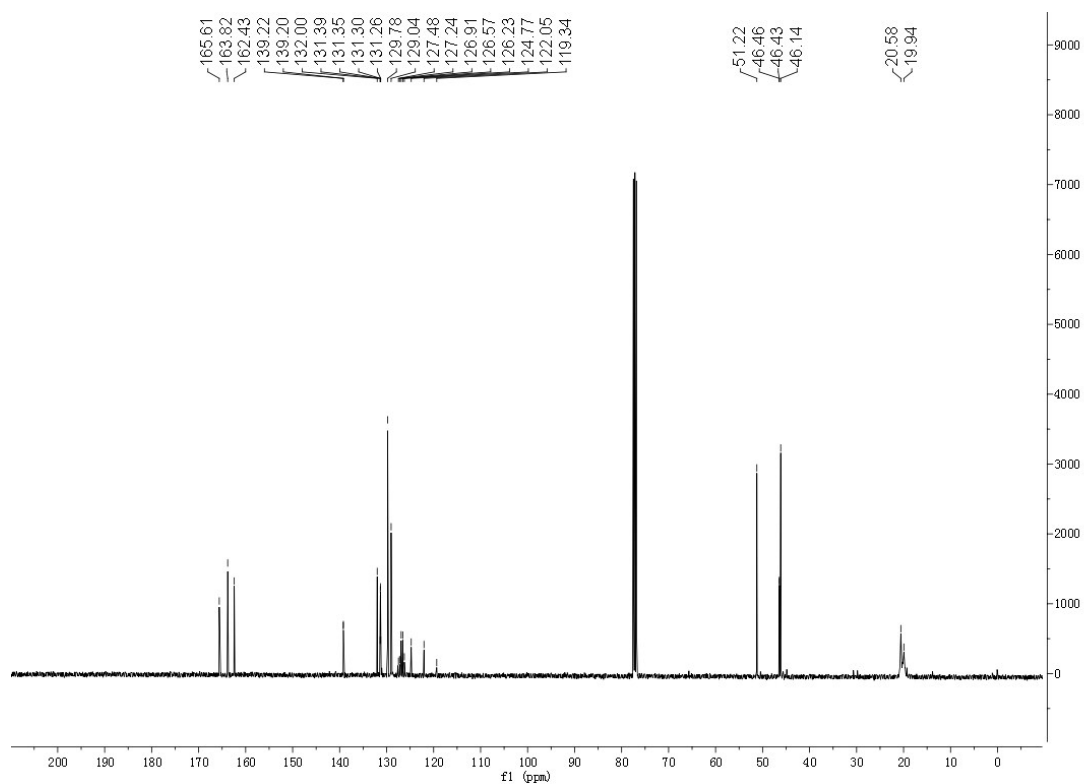

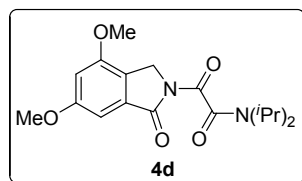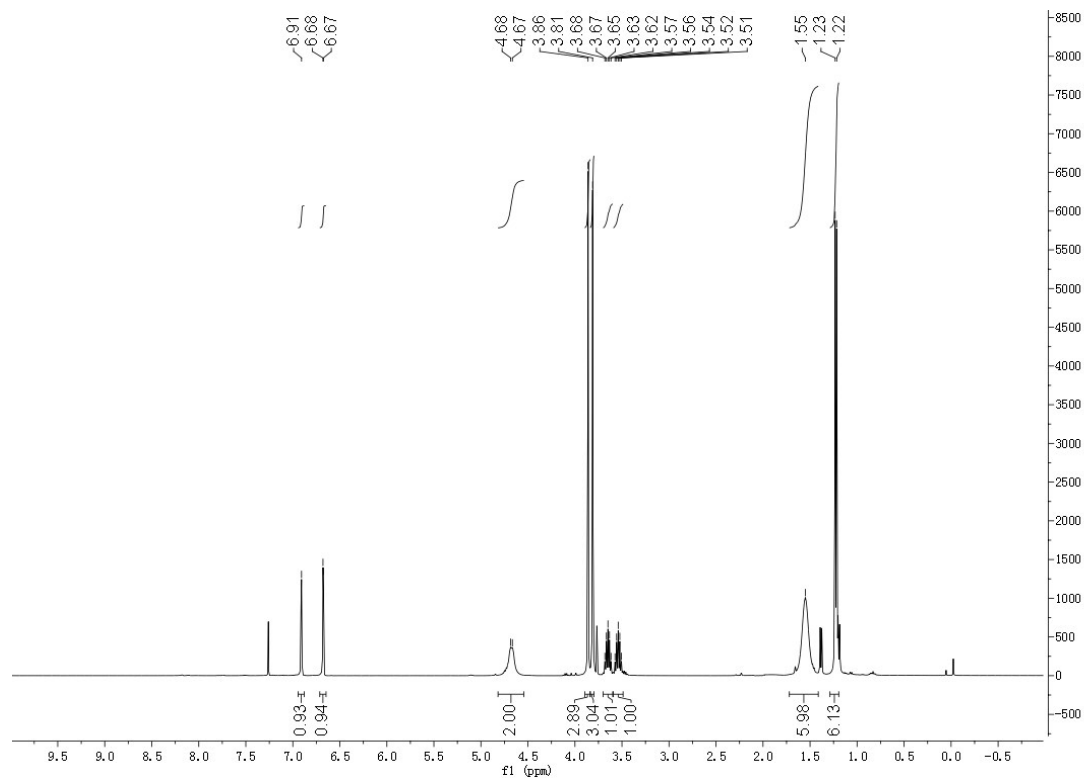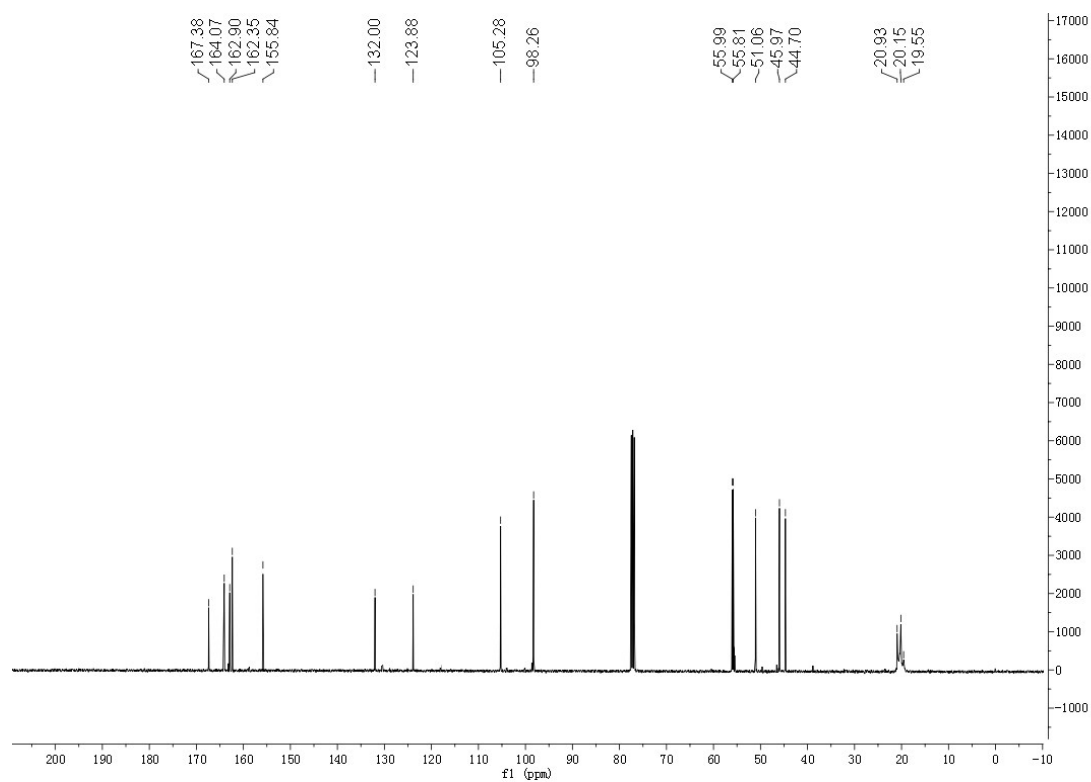

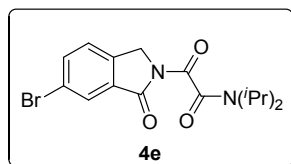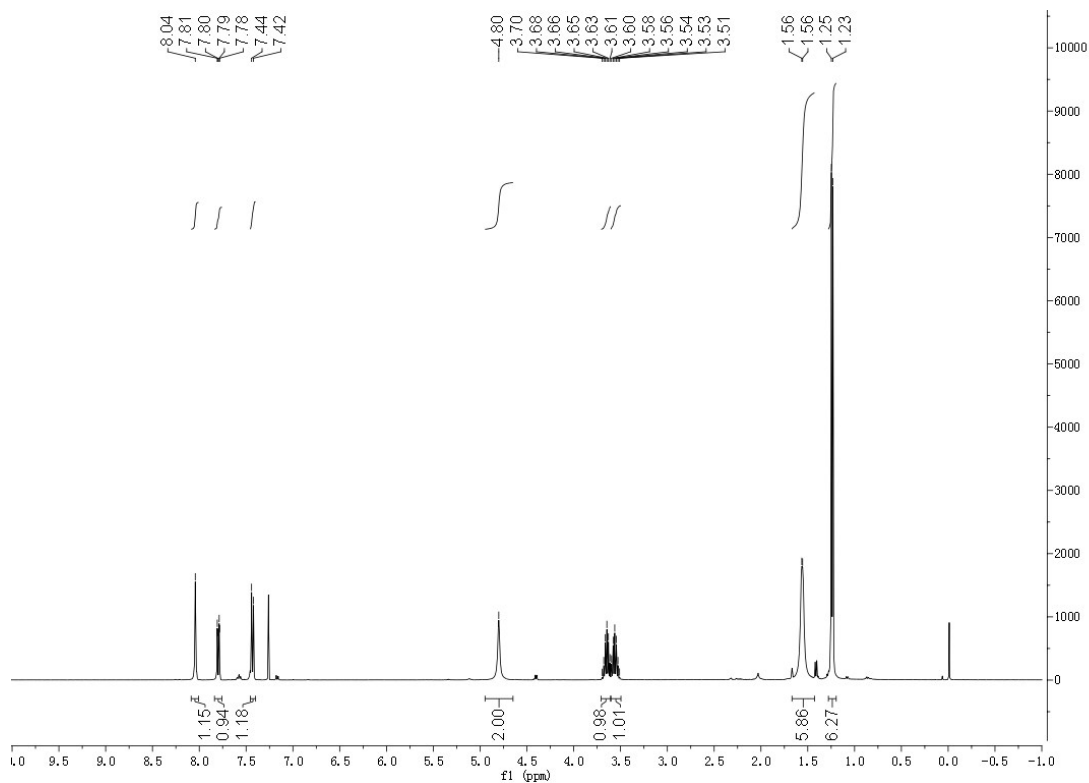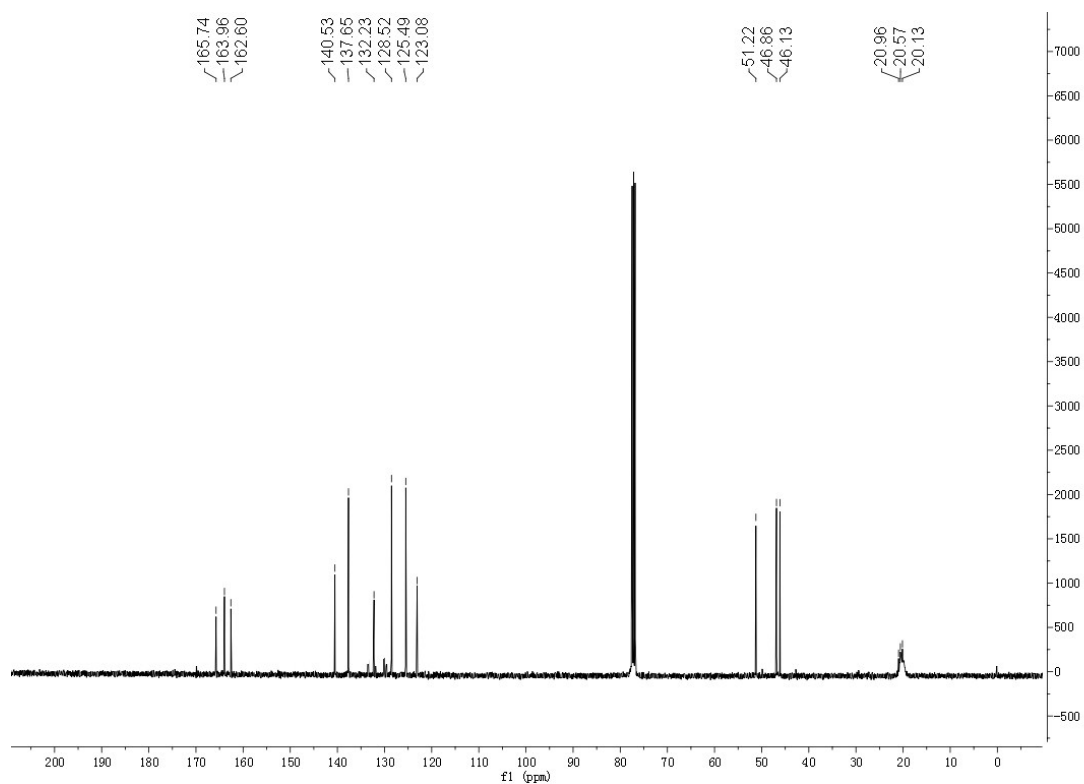

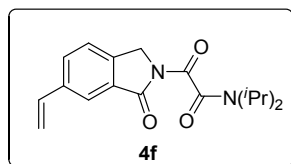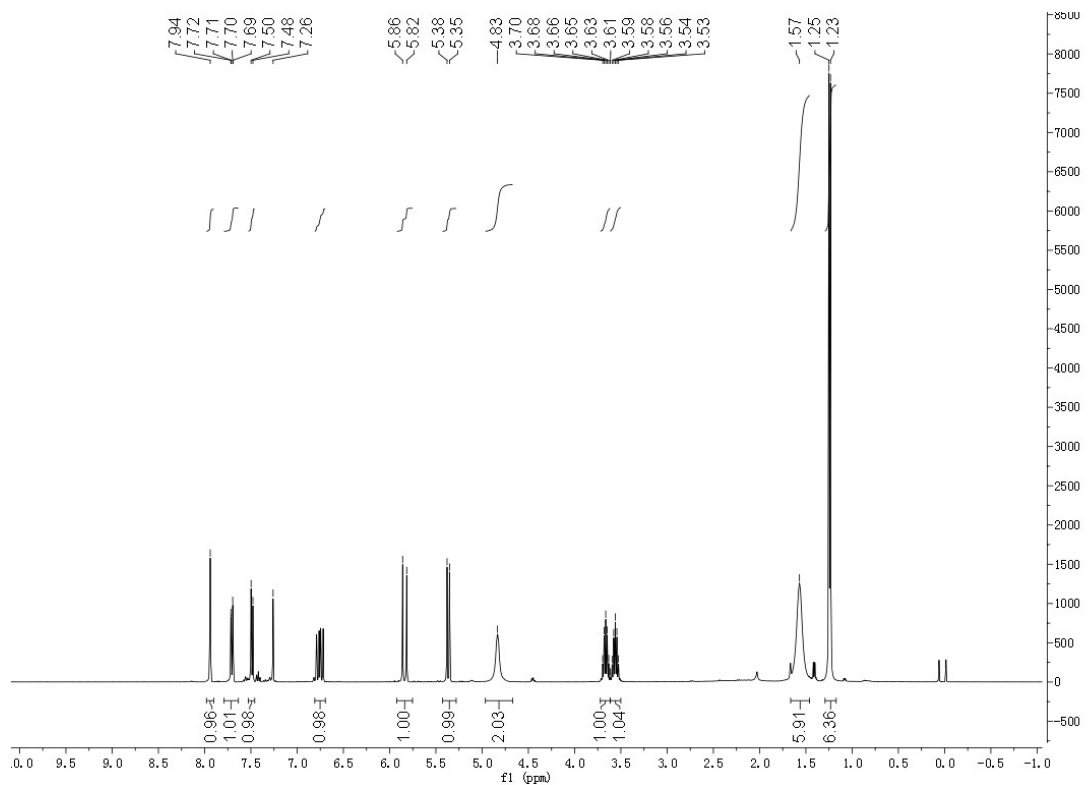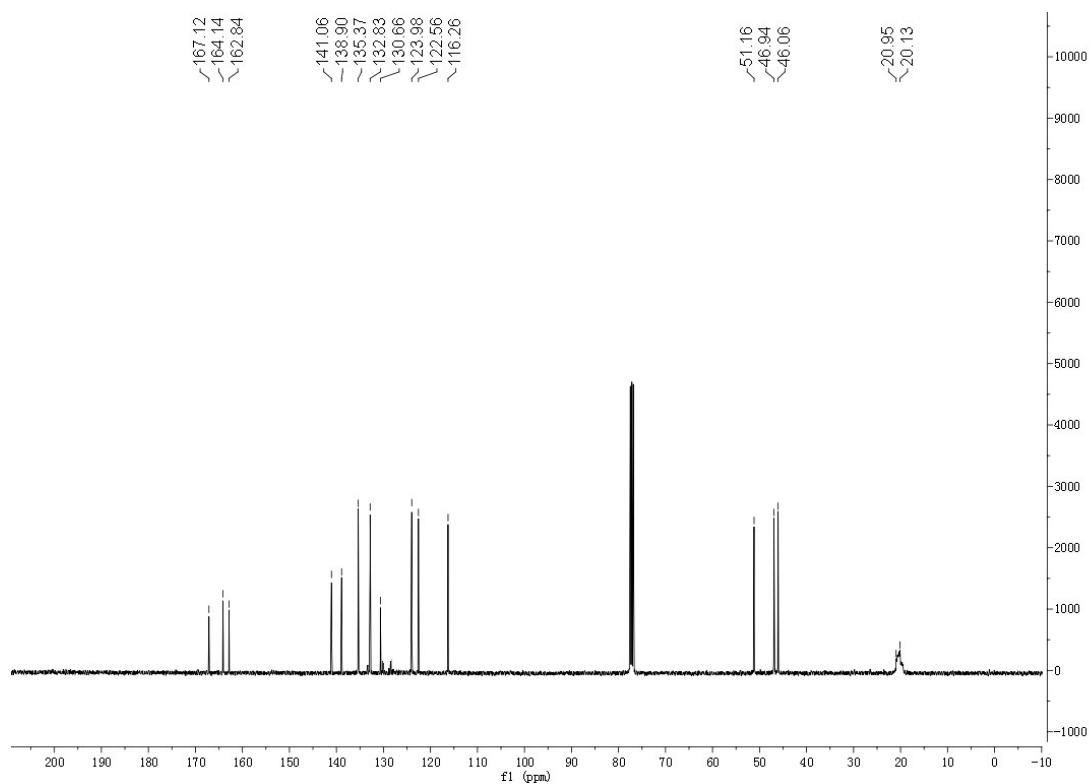

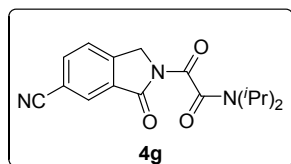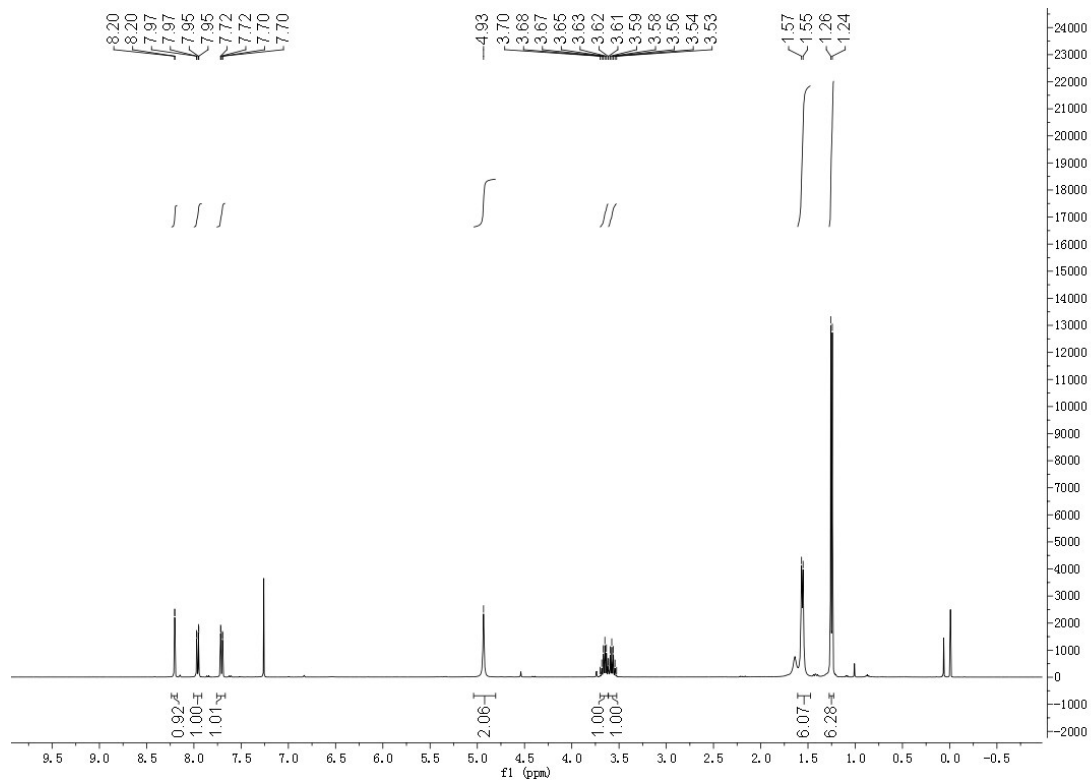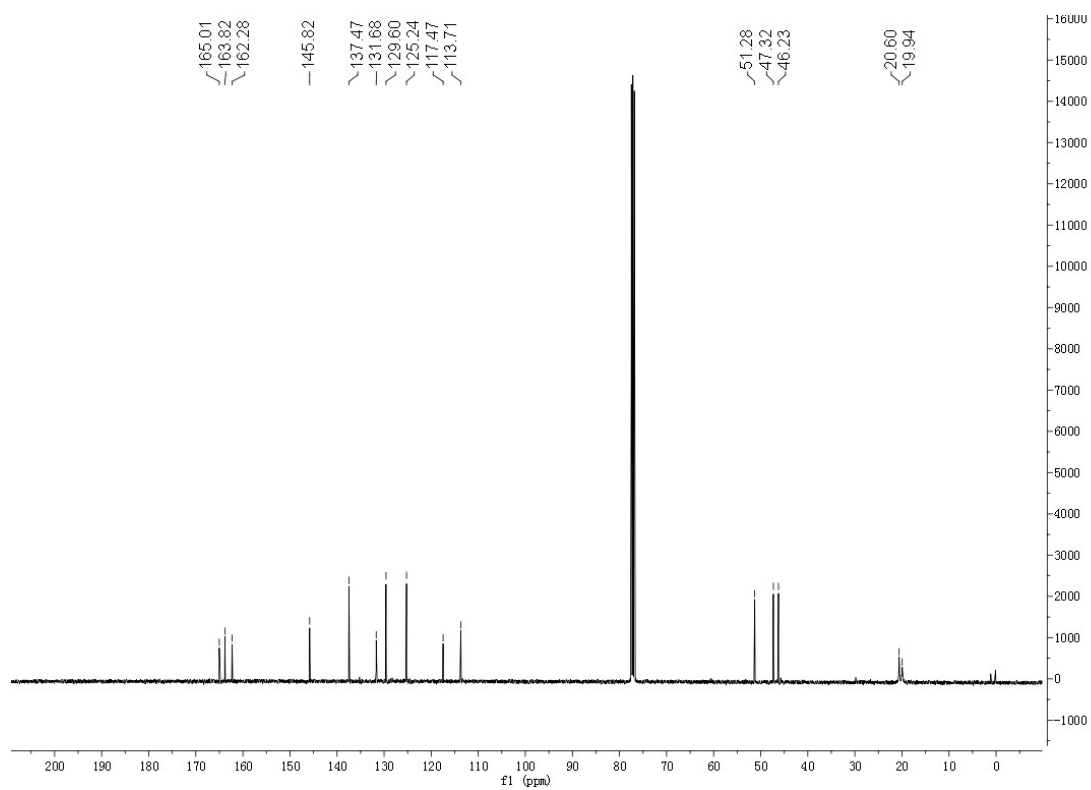

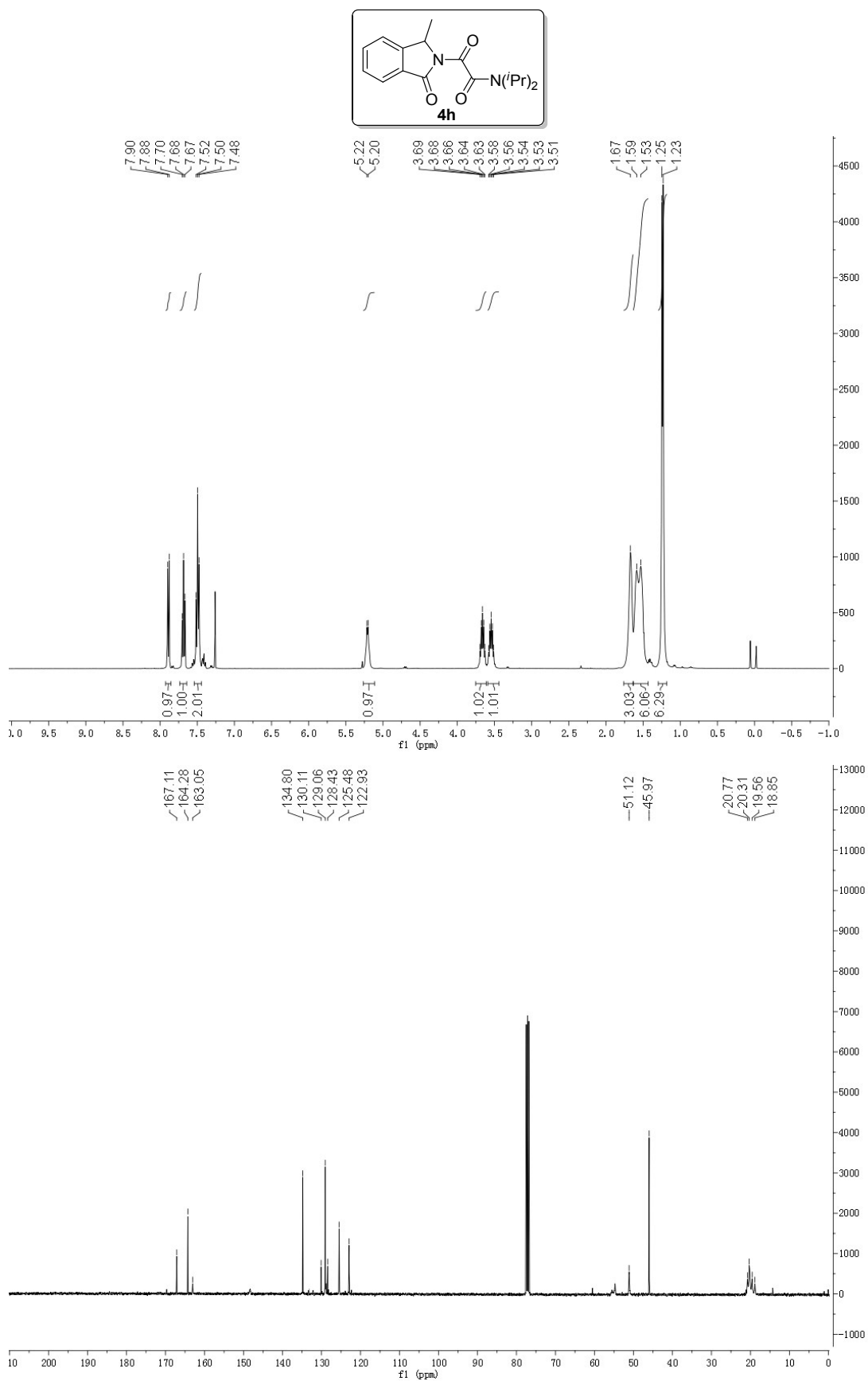

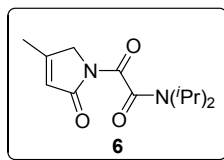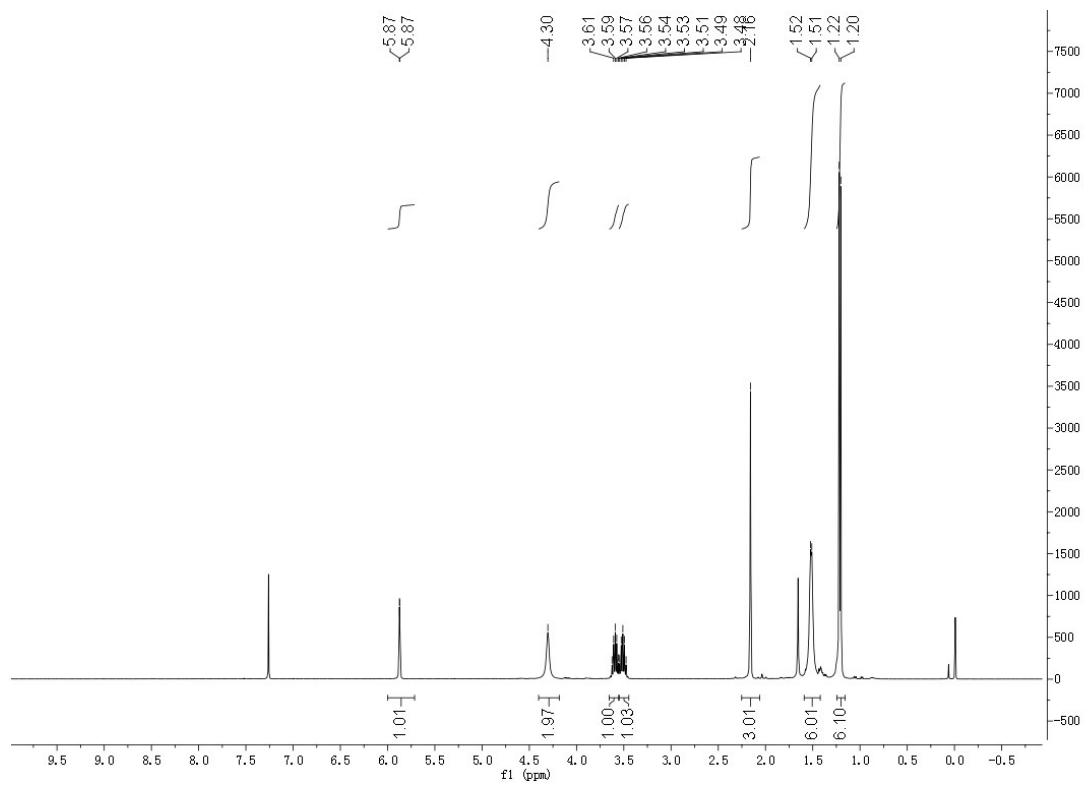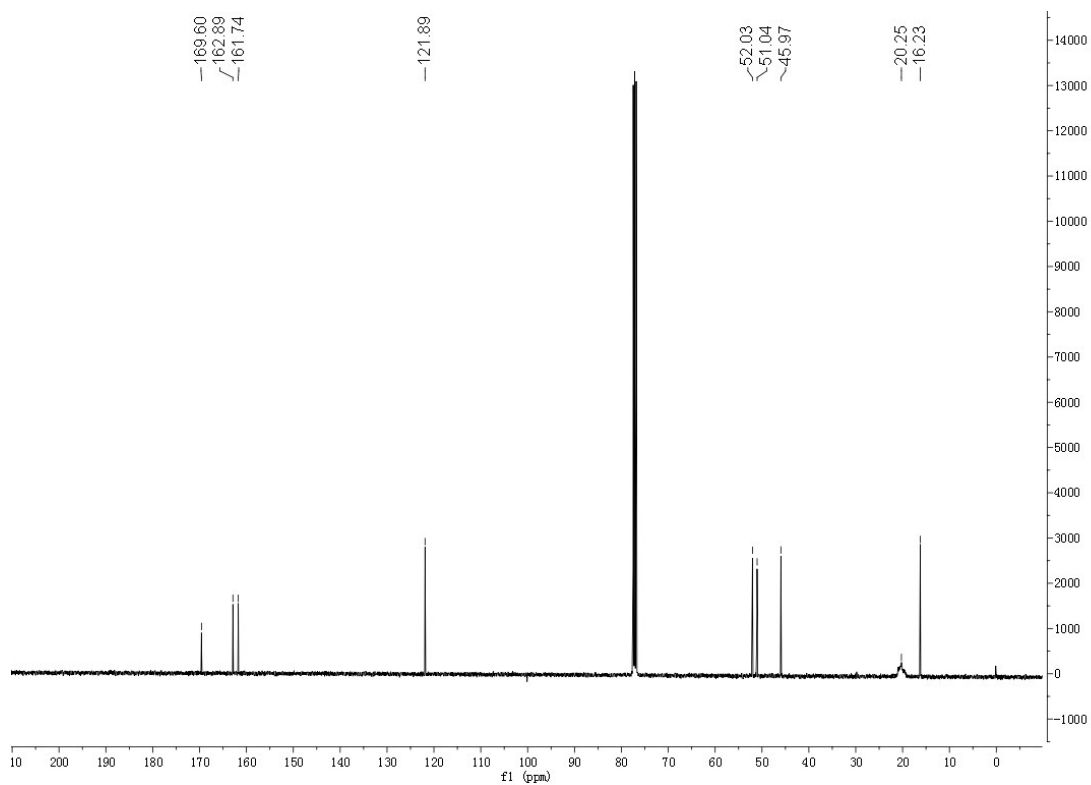

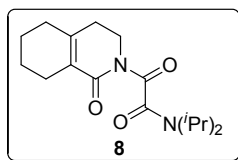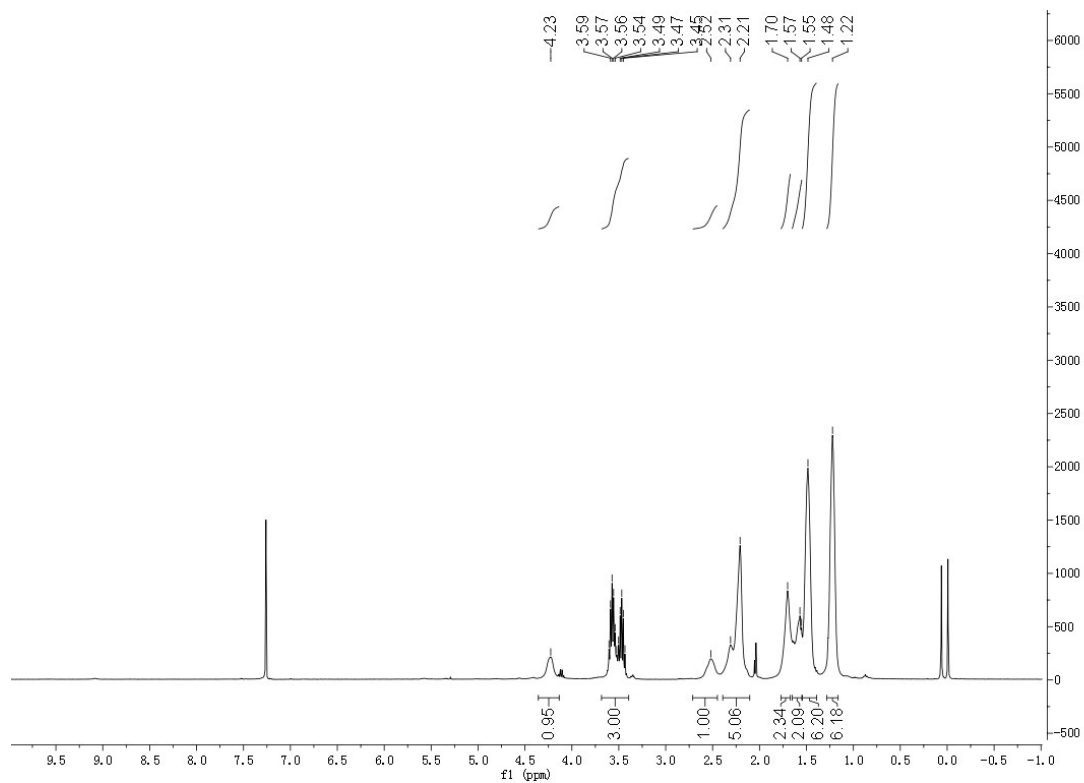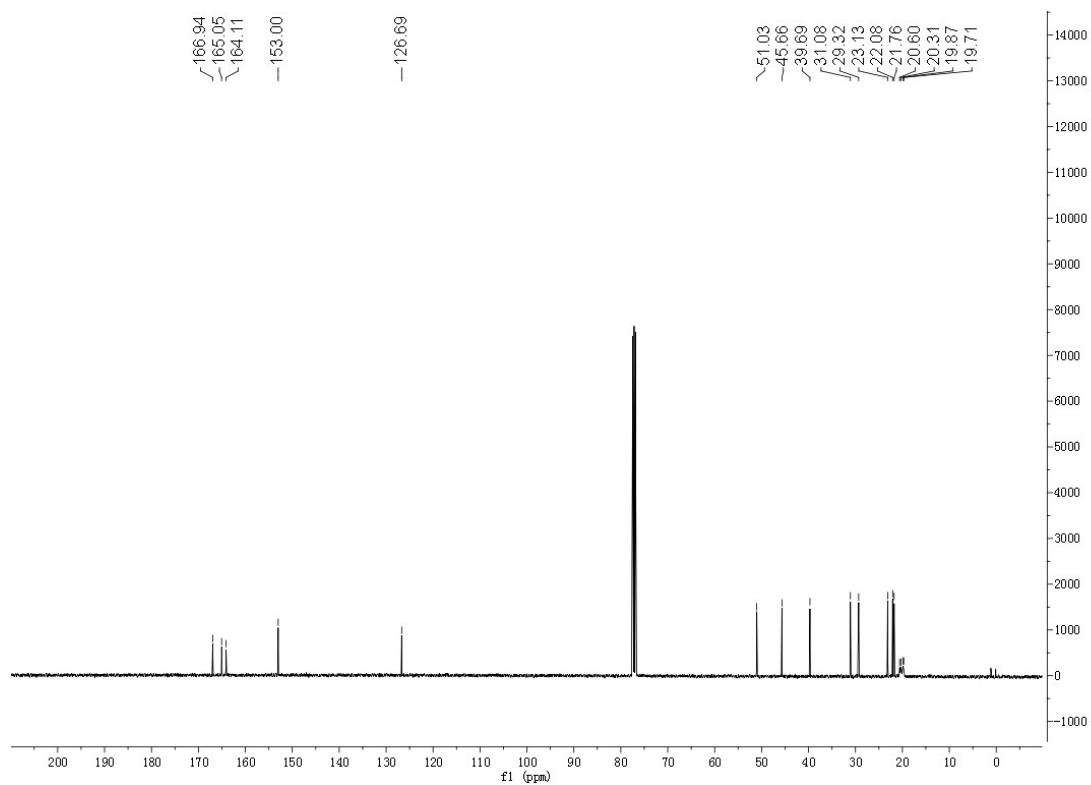

Supplement: Supplementary file 2 [file SC-006-C5SC00519A-s002.pdf]
